# Multiresidue antibiotic-metabolite quantification method using ultra-performance liquid chromatography coupled with tandem mass spectrometry for environmental and public exposure estimation

**DOI:** 10.1007/s00216-021-03573-4

**Published:** 2021-09-08

**Authors:** Elizabeth Holton, Barbara Kasprzyk-Hordern

**Affiliations:** Department of Chemistry, University of Bath, Bath, BA2 7AY UK

**Keywords:** Antibiotic, Metabolite, Liquid chromatography, Mass spectrometry, Wastewater-based epidemiology, Water fingerprinting

## Abstract

This manuscript describes a new multiresidue method utilising ultra-performance liquid chromatography tandem mass spectrometry (UPLC-MS/MS) via multiple reaction monitoring (MRM), for the identification and quantification of 58 antibiotics and their 26 metabolites, in various solid and liquid environmental matrices. The method was designed with a ‘one health’ approach in mind requiring multidisciplinary and multisectoral collaborative efforts. It enables comprehensive evaluation of antibiotic usage in surveyed communities via wastewater-based epidemiology, as well as allowing for the assessment of potential environmental impacts. The instrumental performance was very good, demonstrating linearity up to 3000 μg L^−1^, and high accuracy and precision. The method accuracy in several compounds was significantly improved by dividing calibration curves into separate ranges. This was accompanied by applying a weighting factor (1/*x*). Microwave-assisted and/or solid-phase extraction of analytes from liquid and solid matrices provided good recoveries for most compounds, with only a few analytes underperforming. Method quantification limits were determined as low as 0.017 ng L^−1^ in river water, 0.044 ng L^−1^ in wastewater, 0.008 ng g^−1^ in river sediment, and 0.009 ng g^−1^ in suspended solids. Overall, the method was successfully validated for the quantification of 64 analytes extracted from aqueous samples, and 45 from solids. The analytes that underperformed are considered on a semi-quantitative basis, including aminoglycosides and carbapenems. The method was applied to both solid and liquid environmental matrices, whereby several antibiotics and their metabolites were quantified. The most notable antibiotic-metabolite pairs are three sulfonamides and their N-acetyl metabolites; four macrolides/lincomycins and their N-desmethyl metabolites; and five quinolone metabolites.

**Graphical abstract:**

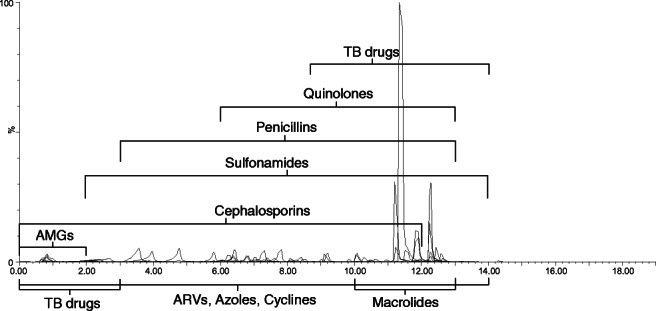

**Supplementary Information:**

The online version contains supplementary material available at 10.1007/s00216-021-03573-4.

## Introduction

Antimicrobial resistance (AMR) is a major global topic, concerning the increasing pathogenic tolerance to antibiotics. Excessive and inappropriate antibiotic use heightens the emergence of antimicrobial resistance genes in pathogenic organisms, resulting in reduced drug susceptibility. Water fingerprinting or wastewater-based epidemiology (WBE) techniques offer an innovative approach for tracking markers of disease and AMR, such as antimicrobials, pollutants, pathogenic DNA, and resistance genes. WBE is a relatively new field of research. It has been used to estimate illicit drug and lifestyle chemical usage trends [1–4], community-wide exposure to pesticides [5, 6], industrial chemicals (e.g. BPA or perfluorinated chemicals) [7–9], or mycotoxins [10]. Moreover, with the advent of the SARS-CoV-2 coronavirus pandemic at the end of 2019 (COVID-19), WBE has been applied globally to track community infection with SAR-CoV-2 [11, 12]. However, there is very limited published research on antibiotics and AMR [13, 14]. WBE has a significant potential to determine the spatiotemporal distribution patterns of antibiotics and resistance genes, such as via predictive modelling of early warning systems for infectious disease, as well as identifying hotspots of AMR emergence and dissemination. As such, WBE should become one of the key tools utilised in the ‘one health’ approach. For example, through longitudinal monitoring, correlations could be determined between exposure, environmental pressures, and the occurrence of resistance genes. Such rich, data-driven research can help to provide mitigation measures spanning from ‘at source’ solutions (e.g. reduction of antibiotic usage and educational campaigns aimed at limitation of direct disposal of unused antibiotics) through technology-based ‘end-of-pipe’ treatment and environmental health-driven measures.

However, there are several research gaps that require attention before the tool can be implemented on a wider regional, national, or indeed international scale. For example, the usage, excretion, and fate of antibiotics are an important aspect of AMR dissemination. In regions where prescription data and pharmacy records are not collated or publicly available, estimations of drug usage can be back-calculated from the quantities in wastewater. Resistance-causing genetic mutations occur naturally, but also as a result of environmental factors, such as selective pressures [15]. There are many social and clinical factors that induce selective pressures for AMR emergence. Incorrect, excessive, and over-use of antimicrobials can harbour inherent resistance, including sub-therapeutic doses (e.g. livestock feed supplements); insufficient prevention and control of infection/disease; poor surveillance facilities in hospitals for diagnoses of resistant strains; ineffective regulatory barriers (e.g. self-prescribing, over-the-counter drugs); inappropriate prescribing (due to access/expense, or prescription without diagnosis); extensive agricultural use; limited availability/uncompleted antibiotic courses; incomplete removal from treated and untreated waste; and stunted development of new pharmaceuticals [15]. But equally, consistent accumulation of excreted or discarded antimicrobials into urban water will create selective pressures. These relationships are documented, particularly in association with wastewater treatment plants (WWTP) [16]. There have also been correlations drawn between the prevalence of polluting agents, such as of biocides and heavy metals, and the spread of AMR [17].

In order to fully embrace the ‘one health’ approach with WBE tools, new multiresidue methods are required that include both antibiotics and their metabolites, in various solid and liquid matrices. Several methods are already published in this field, but many of them either focus on a low number of analytes; analysis of one matrix; or do not include drug metabolites [18–26]. In this paper, a new analytical method has been developed to monitor a wide spectrum of antibiotics and their metabolites, through quantification in both solid and liquid environmental matrices. Assessments of both public and environmental health can be achieved by monitoring urban contamination and community-wide drug usage. Solid components of water systems (suspended particulate matter and river sediments) are important to include due to analyte partitioning and chemical accumulation. Additionally, by including drug metabolites in analyses, a distinction can be made between drug excretion and drug disposal [8]. This method encompasses analysis of multiple matrices, for parent drugs and several associated metabolites in order to provide a comprehensive WBE tool—supporting ‘one health’ research and tackling the AMR challenge.

## Materials and methods

### Analyte selection

There were several considerations when selecting targets: metabolism and excretion mechanisms; availability and usage; aquatic toxicity; and drug resistance. It was important to target accessible drugs, from a wide range of chemical classes, and include both broad- and narrow-spectrum antibiotics. A comprehensive list was compiled from several antibiotic groups. Overall, a total of 58 drugs, 26 metabolites, and 21 stable isotope–labelled internal standards were selected (excluding separate forms of drug complexes) (Table S1, see Supplementary Information, ESM).

Ketoconazole (an antifungal) and its metabolite were included with the antibiotics, as it has the same considerations and emergence of resistant organisms. Sulfasalazine, an anti-inflammatory, was included alongside other sulfonamides. Tuberculosis (TB) drugs were added due to the significance and high prevalence in several countries, particularly those with limited health resources. Antiretrovirals, emtricitabine, and lamivudine were included because of the significant association of tuberculosis and HIV coinfection [27]. Thalidomide, an immunomodulatory anti-leprosy drug, has been referenced as an adjunctive treatment in some tuberculosis cases [28]. Major metabolites were selected where possible. Enrofloxacin (primarily a veterinary antibiotic) was included due to its metabolism into ciprofloxacin. d-Cycloserine, a TB antibiotic and the active metabolite of prodrug terizidone, was retained in the method after terizidone was excluded (and so is unpaired in antibiotic-metabolite analyses).

### Materials

Analytical standards and deuterated (stable isotope–labelled) standards were purchased from Sigma-Aldrich (Gillingham, UK), TRC (Toronto, Canada), LGC (Middlesex, UK), or MCE (Cambridge, UK); the list of which is collated in ESM Table S1. Methanol, MeOH, was HPLC-grade (Sigma-Aldrich). Water, H_2_O, was of 18.2 MΩ quality (Elga, Marlow, UK). Glassware was deactivated using 5% dimethylchlorosilane (DMCS) in toluene (Sigma-Aldrich) to mitigate the loss of basic chemicals onto −OH sites present on glass surfaces. This consisted of rinsing once with DMCS, twice with toluene and three times with MeOH. Several mobile-phase buffers were tested during method development, including ammonium acetate (NH_4_OAc), ammonium fluoride (NH_4_F), formic acid (> 95%, HCOOH), and acetic acid (1.0 M, CH_3_COOH) purchased from Sigma-Aldrich. Oasis HLB (60 mg, 3 mL) SPE cartridges, polypropylene LC vials, and Whatman GF/F 0.7-μm filters were purchased from Waters (Manchester, UK).

### Sample collection and extraction methods

**Fig. 1:**
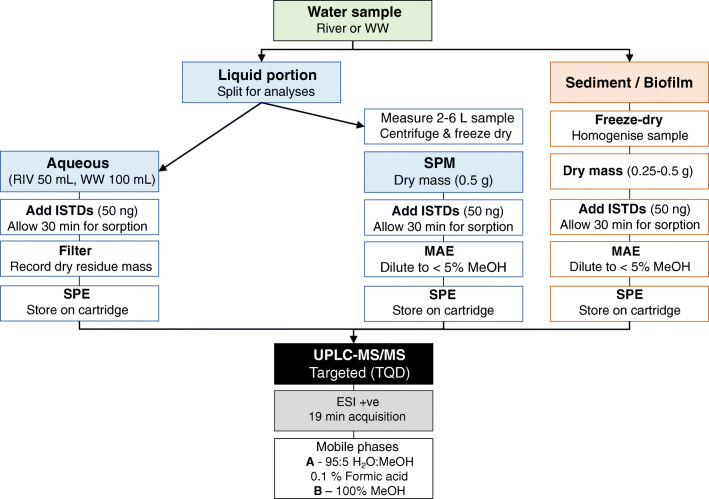
Schematic for matrix-specific sample processing. WW, wastewater; RIV, river; SPM, suspended particulate matter; ISTDs, stable isotope internal standards; MAE, microwave-assisted extraction; SPE, solid-phase extraction; TQD, triple quadrupole detector

Both solid and aqueous samples were analysed (Fig. 1). Compound partitioning between solid and liquid phases is based on their specific physical-chemical properties. Therefore, extraction from various matrices is desirable for full quantification of analytes within a whole system. However, aqueous samples were spiked with internal standards on the day of collection, at least 30 min before the filtration step. This accounted for the proportion of analyte partitioning into the solid phase and being lost during filtration. Consequently, this design can more accurately represent the total quantity of analyte present, independent of the corresponding solids analyses. This method therefore allows for the quantification of the total mass of antibiotics via the analysis of the aqueous phase and suspended particulate matter (SPM) fraction via the analysis of SPM.

Samples were collected via either 24-h time-proportional composite sampling (wastewater and associated solids) or grab sampling (river water and associated solids). Grab sampling is indicative of the system at a single time point. A composite sample is obtained by combining discrete grab samples collected at regular 15-min time intervals (using an automated sampler), which is representative of a system over time. Wastewater influent and effluent were collected from a WWTP in South West England via composite sampling. Influent SPM was extracted from a grab sample (3.3 g from 12 L, homogenised). SPM would typically be extracted from composite samples. However, due to the large quantities required for validation studies, a grab sample was used in this case. River water and sediment samples were also collected in South West England. Aqueous samples were collected via composite sampling and sediments from a singular grab sample (4.7 dry grammes, homogenised).

#### Extraction procedure for liquid matrices (analyte concentration via SPE)

Wastewater samples (50 mL) and river samples (100 mL) were spiked with 50 ng of each internal standard (50 μL of 1 μg mL^−1^ mix), shaken, and left to partition for at least 30 min at 4 °C. Samples were then filtered using an oven-dried, pre-weighed filter paper (Whatman GF/F 0.7 μm). After drying, filter papers were re-weighed, recording the mass of the suspended particulate matter removed. The filtrates were loaded under vacuum onto conditioned Oasis HLB cartridges (2 mL MeOH, followed by 2 mL H_2_O 0.1% formic acid) at approx. 5 mL min^−1^. Sampling bottles and apparatus were washed with H_2_O (< 10 mL total) during transfers. Cartridges were dried under vacuum, sealed with parafilm, and stored at − 20 °C until elution or shipment. Elution was instigated using 4 mL MeOH, where the eluate was collected in deactivated glass vials. Extracted samples were dried under nitrogen, at 40 °C and re-suspended (500 μL 80:20 H_2_O:MeOH) into LC vials (Waters).

#### Extraction procedure for solid matrices

Two methods were tested for analyte extraction from solid matrices: ultra-sonication-assisted extraction (UAE) and microwave-assisted extraction (MAE). Due to variations in solvent workup, the two methods showed large differences in the chromatography (ESM Figure S4). The MAE method was chosen for analyses due to greater chromatographic peak symmetry, lower method quantification limits, and better analyte recovery.

Suspended particulate matter (SPM) was collected by centrifugation of 1–6 L of aqueous sample (35,000 m/s^2^, 15 min) and accumulating a pellet. Other solids (river sediment and biofilm) were grab samples, independent of volume. Collected solids were dried under vacuum, freeze-dried, homogenised, split for replicate analysis (0.25–0.50 g), and spiked with 50 ng of each internal standards (50 μL of 1 μg mL^−1^ mix). Samples were stored at − 20 °C until extraction or shipment. The microwave-assisted extraction method used was modified from a method developed by Petrie et al. [29] by a substitution for HLB cartridges. The technique utilised an 800 W MARS 6 microwave (CEM, UK), which processed acidified samples over a temperature gradient to 80 °C. Samples were vortexed, filtered (0.7 μm), and diluted with H_2_O (0.1% formic) to < 5% MeOH. Solid-phase extraction was then performed using the same method as for aqueous filtrates.

### Liquid chromatography-mass spectrometry method

Liquid chromatography-mass spectrometry (LC-MS/MS) was performed using a Waters, ACQUITY UPLC™ system coupled to a Xevo TQD-ESI Mass Spectrometer and using a reverse-phase BEH C18 column (50 × 2.1 mm, 1.7 μm) with Acquity column in-line 0.2-μm pre-filter (Waters, Manchester, UK). Conditions were optimised for fast chromatographic separation and high sensitivity across a range of drug classes.

The coordinating programme used was MassLynx V4.1, and the data processing programmes were QuanLynx and TargetLynx (Waters Lab Informatics, UK). The integration parameters in QuanLynx (smoothing, apex track, and window extent) were optimised per analyte to minimise the effects of analyst subjectivity during data processing.

#### Liquid chromatography

The chromatography was optimised in terms of mobile-phase composition, needle washes, sample composition, flow rate, and inlet gradient (ESM, Table S3 and supplementary text).

The best separation and organic purge was achieved using 95:5 H_2_O:MeOH with 0.1% formic acid (mobile phase A) and 100% MeOH (mobile phase B). Strong and weak needle washes were selected to compliment the system. The strong wash used was 1:1:1:1 MeOH:ACN:IPA:H_2_O + 0.1% formic acid, and the weak wash was prepared to mimic the starting mobile phase (95:5 H_2_O:MeOH). The injection volume was 20 μL, via a partial loop injection. Sample composition performed best at 60:40 but was prepared at 80:20 H_2_O:MeOH to enable samples to be co-analysed with pre-established methods. The best results, in terms of system pressure, peak separation, and peak shape, were determined at 0.2 mL min^−1^.

**Fig. 2:**
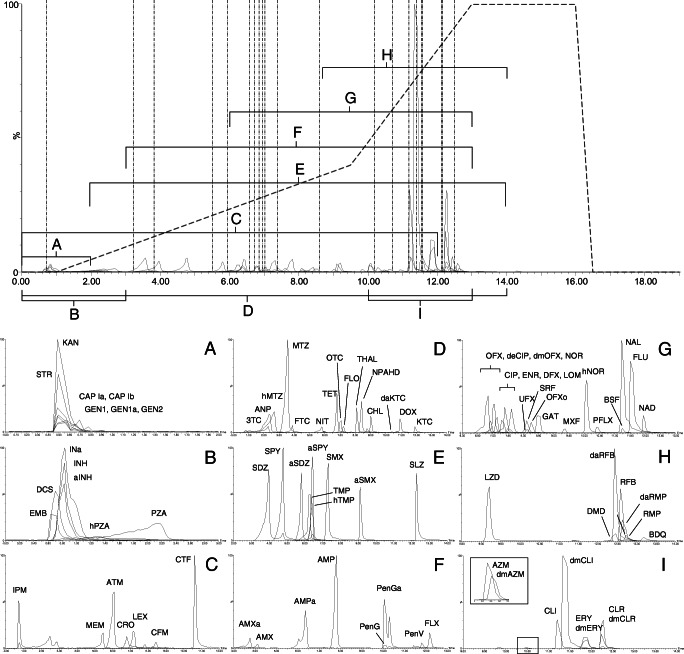
Chromatographic representation of a mobile-phase standard, scaled by relative intensity. Top: overlaid extracted ion chromatograms (EICs) of the full range (0–19 min); highlighting regions by chemical class (A–I); the distribution of internal standards (vertical dashed lines), as well as the elution gradient (dotted line), which displays mobile-phase composition (% B) against time (min). Bottom: overlaid chromatograms, sorted by chemical class. Aminoglycosides (A); TB drugs early-eluting (B); cephalosporins and carbapenems (C); ARVs, azoles, nitrofurans, cyclines, and amphenicols (D); sulfonamides (E); penicillins (F), quinolones (G); TB drugs late-eluting (H); macrolides and lincomycins (I). The EICs have not been smoothed, contrary to the method.

Numerous elution profiles were tested to maximise overall peak separation whilst retaining Gaussian shape. Starting conditions were 0% B, held for 1 min, followed by an 8.5-min gradient to 40% B; 3.5-min gradient to 100% B; 3-min hold; and finally returning to 0% B (0.5 min) to re-equilibrate for 2.5 min. The total run time was 19 min. Fig. 2 shows the distribution of peaks relative to the inlet gradient and internal standard retention times (top), as well as the example chromatographs, organised by drug class (bottom).

#### Mass spectrometry

Triple quadrupole detector (TQD) mass spectrometry offered fully targeted analyses. The source desolvation temperature was 400 °C. The cone gas flow was 100 L h^−1^ and desolvation gas flow was 1000 L h^−1^. Nitrogen was used as the nebulising and desolvation gas, and argon as the collision gas. Specific operating conditions, such as MRM masses and collision energies, were analyte-specific (ESM Table S6).

The parameters were optimised for both general and compound-specific conditions, which included ionisation mode; multiple reaction monitoring (MRM) targets and operating parameters; and acquisition peak widths and points-per-peak.

The majority of compounds favoured positive ESI mode. Sample throughput was a priority; therefore, the negative ionisation method was omitted. Consequently, some compounds experienced reductions in sensitivity, and some were excluded due to insufficient ionisation (including terizidone, cefoxitin, ceftriaxone, minocycline, triclosan, and triclocarban.)

Each analyte was tuned for the two most abundant MRM transitions (precursor to product ion *m*/*z*), and their associated cone voltage and collision energies (ESM Table S6). Capillary voltage was assigned at 3.8 kV. Due to high density of MRM channels (116 channels within 19 min), the retention time windows were staggered (± 0.1 min) where possible to reduce dramatic changes in the number of active channels/dwell time. Analyte acquisition windows were broadened to account for matrix shift. Acquisition parameters, such as peak width and points-per-peak, were tested between 20–60 s and 20–30 points, respectively. They were set at 60 s and 20 ppp to achieve the most Gaussian peak shapes.

In order to calculate any parent/metabolite impurities in the purchased standards, calibration curves were conducted separately and assessed for the presence of metabolites in parent stocks (and vice versa).

### Data processing

Mean peak smoothing parameters (iterations and width) were minimised to preserve resolution, whilst sufficiently removing noise. Data integration was automated (other than removal of false positives) using QuanLynx, to maximise the objectivity of the process. Validation parameters were determined per analyte to aid peak identification, including signal to noise (≥ 3 for detection, ≥ 10 for quantification); compound-specific sensitivity limits (IDL and IQL); and to be within stated thresholds for relative retention time and target ion ratio. All data points are accompanied by a quantitative pass/fail assessment for each parameter.

### Instrument and method performance

The following instrument performance parameters were tested: linearity and range, intra- and inter-day accuracy and precision, IDLs, and IQLs. Method performance was tested using method sensitivity (MDLs and MQLs) and method recovery. Formulae used to assess instrument and method performance are gathered in ESM Table S7.

Linearity and instrumental sensitivity were determined for each analyte by replicate injections of calibration standards over separate days; performed between 0 and 1000 ng mL^−1^ (*n* = 18 * 3) and extended through 500–3000 ng mL^−1^ (*n* = 10 * 3) for selected compounds. Inter- and intra-day precision and accuracy was determined using quality controls at three concentrations (10, 50, and 200 ng mL^−1^), in replicate (Tables 1, 2, and 3).

**Table 1:** Chromatographic and validation parameters for aqueous analyses in mobile phase, ordered by retention time

#	Chemical	Abbrev	Weighted linear calibration curves (μg L^−1^) and associated r^2^ value						Absolute and relative retention times (min) ≠				Ion Ratio (by range, μg L^−1^)	
			Range 1	r^2^	Range 2	r^2^	Range 3	r^2^	STD t_R(abs)_	STD t_R(rel)_	Corresponding ISTD	ISTD t_R(abs)_	0–100	100–1000
1	Gentamycin C1	GEN1	9.400–150	0.807	150–375	0.975	–	–	0.52 ± 0.1	6.30 ± 0.6	Metronidazole D4	3.25 ± 0.3	#N/A	#N/A
2	Gentamycin C1a	GEN1a	6.900–55	0.964	55–275	0.955	–	–	0.54 ± 0.1	6.05 ± 0.6	Metronidazole D4	3.25 ± 0.3	#N/A	#N/A
3	Gentamycin C2 C2a C2b	GEN2	17.500–140	0.920	140–350	0.986	–	–	0.54 ± 0.1	6.08 ± 0.9	Metronidazole D4	3.25 ± 0.3	#N/A	#N/A
4	Kanamycin A	KAN	11.875–95	0.927	95–950	0.992	–	–	0.54 ± 0.1	6.02 ± 0.6	Metronidazole D4	3.25 ± 0.3	#N/A	#N/A
5	Capreomycin IA	CAPIa	22.071–441	0.964	–	–	–	–	0.55 ± 0.1	5.97 ± 0.6	Metronidazole D4	3.25 ± 0.3	#N/A	#N/A
6	Capreomycin IB	CAPIb	22.071–441	0.965	–	–	–	–	0.56 ± 0.1	5.86 ± 0.6	Metronidazole D4	3.25 ± 0.3	#N/A	#N/A
7	Streptomycin A	STR	40.000–800	0.979	–	–	–	–	0.57 ± 0.1	5.69 ± 0.6	Metronidazole D4	3.25 ± 0.3	#N/A	#N/A
8	Ethambutol	EMB	0.010–100	0.999	100–200	0.996	200–1000	0.994	0.65 ± 0.1	5.02 ± 0.5	Metronidazole D4	3.25 ± 0.3	#N/A	#N/A
9	D-cycloserine	DCS	0.500–200	0.998	200–1000	0.989	–	–	0.66 ± 0.1	4.94 ± 0.5	Metronidazole D4	3.25 ± 0.3	0.3 ± 0.0	0.3 ± 0.0
10	Imipenem	IPM	5.000–500	0.993	–	–	–	–	0.72 ± 0.1	4.57 ± 0.5	Metronidazole D4	3.25 ± 0.3	1.2 ± 0.2	1.0 ± 0.1
11	Isoniazid	INH	0.500–1000	0.997	1000–3000	0.996	–	–	0.74 ± 0.1	0.98 ± 0.1	Isoniazid D4	0.72 ± 0.1	7.9 ± 0.8	8.8 ± 0.9
12	Isonicotinic acid	INa	0.500–1000	0.997	1000–3000	0.997	–	–	0.75 ± 0.1	0.96 ± 0.1	Isoniazid D4	0.72 ± 0.1	3.9 ± 0.6	3.8 ± 0.4
13	Acetyl-isoniazid	aINH	0.500–1000	0.997	–	–	–	–	0.79 ± 0.1	0.94 ± 0.1	Isoniazid D4	0.72 ± 0.1	0.5 ± 0.1	0.5 ± 0.1
14	5-Hydroxy-pyrazinoic acid	hPZA	0.100–750	0.997	–	–	–	–	0.84 ± 0.2	3.98 ± 0.8	Metronidazole D4	3.25 ± 0.3	0.1 ± 0.0	0.07 ± 0.01
15	Pyrazinamide	PZA	5.000–100	0.994	100–1000	0.994	–	–	1.47 ± 0.4	2.47 ± 1.0	Metronidazole D4	3.25 ± 0.3	1.0 ± 0.1	0.8 ± 0.1
16	2-Amino-1-(4-nitrophenyl)-1,3-propanediol	ANP	5.000–25	0.997	25–100	0.996	100–1000	0.997	2.05 ± 0.2	1.59 ± 0.2	Metronidazole D4	3.25 ± 0.3	2.4 ± 0.4	2.4 ± 0.2
17	Lamivudine	3TC	1.000–1000	0.995	–	–	–	–	2.30 ± 0.6	1.66 ± 0.3	Metronidazole D4	3.25 ± 0.3	6.3 ± 0.9	7.4 ± 0.7
18	Hydroxy-metronidazole	hMTZ	0.100–100	0.995	100–750	0.998	–	–	2.32 ± 0.2	1.40 ± 0.1	Metronidazole D4	3.25 ± 0.3	1.4 ± 0.2	1.3 ± 0.1
19	Metronidazole	MTZ	0.100–500	0.997	500–1000	0.997	–	–	3.30 ± 0.3	0.99 ± 0.1	Metronidazole D4	3.25 ± 0.3	1.8 ± 0.3	1.8 ± 0.2
20	Amoxicilloic acid	AMXa	1.000–750	0.999	–	–	–	–	3.50 ± 0.3	1.10 ± 0.1	Amoxicillin D4	3.82 ± 0.4	1.3 ± 0.3	1.3 ± 0.1
21	Emtricitabine	FTC	0.500–200	0.996	50–1000	0.998	1000–3000	0.985	3.63 ± 0.4	0.90 ± 0.1	Metronidazole D4	3.25 ± 0.3	12.4 ± 2.5	13.2 ± 1.3
22	Sulfadiazine	SDZ	0.050–1000	0.999	–	–	–	–	3.70 ± 0.4	1.74 ± 0.2	Sulfamethoxazole D4	6.97 ± 0.7	0.9 ± 0.1	0.9 ± 0.1
23	Amoxicillin	AMX	5.000–500	0.995	–	–	–	–	3.85 ± 0.4	0.99 ± 0.1	Amoxicillin D4	3.82 ± 0.4	3.3 ± 0.7	2.9 ± 0.3
24	Sulfapyridine	SPY	0.010–1000	0.999	–	–	–	–	4.50 ± 0.5	1.47 ± 0.1	Sulfamethoxazole D4	6.97 ± 0.7	1.2 ± 0.1	1.2 ± 0.1
25	Meropenem	MEM	5.000–500	0.992	–	–	–	–	5.24 ± 0.5	1.13 ± 0.1	Trimethoprim D9	5.91 ± 0.6	6.3 ± 0.9	6.2 ± 0.6
26	N-acetyl sulfadiazine	aSDZ	0.070–25	0.996	25–750	0.998	–	–	5.54 ± 0.6	1.26 ± 0.1	Sulfamethoxazole D4	6.97 ± 0.7	1.5 ± 0.3	1.5 ± 0.2
27	Nitrofurantoin	NIT	1.000–200	0.996	200–1500	0.997	–	–	5.54 ± 0.6	1.00 ± 0.1	Nitrofurantoin 13C3	5.54 ± 0.6	0.57 ± 0.09	0.53 ± 0.06
28	Aztreonam	ATM	1.000–750	0.996	–	–	–	–	5.85 ± 0.6	1.01 ± 0.1	Trimethoprim D9	5.91 ± 0.6	0.9 ± 0.2	0.79 ± 0.08
29	Ampicilloic acid	AMPa	0.500–750	0.999	–	–	–	–	6.01 ± 0.6	1.24 ± 0.1	Ampicillin D5	7.42 ± 0.7	2.0 ± 0.4	1.8 ± 0.2
30	Trimethoprim	TMP	0.500–500	0.996	–	–	–	–	6.01 ± 0.6	0.98 ± 0.1	Trimethoprim D9	5.91 ± 0.6	0.89 ± 0.09	0.9 ± 0.1
31	4-hydroxy-trimethoprim	hTMP	0.013–63	0.996	63–95	0.994	–	–	6.15 ± 0.6	0.96 ± 0.1	Trimethoprim D9	5.91 ± 0.6	3.9 ± 0.4	3.9 ± 0.4
32	N-acetyl sulfapyridine	aSPY	0.556–25	0.995	25–750	0.996	–	–	6.19 ± 0.6	1.13 ± 0.1	Sulfamethoxazole D4	6.97 ± 0.7	1.1 ± 0.2	1.1 ± 0.1
33	Tetracycline	TET	0.500–200	0.997	200–750	0.994	–	–	6.55 ± 0.7	1.64 ± 0.3	Doxcycline D3	10.73 ± 1.1	2.3 ± 0.5	2.1 ± 0.2
34	Ofloxacin (Levofloxacin)	OFX	0.100–750	0.995	–	–	–	–	6.59 ± 0.7	1.00 ± 0.1	Ofloxacin D3	6.58 ± 0.7	1.1 ± 0.2	1.0 ± 0.1
35	Desethylene ciprofloxacin	deCIP	0.500–100	0.998	100–500	0.999	–	–	6.71 ± 0.7	1.01 ± 0.1	Desmethyl-ofloxacin D8	6.76 ± 0.7	2.6 ± 0.5	2.4 ± 0.2
36	Oxytetracycline	OTC	1.000–400	0.996	–	–	–	–	6.77 ± 0.7	1.02 ± 0.1	Cefalexin D5	6.87 ± 0.7	3.9 ± 0.8	3.8 ± 0.4
37	Desmethyl-ofloxacin	dmOFX	0.500–50	0.994	50–750	0.994	–	–	6.82 ± 0.7	0.99 ± 0.1	Desmethyl-ofloxacin D8	6.76 ± 0.7	1.2 ± 0.2	1.1 ± 0.1
38	Ceftriaxone	CRO	25.000–750	0.991	–	–	–	–	6.82 ± 0.7	1.01 ± 0.1	Cefalexin D5	6.87 ± 0.7	0.8 ± 0.2	0.8 ± 0.2
39	Cefalexin	LEX	1.250–500	0.997	–	–	–	–	6.92 ± 0.7	0.99 ± 0.1	Cefalexin D5	6.87 ± 0.7	0.9 ± 0.2	0.9 ± 0.1
40	Florfenicol	FLO	10.000–200	0.988	200–750	0.996	–	–	6.92 ± 0.7	1.24 ± 0.1	Chloramphenicol D5	8.60 ± 0.9	4.9 ± 1.0	5.5 ± 0.8
41	Norfloxacin	NOR	0.010–200	0.992	200–500	0.993	500–1000	0.983	7.00 ± 0.7	0.97 ± 0.1	Desmethyl-ofloxacin D8	6.76 ± 0.7	1.4 ± 0.3	1.3 ± 0.1
42	Sulfamethoxazole	SMX	0.010–200	0.995	200–1000	0.997	–	–	7.02 ± 0.7	0.99 ± 0.1	Sulfamethoxazole D4	6.97 ± 0.7	1.3 ± 0.3	1.2 ± 0.1
43	Ciprofloxacin	CIP	0.500–400	0.996	–	–	–	–	7.14 ± 0.7	0.95 ± 0.1	Desmethyl-ofloxacin D8	6.76 ± 0.7	4.0 ± 0.6	5.1 ± 0.8
44	Enrofloxacin	ENR	0.100–100	0.992	100–750	0.992	–	–	7.20 ± 0.7	0.94 ± 0.1	Desmethyl-ofloxacin D8	6.76 ± 0.7	2.4 ± 0.5	2.5 ± 0.2
45	Danofloxacin	DFX	5.000–750	0.997	–	–	–	–	7.34 ± 0.7	0.92 ± 0.1	Desmethyl-ofloxacin D8	6.76 ± 0.7	3.1 ± 1.6	11.4 ± 3.4
46	Lomefloxacin	LOM	0.100–500	0.997	–	–	–	–	7.44 ± 0.7	0.91 ± 0.1	Desmethyl-ofloxacin D8	6.76 ± 0.7	2.2 ± 0.4	2.2 ± 0.2
47	Ampicillin	AMP	5.000–200	0.997	–	–	–	–	7.49 ± 0.7	0.99 ± 0.1	Ampicillin D5	7.42 ± 0.7	5.1 ± 0.8	5.2 ± 0.5
48	Thalidomide	THAL	1.000–100	0.998	100–750	0.998	–	–	7.87 ± 0.8	0.75 ± 0.1	Trimethoprim D9	5.91 ± 0.6	2.4 ± 0.2	2.3 ± 0.2
49	Ulifloxacin	UFX	5.000–500	0.986	–	–	–	–	7.90 ± 0.8	0.86 ± 0.1	Desmethyl-ofloxacin D8	6.76 ± 0.7	1.0 ± 0.2	1.1 ± 0.1
50	Sarafloxacin	SRF	0.500–500	0.992	–	–	–	–	7.92 ± 0.8	0.85 ± 0.1	Desmethyl-ofloxacin D8	6.76 ± 0.7	1.7 ± 0.2	2.5 ± 0.4
51	Ofloxacin N-oxide	OFXo	12.000–75	0.995	75–1000	0.995	–	–	8.07 ± 0.8	0.84 ± 0.1	Desmethyl-ofloxacin D8	6.76 ± 0.7	0.9 ± 0.2	0.9 ± 0.1
52	1-(2-nitrobenzylidenamino)-2,4-imidazolidinedione	NPAHD	0.100–200	0.994	200–1000	0.997	–	–	8.11 ± 0.8	1.06 ± 0.1	Chloramphenicol D5	8.60 ± 0.9	1.9 ± 0.4	1.8 ± 0.2
53	Cefixime	CFM	5.000–750	0.996	–	–	–	–	8.20 ± 0.8	0.72 ± 0.1	Trimethoprim D9	5.91 ± 0.6	2.7 ± 0.4	2.2 ± 0.3
54	Gatifloxacin	GAT	0.010–500	0.996	–	–	–	–	8.32 ± 0.8	0.81 ± 0.1	Desmethyl-ofloxacin D8	6.76 ± 0.7	1.5 ± 0.3	1.6 ± 0.2
55	Chloramphenicol	CHL	0.500–200	0.997	200–1000	0.998	–	–	8.69 ± 0.9	0.99 ± 0.1	Chloramphenicol D5	8.60 ± 0.9	0.5 ± 0.1	0.6 ± 0.1
56	N-acetyl sulfamethoxazole	aSMX	0.063–475	0.997	475–1500	0.997	–	–	8.90 ± 0.9	0.78 ± 0.1	Sulfamethoxazole D4	6.97 ± 0.7	0.8 ± 0.2	0.8 ± 0.1
57	Linezolid	LZD	0.100–1000	0.994	–	–	–	–	8.93 ± 0.9	0.96 ± 0.1	Chloramphenicol D5	8.60 ± 0.9	1.4 ± 0.3	1.4 ± 0.1
58	Moxifloxacin	MXF	1.250–500	0.997	–	–	–	–	9.14 ± 0.9	0.74 ± 0.1	Desmethyl-ofloxacin D8	6.76 ± 0.7	2.1 ± 0.4	1.8 ± 0.3
59	Hydroxy-norfloxacin	hNOR	1.000–100	0.987	100–1000	0.995	–	–	9.82 ± 1.0	0.69 ± 0.1	Desmethyl-ofloxacin D8	6.76 ± 0.7	1.1 ± 0.2	1.1 ± 0.1
60	Penicilloic G acid	PenGa	0.500–750	0.997	–	–	–	–	9.94 ± 1.0	1.14 ± 0.1	Penicillin G D7	11.29 ± 1.1	1.0 ± 0.2	1.0 ± 0.1
61	Prulifloxacin	PFLX	1.000–400	0.997	–	–	–	–	10.18 ± 1.0	0.66 ± 0.1	Desmethyl-ofloxacin D8	6.76 ± 0.7	1.2 ± 0.2	1.1 ± 0.1
62	Azithromycin	AZM	0.050–1000	0.998	–	–	–	–	10.22 ± 1.0	1.15 ± 0.1	Erythromycin 13C3,D3	11.74 ± 1.2	1.4 ± 0.3	1.1 ± 0.1
63	N-desmethyl azithromycin	dmAZM	1.250–1000	0.996	–	–	–	–	10.29 ± 1.0	1.14 ± 0.1	Erythromycin 13C3,D3	11.74 ± 1.2	0.9 ± 0.2	0.9 ± 0.1
64	Ceftiofur	CTF	0.500–750	0.997	–	–	–	–	10.45 ± 1.0	0.93 ± 0.1	Flumequine 13C3	11.42 ± 1.1	1.9 ± 0.4	1.8 ± 0.2
65	Deacetyl-ketoconazole	daKTC	1.250–100	0.996	100–500	0.976	–	–	10.49 ± 1.0	1.12 ± 0.1	Ketoconazole D3	11.80 ± 1.2	1.7 ± 0.5	1.7 ± 0.2
66	Doxycycline	DOX	1.250–750	0.998	–	–	–	–	10.77 ± 1.1	1.00 ± 0.1	Doxcycline D3	10.73 ± 1.1	0.8 ± 0.2	0.8 ± 0.1
67	Clindamycin	CLI	0.500–200	0.998	200–3000	0.997	–	–	11.09 ± 1.1	1.06 ± 0.1	Erythromycin 13C3,D3	11.74 ± 1.2	63.8 ± 12.8	74.5 ± 7.4
68	Nalidixic acid	NAL	0.010–500	0.998	–	–	–	–	11.14 ± 1.1	1.03 ± 0.1	Flumequine 13C3	11.42 ± 1.1	1.7 ± 0.2	1.7 ± 0.2
69	Besifloxacin	BSF	1.250–750	0.988	–	–	–	–	11.18 ± 1.1	1.02 ± 0.1	Flumequine 13C3	11.42 ± 1.1	2.5 ± 0.4	2.0 ± 0.3
70	N-desmethyl clindamycin	dmCLI	0.005–200	0.992	–	–	–	–	11.26 ± 1.1	1.04 ± 0.1	Erythromycin 13C3,D3	11.74 ± 1.2	12.7 ± 1.9	11.6 ± 2.3
71	Penicillin G	PenG	0.500–400	0.994	–	–	–	–	11.32 ± 1.1	1.00 ± 0.1	Penicillin G D7	11.29 ± 1.1	1.3 ± 0.5	0.9 ± 0.5
72	Flumequine	FLU	0.010–200	0.996	200–1000	0.997	–	–	11.45 ± 1.1	1.00 ± 0.1	Flumequine 13C3	11.42 ± 1.1	2.9 ± 0.4	2.8 ± 0.3
73	Penicillin V	PenV	10.000–200	0.995	200–1000	0.993	–	–	11.72 ± 1.2	0.96 ± 0.1	Penicillin G D7	11.29 ± 1.1	4.4 ± 0.9	4.4 ± 0.7
74	Erythromycin	ERY	0.005–200	0.998	200–1000	0.999	1000–3000	0.996	11.74 ± 1.2	1.00 ± 0.1	Erythromycin 13C3,D3	11.74 ± 1.2	3.7 ± 0.6	3.8 ± 0.4
75	N-demethyl erythromycin	dmERY	0.007–1100	0.996	1100–3000	0.988	–	–	11.74 ± 1.2	1.00 ± 0.1	Erythromycin 13C3,D3	11.74 ± 1.2	3.6 ± 1.3	15.7 ± 3.9
76	Ketoconazole	KTC	0.500–200	0.997	–	–	–	–	11.81 ± 1.2	1.00 ± 0.1	Ketoconazole D3	11.80 ± 1.2	5.8 ± 1.2	5.8 ± 1.2
77	Nadifloxacin	NAD	1.000–400	0.969	–	–	–	–	11.86 ± 1.2	0.96 ± 0.1	Flumequine 13C3	11.42 ± 1.1	1.0 ± 0.1	1.0 ± 0.1
78	Flucloxacillin	FLX	0.500–1000	0.992	–	–	–	–	12.09 ± 1.2	0.95 ± 0.1	Flumequine 13C3	11.42 ± 1.1	3.5 ± 0.9	3.7 ± 0.6
79	N-desmethyl clarithromycin	dmCLR	0.017–1250	0.998	1250–2000	0.981	–	–	12.15 ± 1.2	1.00 ± 0.1	Clarithromycin D3	12.15 ± 1.2	4.4 ± 0.9	4.1 ± 0.4
80	Clarithromycin	CLR	0.005–200	0.997	200–3000	0.998	–	–	12.15 ± 1.2	1.00 ± 0.1	Clarithromycin D3	12.15 ± 1.2	8.2 ± 1.6	8.1 ± 0.8
81	Sulfasalazine	SLZ	1.250–1000	0.999	–	–	–	–	12.19 ± 1.2	1.00 ± 0.1	Sulfasalazine D4	12.14 ± 1.2	1.3 ± 0.3	1.2 ± 0.1
82	Delamanid	DMD	0.500–400	0.996	–	–	–	–	12.31 ± 1.2	1.01 ± 0.1	Rifabutin D7	12.48 ± 1.2	39.4 ± 11.8	38.2 ± 5.7
83	25-O-desacetyl rifabutin	daRFB	0.100–100	0.996	100–750	0.980	–	–	12.34 ± 1.2	1.01 ± 0.1	Rifabutin D7	12.48 ± 1.2	2.2 ± 0.6	2.2 ± 0.3
84	25-desacetyl rifampicin	daRMP	5.000–500	0.937	–	–	–	–	12.43 ± 1.2	1.00 ± 0.1	Rifabutin D7	12.48 ± 1.2	2.0 ± 0.4	1.8 ± 0.2
85	Rifabutin	RFB	0.500–400	0.996	–	–	–	–	12.49 ± 1.2	1.00 ± 0.1	Rifabutin D7	12.48 ± 1.2	1.4 ± 0.3	1.5 ± 0.2
86	Rifampicin	RMP	1.250–1000	0.989	–	–	–	–	12.57 ± 1.3	0.99 ± 0.1	Rifabutin D7	12.48 ± 1.2	1.7 ± 0.5	1.7 ± 0.3
87	Bedaquiline	BDQ	5.000–400	0.778	–	–	–	–	13.07 ± 1.3	0.95 ± 0.1	Rifabutin D7	12.48 ± 1.2	#N/A	#N/A

**Table 2:** Chromatographic and validation parameters for solid analyses in mobile phase, ordered by retention time (only displaying those that differ from aqueous-phase analysis)

**Number**	**Chemical**	**Abbrev**	Weighted linear calibration curves (μg L^−1^) and associated *r*^2^ value						Absolute and relative retention times (min) ≠				Ion ratio (by range, μg L^−1^)	
			**Range 1**	***r***^**2**^	**Range 2**	***r***^**2**^	**Range 3**	***r***^**2**^	**STD** ***t***_**R(abs)**_	**STD** ***t***_**R(rel)**_	**Corresponding ISTD**	**ISTD** ***t***_**R(abs)**_	**0–100**	**100–1000**
9	d-Cycloserine	DCS	1.000–750	0.997	-	-	-	-	0.66 ± 0.1	4.94 ± 0.5	Metronidazole D4	3.25 ± 0.3	0.3 ± 0.05	0.3 ± 0.04
11	Isoniazid	INH	0.500–200	0.997	200–1800	0.995	-	-	0.74 ± 0.1	4.33 ± 0.4	Metronidazole D4	3.25 ± 0.3	7.9 ± 0.8	8.8 ± 0.9
12	Isonicotinic acid	INa	0.500–200	0.999	200–3000	0.995	-	-	0.75 ± 0.1	4.48 ± 0.4	Metronidazole D4	3.25 ± 0.3	3.9 ± 0.6	3.8 ± 0.4
13	Acetyl-isoniazid	aINH	0.500–100	0.992	100-1000	0.996	-	-	0.79 ± 0.1	4.12 ± 0.4	Metronidazole D4	3.25 ± 0.3	0.5 ± 0.1	0.5 ± 0.1
14	5-Hydroxy-pyrazinoic acid	hPZA	1.000–750	0.998	-	-	-	-	0.84 ± 0.2	3.98 ± 0.8	Metronidazole D4	3.25 ± 0.3	4.5 ± 1.8	14.2 ± 1.4
15	Pyrazinamide	PZA	1.250–1000	0.998	-	-	-	-	1.47 ± 0.4	2.47 ± 1.0	Metronidazole D4	3.25 ± 0.3	1.0 ± 0.1	1.3 ± 0.1
20	Amoxicilloic acid	AMXa	1.000–1000	0.998	-	-	-	-	3.50 ± 0.3	2.00 ± 0.2	Sulfamethoxazole D4	6.97 ± 0.7	1.3 ± 0.3	1.3 ± 0.1
23	Amoxicillin	AMX	5.000–100	0.995	100–1000	0.997	-	-	3.85 ± 0.4	1.81 ± 0.2	Sulfamethoxazole D4	6.97 ± 0.7	3.3 ± 0.7	2.9 ± 0.3
27	Nitrofurantoin	NIT	5.000–1000	0.998	-	-	-	-	5.54 ± 0.6	1.00 ± 0.1	Nitrofurantoin 13C3	5.54 ± 0.6	1.8 ± 0.3	1.9 ± 0.2
29	Ampicilloic acid	AMPa	0.500–750	0.997	-	-	-	-	6.01 ± 0.6	1.16 ± 0.1	Sulfamethoxazole D4	6.97 ± 0.7	2.0 ± 0.4	1.8 ± 0.2
32	N-Acetyl sulfapyridine	aSPY	0.500–25	0.996	25–750	0.997	-	-	6.19 ± 0.6	1.13 ± 0.1	Sulfamethoxazole D4	6.97 ± 0.7	0.9 ± 0.1	0.9 ± 0.1
33	Tetracycline	TET	0.500–200	0.996	200–500	0.995	-	-	6.55 ± 0.7	1.03 ± 0.1	Desmethyl-ofloxacin D8	6.76 ± 0.7	2.3 ± 0.5	2.1 ± 0.2
36	Oxytetracycline	OTC	1.000–200	0.998	200–500	0.994	-	-	6.77 ± 0.7	1.00 ± 0.1	Desmethyl-ofloxacin D8	6.76 ± 0.7	3.9 ± 0.8	3.8 ± 0.4
38	Ceftriaxone	CRO	25.000–1000	0.994	-	-	-	-	6.82 ± 0.7	0.86 ± 0.1	Trimethoprim D9	5.91 ± 0.6	0.8 ± 0.2	0.8 ± 0.2
39	Cefalexin	LEX	1.250–100	0.994	100–750	0.997	-	-	6.92 ± 0.7	0.85 ± 0.1	Trimethoprim D9	5.91 ± 0.6	0.9 ± 0.2	0.9 ± 0.1
47	Ampicillin	AMP	5.000–1000	0.998	-	-	-	-	7.49 ± 0.7	0.93 ± 0.1	Sulfamethoxazole D4	6.97 ± 0.7	0.2 ± 0.03	0.2 ± 0.02
48	Thalidomide	THAL	1.000–100	0.998	100–750	0.998	-	-	7.87 ± 0.8	0.75 ± 0.1	Trimethoprim D9	5.91 ± 0.6	2.4 ± 0.2	2.3 ± 0.2
50	Sarafloxacin	SRF	0.500–1000	0.998	-	-	-	-	7.92 ± 0.8	0.85 ± 0.1	Desmethyl-ofloxacin D8	6.76 ± 0.7	0.6 ± 0.1	0.4 ± 0.1
59	Hydroxy-norfloxacin	hNOR	1.000–750	0.987	-	-	-	-	9.82 ± 1.0	0.69 ± 0.1	Desmethyl-ofloxacin D8	6.76 ± 0.7	0.9 ± 0.2	0.9 ± 0.1
60	Penicilloic G acid	PenGa	0.500–750	0.997	-	-	-	-	9.94 ± 1.0	0.70 ± 0.1	Sulfamethoxazole D4	6.97 ± 0.7	1.0 ± 0.2	1.0 ± 0.1
62	Azithromycin	AZM	0.050–1000	0.999	-	-	-	-	10.22 ± 1.0	1.19 ± 0.1	Clarithromycin D3	12.15 ± 1.2	1.4 ± 0.3	1.1 ± 0.1
63	N-Desmethyl azithromycin	dmAZM	1.250–400	0.991	-	-	-	-	10.29 ± 1.0	1.18 ± 0.1	Clarithromycin D3	12.15 ± 1.2	1.1 ± 0.3	1.1 ± 0.1
64	Ceftiofur	CTF	0.500–200	0.997	-	-	-	-	10.45 ± 1.0	1.16 ± 0.1	Clarithromycin D3	12.15 ± 1.2	1.9 ± 0.4	1.8 ± 0.2
65	Deacetyl-ketoconazole	daKTC	1.250–200	0.993	200–500	0.995	-	-	10.49 ± 1.0	1.09 ± 0.1	Flumequine 13C3	11.42 ± 1.1	1.7 ± 0.5	1.7 ± 0.2
66	Doxycycline	DOX	1.250–1000	0.991	-	-	-	-	10.77 ± 1.1	1.15 ± 0.1	Rifabutin D7	12.48 ± 1.2	0.8 ± 0.2	0.8 ± 0.1
67	Clindamycin	CLI	0.500–1000	0.993	-	-	-	-	11.09 ± 1.1	1.03 ± 0.1	Flumequine 13C3	11.42 ± 1.1	63.8 ± 12.8	74.5 ± 7.4
70	N-Desmethyl clindamycin	dmCLI	0.005–200	0.994	-	-	-	-	11.26 ± 1.1	1.01 ± 0.1	Flumequine 13C3	11.42 ± 1.1	12.7 ± 1.9	11.6 ± 2.3
71	Penicillin G	PenG	0.500–500	0.994	-	-	-	-	11.32 ± 1.1	0.61 ± 0.1	Sulfamethoxazole D4	6.97 ± 0.7	1.3 ± 0.5	0.9 ± 0.5
73	Penicillin V	PenV	10.00–1000	0.992	-	-	-	-	11.72 ± 1.2	0.59 ± 0.1	Sulfamethoxazole D4	6.97 ± 0.7	4.4 ± 0.9	4.4 ± 0.7
74	Erythromycin	ERY	0.005–200	0.997	-	-	-	-	11.74 ± 1.2	1.03 ± 0.1	Clarithromycin D3	12.15 ± 1.2	3.7 ± 0.6	3.8 ± 0.4
75	N-Demethyl erythromycin	dmERY	0.034–136	0.999	-	-	-	-	11.74 ± 1.2	1.03 ± 0.1	Clarithromycin D3	12.15 ± 1.2	3.6 ± 1.3	15.7 ± 3.9
81	Sulfasalazine	SLZ	5.000–1000	0.997	-	-	-	-	12.19 ± 1.2	0.57 ± 0.1	Sulfamethoxazole D4	6.97 ± 0.7	1.4 ± 0.3	1.2 ± 0.1

**Table 3:** Chromatographic instrument performance, ordered by retention time

#	Chemical	Abbrev	Instrumental limits (μg L^−1^)		Intra-day performance (%)			Inter-day performance (%)		
			IDL	IQL	Accuracy	Precision	n	Accuracy	Precision	n
*Calibrations for aqueous analyses*										
1	Gentamycin C1	GEN1	2.820	9.400	103.0	60.9	5	97.6	8.9	8
2	Gentamycin C1a	GEN1a	2.070	6.900	86.9	55.9	3	82.6	39.7	6
3	Gentamycin C2 C2a C2b	GEN2	5.250	17.500	76.3	57.8	3	96.4	23.0	9
4	Kanamycin A	KAN	3.563	11.875	72.3	31.4	4	67.1	33.7	12
5	Capreomycin IA	CAPIa	6.621	22.071	85.3	51.2	2	71.7	16.0	12
6	Capreomycin IB	CAPIb	6.621	22.071	85.5	57.5	2	76.2	20.4	12
7	Streptomycin A	STR	12.000	40.000	109.9	17.0	6	109.2	20.0	15
8	Ethambutol	EMB	0.003	0.010	79.0	20.0	9	79.1	22.2	27
9	D-cycloserine	DCS	0.150	0.500	101.1	8.7	9	102.1	9.9	27
10	Imipenem	IPM	1.500	5.000	139.1	21.7	8	140.6	24.8	26
11	Isoniazid	INH	0.150	0.500	103.8	7.8	9	106.7	12.0	27
12	Isonicotinic acid	INa	0.150	0.500	97.6	9.8	9	97.7	10.3	27
13	Acetyl-isoniazid	aINH	0.150	0.500	101.8	9.5	9	101.5	9.7	27
14	5-Hydroxy-pyrazinoic acid	hPZA	0.030	0.100	97.7	21.4	7	103.7	21.4	18
15	Pyrazinamide	PZA	1.500	5.000	106.9	17.5	9	106.6	16.2	25
16	2-Amino-1-(4-nitrophenyl)-1,3-propanediol	ANP	1.500	5.000	105.1	10.6	7	110.2	14.5	21
17	Lamivudine	3TC	0.300	1.000	95.5	11.6	9	98.0	11.9	27
18	Hydroxy-metronidazole	hMTZ	0.030	0.100	99.3	9.8	9	94.0	9.6	26
19	Metronidazole	MTZ	0.030	0.100	102.9	7.9	9	104.4	9.4	26
20	Amoxicilloic acid	AMXa	0.300	1.000	103.6	6.5	9	103.6	10.5	27
21	Emtricitabine	FTC	0.150	0.500	91.3	18.7	7	86.2	19.7	22
22	Sulfadiazine	SDZ	0.015	0.050	101.9	11.7	8	99.4	12.9	26
23	Amoxicillin	AMX	1.500	5.000	105.0	7.6	8	105.7	10.0	25
24	Sulfapyridine	SPY	0.003	0.010	100.8	5.9	9	100.6	7.6	27
25	Meropenem	MEM	1.500	5.000	84.0	13.8	9	82.3	12.7	22
26	N-acetyl sulfadiazine	aSDZ	0.021	0.070	95.1	3.1	5	99.1	5.3	18
27	Nitrofurantoin	NIT	0.300	1.000	88.7	11.7	9	89.0	11.5	25
28	Aztreonam	ATM	0.300	1.000	84.8	13.4	9	87.0	17.8	25
29	Ampicilloic acid	AMPa	0.150	0.500	97.0	14.4	9	97.5	15.4	25
30	Trimethoprim	TMP	0.150	0.500	106.7	11.2	8	106.7	13.8	22
31	4-hydroxy-trimethoprim	hTMP	0.004	0.013	99.9	12.0	8	100.1	12.5	24
32	N-acetyl sulfapyridine	aSPY	0.167	0.556	105.3	5.9	9	105.0	6.5	25
33	Tetracycline	TET	0.150	0.500	92.7	6.9	5	103.1	20.4	16
34	Ofloxacin (Levofloxacin)	OFX	0.030	0.100	97.0	8.3	8	100.7	11.0	23
35	Desethylene ciprofloxacin	deCIP	0.150	0.500	91.6	11.9	7	89.3	13.5	21
36	Oxytetracycline	OTC	0.300	1.000	102.6	15.4	9	97.3	15.3	22
37	Desmethyl-ofloxacin	dmOFX	0.150	0.500	95.7	6.5	7	96.9	11.0	20
38	Ceftriaxone	CRO	7.500	25.000	104.2	17.4	6	91.5	20.3	16
39	Cefalexin	LEX	0.375	1.250	90.0	10.0	9	90.1	10.4	24
40	Florfenicol	FLO	3.000	10.000	111.3	17.9	9	114.3	18.2	26
41	Norfloxacin	NOR	0.003	0.010	103.0	15.0	8	96.5	19.9	25
42	Sulfamethoxazole	SMX	0.002	0.005	100.0	7.0	9	101.4	9.4	27
43	Ciprofloxacin	CIP	0.150	0.500	101.1	10.0	8	96.6	20.1	26
44	Enrofloxacin	ENR	0.030	0.100	110.1	13.1	8	109.7	14.7	24
45	Danofloxacin	DFX	1.500	5.000	112.0	47.9	8	93.3	39.5	10
46	Lomefloxacin	LOM	0.030	0.100	112.6	21.4	9	118.1	20.8	27
47	Ampicillin	AMP	1.500	5.000	101.4	15.8	9	105.1	17.9	25
48	Thalidomide	THAL	0.300	1.000	102.4	16.1	9	102.8	17.2	24
49	Ulifloxacin	UFX	1.500	5.000	115.9	30.7	5	121.9	37.9	15
50	Sarafloxacin	SRF	0.150	0.500	92.6	10.8	9	92.8	13.8	25
51	Ofloxacin N-oxide	OFXo	3.600	12.000	96.7	8.9	6	97.6	13.6	18
52	1-(2-nitrobenzylidenamino)-2,4-imidazolidinedione	NPAHD	0.030	0.100	101.9	9.5	9	102.8	13.5	25
53	Cefixime	CFM	1.500	5.000	92.1	22.5	9	83.0	20.2	27
54	Gatifloxacin	GAT	0.003	0.010	99.6	7.1	7	102.9	13.5	24
55	Chloramphenicol	CHL	0.150	0.500	110.3	19.1	8	115.1	22.7	19
56	N-acetyl sulfamethoxazole	aSMX	0.019	0.063	101.1	8.2	9	101.3	9.5	27
57	Linezolid	LZD	0.030	0.100	98.2	11.5	5	96.2	19.0	17
58	Moxifloxacin	MXF	0.375	1.250	92.0	15.9	8	90.3	17.1	22
59	Hydroxy-norfloxacin	hNOR	3.600	12.000	99.6	16.2	8	98.8	17.3	22
60	Penicilloic G acid	PenGa	0.150	0.500	100.8	10.2	7	100.9	13.3	23
61	Prulifloxacin	PFLX	0.300	1.000	89.7	24.0	8	84.9	22.6	25
62	Azithromycin	AZM	0.015	0.050	91.2	17.7	7	89.5	24.7	20
63	N-desmethyl azithromycin	dmAZM	0.375	1.250	93.3	15.1	6	91.1	15.0	18
64	Ceftiofur	CTF	0.150	0.500	103.1	17.0	7	110.1	15.8	20
65	Deacetyl-ketoconazole	daKTC	0.375	1.250	102.8	5.3	3	94.4	12.1	6
66	Doxycycline	DOX	0.375	1.250	96.6	11.9	8	96.6	14.2	23
67	Clindamycin	CLI	0.150	0.500	93.9	16.4	9	94.2	17.7	25
68	Nalidixic acid	NAL	0.003	0.010	105.0	16.1	9	101.9	17.3	25
69	Besifloxacin	BSF	0.375	1.250	85.2	13.7	8	81.3	19.2	24
70	N-desmethyl clindamycin	dmCLI	0.002	0.005	93.5	19.7	9	86.9	17.3	26
71	Penicillin G	PenG	0.150	0.500	123.8	21.9	5	124.3	27.8	17
72	Flumequine	FLU	0.003	0.010	102.9	12.4	9	102.9	12.4	25
73	Penicillin V	PenV	3.000	10.000	93.3	25.7	8	90.1	27.1	21
74	Erythromycin	ERY	0.002	0.005	103.3	8.3	9	101.5	11.0	26
75	N-demethyl erythromycin	dmERY	0.002	0.007	104.0	8.8	9	103.2	10.5	26
76	Ketoconazole	KTC	0.003	0.010	101.9	20.7	6	99.6	19.3	17
77	Nadifloxacin	NAD	0.300	1.000	90.2	19.3	9	98.4	24.3	24
78	Flucloxacillin	FLX	0.150	0.500	99.2	15.5	8	108.8	19.3	26
79	N-desmethyl clarithromycin	dmCLR	0.005	0.017	97.6	6.2	9	98.7	9.0	25
80	Clarithromycin	CLR	0.002	0.005	104.8	9.6	9	106.2	10.5	27
81	Sulfasalazine	SLZ	0.375	1.250	109.6	9.7	8	108.6	14.1	24
82	Delamanid	DMD	0.150	0.500	95.3	27.9	8	97.5	31.3	22
83	25-O-desacetyl rifabutin	daRFB	0.030	0.100	104.5	27.5	9	102.4	21.2	22
84	25-desacetyl rifampicin	daRMP	1.500	5.000	117.3	33.7	6	103.9	38.1	17
85	Rifabutin	RFB	0.150	0.500	100.4	14.1	8	105.4	14.0	25
86	Rifampicin	RMP	0.375	1.250	114.4	34.0	7	97.1	42.4	19
87	Bedaquiline	BDQ	1.500	5.000	excluded			125.7	45.1	6
*Alternative calibrations for solids analyses*										
9	D-cycloserine	DCS	0.300	1.000	96.7	12.7	9	101.2	15.5	27
11	Isoniazid	INH	0.150	0.500	87.3	24.7	9	83.3	24.2	27
12	Isonicotinic acid	INa	0.150	0.500	98.8	5.1	6	100.2	8.7	18
13	Acetyl-isoniazid	aINH	0.150	0.500	99.9	4.9	6	100.3	5.0	18
14	5-Hydroxy-pyrazinoic acid	hPZA	0.300	1.000	102.9	9.0	8	102.8	11.8	23
15	Pyrazinamide	PZA	0.375	1.250	97.6	11.1	9	100.0	10.2	24
20	Amoxicilloic acid	AMXa	0.300	1.000	97.9	7.1	9	102.5	12.3	27
23	Amoxicillin	AMX	1.500	5.000	102.3	15.9	9	109.7	14.5	27
27	Nitrofurantoin	NIT	1.500	5.000	102.4	9.2	8	99.7	11.9	26
29	Ampicilloic acid	AMPa	0.150	0.500	97.0	8.4	9	101.6	10.7	27
32	N-acetyl sulfapyridine	aSPY	0.150	0.500	103.3	5.4	8	100.9	9.2	26
33	Tetracycline	TET	0.150	0.500	106.5	10.2	7	112.7	12.3	20
36	Oxytetracycline	OTC	0.300	1.000	102.0	11.0	7	105.0	28.3	25
38	Ceftriaxone	CRO	7.500	25.000	91.2	16.9	6	84.8	17.8	18
39	Cefalexin	LEX	0.375	1.250	89.8	19.6	9	77.6	18.1	26
47	Ampicillin	AMP	1.500	5.000	102.2	7.1	9	103.6	9.5	27
48	Thalidomide	THAL	0.300	1.000	87.9	13.3	9	86.3	18.9	26
50	Sarafloxacin	SRF	0.150	0.500	101.6	12.5	8	113.6	17.8	25
59	Hydroxy-norfloxacin	hNOR	0.300	1.000	97.3	15.7	7	104.5	17.9	19
60	Penicilloic G acid	PenGa	0.150	0.500	107.3	7.7	9	111.4	11.7	27
62	Azithromycin	AZM	0.015	0.050	89.6	18.7	7	82.5	24.6	21
63	N-desmethyl azithromycin	dmAZM	0.375	1.250	85.2	35.8	5	92.1	28.5	12
64	Ceftiofur	CTF	0.150	0.500	102.6	27.9	8	101.8	28.4	24
65	Deacetyl-ketoconazole	daKTC	0.375	1.250	70.7	23.4	4	76.6	33.7	13
66	Doxycycline	DOX	0.375	1.250	92.0	33.7	9	82.0	26.6	24
67	Clindamycin	CLI	0.150	0.500	155.3	28.9	9	153.3	33.8	27
70	N-desmethyl clindamycin	dmCLI	0.002	0.005	98.8	25.4	9	82.7	25.0	27
71	Penicillin G	PenG	0.150	0.500	86.4	24.6	8	91.9	21.7	16
73	Penicillin V	PenV	3.000	10.000	101.9	22.7	8	94.2	25.8	26
75	N-demethyl erythromycin	dmERY	0.002	0.007	104.9	34.7	9	96.8	33.4	27
74	Erythromycin	ERY	0.002	0.005	98.6	33.4	9	94.9	31.6	27
81	Sulfasalazine	SLZ	1.500	5.000	88.4	8.1	7	110.4	16.8	23

Grey text indicates poor performance (accuracy beyond 100 ± 15%, or precision of >25% error)

Accuracy was determined from physical duplicates of quality controls, injected in replicate, calculated by the measured-concentration over the theoretical-concentration, as a percentage. Precision was defined as the standard deviation from these same samples. Both inter- and intra-day tests were performed (Table 3).

Matrix recoveries were quantified by spiking aqueous and solid matrices with four quantities of standard (0, 5, 25, 100 ng) at the beginning of extraction processes. For aqueous matrix, samples were spiked before filtration; for solids, samples were spiked before microwave extraction. Method performance was determined by subtracting the average unspiked matrix blanks from the analyte mass and calculating the percentage mass that was recovered throughout sample preparation. Method detection limits were then calculated per matrix using instrumental limits, matrix-specific recovery, and the sample SPE concentration factor (ESM Table S7). Concentration factors per matrix were calculated as the sample volume, or mass, over elution volume, i.e. Milli-Q and river water, 200; wastewater, 100; influent SPM, 0.52; river sediment, 0.54.

## Results and discussion

### Instrument and method performance

#### Instrument performance

Two techniques were used to improve accuracy, particularly at the lower end of instrumental detection: calibration curves were weighted at 1/*x*, and several curves were divided into separate concentration ranges. The weighting scheme was chosen by observing the accuracy of different weighting factors (no weighting, 1/*x*, and 1/*x*^2^) across the range—an example of which is displayed in ESM Figure S1. The unweighted curves typically produced the highest *r*^2^ values but had large percentage errors at low concentrations. By applying a weighting factor of 1/*x*^2^, percentage error was more consistent across the entire range, but the error was often above the accepted ≤ ± 15% error [30] and would give poor *r*^2^ values (< 0.95). Consequently, a factor of 1/*x* produced the best accuracy at the lowest variance.

The division of calibration curves was also performed to improve overall accuracy. Calculations of linear regression are dictated by absolute variance; therefore, the slope is generally dominated by data from the highest concentrations. As the instrumental response approaches saturation, the curve can become biased, decreasing the gradient of the line. Across a large concentration range, this effect has negligible impact on the *r*^2^, but may shift the intercept, and therefore reduce the accuracy around the IQL. An example of this process is shown across three ranges, in ESM Figure S2. The close-up of the origin shows significantly greater accuracy in the lowest calibration range compared to the higher.

Table 3 displays the results for sensitivity (instrument detection limits, IDL, and quantification limits, IQL) as well as performance (accuracy and precision). IQLs were determined from the lowest calibration standard which consistently gave signal-to-noise ratios of ≥ 10, and IDLs were then calculated for an extrapolated signal-to-noise equal to 3. IDLs varied from 0.0015 to 12.0 μg L^−1^ and the quantitative ranges extended to between 95 and 3000 μg L^−1^ utilising between one and three linear calibration ranges. Generally, macrolides achieved the lowest limits of detection and aminoglycosides the highest. The analytes with the poorest sensitivity (AMGs) elute in the first minute, within the dead volume region. The method was not optimised to accommodate these compounds, but they were kept for the intention of semi-quantitative analyses. Other drugs degraded more quickly in the sample composition (80:20 H_2_O:MeOH), notably the isoniazids and β-lactams. It was therefore important to store standards in methanol, where possible, and remake quality control mixtures before each use.

Inter- and intra-day accuracy and precision were calculated per analyte. Most performed well, 65 and 63 analytes achieved 90–110% accuracy for intra-day and inter-day studies, respectively. However, several analytes had poor concordance between samples. Approximately 50% of analytes achieved standard deviations of < 15% across both studies. This is likely due to the number of active MRM channels per cycle (a total of 120 channels within the 19-min method). To accommodate, channel windows were minimised to maximise dwell time and staggered to reduce noise. Analytes that performed worst (≥ 25% stdev) are formatted in grey text and may be considered semi-quantitative in the corresponding analysis phase. These included aminoglycosides (GEN, KAN, and CAP), β-lactams (IPM, MEM, ATM, CFM, PenG, PenV), and some TB drugs (EMB, DMD, and rifamycins). Alternate calibrations used for solids were designed to improve analyte quantitation from different matrices. Most examples have comparable instrumental performance, but some analytes were labelled as semi-quantitative during solid analysis (OTC, dmAZM, CTF, daKTC, DOX, CLI, dmCLI, dmERY, and ERY).

#### Method performance

Matrix retention times (*t*_R_) and ion ratios were obtained from spiked matrices. Only data with a signal-to-noise ratio ≥ 10 were used to calculate these parameters. Accepted tolerances for retention times were typically ±10 %. Ion ratios often varied with concentration due to differences in product ion sensitivity or channel-specific interference. Consequently, ion ratio parameters were conditional on sample concentration (stated for regions 0–100 ng L^−1^ and 100–1000 ng mL^−1^). Typically, standard deviations of validation criteria are lower at high concentrations, where matrix effects are lower. All validation parameters are outlined in ESM Tables S4a-5b. Visualisations of matrix effects in wastewater influent and SPM (via MAE) are displayed in ESM Figure S3. The overlaid chromatograms are scaled by relative intensity, per class, meaning absolute signal suppression can be observed within drug classes. Suppression, and/or poor recovery can be seen for the aminoglycosides (A), some TB drugs (B), carbapenems (C), and two macrolides and their associated metabolites (I).

Recovery from matrix was more varied due to several internal standard compatibilities. Several drugs (INHs, KTCs, BSF, penicillins, DOX, PLFX) suffered from high matrix interference, lowering reproducibility and increasing standard deviations. Some interference was only observed in the channel for product ion 1, or only in solid samples. Consequently, for solid analysis some analytes were re-calibrated using product ion 2 and several were assigned different internal standards (ESM Tables S2 and S6, respectively).

**Fig. 3:**
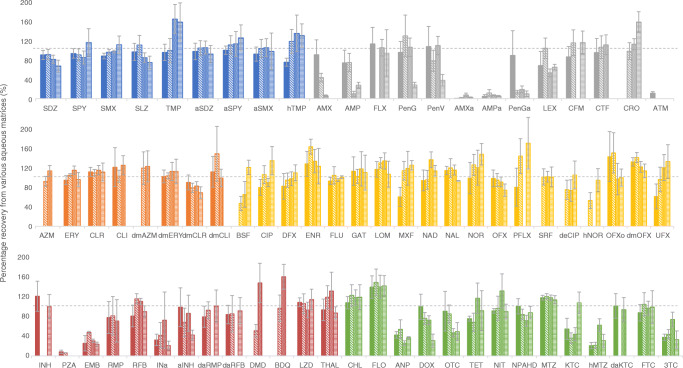
Method recovery from aqueous matrix, ordered by class (colour) and matrix (bar fill type). The drug classes are sulfonamides (blue); β-lactams (grey); macrolides and lincomycins (orange); quinolones (yellow); tuberculosis drugs (red); and others: amphenicols, cyclines, nitrofurans, azoles, and antiretrovirals (green). The matrices are Milli-Q water (solid bar); river water (diagonal bar); effluent wastewater (dotted bar); and influent wastewater (horizontal striped bar). Error bars represent replicate standard deviation (*n* ≤ 18).

**Fig. 4:**
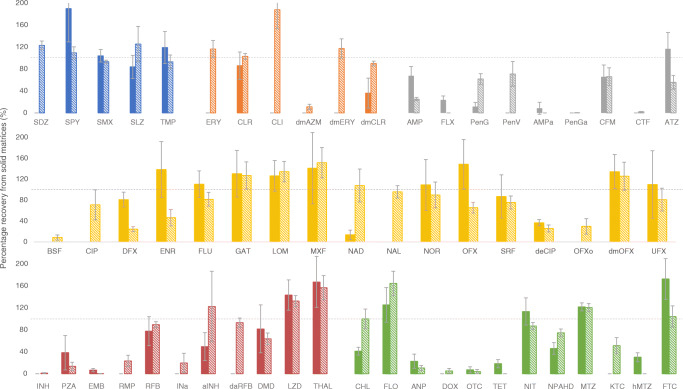
Method recovery from solid matrix, ordered by class (colour) and matrix (bar fill type). The drug classes are sulfonamides (blue); macrolides and lincomycins (orange); β-lactams (grey); quinolones (yellow); tuberculosis drugs (red); and others: amphenicols, cyclines, nitrofurans, azoles, and antiretrovirals (green). The matrices are influent suspended particulate matter (solid bar), and river sediment (diagonal bar). Error bars represent replicate standard deviation (*n* ≤ 18).

Figures 3 and 4 demonstrate the matrix recoveries for aqueous and solid extractions, respectively; numerical recoveries per matrix are outlined in ESM Table S8. Percentage recoveries were varied throughout both the drug classes and the different matrices. Sulfonamides, macrolides, lincomycins, and quinolones generally performed well, achieving good recoveries from all aqueous extractions. Some exceptions include trimethoprims (TMP and hTMP), which suffered some interference in wastewater samples, and large standard deviations in quinolones (ENR, NOR, PFLX, and OFXo). Sulfonamides, macrolides, and quinolones also achieved the best recoveries from solids, although several metabolites were excluded from the study. Concordance was poor for several analytes, largely attributed to imperfect internal standard pairings. Deuterated or C13 analogues of analytes were used as internal standards. Unfortunately, not all analytes had their labelled equivalents. Therefore, on some occasions, any analyte-specific properties (such as matrix effects, analyte stability, solid-liquid partitioning) affected the analyte and internal standard differently, causing decreased precision and/or accuracy. Percentage recoveries of < 100 are indicative of insufficient extraction, low analyte stability, or matrix interference. This was observed predominantly in metabolites, β-lactams, PZA, EMB, 3TC, and more ‘dirty’ matrices. Poor recoveries may also be biased by less suited internal standards. High standard deviation or a percentage recovery of > 100 is also indicative of this. If an internal standard has a lower recovery than its paired analyte, the analyte concentration is artificially amplified. Extreme cases of this (200+ %) were excluded, as were outliers, and analytes labelled as only semi-quantitative (e.g. aminoglycosides). Additionally, the method did not recover carbapenems (IPM & MEM) sufficiently to be quantified in matrix. hPZA and DCS were quantifiable, but the recovered quantities were indistinguishable from matrix blanks—suggesting either chromatographic interference or degradation of the analytical standard.

Method detection limits, calculated from the respective matrix recoveries, were as low as 0.0051 ng L^−1^ (dmCLI) in aqueous matrices and 0.0024 ng g^−1^ (ERY) in solids (ESM Table S9). Sulfonamides, macrolides, and azoles achieved the lowest method limits. For example, macrolide MQLs ranged from 0.017 to 10.3 ng L^−1^ in aqueous and 0.008–20.9 ng g^−1^ in solids. Several method limits were determined above the analyte’s quantitative linear range, due to very low (< 15%) recoveries—namely aminoglycosides (TB MDR) and penicillins. Yet overall, the majority of the analytes were considered quantifiable within expected urban sample concentrations.

### Application to environmental matrices

Aqueous matrices included wastewater influent, effluent, and river samples. Solid matrices included wastewater suspended particulate material (SPM), and river sediment. Samples were homogenised and analysed in physical replicate (*n* = 3), whereby each replicate was injected twice. Standard deviations were calculated from the six injections, per matrix. A summary of the sample analyses is displayed in Table 4. Most (60–80%) antibiotics and their metabolites were found in all studied matrices at concentrations varying from 0.097 to 216 ng L^−1^ for aqueous, and 0.36–68,911 ng g^−1^ for solids. Several analytes were found at levels below the corresponding method limits for detection or quantification—these were marked as such rather than quoting numerical values. The results were from singular homogenised samples; therefore, they may not be representative of typical day-to-day concentrations. Further analyses would be required to draw meaningful evaluations of water system health/contamination.

**Table 4:** Quantification of analytes in urban samples, ordered by drug class

Drug class	Abbrev	River water (ng L^−1^)	Effluent WW (ng L^−1^)	Influent WW (ng L^−1^)	Influent SPM (ng g^−1^)	River Sediment (ng g^−1^)
Sulfonamide &						
Trimethoprim	SDZ	<DL	<DL	<DL	10.9 ± 3.8	2.8 ± 0.4
	SPY	0.94 ± 0.05	2.3 ± 0.1	<QL	1215 ± 280	138 ± 16
	SMX	0.64 ± 0.11	2.5 ± 0.3	12.7 ± 2.2	8.7 ± 2.6	<QL
	SLZ	9.7 ± 0.7	15.9 ± 1.9	19.3 ± 3.8	<QL	11.9 ± 1.6
	TMP	6.0 ± 1.1	39.4 ± 8.8	82.7 ± 31.3	37.4 ± 11.4	1.9 ± 0.3
	aSDZ	<DL	ND	<DL	ND	<QL
	aSPY	<QL	7.6 ± 0.2	22.6 ± 1.5	43.3 ± 9.4	15.1 ± 2.7
	aSMX	1.7 ± 0.6	8.5 ± 1.4	52.5 ± 8.9	<QL	ND
	hTMP	<	<QL	0.39 ± 0.09	ND	<QL
Macrolide &	AZM	<QL	ND	<DL	ND	<QL
Lincomycin	ERY	9.5 ± 2.0	32.1 ± 7.7	86.5 ± 19.8	9.9 ± 11.4	<DL
	CLR	17.5 ± 1.3	77.2 ± 1.6	121 ± 13	151 ± 39	5.0 ± 0.2
	CLI	ND	<QL	97.4 ± 7.9	172 ± 88	4.0 ± 0.2
	dmAZM	ND	ND	ND	ND	<QL
	dmERY	2.2 ± 1.0	7.4 ± 2.5	61.7 ± 20.3	ND	<DL
	dmCLR	8.5 ± 0.5	21.1 ± 1.3	30.0 ± 7.3	65 ± 101	2.4 ± 1.4
	dmCLI	0.10 ± 0.03	0.38 ± 0.19	27.7 ± 5.1	2.6 ± 1.3	0.36 ± 0.03
β-LACTAMS						
Penicillin	AMX	ND	ND	ND	<QL	<DL
	AMP	<DL	ND	ND	<QL	<QL
	FLX	3.0 ± 1.3	12.3 ± 1.2	217 ± 67	ND	ND
	PenG	<QL	ND	<DL	12.0 ± 4.8	ND
	PenV	<QL	ND	<QL	ND	ND
	AMXa	ND	ND	<QL	ND	ND
	AMPa	ND	ND	ND	13.9 ± 7.2	ND
	PenGa	<QL	<QL	<QL	12.7 ± 9.3	<QL
Cefalosporin	LEX	ND	ND	ND	ND	<DL
	CFM	ND	ND	ND	<QL	<QL
	CTF	2.5 ± 0.1	<QL	ND	<QL	ND
	CRO	<QL	<QL	<QL	ND	ND
Monobactam	ATM	<QL	<QL	7.9 ± 1.2	<DL	<DL
Carbapenem	IPM	ND	ND	ND	ND	21.9 ± 5.1
	MEM	ND	11.9	13.0 ± 1.4	36.1 ± 9.0	18.2 ± 0.2
Quinolone	BSF	<QL	<QL	<QL	<QL	<QL
	CIP	ND	5.9 ± 2.3	60.6 ± 12.0	1593 ± 374	27.9 ± 18.5
	DFX	ND	ND	<QL	ND	<QL
	ENR	0.62 ± 0.56	1.8 ± 0.4	6.8 ± 6.7	10.6 ± 5.8	7.6 ± 1.9
	FLU	0.32 ± 0.16	0.45 ± 0.18	0.89 ± 0.59	3.7 ± 1.7	0.91 ± 0.33
	GAT	ND	<QL	<QL	10.7 ± 4.0	<QL
	LOM	<DL	0.76 ± 0.43	3.9 ± 2.3	9.1 ± 4.2	4.9 ± 2.4
	MXF	ND	<QL	17.0 ± 8.3	<QL	<QL
	NAD	<QL	<QL	<QL	ND	76.3 ± 13.7
	NAL	ND	0.9 ± 0.5	1.0 ± 0.5	14.3 ± 5.0	0.76 ± 0.32
	NOR	<QL	22.0 ± 27.8	13.1 ± 4.4	214 ± 47	63.2 ± 20.4
	OFX	0.72 ± 0.22	1.2 ± 0.1	5.9 ± 1.7	47.4 ± 9.1	8.6 ± 4.1
	PFLX	4.2 ± 1.0	<QL	<QL	137 ± 67	<QL
	SRF	ND	ND	<QL	4.0 ± 2.8	5.1 ± 3.7
	deCIP	ND	<QL	10.9 ± 5.4	13.8 ± 4.1	<QL
	hNOR	ND	ND	<QL	25.7 ± 12.4	6.7 ± 1.2
	OFXo	<DL	<QL	<DL	<DL	<DL
	dmOFX	<QL	<QL	<QL	8.4 ± 3.7	<QL
	UFX	<QL	<QL	<QL	<QL	<QL
TB Drugs						
TB (1st line)	INH	29.1 ± 14.8	27.8 ± 10.0	18.0 ± 4.2	43.4 ± 3.9	<QL
	PZA	ND	<QL	8.0 ± 1.3	69.4 ± 14.6	<QL
	EMB	ND	0.35 ± 0.15	0.59 ± 0.22	2.2 ± 0.5	ND
	RMP	<QL	38.4 ± 27.1	ND	<DL	181 ± 51
	RFB	ND	<QL	ND	<DL	<DL
	INa	123 ± 57	163 ± 46	133 ± 42	2180 ± 784	31.7 ± 9.7
	aINH	16.2 ± 5.2	25.8 ± 7.7	23.7 ± 9.0	5.5 ± 2.1	<QL
	hPZA	ND	<QL	186 ± 19	210 ± 170	<QL
	daRMP	ND	ND	ND	ND	ND
	daRFB	<QL	<QL	ND	<DL	ND
TB (MDR)	CAP	ND	27.4	29.7 ± 1.1	<DL	ND
	GEN	30.2 ± 0.1	2.5	24.3 ± 0.5	ND	ND
	KAN	ND	ND	<QL	308 ± 91	126.0
	STR	ND	ND	<QL	ND	ND
	DCS	<DL	<DL	0.66 ± 0.20	128 ± 29	21.5 ± 2.6
TB (other)	DMD	<QL	<QL	<QL	ND	55.7 ± 0.4
	BDQ	ND	ND	97.7 ± 38.2	<DL	<QL
	LZD	<QL	1.9 ± 0.3	3.3 ± 0.6	ND	<DL
	THAL	ND	<QL	ND	79.6 ± 30.1	ND
Other						
Amphenicol	CHL	2.8 ± 1.6	ND	ND	ND	6.8 ± 3.7
	FLO	ND	ND	ND	ND	ND
	ANP	ND	ND	ND	ND	ND
Cycline	DOX	27.4 ± 9.2	<QL	<QL	<DL	ND
	OTC	<QL	<QL	<QL	<QL	<QL
	TET	<QL	<QL	24.2 ± 4.5	86.3 ± 29.8	18.5 ± 16.7
Nitrofuran	NIT	ND	ND	ND	176 ± 82	<QL
	NPAHD	ND	<DL	ND	<QL	ND
Azole	MTZ	2.3 ± 0.1	25.4 ± 0.4	26.7 ± 0.5	ND	<QL
	KTC	ND	<QL	7.3 ± 1.5	177 ± 139	7.5 ± 5.4
	hMTZ	ND	16.3 ± 3.7	16.1 ± 1.8	68.6 ± 17.7	ND
	daKTC	ND	ND	45.8 ± 5.1	1998 ± 794	ND
ARV	FTC	4.8 ± 2.1	22.8 ± 5.8	77.6 ± 5.6	68.0 ± 18.0	28.0 ± 7.1
	3TC	ND	<QL	<QL	ND	ND

*WW* wastewater, *TB* tuberculosis, *TB MDR* multidrug resistant, *ARV* antiretroviral, *ND* not detected, *QL* quantification limit, *DL* detection limit. Per matrix, standard deviations were calculated from replicates (*n* ≤ 6); samples are not related in these examples i.e., influent aqueous does not correspond to the effluent or influent suspended particulate matter, and the river matrices do not correspond to each other

The main aim of the paper was to develop a method that allows for the simultaneous analysis of several groups of antibiotics in various environmental matrices. Special emphasis was placed on quantification of antibiotic-metabolite pairs. Ability to quantify both antibiotics and their corresponding metabolites in wastewater and in the wider environment can help with understanding patterns of consumption of antibiotics in studied areas as well as providing evidence for direct disposal of antibiotics (e.g. if the antibiotic/metabolite ratio is outside the confidence limit set using human metabolism data). Indeed, antibiotic-metabolite pairs were identified and quantified in most environmental matrices. Pairings include SDZ-aSDZ, SPY-aSPY, SMX-aSMX, and TMP-hTMP belonging to sulphonamide/trimethoprim antibiotic group; AZM-dmAZM, ERY-dmERY, CLR-dmCLR, and CLI-dmCLI in macrolides/lincomycins; AMX-AMXa, AMP-AMPa, and PenG-PenGa in β-lactams; ENR-CIP-deCIP, NOR-hNOR, OFX-dmOFX/oOFX, and PFLX-UFX in quinolones; INH-aINH/INa, PZA-hPZA, RMP-daRMP, and RFB-daRFB in TB drugs; CHL-ANP in amphenicols; NIT-NPAHD in nitrofurans; and MTZ-hMTZ and KTC-daKTC in azoles. In several cases, metabolite concentrations were higher than the antibiotics themselves.

## Conclusions

This method was developed for the quantification of 58 parent drugs (54 antibiotics, 1 antifungal, 2 antiretrovirals, and 1 TB-relevant drug) and 26 metabolites (25 antibiotics and 1 antifungal). Drug compositions are often comprised of several chemical forms, for example capreomycins IA and IB. Drugs with significant proportions (> 5%) of different mass forms were designed and validated separately, but the results were combined and quoted for the encompassing drug. Quantitative analysis was achieved for the majority of targets for the aqueous (66; > 78%) and solid (58; > 69%) calibrations but was regarded as only semi-quantitative for the remaining targets. High sample throughput was a requirement of this LC-MS method due to research design involving analysis of a large number of samples in a high-demand research laboratory. The chromatography was compressed and optimised into a 19-min gradient, for spectrometric analysis of 116 MRM channels.

Recovery of analytes from different matrices was variable. The number of analytes successfully recovered was determined per matrix. From ‘clean’ water (Milli-Q or river water), 64 were recovered quantitatively; due to validation and recovery performance, 18 were considered semi-quantitative, and 2 were not detectable. For wastewater (influent or effluent), the numbers of analytes were 63, 19, and 2, respectively, and for solids (SPM or sediment), they were 45, 37, and 2, respectively. Recovery tests were performed using one homogenised sample in triplicate per matrix. In these samples, several analytes were quoted as above their linear quantitative ranges. If the baseline level of analyte was lower, or a dilution was made, those analytes would likely produce quantitative results. Based on matrix-specific recoveries, MQLs were determined as low as 0.017 ng L^−1^ in river water, 0.044 ng L^−1^ in wastewater, 0.008 ng g^−1^ in river sediment, and 0.009 ng g^−1^ in wastewater SPM. Overall, these results demonstrate the success and versatility of the method in terms of the high number of analytes and quantitation of different environmental matrices.

In environmental samples, most analytes (60–80%) were quantified in all studied matrices at concentrations varying from 0.097 to 216 ng L^−1^ for aqueous and 0.36–68,911 ng g^−1^ for solids. Another feature of this method, and a distinction from a lot of existing WBE research, was the inclusion of drug metabolites. Several antibiotic-metabolite pairs were successfully quantified. These include four pairings belonging to sulphonamide/trimethoprim antibiotic group; four in macrolides/lincomycins; three in β-lactams; four in quinolones; four in TB drugs; one in amphenicols; one in nitrofurans; and two in azoles. The ability to quantify both antibiotics and their corresponding metabolites in wastewater and in the wider environment is of particular importance; it can help with understanding patterns of consumption of antibiotics in studied areas, as well as providing evidence for direct disposal of antibiotics. Understanding AB-MET relationships during wastewater treatment and in the wider environment will help with unravelling complexities of the environmental fate of antibiotics and resulting risks they pose, both in the context of chemical toxicity and AMR.

## Supplementary information

ESM 1 (DOCX 3576 kb)

## Data Availability

The datasets generated during and/or analysed during the current study are available from the corresponding author on reasonable request.
