# Supplementary material for: Multiresidue antibiotic-metabolite quantification method using ultra-performance liquid chromatography coupled with tandem mass spectrometry for environmental and public exposure estimation
Source: Anal Bioanal Chem. 2021 Sep 8;413(23):5901–20. doi: 10.1007/s00216-021-03573-4 (PMC8425450; doi:10.1007/s00216-021-03573-4)
Supplement: Supplementary file 1 — (DOCX 3576 kb) [file 216_2021_3573_MOESM1_ESM.docx]

**Multiresidue antibiotic-metabolite quantification method using ultra-performance liquid chromatography coupled with tandem mass spectrometry for environmental and public exposure estimation**

Analytical and Bioanalytical Chemistry

**Elizabeth Holton, Barbara Kasprzyk-Hordern**

Department of Chemistry, University of Bath, Bath, BA2 7AY, UK

**2.4.1. Liquid chromatography (supplementary information)**

**Table S1** Chemical information, ordered by class

**Table S2** Chromatographic and validation parameters for solids analyses in mobile phase, ordered by retention time (in full)

**Table S3** Liquid chromatography and mass spectrometry setup

**Table S4a** Chromatographic and validation parameters: Matrix retention times for aqueous analyses, ordered by retention time

**Table S4b** Chromatographic and validation parameters: Matrix retention times for solids analyses, ordered by retention time

**Table S5a** Chromatographic and validation parameters: Matrix ion ratios for aqueous analyses, ordered by retention time

**Table S5b** Chromatographic and validation parameters: Matrix ion ratios for solids analyses, ordered by retention time

**Table S6** Mass spectrometry parameters, ordered by retention time

**Table S7** Equations for calculating concentration, instrument performance, and method performance

**Table S8** Method recovery from urban matrices, ordered by drug class

**Table S9** Method detection and quantification limits per matrix, ordered by retention time

**Table S10** Compound structural information, grouped by drug class and corresponding drug metabolites (9 pages)

**Figure S1** Weighting schemes

**Figure S2** Split calibration ranges

**Figure S3** Overlaid chromatograms, scaled by relative intensity, highlighting regions by chemical class

**Figure S4** Overlaid chromatograms, scaled by relative intensity

*2.4.1. Liquid chromatography (supplementary information)*

The chromatography was optimised in terms of mobile phase composition, needle washes, sample composition, flow rate, and inlet gradient (Online resource T2).

A starting mobile phase of water/methanol with 0.1% formic acid produced the strongest ionisation across the range of analytes in positive ESI mode. Outperforming other buffers such as ammonium acetate (5mM NH_4_OAc) with 0.3 % acetic acid, and ammonium fluoride (1mM NH_4_F). The preparation of water and methanol was also tested at several ratios for the aqueous (A) line, such as 80:20 and 60:40 H_2_O:MeOH. The best separation and organic purge was achieved using 95:5 H_2_O:MeOH with 0.1 % formic acid (mobile phase A) and 100 % MeOH (mobile phase B).

Strong and weak needle washes were selected to compliment the system. The strong wash used was 1:1:1:1 MeOH:ACN:IPA:H_2_O + 0.1 % formic acid and the weak wash was prepared to mimic the starting mobile phase (95:5 H_2_O:MeOH). The injection volume was 20 µL, via a partial loop injection.

Five ratios were assessed for the sample make-up (0:100, 20:80, 40:60, 60:40, and 80:20 H_2_O:MeOH). At high concentrations of organic modifier, the effects of longitudinal diffusion were reduced, which increased analyte velocity and sharpened peaks. But as analyte retention decreased, peak splitting occurred upon solvent mixing. Sample composition performed best at 60:40 but was prepared at 80:20 H_2_O:MeOH to enable samples to be co-analysed with pre-established methods.

The method flow rate was tested across the range of 0.04 - 0.3 mL min^-1^. The best results, in terms of system pressure, peak separation, and peak shape; were determined at 0.2 mL min^-1^.

Numerous elution profiles were tested to maximise overall peak separation whilst retaining Gaussian shape. Starting conditions were: 0 % B, held for 1 min; followed by an 8.5 min gradient to 40 % B; 3.5 min gradient to 100 % B; 3 min hold; finally returning to 0 % B (0.5 min) to re-equilibrate for 2.5 min. Two gradients were utilised; operating at a gradual rate during aqueous-based elution (0-40 % B), followed by a much steeper gradient towards the high-organic hold (40-100 % B). The former enhanced the initial peak separation, and the latter vastly shortened the overall chromatographic period. The re-equilibration hold (2.5 min), plus LC pre-injection liquid handling (needle wash cycles, sample aspiration, etc. approx. 0.5 min), was optimised as the minimum acceptable duration: equivalent to >5 column volumes. System pressures consistently reached equilibrium over this duration and analyte carryover was negligible. The total run time was 19 min. Figure 2 shows the distribution of peaks relative to the inlet gradient and internal standard retention times (top); as well as the example chromatographs, organised by drug class (bottom).

| **Table S1** Chemical information, ordered by class groupings | | | | |  | |  | |  | |  | |  | |  | |  |
| --- | --- | --- | --- | --- | --- | --- | --- | --- | --- | --- | --- | --- | --- | --- | --- | --- | --- |
|  | | | | |  | |  | |  | |  | |  | |  | |  |
| **Grouping** | | **Chemical** | **Abbrev** | **Class A** | | **Class B** | | **CAS No.** | | **Salt form** θ | | **Molec. Formula** | | **M.I. mass** | | **Supplier** | |
| Sulfonamide & | | Sulfadiazine | SDZ | Sulfonamide | | Parent | | 68-35-9 | |  | | C10H10N4O2S | | 250.05 | | Sigma-Aldrich | |
| Trimethoprim | | Sulfapyridine | SPY | Sulfonamide | | Parent | | 144-83-2 | |  | | C11H11N3O2S | | 249.06 | | Sigma-Aldrich | |
|  | | Sulfamethoxazole | SMX | Sulfonamide | | Parent | | 723-46-6 | |  | | C10H11N3O3S | | 253.05 | | Sigma-Aldrich | |
|  | | Sulfasalazine | SLZ | Sulfonamides | | Parent | | 599-79-1 | |  | | C18H14N4O5S | | 398.07 | | Sigma-Aldrich | |
|  | | Trimethoprim | TMP | Trimethoprim | | Parent | | 738-70-5 | |  | | C14H18N4O3 | | 290.14 | | Sigma-Aldrich | |
|  | | N-acetyl sulfadiazine | aSDZ | Sulfonamide | | Metabolite | | 127-74-2 | |  | | C12H12N4O3S | | 292.06 | | TRC | |
|  | | N-acetyl sulfapyridine | aSPY | Sulfonamide | | Metabolite | | 19077-98-6 | |  | | C13H13N3O3S | | 291.07 | | TRC | |
|  | | N-acetyl sulfamethoxazole | aSMX | Sulfonamide | | Metabolite | | 21312-10-7 | |  | | C12H13N3O4S | | 295.06 | | TRC | |
|  | | 4-hydroxy-trimethoprim | hTMP | Trimethoprim | | Metabolite | | 112678-48-5 | |  | | C14H18N4O4 | | 306.13 | | TRC | |
| Macrolide | | Azithromycin | AZM | Macrolide | | Parent | | 83905-01-5 | |  | | C38H72N2O12 | | 748.51 | | LCG | |
| & Lincomycin | | Erythromycin | ERY | Macrolide | | Parent | | 114-07-8 | |  | | C37H67NO13 | | 733.46 | | Sigma-Aldrich | |
|  | | Clarithromycin | CLR | Macrolide | | Parent | | 81103-11-9 | |  | | C38H69NO13 | | 747.48 | | Sigma-Aldrich | |
|  | | Clindamycin | CLI | Lincomycin | | Parent | | 18323-44-9 | | Hydrochloride | | C18H33ClN2O5S | | 424.18 | | Sigma-Aldrich | |
|  | | N-desmethyl azithromycin | dmAZM | Macrolide | | Metabolite | | 172617-84-4 | |  | | C37H70N2O12 | | 734.49 | | TRC | |
|  | | N-desmethyl erythromycin A | dmERY | Macrolide | | Metabolite | | 992-62-1 | |  | | ‎C36H65NO13 | | 719.45 | | TRC | |
|  | | N-desmethyl clarithromycin | dmCLR | Macrolide | | Metabolite | | 101666-68-6 | |  | | C37H67NO13 | | 733.46 | | TRC | |
|  | | N-desmethyl clindamycin | dmCLI | Lincomycin | | Metabolite | | 22431-45-4 | |  | | C17H31ClN2O5S | | 410.16 | | TRC | |
| **β-LACTAMS** | |  |  |  | |  | |  | |  | |  | |  | |  | |
| Penicillin | | Amoxicillin | AMX | Penicillin | | Parent | | 26787-78-0 | |  | | C16H19N3O5S | | 365.10 | | Fluka | |
|  | | Ampicillin | AMP | Penicillin | | Parent | | 69-53-4 | | Trihydrate | | C16H19N3O4S | | 349.11 | | Fluka | |
|  | | Flucloxacillin | FLX | Penicillin | | Parent | | 5250-39-5 | | Sodium | | C19H17ClFN3O5S | | 453.06 | | Fluka | |
|  | | Penicillin G | PenG | Penicillin | | Parent | | 113-98-4 | | Sodium | | C16H18N2O4S | | 334.10 | | Fluka | |
|  | | Penicillin V | PenV | Penicillin | | Parent | | 132-98-9 | | Potassium | | C16H18N2O5S | | 350.09 | | Sigma-Aldrich | |
|  | | Amoxicilloic acid | AMXa | Penicillin | | Metabolite | | 42947-63-7 ¥ | | Trisodium salt | | C16H21N3O6S | | 383.12 | | TRC | |
|  | | Ampicilloic acid | AMPa | Penicillin | | Metabolite | | 32746-94-4 | |  | | C16H21N3O5S | | 367.12 | | TRC | |
|  | | Penicilloic G acid | PenGa | Penicillin | | Metabolite | | 11039-68-2 | |  | | C9H14N2O5S | | 262.06 | | TRC | |
| Cefalosporin | | Cefalexin | LEX | Cefalosporin | | Parent | | 23325-78-2 | | Monohydrate | | C16H17N3O4S | | 347.09 | | Fluka | |
|  | | Cefixime | CFM | Cefalosporin | | Parent | | 79350-37-1 | | Trihydrate | | C16H15N5O7S2 | | 453.04 | | Fluka | |
|  | | Ceftiofur | CTF | Cefalosporin | | Parent | | 104010-37-9 | |  | | ‎C19H17N5O7S3 | | 523.03 | | Fluka | |
|  | | Ceftriaxone | CRO | Cefalosporin | | Parent | | 104376-79-6 | | Disodium hemi(heptahydrate) | | C18H18N8O7S3 | | 554.05 | | Sigma-Aldrich | |
| Monobactam | | Aztreonam | ATM | Monobactam | | Parent | | 78110-38-0 | |  | | C13H17N5O8S2 | | 435.05 | | TRC | |
| Carbapenem | | Imipenem | IPM | Carbapenem | | Parent | | 64221-86-9 | |  | | C12H17N3O4S | | 299.09 | | Sigma-Aldrich | |
|  | | Meropenem | MEM | Carbapenem | | Parent | | 119478-56-7 | | Trihydrate | | C17H25N3O5S | | 383.15 | | Sigma-Aldrich | |
| Quinolone | | Besifloxacin | BSF | Quinolone | | Parent | | 405165-61-9 | | Hydrochloride | | C19H21ClFN3O3 | | 393.13 | | MCE | |
|  | | Ciprofloxacin | CIP | Quinolone | | Parent/Metab. | | 85721-33-1 | |  | | C17H18FN3O3 | | 331.13 | | Fluka | |
|  | | Danofloxacin | DFX | Quinolone | | Parent | | 119478-55-6 | | Mesylate | | C19H20FN3O3 | | 357.15 | | LCG | |
|  | | Enrofloxacin | ENR | Quinolone | | Parent | | 93106-60-6 | |  | | C19H22FN3O3 | | 359.16 | | Sigma-Aldrich | |
|  | | Flumequine | FLU | Quinolone | | Parent | | 42835-25-6 | |  | | C14H12FNO3 | | 261.08 | | Fluka | |
|  | | Gatifloxacin | GAT | Quinolone | | Parent | | 112811-59-3 | |  | | C19H22FN3O4 | | 375.16 | | TRC | |
|  | | Lomefloxacin | LOM | Quinolone | | Parent | | 98079-52-8 | | Hydrochloride | | C17H19F2N3O3 | | 351.14 | | Sigma-Aldrich | |
|  | | Moxifloxacin | MXF | Quinolone | | Parent | | 268545-13-7 | | Hydrochloride | | C21H24FN3O4 | | 401.18 | | MCE | |
|  | | Nadifloxacin | NAD | Quinolone | | Parent | | 124858-35-1 | |  | | C19H21FN2O4 | | 360.15 | | MCE | |
|  | | Nalidixic acid | NAL | Quinolone | | Parent | | 389-08-2 | | Sodium | | C12H12N2O3 | | 232.08 | | Sigma-Aldrich | |
|  | | Norfloxacin | NOR | Quinolone | | Parent | | 70458-96-7 | |  | | C16H18FN3O3 | | 319.13 | | Sigma-Aldrich | |
|  | | Ofloxacin (Levofloxacin) * | OFX | Quinolone | | Parent | | 82419-36-1 | |  | | C18H20FN3O4 | | 361.14 | | Sigma-Aldrich | |
|  | | Prulifloxacin | PFLX | Quinolone | | Parent (prodrug) | | 123447-62-1 | |  | | C21H20FN3O6S | | 461.11 | | Sigma-Aldrich | |
| **Grouping** | | **Chemical** | **Abbrev** | **Class A** | | **Class B** | | **CAS No.** | | **Salt form θ** | | **Molec. Formula** | | **M.I. mass** | | **Supplier** | |
|  | | Sarafloxacin | SRF | Quinolone | | Parent | | 91296-87-6 | | Hydrochloride | | C20H17F2N3O3 | | 385.12 | | Sigma-Aldrich | |
|  | | Desethylene ciprofloxacin | deCIP | Quinolone | | Metabolite | | 528851-31-2 | | Hydrochloride | | C15H16FN3O3 | | 305.12 | | TRC | |
|  | | Hydroxy-norfloxacin | hNOR | Quinolone | | Metabolite | | 109142-49-6 | |  | | C16H18FN3O4 | | 335.13 | | TRC | |
|  | | Ofloxacin N-oxide | OFXo | Quinolone | | Metabolite | | 104721-52-0 | | Acetic acid salt | | C18H20FN3O5 | | 377.14 | | TRC | |
|  | | Desmethyl-ofloxacin | dmOFX | Quinolone | | Metabolite | | 82419-52-1 | |  | | C17H18FN3O4 | | 347.13 | | TRC | |
|  | | Ulifloxacin | UFX | Quinolone | | Metabolite | | 112984-60-8 | |  | | C16H16FN3O3S | | 349.09 | | TRC | |
| TB (1st line) | | Isoniazid | INH | Isoniazid | | Parent | | 54-85-3 | |  | | C6H7N3O | | 137.06 | | Sigma-Aldrich | |
|  | | Pyrazinamide | PZA | Pyrazinamide | | Parent | | 98-96-4 | |  | | C5H5N3O | | 123.04 | | Sigma-Aldrich | |
|  | | Ethambutol | EMB | Ethambutol | | Parent | | 74-55-5 | | Dihydrochloride | | C10H24N2O2 | | 204.18 | | Sigma-Aldrich | |
|  | | Rifampicin | RMP | Rifamycin | | Parent | | 13292-46-1 | |  | | C43H58N4O12 | | 822.41 | | Sigma-Aldrich | |
|  | | Rifabutin | RFB | Rifamycin | | Parent | | 72559-06-9 | |  | | C46H62N4O11 | | 846.44 | | Sigma-Aldrich | |
|  | | Isonicotinic acid | INa | Isoniazid | | Metabolite | | 55-22-1 | |  | | C6H5NO2 | | 123.03 | | Sigma-Aldrich | |
|  | | Acetyl-isoniazid | aINH | Isoniazid | | Metabolite | | 1078-38-2 | |  | | C8H9N3O2 | | 179.07 | | Sigma-Aldrich | |
|  | | 5-Hydroxy-pyrazinoic acid | hPZA | Pyrazinamide | | Metabolite | | 34604-60-9 | |  | | C5H4N2O3 | | 140.02 | | Sigma-Aldrich | |
|  | | 25-desacetyl rifampicin | daRMP | Rifamycin | | Metabolite | | 16783-99-6 | |  | | C41H56N4O11 | | 780.39 | | Sigma-Aldrich | |
|  | | 25-O-desacetyl rifabutin | daRFB | Rifamycin | | Metabolite | | 100324-63-8 | |  | | C44H60N4O10 | | 804.43 | | TRC | |
| TB (MDR) | | Capreomycin IA ≠ | CAPIa | Aminoglycoside | | Parent | | 1405-37-4 | | Sulfate | | C25H44N14O8 | | 668.35 | | TRC | |
|  | | Capreomycin IB ≠ | CAPIb | Aminoglycoside | | Parent | | 1405-37-4 | | Sulfate | | C25H44N14O7 | | 652.35 | | TRC | |
|  | | Gentamycin C1 ≠ | GEN1 | Aminoglycoside | | Parent | | 1405-41-0 | | Sulfate salt hydrate | | C21H43N5O7 | | 477.32 | | Fluka | |
|  | | Gentamycin C1a ≠ | GEN1a | Aminoglycoside | | Parent | | 1405-41-0 | | Sulfate salt hydrate | | C19H39N5O7 | | 449.28 | | Fluka | |
|  | | Gentamycin C2 C2a C2b ≠ | GEN2 | Aminoglycoside | | Parent | | 1405-41-0 | | Sulfate salt hydrate | | C20H43N5O7 | | 465.32 | | Fluka | |
|  | | Kanamycin A ≠ | KAN | Aminoglycoside | | Parent | | 25389-94-0 | | Sulfate | | C18H36N4O11 | | 484.24 | | Sigma-Aldrich | |
|  | | Streptomycin A ≠ | STR | Aminoglycoside | | Parent | | 3810-74-0 | | Sulfate | | C21H39N7O12 | | 581.27 | | Sigma-Aldrich | |
|  | | D-cycloserine | DCS | Isoxazole | | Parent/Metab. | | 68-41-7 | |  | | C3H6N2O2 | | 102.04 | | TRC | |
| TB (other) | | Delamanid | DMD | Nitroimidazole | | Parent | | 681492-22-8 | |  | | C25H25F3N4O6 | | 534.17 | | Sigma-Aldrich | |
|  | | Bedaquiline | BDQ | Diarylquinoline | | Parent | | 843663-66-1 | |  | | C32H31BrN2O2 | | 554.16 | | Sigma-Aldrich | |
|  | | Linezolid | LZD | Oxazolidinone | | Parent | | 165800-03-3 | |  | | C16H20FN3O4 | | 337.14 | | Sigma-Aldrich | |
|  | | Thalidomide | THAL | Thalidomide | | Parent | | 50-35-1 | |  | | C13H10N2O4 | | 258.06 | | Sigma-Aldrich | |
| **OTHER** | |  |  |  | |  | |  | |  | |  | |  | |  | |
| Amphenicol | | Chloramphenicol | CHL | Amphenicol | | Parent | | 56-75-7 | |  | | C11H12Cl2N2O5 | | 322.01 | | Sigma-Aldrich | |
|  | | Florfenicol | FLO | Amphenicol | | Parent | | 73231-34-2 | |  | | C12H14Cl2FNO4S | | 357.00 | | MCE | |
|  | | 2-Amino-1-(4-nitrophenyl)-1,3-propanediol | ANP | Amphenicol | | Metabolite | | 2964-48-9 | |  | | C9H12N2O4 | | 212.08 | | Sigma-Aldrich | |
| Cycline | | Doxycycline | DOX | Cycline | | Parent | | 24390-14-5 | | Hyclate | | C22H24N2O8 | | 444.15 | | Sigma-Aldrich | |
|  | | Oxytetracycline | OTC | Cycline | | Parent | | 2058-46-0 | | Hydrochloride | | C22H24N2O9 | | 460.15 | | TRC | |
|  | | Tetracycline | TET | Cycline | | Parent | | 64-75-5 | | Hydrochloride | | C22H24N2O8 | | 444.15 | | Sigma-Aldrich | |
| Nitrofuran | | Nitrofurantoin | NIT | Nitrofuran | | Parent | | 67-20-9 | |  | | C8H6N4O5 | | 238.03 | | Sigma-Aldrich | |
|  | | 1-(2-nitrobenzylidenamino)-2,4-imidazolidinedione | NPAHD | Nitrofuran | | Metabolite | | 623145-57-3 | |  | | C10H8N4O4 | | 248.05 | | TRC | |
| Azole | | Metronidazole | MTZ | Azole | | Parent | | 443-48-1 | |  | | C6H9N3O3 | | 171.06 | | Sigma-Aldrich | |
|  | | Ketoconazole | KTC | Azole | | Parent | | 65277-42-1 | |  | | C26H28Cl2N4O4 | | 530.15 | | Sigma-Aldrich | |
|  | | Hydroxy-metronidazole | hMTZ | Azole | | Metabolite | | 1215071-08-1 | |  | | C6H9N3O4 | | 187.06 | | TRC | |
|  | | Deacetyl-ketoconazole | daKTC | Azole | | Metabolite | | 67914-61-8 | |  | | C24H26Cl2N4O3 | | 488.14 | | TRC | |
| Antiretroviral | | Emtricitabine | FTC | ARV | | Parent | | 143491-57-0 | |  | | C8H10FN3O3S | | 247.04 | | TRC | |
|  | | Lamivudine | 3TC | ARV | | Parent | | 134678-17-4 | |  | | C8H11N3O3S | | 229.05 | | TRC | |
|  |  | | | | | | | | | | | | | | | | |

Monoisotopic mass (M.I. mass), multi-drug resistant (MDR), tuberculosis (TB), nucleoside reverse transcriptase inhibitor (NRTI)

LC-MS method is not chiral (*); one standard used for all forms within the drug complex (≠); CAS for chiral free acid (¥); salt corrections considered in all calculations, i.e., analysis of the free base (θ)

| **Table S2** Chromatographic and validation parameters for solids analyses in mobile phase, ordered by retention time (grey text: parameters are equivalent for aqueous and solid analyses) | | | | | | | | | | | | | | | | | | | | | | | |  |
| --- | --- | --- | --- | --- | --- | --- | --- | --- | --- | --- | --- | --- | --- | --- | --- | --- | --- | --- | --- | --- | --- | --- | --- | --- |
|  |  |  | Weighted linear calibration curves (µg L^-1^) and associated r^2^ value | | | | | | | | |  | | | Absolute and relative retention times (min) ≠ | | | | | | |  | Ion Ratio (by range, µg L^-1^) | |
| **#** | **Chemical** | **Abbrev** | **Range 1** | **r^2^** | **Range 2** | **r^2^** | **Range 3** | **r^2^** | |  | | | **STD t_R(abs)_** | | | **STD t_R(rel)_** | | **Corresponding ISTD** | | **ISTD t_R(abs)_** | |  | **0-100** | **100-1000** |
| 1 | Gentamycin C1 | GEN1 | 9.400-150 | 0.807 | 150-375 | 0.975 | - | - | |  | | | 0.52±0.1 | | | 6.30±0.6 | | Metronidazole D4 | | 3.25±0.3 | |  | #N/A | #N/A |
| 2 | Gentamycin C1a | GEN1a | 6.900-55 | 0.964 | 55-275 | 0.955 | - | - | |  | | | 0.54±0.1 | | | 6.05±0.6 | | Metronidazole D4 | | 3.25±0.3 | |  | #N/A | #N/A |
| 3 | Gentamycin C2 C2a C2b | GEN2 | 17.500-140 | 0.920 | 140-350 | 0.986 | - | - | |  | | | 0.54±0.1 | | | 6.08±0.9 | | Metronidazole D4 | | 3.25±0.3 | |  | #N/A | #N/A |
| 4 | Kanamycin A | KAN | 11.875-95 | 0.927 | 95-950 | 0.992 | - | - | |  | | | 0.54±0.1 | | | 6.02±0.6 | | Metronidazole D4 | | 3.25±0.3 | |  | #N/A | #N/A |
| 5 | Capreomycin IA | CAPIa | 22.071-441 | 0.964 | - | - | - | - | |  | | | 0.55±0.1 | | | 5.97±0.6 | | Metronidazole D4 | | 3.25±0.3 | |  | #N/A | #N/A |
| 6 | Capreomycin IB | CAPIb | 22.071-441 | 0.965 | - | - | - | - | |  | | | 0.56±0.1 | | | 5.86±0.6 | | Metronidazole D4 | | 3.25±0.3 | |  | #N/A | #N/A |
| 7 | Streptomycin A | STR | 40.000-800 | 0.979 | - | - | - | - | |  | | | 0.57±0.1 | | | 5.69±0.6 | | Metronidazole D4 | | 3.25±0.3 | |  | #N/A | #N/A |
| 8 | Ethambutol | EMB | 0.010-100 | 0.999 | 100-200 | 0.996 | 200-1000 | 0.994 | |  | | | 0.65±0.1 | | | 5.02±0.5 | | Metronidazole D4 | | 3.25±0.3 | |  | #N/A | #N/A |
| 9 | D-cycloserine | DCS | 1.000-750 | 0.997 | - | - | - | - | |  | | | 0.66±0.1 | | | 4.94±0.5 | | Metronidazole D4 | | 3.25±0.3 | |  | 0.3±0.05 | 0.3±0.04 |
| 10 | Imipenem | IPM | 5.000-500 | 0.993 | - | - | - | - | |  | | | 0.72±0.1 | | | 4.57±0.5 | | Metronidazole D4 | | 3.25±0.3 | |  | 1.2±0.2 | 1.0±0.1 |
| 11 | Isoniazid | INH | 0.500-200 | 0.997 | 200-1800 | 0.995 | - | - | |  | | | 0.74±0.1 | | | 4.33±0.4 | | Metronidazole D4 | | 3.25±0.3 | |  | 7.9±0.8 | 8.8±0.9 |
| 12 | Isonicotinic acid | INa | 0.500-200 | 0.999 | 200-3000 | 0.995 | - | - | |  | | | 0.75±0.1 | | | 4.48±0.4 | | Metronidazole D4 | | 3.25±0.3 | |  | 3.9±0.6 | 3.8±0.4 |
| 13 | Acetyl-isoniazid | aINH | 0.500-100 | 0.992 | 100-1000 | 0.996 | - | - | |  | | | 0.79±0.1 | | | 4.12±0.4 | | Metronidazole D4 | | 3.25±0.3 | |  | 0.5±0.1 | 0.5±0.1 |
| 14 | 5-Hydroxy-pyrazinoic acid | hPZA | 1.000-750 | 0.998 | - | - | - | - | |  | | | 0.84±0.2 | | | 3.98±0.8 | | Metronidazole D4 | | 3.25±0.3 | |  | 4.5±1.8 | 14.2±1.4 |
| 15 | Pyrazinamide | PZA | 1.250-1000 | 0.998 | - | - | - | - | |  | | | 1.47±0.4 | | | 2.47±1.0 | | Metronidazole D4 | | 3.25±0.3 | |  | 1.0±0.1 | 1.3±0.1 |
| 16 | 2-Amino-1-(4-nitrophenyl)-1,3-propanediol | ANP | 5.000-25 | 0.997 | 25-100 | 0.996 | 100-1000 | 0.997 | |  | | | 2.05±0.2 | | | 1.59±0.2 | | Metronidazole D4 | | 3.25±0.3 | |  | 2.4±0.4 | 2.4±0.2 |
| 17 | Lamivudine | 3TC | 1.000-1000 | 0.995 | - | - | - | - | |  | | | 2.30±0.6 | | | 1.66±0.3 | | Metronidazole D4 | | 3.25±0.3 | |  | 6.3±0.9 | 7.4±0.7 |
| 18 | Hydroxy-metronidazole | hMTZ | 0.100-100 | 0.995 | 100-750 | 0.998 | - | - | |  | | | 2.32±0.2 | | | 1.40±0.1 | | Metronidazole D4 | | 3.25±0.3 | |  | 1.4±0.2 | 1.3±0.1 |
| 19 | Metronidazole | MTZ | 0.100-500 | 0.997 | 500-1000 | 0.997 | - | - | |  | | | 3.30±0.3 | | | 0.99±0.1 | | Metronidazole D4 | | 3.25±0.3 | |  | 1.8±0.3 | 1.8±0.2 |
| 20 | Amoxicilloic acid | AMXa | 1.000-1000 | 0.998 | - | - | - | - | |  | | | 3.50±0.3 | | | 2.00±0.2 | | Sulfamethoxazole D4 | | 6.97±0.7 | |  | 1.3±0.3 | 1.3±0.1 |
| 21 | Emtricitabine | FTC | 0.500-200 | 0.996 | 50-1000 | 0.998 | 1000-3000 | 0.985 | |  | | | 3.63±0.4 | | | 0.90±0.1 | | Metronidazole D4 | | 3.25±0.3 | |  | 12.4±2.5 | 13.2±1.3 |
| 22 | Sulfadiazine | SDZ | 0.050-1000 | 0.999 | - | - | - | - | |  | | | 3.70±0.4 | | | 1.74±0.2 | | Sulfamethoxazole D4 | | 6.97±0.7 | |  | 0.9±0.1 | 0.9±0.1 |
| 23 | Amoxicillin | AMX | 5.000-100 | 0.995 | 100-1000 | 0.997 | - | - | |  | | | 3.85±0.4 | | | 1.81±0.2 | | Sulfamethoxazole D4 | | 6.97±0.7 | |  | 3.3±0.7 | 2.9±0.3 |
| 24 | Sulfapyridine | SPY | 0.010-1000 | 0.999 | - | - | - | - | |  | | | 4.50±0.5 | | | 1.47±0.1 | | Sulfamethoxazole D4 | | 6.97±0.7 | |  | 1.2±0.1 | 1.2±0.1 |
| 25 | Meropenem | MEM | 5.000-500 | 0.992 | - | - | - | - | |  | | | 5.24±0.5 | | | 1.13±0.1 | | Trimethoprim D9 | | 5.91±0.6 | |  | 6.3±0.9 | 6.2±0.6 |
| 26 | N-acetyl sulfadiazine | aSDZ | 0.070-25 | 0.996 | 25-750 | 0.998 | - | - | |  | | | 5.54±0.6 | | | 1.26±0.1 | | Sulfamethoxazole D4 | | 6.97±0.7 | |  | 1.5±0.3 | 1.5±0.2 |
| 27 | Nitrofurantoin | NIT | 5.000-1000 | 0.998 | - | - | - | - | |  | | | 5.54±0.6 | | | 1.00±0.1 | | Nitrofurantoin 13C3 | | 5.54±0.6 | |  | 1.8±0.3 | 1.9±0.2 |
| 28 | Aztreonam | ATM | 1.000-750 | 0.996 | - | - | - | - | |  | | | 5.85±0.6 | | | 1.01±0.1 | | Trimethoprim D9 | | 5.91±0.6 | |  | 0.9±0.2 | 0.8±0.1 |
| 29 | Ampicilloic acid | AMPa | 0.500-750 | 0.997 | - | - | - | - | |  | | | 6.01±0.6 | | | 1.16±0.1 | | Sulfamethoxazole D4 | | 6.97±0.7 | |  | 2.0±0.4 | 1.8±0.2 |
| 30 | Trimethoprim | TMP | 0.500-500 | 0.996 | - | - | - | - | |  | | | 6.01±0.6 | | | 0.98±0.1 | | Trimethoprim D9 | | 5.91±0.6 | |  | 0.9±0.1 | 0.9±0.1 |
| 31 | 4-hydroxy-trimethoprim | hTMP | 0.013-63 | 0.996 | 63-95 | 0.994 | - | - | |  | | | 6.15±0.6 | | | 0.96±0.1 | | Trimethoprim D9 | | 5.91±0.6 | |  | 3.9±0.4 | 3.9±0.4 |
| 32 | N-acetyl sulfapyridine | aSPY | 0.500-25 | 0.996 | 25-750 | 0.997 | - | - | |  | | | 6.19±0.6 | | | 1.13±0.1 | | Sulfamethoxazole D4 | | 6.97±0.7 | |  | 0.9±0.1 | 0.9±0.1 |
| 33 | Tetracycline | TET | 0.500-200 | 0.996 | 200-500 | 0.995 | - | - | |  | | | 6.55±0.7 | | | 1.03±0.1 | | Desmethyl-ofloxacin D8 | | 6.76±0.7 | |  | 2.3±0.5 | 2.1±0.2 |
| 34 | Ofloxacin (Levofloxacin) | OFX | 0.100-750 | 0.995 | - | - | - | - | |  | | | 6.59±0.7 | | | 1.00±0.1 | | Ofloxacin D3 | | 6.58±0.7 | |  | 1.1±0.2 | 1.0±0.1 |
| 35 | Desethylene ciprofloxacin | deCIP | 0.500-100 | 0.998 | 100-500 | 0.999 | - | - | |  | | | 6.71±0.7 | | | 1.01±0.1 | | Desmethyl-ofloxacin D8 | | 6.76±0.7 | |  | 2.6±0.5 | 2.4±0.2 |
| 36 | Oxytetracycline | OTC | 1.000-200 | 0.998 | 200-500 | 0.994 | - | - | |  | | | 6.77±0.7 | | | 1.00±0.1 | | Desmethyl-ofloxacin D8 | | 6.76±0.7 | |  | 3.9±0.8 | 3.8±0.4 |
| 37 | Desmethyl-ofloxacin | dmOFX | 0.500-50 | 0.994 | 50-750 | 0.994 | - | - | |  | | | 6.82±0.7 | | | 0.99±0.1 | | Desmethyl-ofloxacin D8 | | 6.76±0.7 | |  | 1.2±0.2 | 1.1±0.1 |
| 38 | Ceftriaxone | CRO | 25.000-1000 | 0.994 | - | - | - | - | |  | | | 6.82±0.7 | | | 0.86±0.1 | | Trimethoprim D9 | | 5.91±0.6 | |  | 0.8±0.2 | 0.8±0.2 |
| 39 | Cefalexin | LEX | 1.250-100 | 0.994 | 100-750 | 0.997 | - | - | |  | | | 6.92±0.7 | | | 0.85±0.1 | | Trimethoprim D9 | | 5.91±0.6 | |  | 0.9±0.2 | 0.9±0.1 |
| 40 | Florfenicol | FLO | 10.000-200 | 0.988 | 200-750 | 0.996 | - | - | |  | | | 6.92±0.7 | | | 1.24±0.1 | | Chloramphenicol D5 | | 8.60±0.9 | |  | 4.9±1.0 | 5.5±0.8 |
| 41 | Norfloxacin | NOR | 0.010-200 | 0.992 | 200-500 | 0.993 | 500-1000 | 0.983 | |  | | | 7.00±0.7 | | | 0.97±0.1 | | Desmethyl-ofloxacin D8 | | 6.76±0.7 | |  | 1.4±0.3 | 1.3±0.1 |
| 42 | Sulfamethoxazole | SMX | 0.010-200 | 0.995 | 200-1000 | 0.997 | - | - | |  | | | 7.02±0.7 | | | 0.99±0.1 | | Sulfamethoxazole D4 | | 6.97±0.7 | |  | 1.3±0.3 | 1.2±0.1 |
| 43 | Ciprofloxacin | CIP | 0.500-400 | 0.996 | - | - | - | - | |  | | | 7.14±0.7 | | | 0.95±0.1 | | Desmethyl-ofloxacin D8 | | 6.76±0.7 | |  | 4.0±0.6 | 5.1±0.8 |
| *Table S2 continued* | | | | | | | | | | | | | | | | | | | | | | | |  |
| **#** | **Chemical** | **Abbrev** | **Range 1** | **r^2^** | **Range 2** | **r^2^** | **Range 3** | | **r^2^** | |  | | | **STD t_R(abs)_** | | | **STD t_R(rel)_** | | **Corresponding ISTD** | | **ISTD t_R(abs)_** |  | **0-100** | **100-1000** |
| 44 | Enrofloxacin | ENR | 0.100-100 | 0.992 | 100-750 | 0.992 | - | | - | |  | | | 7.20±0.7 | | | 0.94±0.1 | | Desmethyl-ofloxacin D8 | | 6.76±0.7 |  | 2.4±0.5 | 2.5±0.2 |
| 45 | Danofloxacin | DFX | 5.000-750 | 0.997 | - | - | - | | - | |  | | | 7.34±0.7 | | | 0.92±0.1 | | Desmethyl-ofloxacin D8 | | 6.76±0.7 |  | 3.1±1.6 | 11.4±3.4 |
| 46 | Lomefloxacin | LOM | 0.100-500 | 0.997 | - | - | - | | - | |  | | | 7.44±0.7 | | | 0.91±0.1 | | Desmethyl-ofloxacin D8 | | 6.76±0.7 |  | 2.2±0.4 | 2.2±0.2 |
| 47 | Ampicillin | AMP | 5.000-1000 | 0.998 | - | - | - | | - | |  | | | 7.49±0.7 | | | 0.93±0.1 | | Sulfamethoxazole D4 | | 6.97±0.7 |  | 0.2±0.03 | 0.2±0.02 |
| 48 | Thalidomide | THAL | 1.000-100 | 0.998 | 100-750 | 0.998 | - | | - | |  | | | 7.87±0.8 | | | 0.75±0.1 | | Trimethoprim D9 | | 5.91±0.6 |  | 2.4±0.2 | 2.3±0.2 |
| 49 | Ulifloxacin | UFX | 5.000-500 | 0.986 | - | - | - | | - | |  | | | 7.90±0.8 | | | 0.86±0.1 | | Desmethyl-ofloxacin D8 | | 6.76±0.7 |  | 1.0±0.2 | 1.1±0.1 |
| 50 | Sarafloxacin | SRF | 0.500-1000 | 0.998 | - | - | - | | - | |  | | | 7.92±0.8 | | | 0.85±0.1 | | Desmethyl-ofloxacin D8 | | 6.76±0.7 |  | 0.6±0.1 | 0.4±0.1 |
| 51 | Ofloxacin N-oxide | OFXo | 12.000-75 | 0.995 | 75-1000 | 0.995 | - | | - | |  | | | 8.07±0.8 | | | 0.84±0.1 | | Desmethyl-ofloxacin D8 | | 6.76±0.7 |  | 0.9±0.2 | 0.9±0.1 |
| 52 | 1-(2-nitrobenzylidenamino)-2,4-imidazolidinedione | NPAHD | 0.100-200 | 0.994 | 200-1000 | 0.997 | - | | - | |  | | | 8.11±0.8 | | | 1.06±0.1 | | Chloramphenicol D5 | | 8.60±0.9 |  | 1.9±0.4 | 1.8±0.2 |
| 53 | Cefixime | CFM | 5.000-750 | 0.996 | - | - | - | | - | |  | | | 8.20±0.8 | | | 0.72±0.1 | | Trimethoprim D9 | | 5.91±0.6 |  | 2.7±0.4 | 2.2±0.3 |
| 54 | Gatifloxacin | GAT | 0.010-500 | 0.996 | - | - | - | | - | |  | | | 8.32±0.8 | | | 0.81±0.1 | | Desmethyl-ofloxacin D8 | | 6.76±0.7 |  | 1.5±0.3 | 1.6±0.2 |
| 55 | Chloramphenicol | CHL | 0.500-200 | 0.997 | 200-1000 | 0.998 | - | | - | |  | | | 8.69±0.9 | | | 0.99±0.1 | | Chloramphenicol D5 | | 8.60±0.9 |  | 0.5±0.1 | 0.6±0.1 |
| 56 | N-acetyl sulfamethoxazole | aSMX | 0.063-475 | 0.997 | 475-1500 | 0.997 | - | | - | |  | | | 8.90±0.9 | | | 0.78±0.1 | | Sulfamethoxazole D4 | | 6.97±0.7 |  | 0.8±0.2 | 0.8±0.1 |
| 57 | Linezolid | LZD | 0.100-1000 | 0.994 | - | - | - | | - | |  | | | 8.93±0.9 | | | 0.96±0.1 | | Chloramphenicol D5 | | 8.60±0.9 |  | 1.4±0.3 | 1.4±0.1 |
| 58 | Moxifloxacin | MXF | 1.250-500 | 0.997 | - | - | - | | - | |  | | | 9.14±0.9 | | | 0.74±0.1 | | Desmethyl-ofloxacin D8 | | 6.76±0.7 |  | 2.1±0.4 | 1.8±0.3 |
| 59 | Hydroxy-norfloxacin | hNOR | 1.000-750 | 0.987 | - | - | - | | - | |  | | | 9.82±1.0 | | | 0.69±0.1 | | Desmethyl-ofloxacin D8 | | 6.76±0.7 |  | 0.9±0.2 | 0.9±0.1 |
| 60 | Penicilloic G acid | PenGa | 0.500-750 | 0.997 | - | - | - | | - | |  | | | 9.94±1.0 | | | 0.70±0.1 | | Sulfamethoxazole D4 | | 6.97±0.7 |  | 1.0±0.2 | 1.0±0.1 |
| 61 | Prulifloxacin | PFLX | 1.000-400 | 0.997 | - | - | - | | - | |  | | | 10.18±1.0 | | | 0.66±0.1 | | Desmethyl-ofloxacin D8 | | 6.76±0.7 |  | 1.2±0.2 | 1.1±0.1 |
| 62 | Azithromycin | AZM | 0.050-1000 | 0.999 | - | - | - | | - | |  | | | 10.22±1.0 | | | 1.19±0.1 | | Clarithromycin D3 | | 12.15±1.2 |  | 1.4±0.3 | 1.1±0.1 |
| 63 | N-desmethyl azithromycin | dmAZM | 1.250-400 | 0.991 | - | - | - | | - | |  | | | 10.29±1.0 | | | 1.18±0.1 | | Clarithromycin D3 | | 12.15±1.2 |  | 1.1±0.3 | 1.1±0.1 |
| 64 | Ceftiofur | CTF | 0.500-200 | 0.997 | - | - | - | | - | |  | | | 10.45±1.0 | | | 1.16±0.1 | | Clarithromycin D3 | | 12.15±1.2 |  | 1.9±0.4 | 1.8±0.2 |
| 65 | Deacetyl-ketoconazole | daKTC | 1.250-200 | 0.993 | 200-500 | 0.995 | - | | - | |  | | | 10.49±1.0 | | | 1.09±0.1 | | Flumequine 13C3 | | 11.42±1.1 |  | 1.7±0.5 | 1.7±0.2 |
| 66 | Doxycycline | DOX | 1.250-1000 | 0.991 | - | - | - | | - | |  | | | 10.77±1.1 | | | 1.15±0.1 | | Rifabutin D7 | | 12.48±1.2 |  | 0.8±0.2 | 0.8±0.1 |
| 67 | Clindamycin | CLI | 0.500-1000 | 0.993 | - | - | - | | - | |  | | | 11.09±1.1 | | | 1.03±0.1 | | Flumequine 13C3 | | 11.42±1.1 |  | 63.8±12.8 | 74.5±7.4 |
| 68 | Nalidixic acid | NAL | 0.010-500 | 0.998 | - | - | - | | - | |  | | | 11.14±1.1 | | | 1.03±0.1 | | Flumequine 13C3 | | 11.42±1.1 |  | 1.7±0.2 | 1.7±0.2 |
| 69 | Besifloxacin | BSF | 1.250-750 | 0.988 | - | - | - | | - | |  | | | 11.18±1.1 | | | 1.02±0.1 | | Flumequine 13C3 | | 11.42±1.1 |  | 2.5±0.4 | 2.0±0.3 |
| 70 | N-desmethyl clindamycin | dmCLI | 0.005-200 | 0.994 | - | - | - | | - | |  | | | 11.26±1.1 | | | 1.01±0.1 | | Flumequine 13C3 | | 11.42±1.1 |  | 12.7±1.9 | 11.6±2.3 |
| 71 | Penicillin G | PenG | 0.500-500 | 0.994 | - | - | - | | - | |  | | | 11.32±1.1 | | | 0.61±0.1 | | Sulfamethoxazole D4 | | 6.97±0.7 |  | 1.3±0.5 | 0.9±0.5 |
| 72 | Flumequine | FLU | 0.010-200 | 0.996 | 200-1000 | 0.997 | - | | - | |  | | | 11.45±1.1 | | | 1.00±0.1 | | Flumequine 13C3 | | 11.42±1.1 |  | 2.9±0.4 | 2.8±0.3 |
| 73 | Penicillin V | PenV | 10.00-1000 | 0.992 | - | - | - | | - | |  | | | 11.72±1.2 | | | 0.59±0.1 | | Sulfamethoxazole D4 | | 6.97±0.7 |  | 4.4±0.9 | 4.4±0.7 |
| 74 | Erythromycin | ERY | 0.005-200 | 0.997 | - | - | - | | - | |  | | | 11.74±1.2 | | | 1.03±0.1 | | Clarithromycin D3 | | 12.15±1.2 |  | 3.7±0.6 | 3.8±0.4 |
| 75 | N-demethyl erythromycin | dmERY | 0.034-136 | 0.999 | - | - | - | | - | |  | | | 11.74±1.2 | | | 1.03±0.1 | | Clarithromycin D3 | | 12.15±1.2 |  | 3.6±1.3 | 15.7±3.9 |
| 76 | Ketoconazole | KTC | 0.500-200 | 0.997 | - | - | - | | - | |  | | | 11.81±1.2 | | | 1.00±0.1 | | Ketoconazole D3 | | 12.31±1.2 |  | 5.8±1.2 | 5.8±1.2 |
| 77 | Nadifloxacin | NAD | 1.000-400 | 0.969 | - | - | - | | - | |  | | | 11.86±1.2 | | | 0.96±0.1 | | Flumequine 13C3 | | 11.42±1.1 |  | 1.0±0.1 | 1.0±0.1 |
| 78 | Flucloxacillin | FLX | 0.500-1000 | 0.992 | - | - | - | | - | |  | | | 12.09±1.2 | | | 0.95±0.1 | | Flumequine 13C3 | | 11.42±1.1 |  | 3.5±0.9 | 3.7±0.6 |
| 79 | N-desmethyl clarithromycin | dmCLR | 0.017-1250 | 0.998 | 1250-2000 | 0.981 | - | | - | |  | | | 12.15±1.2 | | | 1.00±0.1 | | Clarithromycin D3 | | 12.15±1.2 |  | 4.4±0.9 | 4.1±0.4 |
| 80 | Clarithromycin | CLR | 0.005-200 | 0.997 | 200-3000 | 0.998 | - | | - | |  | | | 12.15±1.2 | | | 1.00±0.1 | | Clarithromycin D3 | | 12.15±1.2 |  | 8.2±1.6 | 8.1±0.8 |
| 81 | Sulfasalazine | SLZ | 5.000-1000 | 0.997 | - | - | - | | - | |  | | | 12.19±1.2 | | | 0.57±0.1 | | Sulfamethoxazole D4 | | 6.97±0.7 |  | 1.4±0.3 | 1.2±0.1 |
| 82 | Delamanid | DMD | 25.000-750 | 0.997 | - | - | - | | - | |  | | | 12.31±1.2 | | | 0.90±0.1 | | Clarithromycin D3 | | 12.15±1.2 |  | 39.4±11.8 | 38.2±5.7 |
| 83 | 25-O-desacetyl rifabutin | daRFB | 0.100-100 | 0.996 | 100-750 | 0.980 | - | | - | |  | | | 12.34±1.2 | | | 1.01±0.1 | | Rifabutin D7 | | 12.48±1.2 |  | 2.2±0.6 | 2.2±0.3 |
| 84 | 25-desacetyl rifampicin | daRMP | 5.000-500 | 0.937 | - | - | - | | - | |  | | | 12.43±1.2 | | | 1.00±0.1 | | Rifabutin D7 | | 12.48±1.2 |  | 2.0±0.4 | 1.8±0.2 |
| 85 | Rifabutin | RFB | 0.500-400 | 0.996 | - | - | - | | - | |  | | | 12.49±1.2 | | | 1.00±0.1 | | Rifabutin D7 | | 12.48±1.2 |  | 1.4±0.3 | 1.5±0.2 |
| 86 | Rifampicin | RMP | 1.250-1000 | 0.989 | - | - | - | | - | |  | | | 12.57±1.3 | | | 0.99±0.1 | | Rifabutin D7 | | 12.48±1.2 |  | 1.7±0.5 | 1.7±0.3 |
| 87 | Bedaquiline | BDQ | 5.000-400 | 0.778 | - | - | - | | - | |  | | | 13.07±1.3 | | | 0.95±0.1 | | Rifabutin D7 | | 12.48±1.2 |  | #N/A | #N/A |

| **Table S3** Liquid chromatography and mass spectrometry instrumentation setup | |  |
| --- | --- | --- |
| **Instrumentation** |  | |
| *Liquid chromatography* | Waters, ACQUITY UPLC ™ system (Waters, UK) | |
| *Column* | Waters, ACQUITY UPLC BEH C18 (50 × 2.1mm, 1.7μm) | |
| *Mass spectrometry* | Xevo TQD (Waters, UK) | |
| *Source* | Electrospray ionisation (ESI) | |
| *Coordinating LC-MS system* | MassLynx (Waters, UK) | |
|  |  | |
| **Liquid chromatography** |  | |
| *Needle wash (strong)* | 1:1:1:1 MeOH:ACN:IPA:H2O + 0.1 % formic acid | |
| *Needle wash (weak)* | 95:5 H_2_O:MeOH | |
| *Mobile phase A* | 95:5 H_2_O:MeOH with 0.1% formic acid | |
| *Mobile phase B* | MeOH (100%) | |
| *Injection* | 20 µL, 80:20 H_2_O:MeOH, partial-loop | |
| *Inlet gradient elution* | 19 min method:  0-1min (0% B), 1-9.5min (0-40% B), 9.5-13min (40-100% B), 13-16min (100% B), 16-16.5min (100-0% B), 16.5-19min (0% B) | |
| *Flow rate* | 0.2 mL min^-1^ | |
| *Column temperature* | 25°C | |
|  |  | |
| **Mass spectrometry** |  | |
| *Nebulizing gas* | Nitrogen | |
| *Desolvation gas* | N_2_ (1000 L/hr; 400°C) | |
| *Collision gas* | Argon (99.999%) | |
| *Capillary voltage* | 3.8 kV | |
| *Analyses mode* | Positive | |
| *Acquisition mode* | Multiple reaction monitoring (MRM) | |
| *Acquisition rate* | 10 scans sec^-1^ | |
| *Mass resolution ** | 0.75 Da | |
|  |  | |
| **Data processing** |  | |
| *Processing software* | TargetLynx V4.1 (Waters Lab Informatics, UK) | |
|  |  | |
| Triple quadrupole detector (TQD); bridged ethylene hybrid (BEH); ultrahigh performance liquid chromatography (UPLC) | |  |
| (*) Resolution value approximated based on an analyte m/z of 200 | |  |

| **Table S4a** Chromatographic and validation parameters: Matrix retention times for aqueous analyses, ordered by retention time | | | | | | | | |  | | |
| --- | --- | --- | --- | --- | --- | --- | --- | --- | --- | --- | --- |
|  |  |  |  | |  |  | | | |  |  |
|  |  |  | Absolute and relative t_R_  in mobile phase (min) ≠ | | Absolute and relative t_R_  in clean aqueous samples (min) ≠ | | Absolute and relative t_R_  in dirty aqueous samples (min) ≠ | | | |  |
| **#** | **Chemical** | **Abbrev** | **t_R(abs)_** | **t_R(rel)_** | **t_R(abs)_** | **t_R(rel)_** | **t_R(abs)_** | **t_R(rel)_** | | |  |
| 1 | Gentamycin C1 | GEN1 | 0.52±0.1 | 6.30±0.6 | 0.76±0.1 | 4.63±0.5 | 0.68±0.1 | 5.35±1.3 | | |  |
| 2 | Gentamycin C1a | GEN1a | 0.54±0.1 | 6.05±0.6 | 0.74±0.1 | 4.75±0.5 | 0.75±0.1 | 4.78±0.7 | | |  |
| 3 | Gentamycin C2 C2a C2b | GEN2 | 0.54±0.1 | 6.08±0.9 | 0.75±0.1 | 4.67±0.5 | 0.76±0.1 | 4.68±0.5 | | |  |
| 4 | Kanamycin A | KAN | 0.54±0.1 | 6.02±0.6 | 0.72±0.1 | 4.85±0.5 | 0.81±0.1 | 4.33±0.4 | | |  |
| 5 | Capreomycin IA | CAPIa | 0.55±0.1 | 5.97±0.6 | 0.76±0.1 | 4.60±0.5 | 0.82±0.2 | 4.36±0.7 | | |  |
| 6 | Capreomycin IB | CAPIb | 0.56±0.1 | 5.86±0.6 | 0.78±0.1 | 4.54±0.5 | 0.83±0.1 | 4.27±0.6 | | |  |
| 7 | Streptomycin A | STR | 0.57±0.1 | 5.69±0.6 | 0.67±0.1 | 5.25±0.5 | 0.72±0.1 | 4.89±0.5 | | |  |
| 8 | Ethambutol | EMB | 0.65±0.1 | 5.02±0.5 | 0.69±0.1 | 5.07±0.5 | 0.74±0.1 | 4.77±0.5 | | |  |
| 9 | D-cycloserine | DCS | 0.66±0.1 | 4.94±0.5 | 0.68±0.1 | 5.14±0.5 | 0.70±0.1 | 5.02±0.5 | | |  |
| 10 | Imipenem | IPM | 0.72±0.1 | 4.57±0.5 | 0.74±0.1 | 4.75±0.5 | 0.77±0.1 | 4.55±0.5 | | |  |
| 11 | Isoniazid | INH | 0.74±0.1 | 0.98±0.1 | 0.85±0.1 | 1.01±0.1 | 1.53±0.2 | 0.56±0.1 | | |  |
| 12 | Isonicotinic acid | INa | 0.75±0.1 | 0.96±0.1 | 0.82±0.1 | 0.99±0.1 | 0.85±0.1 | 1.01±0.2 | | |  |
| 13 | Acetyl-isoniazid | aINH | 0.79±0.1 | 0.94±0.1 | 0.84±0.1 | 0.97±0.2 | 0.83±0.1 | 1.02±0.2 | | |  |
| 14 | 5-Hydroxy-pyrazinoic acid | hPZA | 0.84±0.2 | 3.98±0.8 | 0.86±0.2 | 4.20±0.6 | 0.81±0.2 | 4.40±0.7 | | |  |
| 15 | Pyrazinamide | PZA | 1.47±0.4 | 2.47±1.0 | 2.05±0.3 | 1.84±0.6 | 1.70±0.2 | 2.05±0.2 | | |  |
| 16 | 2-Amino-1-(4-nitrophenyl)-1,3-propanediol | ANP | 2.05±0.2 | 1.59±0.2 | 2.32±0.2 | 1.51±0.2 | 2.31±0.2 | 1.52±0.2 | | |  |
| 17 | Lamivudine | 3TC | 2.30±0.6 | 1.66±0.3 | 1.95±0.2 | 1.85±0.2 | 1.93±0.2 | 1.80±0.2 | | |  |
| 18 | Hydroxy-metronidazole | hMTZ | 2.32±0.2 | 1.40±0.1 | 2.64±0.3 | 1.33±0.1 | 2.63±0.3 | 1.33±0.1 | | |  |
| 19 | Metronidazole | MTZ | 3.30±0.3 | 0.99±0.1 | 3.55±0.4 | 0.98±0.1 | 3.56±0.4 | 0.98±0.1 | | |  |
| 20 | Amoxicilloic acid | AMXa | 3.50±0.3 | 1.10±0.1 | 3.71±0.4 | 1.11±0.1 | 3.68±0.4 | 1.13±0.1 | | |  |
| 21 | Emtricitabine | FTC | 3.63±0.4 | 0.90±0.1 | 3.84±0.4 | 0.91±0.1 | 3.87±0.4 | 0.91±0.1 | | |  |
| 22 | Sulfadiazine | SDZ | 3.70±0.4 | 1.74±0.2 | 4.03±0.4 | 1.81±0.2 | 4.05±0.4 | 1.79±0.2 | | |  |
| 23 | Amoxicillin | AMX | 3.85±0.4 | 0.99±0.1 | 4.39±0.4 | 1.00±0.1 | 4.11±0.4 | 1.00±0.1 | | |  |
| 24 | Sulfapyridine | SPY | 4.50±0.5 | 1.47±0.1 | 4.75±0.5 | 1.52±0.2 | 4.75±0.5 | 1.52±0.2 | | |  |
| 25 | Meropenem | MEM | 5.24±0.5 | 1.13±0.1 | 5.41±0.5 | 1.13±0.1 | 5.54±0.6 | 1.12±0.1 | | |  |
| 26 | N-acetyl sulfadiazine | aSDZ | 5.54±0.6 | 1.26±0.1 | 5.86±0.6 | 1.24±0.1 | 5.89±0.6 | 1.23±0.1 | | |  |
| 27 | Nitrofurantoin | NIT | 5.54±0.6 | 1.00±0.1 | 5.96±0.6 | 1.00±0.1 | 5.70±0.6 | 1.01±0.1 | | |  |
| 28 | Aztreonam | ATM | 5.85±0.6 | 1.01±0.1 | 6.03±0.6 | 1.01±0.1 | 6.00±0.6 | 1.02±0.1 | | |  |
| 29 | Ampicilloic acid | AMPa | 6.01±0.6 | 1.24±0.1 | 6.30±0.6 | 1.20±0.1 | 6.28±0.6 | 1.20±0.1 | | |  |
| 30 | Trimethoprim | TMP | 6.01±0.6 | 0.98±0.1 | 6.24±0.6 | 0.98±0.1 | 6.23±0.6 | 0.75±0.1 | | |  |
| 31 | 4-hydroxy-trimethoprim | hTMP | 6.15±0.6 | 0.96±0.1 | 6.39±0.6 | 0.96±0.1 | 6.37±0.6 | 0.96±0.1 | | |  |
| 32 | N-acetyl sulfapyridine | aSPY | 6.19±0.6 | 1.13±0.1 | 6.40±0.6 | 1.13±0.1 | 6.38±0.6 | 1.13±0.1 | | |  |
| 33 | Tetracycline | TET | 6.55±0.7 | 1.64±0.3 | 6.79±0.7 | 1.62±0.2 | 6.79±0.7 | 1.62±0.2 | | |  |
| 34 | Ofloxacin (Levofloxacin) | OFX | 6.59±0.7 | 1.00±0.1 | 6.79±0.7 | 1.00±0.1 | 6.79±0.7 | 1.00±0.1 | | |  |
| 35 | Desethylene ciprofloxacin | deCIP | 6.71±0.7 | 1.01±0.1 | 6.93±0.7 | 1.03±0.1 | 6.93±0.7 | 1.03±0.1 | | |  |
| 36 | Oxytetracycline | OTC | 6.77±0.7 | 1.02±0.1 | 7.03±0.7 | 1.02±0.1 | 7.10±0.7 | 1.01±0.1 | | |  |
| 37 | Desmethyl-ofloxacin | dmOFX | 6.82±0.7 | 0.99±0.1 | 7.02±0.7 | 0.99±0.1 | 7.02±0.7 | 0.99±0.1 | | |  |
| 38 | Ceftriaxone | CRO | 6.82±0.7 | 1.01±0.1 | 6.83±0.7 | 1.04±0.1 | 6.80±0.7 | 1.04±0.1 | | |  |
| 39 | Cefalexin | LEX | 6.92±0.7 | 0.99±0.1 | 7.15±0.7 | 0.99±0.1 | 7.14±0.7 | 0.99±0.1 | | |  |
| 40 | Florfenicol | FLO | 6.92±0.7 | 1.24±0.1 | 7.20±0.7 | 1.24±0.1 | 7.18±0.7 | 1.24±0.1 | | |  |
| 41 | Norfloxacin | NOR | 7.00±0.7 | 0.97±0.1 | 7.11±0.7 | 0.98±0.1 | 7.11±0.7 | 0.98±0.1 | | |  |
| 42 | Sulfamethoxazole | SMX | 7.02±0.7 | 0.99±0.1 | 7.26±0.7 | 0.99±0.1 | 7.24±0.7 | 0.99±0.1 | | |  |
| 43 | Ciprofloxacin | CIP | 7.14±0.7 | 0.95±0.1 | 7.31±0.7 | 0.98±0.1 | 7.32±0.7 | 0.98±0.1 | | |  |
| 44 | Enrofloxacin | ENR | 7.20±0.7 | 0.94±0.1 | 7.42±0.7 | 0.96±0.1 | 7.42±0.7 | 0.96±0.1 | | |  |
| 45 | Danofloxacin | DFX | 7.34±0.7 | 0.92±0.1 | 7.65±0.8 | 0.93±0.1 | 7.55±0.8 | 0.92±0.1 | | |  |
| 46 | Lomefloxacin | LOM | 7.44±0.7 | 0.91±0.1 | 7.61±0.8 | 0.94±0.1 | 7.60±0.8 | 0.94±0.1 | | |  |
| 47 | Ampicillin | AMP | 7.49±0.7 | 0.99±0.1 | 7.76±0.8 | 0.98±0.1 | 7.75±0.8 | 0.97±0.1 | | |  |
| 48 | Thalidomide | THAL | 7.87±0.8 | 0.75±0.1 | 8.11±0.8 | 0.75±0.1 | 8.07±0.8 | 0.88±0.1 | | |  |
| 49 | Ulifloxacin | UFX | 7.90±0.8 | 0.86±0.1 | 8.13±0.8 | 0.88±0.1 | 8.18±0.8 | 0.87±0.1 | | |  |
| 50 | Sarafloxacin | SRF | 7.92±0.8 | 0.85±0.1 | 8.11±0.8 | 0.88±0.1 | 8.14±0.8 | 0.88±0.1 | | |  |
| 51 | Ofloxacin N-oxide | OFXo | 8.07±0.8 | 0.84±0.1 | 8.27±0.8 | 0.84±0.1 | 8.27±0.8 | 0.84±0.1 | | |  |
| 52 | 1-(2-nitrobenzylidenamino)-2,4-imidazolidinedione | NPAHD | 8.11±0.8 | 1.06±0.1 | 8.38±0.8 | 1.07±0.1 | 8.51±0.9 | 1.05±0.1 | | |  |
| 53 | Cefixime | CFM | 8.20±0.8 | 0.72±0.1 | 8.36±0.8 | 0.73±0.1 | 8.28±0.8 | 0.80±0.1 | | |  |
| 54 | Gatifloxacin | GAT | 8.32±0.8 | 0.81±0.1 | 8.49±0.8 | 0.84±0.1 | 8.51±0.9 | 0.84±0.1 | | |  |
| 55 | Chloramphenicol | CHL | 8.69±0.9 | 0.99±0.1 | 9.04±0.9 | 0.99±0.1 | 9.03±0.9 | 0.99±0.1 | | |  |
| 56 | N-acetyl sulfamethoxazole | aSMX | 8.90±0.9 | 0.78±0.1 | 9.13±0.9 | 0.79±0.1 | 9.09±0.9 | 0.79±0.1 | | |  |
| 57 | Linezolid | LZD | 8.93±0.9 | 0.96±0.1 | 9.19±0.9 | 0.97±0.1 | 9.14±0.9 | 0.98±0.1 | | |  |
| 58 | Moxifloxacin | MXF | 9.14±0.9 | 0.74±0.1 | 9.36±0.9 | 0.76±0.1 | 9.41±0.9 | 0.74±0.1 | | |  |
| 59 | Hydroxy-norfloxacin | hNOR | 9.82±1.0 | 0.69±0.1 | 10.07±1.0 | 0.71±0.1 | 10.05±1.0 | 0.71±0.1 | | |  |
| 60 | Penicilloic G acid | PenGa | 9.94±1.0 | 1.14±0.1 | 10.15±1.0 | 1.13±0.1 | 10.07±1.0 | 1.13±0.1 | | |  |
| 61 | Prulifloxacin | PFLX | 10.18±1.0 | 0.66±0.1 | 10.58±1.1 | 0.67±0.1 | 10.49±1.0 | 0.67±0.1 | | |  |
| 62 | Azithromycin | AZM | 10.22±1.0 | 1.15±0.1 | 10.51±1.1 | 1.06±0.1 | 10.58±1.1 | 1.07±0.1 | | |  |
| 63 | N-desmethyl azithromycin | dmAZM | 10.29±1.0 | 1.14±0.1 | 10.57±1.1 | 1.05±0.1 | 10.65±1.1 | 1.10±0.1 | | |  |
| 64 | Ceftiofur | CTF | 10.45±1.0 | 0.93±0.1 | 10.64±1.1 | 1.07±0.1 | 10.62±1.1 | 1.09±0.1 | | |  |
| 65 | Deacetyl-ketoconazole | daKTC | 10.49±1.0 | 1.12±0.1 | 10.91±1.1 | 1.06±0.1 | 10.88±1.1 | 1.06±0.1 | | |  |
| 66 | Doxycycline | DOX | 10.77±1.1 | 1.00±0.1 | 10.98±1.1 | 1.00±0.1 | 11.06±1.1 | 1.00±0.1 | | |  |
| 67 | Clindamycin | CLI | 11.09±1.1 | 1.06±0.1 | 11.32±1.1 | 1.05±0.1 | 11.33±1.1 | 1.06±0.1 | | |  |
| 68 | Nalidixic acid | NAL | 11.14±1.1 | 1.03±0.1 | 11.27±1.1 | 1.03±0.1 | 11.24±1.1 | 1.03±0.1 | | |  |
| 69 | Besifloxacin | BSF | 11.18±1.1 | 1.02±0.1 | 11.32±1.1 | 1.02±0.1 | 11.43±1.1 | 1.01±0.1 | | |  |
| 70 | N-desmethyl clindamycin | dmCLI | 11.26±1.1 | 1.04±0.1 | 11.41±1.1 | 1.04±0.1 | 11.50±1.1 | 1.04±0.1 | | |  |
| 71 | Penicillin G | PenG | 11.32±1.1 | 1.00±0.1 | 11.47±1.1 | 1.00±0.1 | 11.44±1.1 | 1.00±0.1 | | |  |
| *Table S4a continued* | | | | | | | | | | |  |
| **#** | **Chemical** | **Abbrev** | **mP tR(abs)** | **mP tR(rel)** | **Clean tR(abs)** | **Clean tR(rel)** | **Dirty tR(abs)** | **Dirty tR(rel)** | | |  |
| 72 | Flumequine | FLU | 11.45±1.1 | 1.00±0.1 | 11.55±1.2 | 1.00±0.1 | 11.55±1.2 | 1.00±0.1 | | |  |
| 73 | Penicillin V | PenV | 11.72±1.2 | 0.96±0.1 | 11.88±1.2 | 0.97±0.1 | 11.86±1.2 | 0.97±0.1 | | |  |
| 74 | Erythromycin | ERY | 11.74±1.2 | 1.00±0.1 | 11.87±1.2 | 1.00±0.1 | 12.01±1.2 | 1.00±0.1 | | |  |
| 75 | N-demethyl erythromycin | dmERY | 11.74±1.2 | 1.00±0.1 | 11.86±1.2 | 1.00±0.1 | 12.02±1.2 | 1.00±0.1 | | |  |
| 76 | Ketoconazole | KTC | 11.81±1.2 | 1.00±0.1 | 11.94±1.2 | 0.97±0.2 | 12.11±1.2 | 1.00±0.1 | | |  |
| 77 | Nadifloxacin | NAD | 11.86±1.2 | 0.96±0.1 | 11.99±1.2 | 0.96±0.1 | 11.95±1.2 | 0.97±0.1 | | |  |
| 78 | Flucloxacillin | FLX | 12.09±1.2 | 0.95±0.1 | 12.18±1.2 | 0.95±0.1 | 11.98±1.2 | 0.96±0.1 | | |  |
| 79 | N-desmethyl clarithromycin | dmCLR | 12.15±1.2 | 1.00±0.1 | 12.27±1.2 | 1.00±0.1 | 12.50±1.3 | 1.00±0.1 | | |  |
| 80 | Clarithromycin | CLR | 12.15±1.2 | 1.00±0.1 | 12.27±1.2 | 1.00±0.1 | 12.50±1.2 | 1.00±0.1 | | |  |
| 81 | Sulfasalazine | SLZ | 12.19±1.2 | 1.00±0.1 | 12.30±1.2 | 1.00±0.1 | 12.27±1.2 | 1.00±0.1 | | |  |
| 82 | Delamanid | DMD | 12.31±1.2 | 1.01±0.1 | 12.44±1.2 | 1.02±0.1 | 12.52±1.3 | 1.03±0.1 | | |  |
| 83 | 25-O-desacetyl rifabutin | daRFB | 12.34±1.2 | 1.01±0.1 | 12.47±1.2 | 1.01±0.1 | 12.72±1.3 | 1.01±0.1 | | |  |
| 84 | 25-desacetyl rifampicin | daRMP | 12.43±1.2 | 1.00±0.1 | 12.67±1.3 | 1.00±0.1 | 12.86±1.3 | 1.00±0.1 | | |  |
| 85 | Rifabutin | RFB | 12.49±1.2 | 1.00±0.1 | 12.62±1.3 | 1.00±0.1 | 12.87±1.3 | 1.00±0.1 | | |  |
| 86 | Rifampicin | RMP | 12.57±1.3 | 0.99±0.1 | 12.72±1.3 | 0.99±0.1 | 12.93±1.3 | 1.00±0.1 | | |  |
| 87 | Bedaquiline | BDQ | 13.07±1.3 | 0.95±0.1 | 13.18±1.3 | 0.96±0.1 | 13.65±1.4 | 0.97±0.1 | | |  |

| **Table S4b** Chromatographic and validation parameters: Matrix retention times for solids analyses, ordered by retention time | | | | | | | | |  | | |
| --- | --- | --- | --- | --- | --- | --- | --- | --- | --- | --- | --- |
|  |  |  |  | |  |  | | | |  |  |
|  |  |  | Absolute and relative t_R_  in mobile phase (min) ≠ | | Absolute and relative t_R_  in influent SPM (min) ≠ | | Absolute and relative t_R_  in river sediment samples (min) ≠ | | | |  |
| **#** | **Chemical** | **Abbrev** | **t_R(abs)_** | **t_R(rel)_** | **t_R(abs)_** | **t_R(rel)_** | **t_R(abs)_** | **t_R(rel)_** | | |  |
| 1 | Gentamycin C1 | GEN1 | 0.52±0.1 | 6.30±0.6 | 0.89±0.1 | 3.74±0.4 | 0.89±0.1 | 3.74±0.4 | | |  |
| 2 | Gentamycin C1a | GEN1a | 0.54±0.1 | 6.05±0.6 | 0.77±0.2 | 4.17±1.0 | 0.77±0.2 | 4.17±1.0 | | |  |
| 3 | Gentamycin C2 C2a C2b | GEN2 | 0.54±0.1 | 6.08±0.9 | 0.77±0.2 | 4.52±1.1 | 0.77±0.2 | 4.52±1.1 | | |  |
| 4 | Kanamycin A | KAN | 0.54±0.1 | 6.02±0.6 | 0.85±0.1 | 3.93±0.6 | 0.85±0.1 | 3.93±0.6 | | |  |
| 5 | Capreomycin IA | CAPIa | 0.55±0.1 | 5.97±0.6 | 0.81±0.1 | 4.07±0.4 | 0.81±0.1 | 4.07±0.4 | | |  |
| 6 | Capreomycin IB | CAPIb | 0.56±0.1 | 5.86±0.6 | 0.80±0.1 | 4.14±0.4 | 0.80±0.1 | 4.14±0.4 | | |  |
| 7 | Streptomycin A | STR | 0.57±0.1 | 5.69±0.6 | 0.77±0.1 | 4.32±0.4 | 0.77±0.1 | 4.32±0.4 | | |  |
| 8 | Ethambutol | EMB | 0.65±0.1 | 5.02±0.5 | 0.78±0.1 | 4.22±0.4 | 0.78±0.2 | 4.31±1.3 | | |  |
| 9 | D-cycloserine | DCS | 0.66±0.1 | 4.94±0.5 | 0.74±0.1 | 4.44±0.4 | 0.74±0.1 | 4.44±0.4 | | |  |
| 10 | Imipenem | IPM | 0.72±0.1 | 4.57±0.5 | 2.04±0.2 | 1.62±0.2 | 2.04±0.2 | 1.62±0.2 | | |  |
| 11 | Isoniazid | INH | 0.74±0.1 | 4.33±0.4 | 1.32±0.1 | 2.49±0.2 | 1.32±0.1 | 2.49±0.2 | | |  |
| 12 | Isonicotinic acid | INa | 0.75±0.1 | 4.48±0.4 | 0.97±0.1 | 3.35±0.3 | 0.97±0.1 | 3.35±0.3 | | |  |
| 13 | Acetyl-isoniazid | aINH | 0.79±0.1 | 4.12±0.4 | 0.88±0.1 | 3.75±0.4 | 0.88±0.1 | 3.75±0.4 | | |  |
| 14 | 5-Hydroxy-pyrazinoic acid | hPZA | 0.84±0.2 | 3.98±0.8 | 0.92±0.2 | 3.68±0.7 | 1.38±0.1 | 2.34±0.2 | | |  |
| 15 | Pyrazinamide | PZA | 1.47±0.4 | 2.47±1.0 | 1.12±0.2 | 3.02±0.5 | 1.21±0.4 | 2.81±0.7 | | |  |
| 16 | 2-Amino-1-(4-nitrophenyl)-1,3-propanediol | ANP | 2.05±0.2 | 1.59±0.2 | 2.00±0.2 | 1.64±0.2 | 2.00±0.2 | 1.64±0.2 | | |  |
| 17 | Lamivudine | 3TC | 2.30±0.6 | 1.66±0.3 | 1.55±0.2 | 2.08±0.2 | 1.55±0.2 | 2.08±0.2 | | |  |
| 18 | Hydroxy-metronidazole | hMTZ | 2.32±0.2 | 1.40±0.1 | 2.22±0.2 | 1.47±0.1 | 2.22±0.2 | 1.47±0.1 | | |  |
| 19 | Metronidazole | MTZ | 3.30±0.3 | 0.99±0.1 | 3.32±0.3 | 0.98±0.1 | 3.32±0.3 | 0.98±0.1 | | |  |
| 20 | Amoxicilloic acid | AMXa | 3.50±0.3 | 2.00±0.2 | #N/A | #N/A | #N/A | #N/A | | |  |
| 21 | Emtricitabine | FTC | 3.63±0.4 | 0.90±0.1 | 3.61±0.4 | 0.90±0.1 | 3.61±0.4 | 0.90±0.1 | | |  |
| 22 | Sulfadiazine | SDZ | 3.70±0.4 | 1.74±0.2 | 3.99±0.6 | 1.77±0.2 | 3.85±0.4 | 1.80±0.2 | | |  |
| 23 | Amoxicillin | AMX | 3.85±0.4 | 1.81±0.2 | 6.44±0.6 | #N/A | 6.44±0.6 | #N/A | | |  |
| 24 | Sulfapyridine | SPY | 4.50±0.5 | 1.47±0.1 | 4.53±0.5 | 1.53±0.2 | 4.53±0.5 | 1.53±0.2 | | |  |
| 25 | Meropenem | MEM | 5.24±0.5 | 1.13±0.1 | 5.79±0.6 | 1.01±0.1 | 5.79±0.6 | 1.01±0.1 | | |  |
| 26 | N-acetyl sulfadiazine | aSDZ | 5.54±0.6 | 1.26±0.1 | 5.49±0.5 | 1.26±0.1 | 5.49±0.5 | 1.26±0.1 | | |  |
| 27 | Nitrofurantoin | NIT | 5.54±0.6 | 1.00±0.1 | 5.47±0.5 | 1.00±0.1 | 5.47±0.5 | 1.00±0.1 | | |  |
| 28 | Aztreonam | ATM | 5.85±0.6 | 1.01±0.1 | 6.61±0.7 | 0.88±0.1 | 6.61±0.7 | 0.88±0.1 | | |  |
| 29 | Ampicilloic acid | AMPa | 6.01±0.6 | 1.16±0.1 | 7.27±0.7 | 1.02±0.1 | 7.27±0.7 | 1.02±0.1 | | |  |
| 30 | Trimethoprim | TMP | 6.01±0.6 | 0.98±0.1 | 5.94±0.6 | 0.98±0.1 | 5.94±0.6 | 0.98±0.1 | | |  |
| 31 | 4-hydroxy-trimethoprim | hTMP | 6.15±0.6 | 0.96±0.1 | 6.12±0.6 | 0.95±0.1 | 6.12±0.6 | 0.95±0.1 | | |  |
| 32 | N-acetyl sulfapyridine | aSPY | 6.19±0.6 | 1.13±0.1 | 6.17±0.6 | 1.13±0.1 | 6.17±0.6 | 1.13±0.1 | | |  |
| 33 | Tetracycline | TET | 6.55±0.7 | 1.03±0.1 | 6.61±0.7 | 1.01±0.1 | 6.61±0.7 | 1.01±0.1 | | |  |
| 34 | Ofloxacin (Levofloxacin) | OFX | 6.59±0.7 | 1.00±0.1 | 6.46±0.6 | 1.00±0.1 | 6.46±0.6 | 1.00±0.1 | | |  |
| 35 | Desethylene ciprofloxacin | deCIP | 6.71±0.7 | 1.01±0.1 | 6.57±0.7 | 1.01±0.1 | 6.57±0.7 | 1.01±0.1 | | |  |
| 36 | Oxytetracycline | OTC | 6.77±0.7 | 1.00±0.1 | 6.89±0.7 | 0.97±0.1 | 6.89±0.7 | 0.97±0.1 | | |  |
| 37 | Desmethyl-ofloxacin | dmOFX | 6.82±0.7 | 0.99±0.1 | 6.68±0.7 | 0.99±0.1 | 6.68±0.7 | 0.99±0.1 | | |  |
| 38 | Ceftriaxone | CRO | 6.82±0.7 | 0.86±0.1 | #N/A | #N/A | #N/A | #N/A | | |  |
| 39 | Cefalexin | LEX | 6.92±0.7 | 0.85±0.1 | #N/A | #N/A | #N/A | #N/A | | |  |
| 40 | Florfenicol | FLO | 6.92±0.7 | 1.24±0.1 | 6.94±0.7 | 1.26±0.1 | 6.94±0.7 | 1.26±0.1 | | |  |
| 41 | Norfloxacin | NOR | 7.00±0.7 | 0.97±0.1 | 6.76±0.7 | 0.98±0.1 | 6.76±0.7 | 0.98±0.1 | | |  |
| 42 | Sulfamethoxazole | SMX | 7.02±0.7 | 0.99±0.1 | 6.98±0.7 | 0.99±0.1 | 6.98±0.7 | 0.99±0.1 | | |  |
| 43 | Ciprofloxacin | CIP | 7.14±0.7 | 0.95±0.1 | 6.97±0.7 | 0.95±0.1 | 6.97±0.7 | 0.95±0.1 | | |  |
| 44 | Enrofloxacin | ENR | 7.20±0.7 | 0.94±0.1 | 7.07±0.7 | 0.94±0.1 | 7.07±0.7 | 0.94±0.1 | | |  |
| 45 | Danofloxacin | DFX | 7.34±0.7 | 0.92±0.1 | 7.19±0.7 | 0.92±0.1 | 7.19±0.7 | 0.92±0.1 | | |  |
| 46 | Lomefloxacin | LOM | 7.44±0.7 | 0.91±0.1 | 7.28±0.7 | 0.91±0.1 | 7.28±0.7 | 0.91±0.1 | | |  |
| 47 | Ampicillin | AMP | 7.49±0.7 | 0.93±0.1 | 7.45±0.7 | 0.93±0.1 | 7.45±0.7 | 0.93±0.1 | | |  |
| 48 | Thalidomide | THAL | 7.87±0.8 | 0.75±0.1 | 7.81±0.8 | 0.75±0.1 | 7.81±0.8 | 0.75±0.1 | | |  |
| 49 | Ulifloxacin | UFX | 7.90±0.8 | 0.86±0.1 | 7.76±0.8 | 0.86±0.1 | 7.76±0.8 | 0.86±0.1 | | |  |
| 50 | Sarafloxacin | SRF | 7.92±0.8 | 0.85±0.1 | 7.78±0.8 | 0.85±0.1 | 7.78±0.8 | 0.85±0.1 | | |  |
| 51 | Ofloxacin N-oxide | OFXo | 8.07±0.8 | 0.84±0.1 | 7.92±0.8 | 0.84±0.1 | 7.92±0.8 | 0.84±0.1 | | |  |
| 52 | 1-(2-nitrobenzylidenamino)-2,4-imidazolidinedione | NPAHD | 8.11±0.8 | 1.06±0.1 | 8.05±0.8 | 1.09±0.1 | 8.05±0.8 | 1.09±0.1 | | |  |
| 53 | Cefixime | CFM | 8.20±0.8 | 0.72±0.1 | 8.07±0.8 | 0.72±0.1 | 8.07±0.8 | 0.72±0.1 | | |  |
| 54 | Gatifloxacin | GAT | 8.32±0.8 | 0.81±0.1 | 8.16±0.8 | 0.82±0.1 | 8.16±0.8 | 0.82±0.1 | | |  |
| 55 | Chloramphenicol | CHL | 8.69±0.9 | 0.99±0.1 | 8.74±0.9 | 1.00±0.1 | 8.74±0.9 | 1.00±0.1 | | |  |
| 56 | N-acetyl sulfamethoxazole | aSMX | 8.90±0.9 | 0.78±0.1 | 8.85±0.9 | 0.78±0.1 | 8.85±0.9 | 0.78±0.1 | | |  |
| 57 | Linezolid | LZD | 8.93±0.9 | 0.96±0.1 | 8.90±0.9 | 0.98±0.1 | 8.90±0.9 | 0.98±0.1 | | |  |
| 58 | Moxifloxacin | MXF | 9.14±0.9 | 0.74±0.1 | 9.00±0.9 | 0.74±0.1 | 9.00±0.9 | 0.74±0.1 | | |  |
| 59 | Hydroxy-norfloxacin | hNOR | 9.82±1.0 | 0.69±0.1 | 9.71±1.0 | 0.70±0.1 | 9.71±1.0 | 0.70±0.1 | | |  |
| 60 | Penicilloic G acid | PenGa | 9.94±1.0 | 0.70±0.1 | 10.36±1.0 | 1.08±0.1 | 10.36±1.0 | 1.08±0.1 | | |  |
| 61 | Prulifloxacin | PFLX | 10.18±1.0 | 0.66±0.1 | 10.45±1.0 | 0.67±0.1 | 10.45±1.0 | 0.67±0.1 | | |  |
| 62 | Azithromycin | AZM | 10.22±1.0 | 1.19±0.1 | 10.18±1.0 | 1.20±0.1 | 10.18±1.0 | 1.20±0.1 | | |  |
| 63 | N-desmethyl azithromycin | dmAZM | 10.29±1.0 | 1.18±0.1 | 10.80±1.1 | 1.13±0.1 | 10.80±1.1 | 1.13±0.1 | | |  |
| 64 | Ceftiofur | CTF | 10.45±1.0 | 1.16±0.1 | 10.21±1.0 | 1.20±0.1 | 10.21±1.0 | 1.20±0.1 | | |  |
| 65 | Deacetyl-ketoconazole | daKTC | 10.49±1.0 | 1.09±0.1 | 10.70±1.1 | 1.07±0.1 | 10.70±1.1 | 1.07±0.1 | | |  |
| 66 | Doxycycline | DOX | 10.77±1.1 | 1.15±0.1 | 11.11±1.1 | 1.13±0.1 | 11.11±1.1 | 1.13±0.1 | | |  |
| 67 | Clindamycin | CLI | 11.09±1.1 | 1.03±0.1 | 11.07±1.1 | 1.03±0.1 | 11.07±1.1 | 1.03±0.1 | | |  |
| 68 | Nalidixic acid | NAL | 11.14±1.1 | 1.03±0.1 | 11.08±1.1 | 1.03±0.1 | 11.08±1.1 | 1.03±0.1 | | |  |
| 69 | Besifloxacin | BSF | 11.18±1.1 | 1.02±0.1 | 11.43±1.1 | 1.00±0.1 | 11.43±1.1 | 1.00±0.1 | | |  |
| 70 | N-desmethyl clindamycin | dmCLI | 11.26±1.1 | 1.01±0.1 | 11.27±1.1 | 1.01±0.1 | 11.27±1.1 | 1.01±0.1 | | |  |
| 71 | Penicillin G | PenG | 11.32±1.1 | 0.61±0.1 | 11.44±1.1 | 0.61±0.1 | 11.44±1.1 | 0.61±0.1 | | |  |
| *Table S4b continued* | | | | | | | | | | |  |
| **#** | **Chemical** | **Abbrev** | **mP tR(abs)** | **mP tR(rel)** | **Clean tR(abs)** | **Clean tR(rel)** | **Dirty tR(abs)** | **Dirty tR(rel)** | | |  |
| 72 | Flumequine | FLU | 11.45±1.1 | 1.00±0.1 | 11.41±1.1 | 1.00±0.1 | 11.41±1.1 | 1.00±0.1 | | |  |
| 73 | Penicillin V | PenV | 11.72±1.2 | 0.59±0.1 | 12.03±1.2 | 0.57±0.1 | 12.03±1.2 | 0.57±0.1 | | |  |
| 74 | Erythromycin | ERY | 11.74±1.2 | 1.03±0.1 | 11.67±1.2 | 1.05±0.1 | 11.67±1.2 | 1.05±0.1 | | |  |
| 75 | N-demethyl erythromycin | dmERY | 11.74±1.2 | 1.03±0.1 | 11.78±1.2 | 1.04±0.1 | 11.78±1.2 | 1.04±0.1 | | |  |
| 76 | Ketoconazole | KTC | 11.81±1.2 | 1.00±0.1 | 11.85±1.2 | 1.00±0.1 | 11.85±1.2 | 1.00±0.1 | | |  |
| 77 | Nadifloxacin | NAD | 11.86±1.2 | 0.96±0.1 | 11.90±1.2 | 0.96±0.1 | 11.90±1.2 | 0.96±0.1 | | |  |
| 78 | Flucloxacillin | FLX | 12.09±1.2 | 0.95±0.1 | 12.15±1.2 | 0.94±0.1 | 12.15±1.2 | 0.94±0.1 | | |  |
| 79 | N-desmethyl clarithromycin | dmCLR | 12.15±1.2 | 1.00±0.1 | 12.23±1.2 | 1.00±0.1 | 12.23±1.2 | 1.00±0.1 | | |  |
| 80 | Clarithromycin | CLR | 12.15±1.2 | 1.00±0.1 | 12.23±1.2 | 1.00±0.1 | 12.23±1.2 | 1.00±0.1 | | |  |
| 81 | Sulfasalazine | SLZ | 12.19±1.2 | 0.57±0.1 | 12.23±1.2 | 0.57±0.1 | 12.23±1.2 | 0.57±0.1 | | |  |
| 82 | Delamanid | DMD | 12.31±1.2 | 0.90±0.1 | 12.47±1.2 | 0.98±0.1 | 12.47±1.2 | 0.98±0.1 | | |  |
| 83 | 25-O-desacetyl rifabutin | daRFB | 12.34±1.2 | 1.01±0.1 | 12.42±1.2 | 1.01±0.1 | 12.42±1.2 | 1.01±0.1 | | |  |
| 84 | 25-desacetyl rifampicin | daRMP | 12.43±1.2 | 1.00±0.1 | 12.63±1.3 | 1.00±0.1 | 12.63±1.3 | 1.00±0.1 | | |  |
| 85 | Rifabutin | RFB | 12.49±1.2 | 1.00±0.1 | 12.57±1.3 | 1.00±0.1 | 12.57±1.3 | 1.00±0.1 | | |  |
| 86 | Rifampicin | RMP | 12.57±1.3 | 0.99±0.1 | 12.62±1.3 | 1.00±0.1 | 12.62±1.3 | 1.00±0.1 | | |  |
| 87 | Bedaquiline | BDQ | 13.07±1.3 | 0.95±0.1 | 13.21±1.3 | 0.95±0.1 | 13.21±1.3 | 0.95±0.1 | | |  |

| **Table S5a** Chromatographic and validation parameters: Ion ratios for aqueous analyses, ordered by retention time | | | | | | | | |
| --- | --- | --- | --- | --- | --- | --- | --- | --- |
|  |  |  | Ion Ratio (PI_1_/PI_2_) in mobile phase | | Ion Ratio (PI_1_/PI_2_) in clean samples | | Ion Ratio (PI_1_/PI_2_) in dirty samples | |
| **#** | **Chemical** | **Abbrev** | **0-100 µg L^-1^** | **100-1000 µg L^-1^** | **0-100 µg L^-1^** | **100-1000 µg L^-1^** | **0-100 µg L^-1^** | **100-1000 µg L^-1^** |
| 1 | Gentamycin C1 | GEN1 | #N/A | #N/A | #N/A | #N/A | #N/A | #N/A |
| 2 | Gentamycin C1a | GEN1a | #N/A | #N/A | #N/A | #N/A | #N/A | #N/A |
| 3 | Gentamycin C2 C2a C2b | GEN2 | #N/A | #N/A | #N/A | #N/A | #N/A | #N/A |
| 4 | Kanamycin A | KAN | #N/A | #N/A | #N/A | #N/A | #N/A | #N/A |
| 5 | Capreomycin IA | CAPIa | #N/A | #N/A | #N/A | #N/A | #N/A | #N/A |
| 6 | Capreomycin IB | CAPIb | #N/A | #N/A | #N/A | #N/A | #N/A | #N/A |
| 7 | Streptomycin A | STR | #N/A | #N/A | #N/A | #N/A | #N/A | #N/A |
| 8 | Ethambutol | EMB | #N/A | #N/A | #N/A | #N/A | #N/A | #N/A |
| 9 | D-cycloserine | DCS | 0.32±0.05 | 0.30±0.04 | #N/A | #N/A | #N/A | #N/A |
| 10 | Imipenem | IPM | 1.2±0.2 | 1.0±0.1 | 1.2±0.4 | 1.2±0.4 | 1.4±0.5 | 1.0±0.4 |
| 11 | Isoniazid | INH | 7.9±0.8 | 8.8±0.9 | 4.2±1.0 | 6.0±0.6 | 8.2±1.2 | 8.0±0.8 |
| 12 | Isonicotinic acid | INa | 3.9±0.6 | 3.8±0.4 | 4.5±1.8 | 3.7±1.1 | 3.8±1.0 | 3.7±0.9 |
| 13 | Acetyl-isoniazid | aINH | 0.5±0.1 | 0.52±0.06 | 0.4±0.1 | 0.47±0.05 | 0.26±0.04 | 0.4±0.1 |
| 14 | 5-Hydroxy-pyrazinoic acid | hPZA | 0.15±0.05 | 0.07±0.02 | 0.12±0.04 | 0.10±0.03 | 0.16±0.04 | 0.13±0.02 |
| 15 | Pyrazinamide | PZA | 1.0±0.1 | 0.77±0.08 | 0.7±0.1 | 0.4±0.1 | 0.26±0.06 | 0.29±0.03 |
| 16 | 2-Amino-1-(4-nitrophenyl)-1,3-propanediol | ANP | 2.4±0.4 | 2.4±0.2 | 2.3±0.3 | 2.4±0.2 | 2.3±0.3 | 2.3±0.2 |
| 17 | Lamivudine | 3TC | 6.3±0.9 | 7.4±0.7 | 3.8±0.6 | 4.0±0.4 | 4.1±0.6 | 4.5±0.4 |
| 18 | Hydroxy-metronidazole | hMTZ | 1.4±0.2 | 1.3±0.1 | 1.3±0.1 | 1.3±0.1 | 1.3±0.1 | 1.3±0.1 |
| 19 | Metronidazole | MTZ | 1.8±0.3 | 1.8±0.2 | 1.8±0.2 | 1.8±0.2 | 1.8±0.2 | 1.8±0.2 |
| 20 | Amoxicilloic acid | AMXa | 1.3±0.3 | 1.3±0.1 | 1.1±0.2 | 1.2±0.3 | 1.0±0.3 | 1.1±0.3 |
| 21 | Emtricitabine | FTC | 12.4±2.5 | 13.2±1.3 | 10.8±3.8 | 12.5±1.9 | 12.0±3.0 | 12.4±1.2 |
| 22 | Sulfadiazine | SDZ | 0.9±0.1 | 0.9±0.1 | 1.0±0.2 | 0.9±0.1 | 0.9±0.1 | 0.89±0.09 |
| 23 | Amoxicillin | AMX | 3.3±0.7 | 2.9±0.3 | 4.9±1.5 | 4.6±0.7 | 3.9±1.0 | 3.2±0.8 |
| 24 | Sulfapyridine | SPY | 1.2±0.1 | 1.2±0.1 | 1.2±0.1 | 1.2±0.1 | 1.2±0.1 | 1.2±0.1 |
| 25 | Meropenem | MEM | 6.3±0.9 | 6.2±0.6 | 15.4±1.5 | 6.8±2.4 | 0.8±0.4 | 2.2±1.2 |
| 26 | N-acetyl sulfadiazine | aSDZ | 1.5±0.3 | 1.5±0.2 | 1.5±0.2 | 1.5±0.2 | 1.6±0.4 | 1.5±0.1 |
| 27 | Nitrofurantoin | NIT | 0.57±0.09 | 0.53±0.06 | 0.5±0.1 | 0.45±0.05 | 0.6±0.2 | 0.57±0.06 |
| 28 | Aztreonam | ATM | 0.9±0.2 | 0.79±0.08 | 0.7±0.2 | 0.7±0.1 | 0.8±0.3 | 0.7±0.1 |
| 29 | Ampicilloic acid | AMPa | 2.0±0.4 | 1.8±0.2 | 1.3±0.4 | 1.2±0.4 | 1.2±0.4 | 1.1±0.3 |
| 30 | Trimethoprim | TMP | 0.89±0.09 | 0.9±0.1 | 0.9±0.2 | 0.9±0.1 | 0.9±0.1 | 0.9±0.1 |
| 31 | 4-hydroxy-trimethoprim | hTMP | 3.9±0.4 | 3.9±0.4 | 3.8±0.8 | 4.0±0.4 | 3.4±0.5 | 4.0±0.4 |
| 32 | N-acetyl sulfapyridine | aSPY | 1.1±0.2 | 1.1±0.1 | 1.0±0.1 | 1.0±0.1 | 1.0±0.1 | 1.0±0.1 |
| 33 | Tetracycline | TET | 2.3±0.5 | 2.1±0.2 | 1.7±0.6 | 2.1±0.3 | 2.2±0.4 | 2.1±0.2 |
| 34 | Ofloxacin (Levofloxacin) | OFX | 1.1±0.2 | 1.0±0.1 | 1.1±0.2 | 1.1±0.1 | 1.1±0.2 | 1.1±0.1 |
| 35 | Desethylene ciprofloxacin | deCIP | 2.6±0.5 | 2.4±0.2 | 2.4±0.6 | 2.4±0.2 | 2.5±0.6 | 2.3±0.3 |
| 36 | Oxytetracycline | OTC | 3.9±0.8 | 3.8±0.4 | 2.3±0.7 | 3.1±0.6 | 6.4±1.9 | 5.5±1.4 |
| 37 | Desmethyl-ofloxacin | dmOFX | 1.2±0.2 | 1.1±0.1 | 1.2±0.2 | 1.1±0.1 | 1.2±0.4 | 1.1±0.2 |
| 38 | Ceftriaxone | CRO | 0.8±0.2 | 0.8±0.2 | 0.8±0.2 | 1.0±0.2 | 0.7±0.2 | 0.9±0.1 |
| 39 | Cefalexin | LEX | 0.9±0.2 | 0.9±0.1 | 0.9±0.3 | 1.0±0.3 | 1.1±0.3 | 0.9±0.1 |
| 40 | Florfenicol | FLO | 4.9±1.0 | 5.5±0.8 | 4.4±1.3 | 5.3±0.8 | 4.0±1.4 | 5.0±1.0 |
| 41 | Norfloxacin | NOR | 1.4±0.3 | 1.3±0.1 | 1.4±0.4 | 1.4±0.2 | 1.5±0.4 | 1.3±0.4 |
| 42 | Sulfamethoxazole | SMX | 1.3±0.3 | 1.2±0.1 | 1.2±0.3 | 1.2±0.1 | 1.3±0.2 | 1.3±0.1 |
| 43 | Ciprofloxacin | CIP | 4.0±0.6 | 5.1±0.8 | 4.2±1.1 | 4.1±1.0 | 4.5±1.1 | 4.5±0.4 |
| 44 | Enrofloxacin | ENR | 2.4±0.5 | 2.5±0.2 | 3.0±0.8 | 2.6±0.3 | 2.4±0.7 | 2.5±0.2 |
| 45 | Danofloxacin | DFX | 3.1±1.6 | 11.4±3.4 | 2.9±0.9 | 4.0±0.6 | 4.2±1.3 | 5.5±1.1 |
| 46 | Lomefloxacin | LOM | 2.2±0.4 | 2.2±0.2 | 2.4±0.6 | 2.5±0.6 | 2.4±0.6 | 2.4±0.2 |
| 47 | Ampicillin | AMP | 5.1±0.8 | 5.2±0.5 | 3.3±1.3 | 3.9±1.4 | 4.2±1.0 | 4.3±1.5 |
| 48 | Thalidomide | THAL | 2.4±0.2 | 2.3±0.2 | 2.2±0.4 | 2.2±0.2 | 2.1±0.6 | 2.3±0.2 |
| 49 | Ulifloxacin | UFX | 1.0±0.2 | 1.1±0.1 | 0.7±0.2 | 0.7±0.1 | 0.6±0.2 | 0.69±0.07 |
| 50 | Sarafloxacin | SRF | 1.7±0.2 | 2.5±0.4 | 2.1±0.7 | 2.0±0.8 | 2.3±0.7 | 2.1±0.3 |
| 51 | Ofloxacin N-oxide | OFXo | 0.9±0.2 | 0.86±0.09 | 1.0±0.1 | 0.9±0.2 | 1.0±0.3 | 1.0±0.2 |
| 52 | 1-(2-nitrobenzylidenamino)-2,4-imidazolidinedione | NPAHD | 1.9±0.4 | 1.8±0.2 | 1.9±0.5 | 1.7±0.2 | 2.0±0.7 | 1.7±0.3 |
| 53 | Cefixime | CFM | 2.7±0.4 | 2.2±0.3 | 1.6±0.6 | 2.0±0.4 | 1.9±0.7 | 1.8±0.4 |
| 54 | Gatifloxacin | GAT | 1.5±0.3 | 1.6±0.2 | 1.3±0.2 | 1.4±0.4 | 1.4±0.4 | 1.3±0.1 |
| 55 | Chloramphenicol | CHL | 0.5±0.1 | 0.59±0.06 | 0.6±0.2 | 0.62±0.07 | 0.21±0.07 | 0.6±0.1 |
| 56 | N-acetyl sulfamethoxazole | aSMX | 0.8±0.2 | 0.79±0.08 | 0.8±0.2 | 0.8±0.2 | 0.8±0.2 | 0.81±0.09 |
| 57 | Linezolid | LZD | 1.4±0.3 | 1.4±0.1 | 1.5±0.3 | 1.4±0.2 | 1.3±0.4 | 1.4±0.2 |
| 58 | Moxifloxacin | MXF | 2.1±0.4 | 1.8±0.3 | 2.9±1.3 | 2.3±0.6 | 1.3±0.3 | 1.0±0.1 |
| 59 | Hydroxy-norfloxacin | hNOR | 1.1±0.2 | 1.1±0.1 | 1.2±0.4 | 1.1±0.1 | 1.2±0.4 | 1.2±0.2 |
| 60 | Penicilloic G acid | PenGa | 1.0±0.2 | 1.0±0.1 | 1.1±0.3 | 1.0±0.1 | 1.0±0.2 | 1.0±0.1 |
| 61 | Prulifloxacin | PFLX | 1.2±0.2 | 1.1±0.1 | 0.8±0.2 | 1.1±0.4 | 0.7±0.2 | 0.8±0.2 |
| 62 | Azithromycin | AZM | 1.4±0.3 | 1.1±0.1 | 1.0±0.2 | 1.1±0.1 | 1.1±0.3 | 1.0±0.2 |
| 63 | N-desmethyl azithromycin | dmAZM | 0.9±0.2 | 0.9±0.1 | 0.9±0.3 | 0.9±0.3 | 0.9±0.3 | 0.8±0.3 |
| 64 | Ceftiofur | CTF | 1.9±0.4 | 1.8±0.2 | 1.7±0.5 | 1.7±0.4 | 1.5±0.4 | 1.7±0.2 |
| 65 | Deacetyl-ketoconazole | daKTC | 1.7±0.5 | 1.7±0.2 | 1.9±0.8 | 1.9±0.9 | 2.0±0.6 | 1.9±0.5 |
| 66 | Doxycycline | DOX | 0.8±0.2 | 0.8±0.1 | 1.4±0.5 | 1.3±0.3 | 1.5±0.4 | 1.3±0.1 |
| 67 | Clindamycin | CLI | 63.8±12.8 | 74.5±7.4 | 29.5±5.9 | 32.4±9.7 | 64.5±16.1 | 67.3±6.7 |
| 68 | Nalidixic acid | NAL | 1.7±0.2 | 1.7±0.2 | 1.7±0.3 | 2.0±0.2 | 1.7±0.3 | 1.9±0.2 |
| 69 | Besifloxacin | BSF | 2.5±0.4 | 2.0±0.3 | 2.2±1.0 | 2.4±0.5 | 1.3±0.6 | 2.2±0.2 |
| 70 | N-desmethyl clindamycin | dmCLI | 12.7±1.9 | 11.6±2.3 | 12.2±1.8 | 12.2±1.2 | 13.0±2.0 | 12.5±1.2 |
| 71 | Penicillin G | PenG | 1.3±0.5 | 0.9±0.5 | 24.7±6.2 | 21.7±6.5 | 10.3±5.7 | 16.0±6.4 |
|  |  |  |  |  |  |  |  |  |
| *Table S5a continued* | | | | | | | | |
| **#** | **Chemical** | **Abbrev** | **M.phase: low** | **M.phase: high** | **Clean: low** | **Clean: high** | **Dirty: low** | **Dirty: high** |
| 72 | Flumequine | FLU | 2.9±0.4 | 2.8±0.3 | 1.7±0.4 | 1.9±0.2 | 1.7±0.3 | 1.9±0.2 |
| 73 | Penicillin V | PenV | 4.4±0.9 | 4.4±0.7 | 6.7±2.0 | 9.2±1.4 | 1.4±0.6 | 6.2±3.1 |
| 74 | Erythromycin | ERY | 3.7±0.6 | 3.8±0.4 | 4.0±0.4 | 4.0±0.4 | 3.7±0.7 | 4.2±0.4 |
| 75 | N-demethyl erythromycin | dmERY | 3.6±1.3 | 15.7±3.9 | 11.6±3.5 | 18.8±1.9 | 0.8±0.2 | 2.7±0.5 |
| 76 | Ketoconazole | KTC | 5.8±1.2 | 5.8±1.2 | 3.3±1.0 | 4.8±1.0 | 4.1±1.2 | 5.2±0.5 |
| 77 | Nadifloxacin | NAD | 1.0±0.1 | 1.0±0.1 | 1.0±0.2 | 0.9±0.1 | 0.9±0.2 | 0.88±0.09 |
| 78 | Flucloxacillin | FLX | 3.5±0.9 | 3.7±0.6 | 2.9±0.7 | 3.2±0.5 | 2.8±0.7 | 2.9±0.4 |
| 79 | N-desmethyl clarithromycin | dmCLR | 4.4±0.9 | 4.1±0.4 | 4.0±0.6 | 4.1±1.0 | 3.8±0.6 | 4.0±0.4 |
| 80 | Clarithromycin | CLR | 8.2±1.6 | 8.1±0.8 | 7.5±1.1 | 7.8±0.8 | 7.8±1.6 | 7.7±0.8 |
| 81 | Sulfasalazine | SLZ | 1.3±0.3 | 1.2±0.1 | 0.6±0.2 | 0.81±0.09 | 0.7±0.2 | 0.78±0.08 |
| 82 | Delamanid | DMD | 39.4±11.8 | 38.2±5.7 | 36.3±16.4 | 36.3±16.4 | 30.5±12.2 | 37.7±5.6 |
| 83 | 25-O-desacetyl rifabutin | daRFB | 2.2±0.6 | 2.2±0.3 | 1.7±0.3 | 2.0±0.2 | 1.6±0.5 | 1.9±0.2 |
| 84 | 25-desacetyl rifampicin | daRMP | 2.0±0.4 | 1.8±0.2 | 1.3±0.4 | 1.6±0.2 | 0.9±0.4 | 1.2±0.3 |
| 85 | Rifabutin | RFB | 1.4±0.3 | 1.5±0.2 | 1.4±0.4 | 1.5±0.2 | 1.5±0.4 | 1.5±0.2 |
| 86 | Rifampicin | RMP | 1.7±0.5 | 1.7±0.3 | 3.1±0.9 | 2.2±0.3 | 15.4±3.1 | 8.4±2.1 |
| 87 | Bedaquiline | BDQ | #N/A | #N/A | 53.8±24.2 | 59.8±17.9 | 0.4±0.1 | 2.5±1.3 |

| **Table S5b** Chromatographic and validation parameters: Ion ratios for solids analyses, ordered by retention time | | | | | | | | |
| --- | --- | --- | --- | --- | --- | --- | --- | --- |
|  |  |  | Ion Ratio (PI_1_/PI_2_) in mobile phase | | Ion Ratio (PI_1_/PI_2_) in SPM samples | | Ion Ratio (PI_1_/PI_2_) in river sediment | |
| **#** | **Chemical** | **Abbrev** | **0-100 µg L^-1^** | **100-1000 µg L^-1^** | **0-100 µg L^-1^** | **100-1000 µg L^-1^** | **0-100 µg L^-1^** | **100-1000 µg L^-1^** |
| 1 | Gentamycin C1 | GEN1 | #N/A | #N/A | #N/A | #N/A | #N/A | #N/A |
| 2 | Gentamycin C1a | GEN1a | #N/A | #N/A | #N/A | #N/A | #N/A | #N/A |
| 3 | Gentamycin C2 C2a C2b | GEN2 | #N/A | #N/A | #N/A | #N/A | #N/A | #N/A |
| 4 | Kanamycin A | KAN | #N/A | #N/A | #N/A | #N/A | #N/A | #N/A |
| 5 | Capreomycin IA | CAPIa | #N/A | #N/A | #N/A | #N/A | #N/A | #N/A |
| 6 | Capreomycin IB | CAPIb | #N/A | #N/A | #N/A | #N/A | #N/A | #N/A |
| 7 | Streptomycin A | STR | #N/A | #N/A | #N/A | #N/A | #N/A | #N/A |
| 8 | Ethambutol | EMB | #N/A | #N/A | #N/A | #N/A | #N/A | #N/A |
| 9 | D-cycloserine | DCS | 3.2±0.5 | 3.3±0.3 | #N/A | #N/A | #N/A | #N/A |
| 10 | Imipenem | IPM | 1.2±0.2 | 1.0±0.1 | 4.5±1.6 | 3.0±0.9 | 4.5±1.6 | 3.0±0.9 |
| 11 | Isoniazid | INH | 7.9±0.8 | 8.8±0.9 | 6.0±0.9 | 6.0±0.9 | 2.5±0.7 | 2.5±0.7 |
| 12 | Isonicotinic acid | INa | 3.9±0.6 | 3.8±0.4 | 4.2±0.4 | 4.2±0.4 | 4.5±0.9 | 4.5±0.9 |
| 13 | Acetyl-isoniazid | aINH | 0.5±0.1 | 0.52±0.06 | 3.8±1.3 | 3.8±1.3 | 4.0±1.6 | 4.0±1.6 |
| 14 | 5-Hydroxy-pyrazinoic acid | hPZA | 4.5±1.8 | 14.2±1.4 | 36.2±14.5 | 36.2±14.5 | 36.2±14.5 | 36.2±14.5 |
| 15 | Pyrazinamide | PZA | 1.0±0.1 | 1.3±0.1 | 1.2±0.4 | 1.2±0.4 | 0.9±0.3 | 0.9±0.3 |
| 16 | 2-Amino-1-(4-nitrophenyl)-1,3-propanediol | ANP | 2.4±0.4 | 2.4±0.2 | 1.9±0.5 | 1.9±0.5 | 1.8±0.3 | 1.8±0.3 |
| 17 | Lamivudine | 3TC | 6.3±0.9 | 7.4±0.7 | #N/A | #N/A | #N/A | #N/A |
| 18 | Hydroxy-metronidazole | hMTZ | 1.4±0.2 | 1.3±0.1 | #N/A | #N/A | #N/A | #N/A |
| 19 | Metronidazole | MTZ | 1.8±0.3 | 1.8±0.2 | 1.5±0.2 | 1.5±0.2 | 1.8±0.5 | 1.6±0.2 |
| 20 | Amoxicilloic acid | AMXa | 1.3±0.3 | 1.3±0.1 | #N/A | #N/A | #N/A | #N/A |
| 21 | Emtricitabine | FTC | 12.4±2.5 | 13.2±1.3 | 7.6±3.0 | 7.6±3.0 | 7.0±3.8 | 7.0±3.8 |
| 22 | Sulfadiazine | SDZ | 0.9±0.1 | 0.9±0.1 | 1.3±0.1 | 1.3±0.1 | 1.3±0.1 | 1.3±0.1 |
| 23 | Amoxicillin | AMX | 3.3±0.7 | 2.9±0.3 | #N/A | #N/A | #N/A | #N/A |
| 24 | Sulfapyridine | SPY | 1.2±0.1 | 1.2±0.1 | 1.3±0.1 | 1.3±0.1 | 1.3±0.1 | 1.3±0.1 |
| 25 | Meropenem | MEM | 6.3±0.9 | 6.2±0.6 | #N/A | #N/A | #N/A | #N/A |
| 26 | N-acetyl sulfadiazine | aSDZ | 1.5±0.3 | 1.5±0.2 | 1.8±0.2 | 1.8±0.2 | 1.8±0.2 | 1.8±0.2 |
| 27 | Nitrofurantoin | NIT | 1.8±0.3 | 1.9±0.2 | 1.9±0.3 | 1.9±0.3 | 2.0±0.4 | 2.0±0.4 |
| 28 | Aztreonam | ATM | 0.9±0.2 | 0.79±0.08 | 1.3±0.3 | 1.3±0.3 | 1.3±0.3 | 1.2±0.2 |
| 29 | Ampicilloic acid | AMPa | 2.0±0.4 | 1.8±0.2 | #N/A | #N/A | #N/A | #N/A |
| 30 | Trimethoprim | TMP | 0.89±0.09 | 0.9±0.1 | 1.3±0.5 | 1.0±0.1 | 1.3±0.5 | 1.0±0.1 |
| 31 | 4-hydroxy-trimethoprim | hTMP | 3.9±0.4 | 3.9±0.4 | 3.2±0.3 | 3.2±0.3 | 3.3±0.3 | 3.3±0.3 |
| 32 | N-acetyl sulfapyridine | aSPY | 0.9±0.1 | 0.9±0.1 | 1.2±0.2 | 1.2±0.2 | 1.2±0.2 | 1.2±0.2 |
| 33 | Tetracycline | TET | 2.3±0.5 | 2.1±0.2 | 0.6±0.1 | 0.6±0.1 | 0.4±0.2 | 0.4±0.2 |
| 34 | Ofloxacin (Levofloxacin) | OFX | 1.1±0.2 | 1.0±0.1 | 1.2±0.2 | 1.2±0.2 | 1.3±0.4 | 1.3±0.4 |
| 35 | Desethylene ciprofloxacin | deCIP | 2.6±0.5 | 2.4±0.2 | 1.7±0.3 | 1.7±0.3 | 1.8±0.5 | 1.6±0.3 |
| 36 | Oxytetracycline | OTC | 3.9±0.8 | 3.8±0.4 | 2.0±0.8 | 1.6±0.7 | 2.0±0.7 | 2.0±0.7 |
| 37 | Desmethyl-ofloxacin | dmOFX | 1.2±0.2 | 1.1±0.1 | 1.7±0.3 | 1.7±0.3 | 1.7±0.4 | 1.7±0.4 |
| 38 | Ceftriaxone | CRO | 0.8±0.2 | 0.8±0.2 | #N/A | #N/A | #N/A | #N/A |
| 39 | Cefalexin | LEX | 0.9±0.2 | 0.9±0.1 | #N/A | #N/A | #N/A | #N/A |
| 40 | Florfenicol | FLO | 4.9±1.0 | 5.5±0.8 | 2.1±0.6 | 2.1±0.6 | 2.7±0.5 | 2.7±0.5 |
| 41 | Norfloxacin | NOR | 1.4±0.3 | 1.3±0.1 | 1.3±0.3 | 1.3±0.3 | 1.2±0.2 | 1.2±0.2 |
| 42 | Sulfamethoxazole | SMX | 1.3±0.3 | 1.2±0.1 | 2.3±0.7 | 2.3±0.7 | 2.0±0.4 | 2.0±0.4 |
| 43 | Ciprofloxacin | CIP | 4.0±0.6 | 5.1±0.8 | 7.9±0.8 | 7.9±0.8 | 5.7±1.7 | 5.7±1.7 |
| 44 | Enrofloxacin | ENR | 2.4±0.5 | 2.5±0.2 | 2.2±0.4 | 2.2±0.4 | 2.1±0.3 | 2.1±0.3 |
| 45 | Danofloxacin | DFX | 3.1±1.6 | 11.4±3.4 | 4.0±1.4 | 4.0±1.4 | 3.6±0.9 | 3.6±0.9 |
| 46 | Lomefloxacin | LOM | 2.2±0.4 | 2.2±0.2 | 3.1±1.2 | 3.1±1.2 | 3.6±0.5 | 3.6±0.5 |
| 47 | Ampicillin | AMP | 0.20±0.03 | 0.19±0.02 | #N/A | #N/A | #N/A | #N/A |
| 48 | Thalidomide | THAL | 2.4±0.2 | 2.3±0.2 | 3.0±0.7 | 3.0±0.7 | 2.2±0.2 | 2.2±0.2 |
| 49 | Ulifloxacin | UFX | 1.0±0.2 | 1.1±0.1 | 1.0±0.4 | 1.4±0.4 | 1.0±0.4 | 1.4±0.4 |
| 50 | Sarafloxacin | SRF | 0.6±0.1 | 0.40±0.07 | 0.4±0.1 | 0.4±0.1 | 0.5±0.1 | 0.5±0.1 |
| 51 | Ofloxacin N-oxide | OFXo | 0.9±0.2 | 0.86±0.09 | #N/A | #N/A | #N/A | #N/A |
| 52 | 1-(2-nitrobenzylidenamino)-2,4-imidazolidinedione | NPAHD | 1.9±0.4 | 1.8±0.2 | 1.2±0.3 | 1.2±0.3 | 1.3±0.3 | 1.3±0.3 |
| 53 | Cefixime | CFM | 2.7±0.4 | 2.2±0.3 | 1.1±0.4 | 1.1±0.4 | 1.4±0.3 | 1.4±0.3 |
| 54 | Gatifloxacin | GAT | 1.5±0.3 | 1.6±0.2 | 0.6±0.1 | 0.6±0.1 | 0.6±0.1 | 0.6±0.1 |
| 55 | Chloramphenicol | CHL | 0.5±0.1 | 0.59±0.06 | 0.2±0.1 | 0.2±0.1 | 0.7±0.1 | 0.7±0.1 |
| 56 | N-acetyl sulfamethoxazole | aSMX | 0.8±0.2 | 0.79±0.08 | 0.7±0.2 | 0.7±0.2 | 0.74±0.08 | 0.74±0.08 |
| 57 | Linezolid | LZD | 1.4±0.3 | 1.4±0.1 | 1.7±0.2 | 1.7±0.2 | 1.7±0.3 | 1.7±0.3 |
| 58 | Moxifloxacin | MXF | 2.1±0.4 | 1.8±0.3 | 1.7±0.7 | 1.3±0.1 | 1.3±0.3 | 1.3±0.3 |
| 59 | Hydroxy-norfloxacin | hNOR | 0.9±0.2 | 0.9±0.1 | #N/A | #N/A | #N/A | #N/A |
| 60 | Penicilloic G acid | PenGa | 1.0±0.2 | 1.0±0.1 | #N/A | #N/A | #N/A | #N/A |
| 61 | Prulifloxacin | PFLX | 1.2±0.2 | 1.1±0.1 | 0.2±0.1 | 0.1±0.1 | 0.3±0.1 | 0.3±0.1 |
| 62 | Azithromycin | AZM | 1.4±0.3 | 1.1±0.1 | 0.9±0.2 | 0.9±0.2 | 1.0±0.4 | 1.0±0.4 |
| 63 | N-desmethyl azithromycin | dmAZM | 1.1±0.3 | 1.1±0.1 | #N/A | #N/A | 2.9±1.3 | 2.9±1.3 |
| 64 | Ceftiofur | CTF | 1.9±0.4 | 1.8±0.2 | 1.1±0.6 | 3.3±1.5 | 1.1±0.6 | 3.3±1.5 |
| 65 | Deacetyl-ketoconazole | daKTC | 1.7±0.5 | 1.7±0.2 | 4.9±1.5 | 3.2±1.0 | 4.9±1.5 | 3.2±1.0 |
| 66 | Doxycycline | DOX | 0.8±0.2 | 0.8±0.1 | 19.9±6.9 | 19.9±6.9 | 3.8±1.2 | 3.8±1.2 |
| 67 | Clindamycin | CLI | 63.8±12.8 | 74.5±7.4 | 26.9±5.4 | 26.9±5.4 | 26.1±7.8 | 26.1±7.8 |
| 68 | Nalidixic acid | NAL | 1.7±0.2 | 1.7±0.2 | 9.7±2.4 | 9.7±2.4 | 10.7±2.1 | 10.7±2.1 |
| 69 | Besifloxacin | BSF | 2.5±0.4 | 2.0±0.3 | 0.9±0.5 | 0.9±0.5 | 2.0±1.2 | 1.8±1.3 |
| 70 | N-desmethyl clindamycin | dmCLI | 12.7±1.9 | 11.6±2.3 | 8.1±1.6 | 8.1±1.6 | 8.1±1.6 | 8.1±1.6 |
| 71 | Penicillin G | PenG | 1.3±0.5 | 0.9±0.5 | 2.4±1.0 | 2.4±1.0 | 24.1±9.6 | 24.1±9.6 |
|  |  |  |  |  |  |  |  |  |
| *Table S5b continued* | | | | | | | | |
| **#** | **Chemical** | **Abbrev** | **M.phase: low** | **M.phase: high** | **SPM: low** | **SPM: high** | **SED: low** | **SED: high** |
| 72 | Flumequine | FLU | 2.9±0.4 | 2.8±0.3 | 10.2±3.1 | 10.2±3.1 | 7.2±2.2 | 7.2±2.2 |
| 73 | Penicillin V | PenV | 4.4±0.9 | 4.4±0.7 | 3.2±1.7 | 7.7±2.7 | 3.2±1.7 | 7.7±2.7 |
| 74 | Erythromycin | ERY | 3.7±0.6 | 3.8±0.4 | 2.4±1.2 | 3.4±1.0 | 2.9±1.0 | 2.9±1.0 |
| 75 | N-demethyl erythromycin | dmERY | 3.6±1.3 | 15.7±3.9 | 0.3±0.2 | 1.7±0.2 | 0.3±0.2 | 1.7±0.2 |
| 76 | Ketoconazole | KTC | 5.8±1.2 | 5.8±1.2 | 5.2±2.3 | 5.2±2.3 | 3.3±1.6 | 3.3±1.6 |
| 77 | Nadifloxacin | NAD | 1.0±0.1 | 1.0±0.1 | 0.3±0.2 | 0.3±0.2 | 0.7±0.2 | 0.4±0.2 |
| 78 | Flucloxacillin | FLX | 3.5±0.9 | 3.7±0.6 | #N/A | #N/A | #N/A | #N/A |
| 79 | N-desmethyl clarithromycin | dmCLR | 4.4±0.9 | 4.1±0.4 | 2.2±0.9 | 2.8±0.3 | 2.7±0.4 | 2.7±0.4 |
| 80 | Clarithromycin | CLR | 8.2±1.6 | 8.1±0.8 | 4.9±1.5 | 4.9±1.5 | 4.7±0.5 | 4.7±0.5 |
| 81 | Sulfasalazine | SLZ | 1.4±0.3 | 1.2±0.1 | 3.5±0.9 | 3.5±0.9 | 1.7±0.3 | 1.7±0.3 |
| 82 | Delamanid | DMD | 39.4±11.8 | 38.2±5.7 | 32.2±12.9 | 47.2±4.7 | 32.2±12.9 | 47.2±4.7 |
| 83 | 25-O-desacetyl rifabutin | daRFB | 2.2±0.6 | 2.2±0.3 | 0.6±0.2 | 0.9±0.2 | 0.6±0.2 | 0.9±0.2 |
| 84 | 25-desacetyl rifampicin | daRMP | 2.0±0.4 | 1.8±0.2 | #N/A | #N/A | #N/A | #N/A |
| 85 | Rifabutin | RFB | 1.4±0.3 | 1.5±0.2 | #N/A | #N/A | #N/A | #N/A |
| 86 | Rifampicin | RMP | 1.7±0.5 | 1.7±0.3 | #N/A | #N/A | #N/A | #N/A |
| 87 | Bedaquiline | BDQ | #N/A | #N/A | 23.5±11.7 | 35.5±12.4 | 23.5±11.7 | 35.5±12.4 |

| **Table** S**6** Mass spectrometry parameters, ordered by retention time | | | | | | | | | | |
| --- | --- | --- | --- | --- | --- | --- | --- | --- | --- | --- |
| **#** | **Chemical** | **Abbrev** | **Monoisotopic mass** | **Precursor ion (m/z)** | **Product ion 1 (m/z)** | **CV (V)** | **CE (eV)** | **Product ion 2 (m/z)** | **CV (V)** | **CE (eV)** |
| 1 | Gentamycin C1 | GEN1 | 477.3 | 478.4 | 157.1 | 25 | 22 | 139.0 | 25 | 34 |
| 2 | Gentamycin C1a | GEN1a | 449.3 | 450.3 | 160.0 | 34 | 25 | 322.3 | 34 | 15 |
| 3 | Gentamycin C2 C2a C2b | GEN2 | 465.3 | 464.3 | 160.0 | 38 | 28 | 322.3 | 38 | 15 |
| 4 | Kanamycin A | KAN | 484.2 | 485.3 | 163.0 | 38 | 26 | 324.1 | 38 | 17 |
| 5 | Capreomycin IA | CAPIa | 668.3 | 669.4 | 98.0 | 78 | 75 | 127.1 | 78 | 94 |
| 6 | Capreomycin IB | CAPIb | 652.4 | 653.3 | 70.0 | 55 | 100 | 491.3 | 55 | 35 |
| 7 | Streptomycin A | STR | 581.3 | 582.3 | 263.2 | 70 | 41 | 246.2 | 70 | 49 |
| 8 | Ethambutol | EMB | 204.2 | 205.1 | 116.0 | 23 | 14 | 120.0 | 23 | 12 |
| 9 | D-cycloserine | DCS | 102.0 | 102.8 | 75.0 | 18 | 8 | 57.8 | 18 | 12 |
| 10 | Imipenem | IPM | 299.1 | 300.0 | 97.8 | 28 | 30 | 126.0 | 28 | 20 |
| 11 | Isoniazid | INH | 137.1 | 138.0 | 121.0 | 25 | 11 | 93.0 | 25 | 20 |
| 12 | Isonicotinic acid | INa | 123.0 | 124.0 | 80.0 | 40 | 18 | 53.0 | 40 | 23 |
| 13 | Acetyl-isoniazid | aINH | 179.1 | 180.0 | 138.0 | 26 | 21 | 121.0 | 26 | 24 |
| 14 | 5-Hydroxy-pyrazinoic acid | hPZA | 140.0 | 141.0 | 68.0 | 22 | 24 | 95.0 | 22 | 18 |
| 15 | Pyrazinamide | PZA | 123.0 | 124.0 | 107.0 | 28 | 9 | 81.1 | 28 | 16 |
| 16 | 2-Amino-1-(4-nitrophenyl)-1,3-propanediol | ANP | 212.1 | 213.1 | 165.0 | 26 | 14 | 119.0 | 26 | 25 |
| 17 | Lamivudine | 3TC | 229.1 | 230.0 | 112.0 | 36 | 26 | 95.0 | 36 | 42 |
| 18 | Hydroxy-metronidazole | hMTZ | 187.1 | 188.0 | 122.9 | 28 | 13 | 126.1 | 28 | 20 |
| 19 | Metronidazole | MTZ | 171.1 | 172.0 | 128.0 | 28 | 18 | 81.9 | 28 | 28 |
| 20 | Amoxicilloic acid | AMXa | 383.1 | 384.0 | 323.0 | 22 | 16 | 189.0 | 22 | 26 |
| 21 | Emtricitabine | FTC | 247.0 | 248.0 | 130.0 | 38 | 26 | 112.8 | 38 | 42 |
| 22 | Sulfadiazine | SDZ | 250.1 | 251.0 | 92.0 | 32 | 26 | 156.0 | 32 | 15 |
| 23 | Amoxicillin | AMX | 365.1 | 366.1 | 114.0 | 28 | 19 | 208.1 | 28 | 17 |
| 24 | Sulfapyridine | SPY | 249.1 | 250.0 | 92.0 | 42 | 30 | 156.0 | 42 | 16 |
| 25 | Meropenem | MEM | 383.2 | 384.1 | 68.1 | 41 | 25 | 254.0 | 41 | 17 |
| 26 | N-acetyl sulfadiazine | aSDZ | 292.1 | 293.0 | 134.0 | 34 | 26 | 198.0 | 34 | 20 |
| 27 | Nitrofurantoin | NIT | 238.0 | 239.0 | 139.0 | 34 | 16 | 122.0 | 34 | 22 |
| 28 | Aztreonam | ATM | 435.1 | 436.0 | 356.0 | 29 | 11 | 313.0 | 29 | 16 |
| 29 | Ampicilloic acid | AMPa | 367.1 | 368.2 | 324.2 | 27 | 13 | 106.0 | 27 | 35 |
| 30 | Trimethoprim | TMP | 290.1 | 291.2 | 230.2 | 26 | 26 | 123.1 | 26 | 36 |
| 31 | 4-hydroxy-trimethoprim | hTMP | 306.1 | 307.1 | 139.0 | 44 | 24 | 97.0 | 44 | 44 |
| 32 | N-acetyl sulfapyridine | aSPY | 291.1 | 292.0 | 134.0 | 34 | 30 | 198.0 | 34 | 20 |
| 33 | Tetracycline | TET | 444.2 | 445.2 | 410.2 | 32 | 26 | 98.0 | 32 | 58 |
| 34 | Ofloxacin (Levofloxacin) | OFX | 361.1 | 362.0 | 318.2 | 45 | 18 | 261.1 | 45 | 28 |
| 35 | Desethylene ciprofloxacin | deCIP | 305.1 | 306.3 | 190.1 | 32 | 35 | 227.0 | 32 | 30 |
| 36 | Oxytetracycline | OTC | 460.1 | 461.2 | 426.2 | 32 | 27 | 444.3 | 32 | 22 |
| 37 | Desmethyl-ofloxacin | dmOFX | 347.1 | 348.3 | 304.2 | 45 | 22 | 261.2 | 45 | 34 |
| 38 | Ceftriaxone | CRO | 554.0 | 555.1 | 167.1 | 28 | 30 | 125.0 | 28 | 75 |
| 39 | Cefalexin | LEX | 347.1 | 348.1 | 106.0 | 32 | 18 | 174.0 | 32 | 12 |
| 40 | Florfenicol | FLO | 357.0 | 358.0 | 241.0 | 34 | 17 | 206.0 | 34 | 25 |
| 41 | Norfloxacin | NOR | 319.1 | 320.2 | 276.1 | 47 | 18 | 233.1 | 47 | 24 |
| 42 | Sulfamethoxazole | SMX | 253.1 | 254.1 | 92.1 | 36 | 30 | 156.1 | 36 | 20 |
| 43 | Ciprofloxacin | CIP | 331.1 | 332.2 | 231.1 | 42 | 40 | 245.1 | 42 | 32 |
| 44 | Enrofloxacin | ENR | 359.2 | 360.2 | 316.2 | 40 | 20 | 245.0 | 40 | 28 |
| 45 | Danofloxacin | DFX | 357.1 | 358.1 | 340.0 | 65 | 20 | 314.2 | 65 | 20 |
| 46 | Lomefloxacin | LOM | 351.1 | 352.0 | 265.0 | 22 | 24 | 308.0 | 22 | 22 |
| 47 | Ampicillin | AMP | 349.1 | 350.2 | 106.0 | 35 | 17 | 160.0 | 35 | 12 |
| 48 | Thalidomide | THAL | 258.1 | 259.2 | 84.0 | 30 | 20 | 186.1 | 30 | 30 |
| 49 | Ulifloxacin | UFX | 349.1 | 350.2 | 263.0 | 42 | 26 | 306.4 | 42 | 22 |
| 50 | Sarafloxacin | SRF | 385.1 | 385.9 | 367.8 | 46 | 23 | 298.8 | 46 | 28 |
| 51 | Ofloxacin N-oxide | OFXo | 377.1 | 378.3 | 246.9 | 28 | 44 | 316.7 | 28 | 18 |
| 52 | 1-(2-nitrobenzylidenamino)-2,4-imidazolidinedione | NPAHD | 248.1 | 249.0 | 133.9 | 32 | 14 | 103.8 | 32 | 22 |
| 53 | Cefixime | CFM | 453.0 | 454.0 | 219.0 | 60 | 25 | 161.0 | 60 | 32 |
| 54 | Gatifloxacin | GAT | 375.2 | 376.3 | 261.0 | 46 | 36 | 332.1 | 46 | 18 |
| 55 | Chloramphenicol | CHL | 322.0 | 323.0 | 274.8 | 20 | 10 | 304.8 | 20 | 10 |
| 56 | N-acetyl sulfamethoxazole | aSMX | 295.1 | 296.0 | 198.0 | 36 | 23 | 134.0 | 36 | 34 |
| 57 | Linezolid | LZD | 337.1 | 338.0 | 296.0 | 35 | 18 | 195.0 | 35 | 25 |
| 58 | Moxifloxacin | MXF | 401.2 | 402.2 | 261.0 | 54 | 23 | 364.0 | 54 | 27 |
| 59 | Hydroxy-norfloxacin | hNOR | 335.1 | 336.2 | 273.1 | 38 | 34 | 245.1 | 38 | 46 |
| 60 | Penicilloic G acid | PenGa | 262.1 | 353.2 | 309.2 | 24 | 14 | 128.0 | 24 | 34 |
| 61 | Prulifloxacin | PFLX | 461.1 | 462.2 | 360.1 | 40 | 30 | 444.2 | 40 | 44 |
|  |  |  |  |  |  |  |  |  |  |  |
| *Table S6 continued* | | | | | | | | | | |
| **#** | **Chemical** | **Abbrev** | **Monoisotopic mass** | **Precursor ion (m/z)** | **Product ion 1 (m/z)** | **CV (V)** | **CE (eV)** | **Product ion 2 (m/z)** | **CV (V)** | **CE (eV)** |
| 62 | Azithromycin | AZM | 748.5 | 749.5 | 83.1 | 60 | 60 | 116.1 | 60 | 54 |
| 63 | N-desmethyl azithromycin | dmAZM | 734.5 | 735.5 | 115.0 | 44 | 58 | 144.2 | 44 | 58 |
| 64 | Ceftiofur | CTF | 523.0 | 524.0 | 241.1 | 50 | 20 | 125.0 | 50 | 50 |
| 65 | Deacetyl-ketoconazole | daKTC | 488.1 | 489.2 | 136.0 | 66 | 46 | 178.1 | 66 | 35 |
| 66 | Doxycycline | DOX | 444.2 | 445.2 | 267.0 | 40 | 40 | 428.6 | 40 | 38 |
| 67 | Clindamycin | CLI | 424.2 | 425.2 | 126.1 | 42 | 32 | 377.2 | 42 | 20 |
| 68 | Nalidixic acid | NAL | 232.1 | 233.2 | 187.0 | 30 | 28 | 215.1 | 30 | 28 |
| 69 | Besifloxacin | BSF | 393.1 | 394.1 | 356.0 | 34 | 14 | 376.4 | 34 | 24 |
| 70 | N-desmethyl clindamycin | dmCLI | 410.2 | 411.1 | 112.0 | 45 | 28 | 363.1 | 45 | 18 |
| 71 | Penicillin G | PenG | 334.1 | 335.2 | 217.1 | 48 | 16 | 176.0 | 48 | 20 |
| 72 | Flumequine | FLU | 261.1 | 262.2 | 201.9 | 28 | 34 | 244.5 | 28 | 26 |
| 73 | Penicillin V | PenV | 350.1 | 351.2 | 114.2 | 54 | 40 | 160.1 | 54 | 40 |
| 74 | Erythromycin | ERY | 733.5 | 734.3 | 158.2 | 34 | 38 | 115.9 | 34 | 60 |
| 75 | N-desmethyl erythromycin | dmERY | 719.4 | 720.5 | 144.1 | 42 | 38 | 562.5 | 42 | 27 |
| 76 | Ketoconazole | KTC | 530.1 | 531.3 | 82.0 | 60 | 50 | 112.1 | 60 | 58 |
| 77 | Nadifloxacin | NAD | 360.1 | 361.3 | 256.8 | 40 | 44 | 282.9 | 40 | 38 |
| 78 | Flucloxacillin | FLX | 453.1 | 454.0 | 196.0 | 60 | 20 | 116.3 | 60 | 20 |
| 79 | N-desmethyl clarithromycin | dmCLR | 733.5 | 734.5 | 144.0 | 36 | 36 | 102.0 | 36 | 48 |
| 80 | Clarithromycin | CLR | 747.5 | 748.5 | 158.1 | 40 | 31 | 590.4 | 40 | 20 |
| 81 | Sulfasalazine | SLZ | 398.1 | 399.2 | 223.1 | 48 | 34 | 199.0 | 48 | 52 |
| 82 | Delamanid | DMD | 534.2 | 535.0 | 352.0 | 25 | 24 | 406.0 | 25 | 22 |
| 83 | 25-O-desacetyl rifabutin | daRFB | 804.4 | 805.4 | 773.4 | 36 | 32 | 95.0 | 36 | 70 |
| 84 | 25-desacetyl rifampicin | daRMP | 780.4 | 781.4 | 749.4 | 22 | 21 | 95.0 | 22 | 62 |
| 85 | Rifabutin | RFB | 846.4 | 847.5 | 815.5 | 22 | 30 | 112.0 | 22 | 60 |
| 86 | Rifampicin | RMP | 822.4 | 823.4 | 95.0 | 22 | 62 | 791.4 | 22 | 30 |
| 87 | Bedaquiline | BDQ | 554.2 | 555.8 | 58.2 | 45 | 35 | 229.1 | 45 | 25 |
| - | Isoniazid-d4 | - | 141.1 | 142.0 | 125.0 | 25 | 20 | - | - | - |
| - | Metronidazole-d4 | - | 175.1 | 176.0 | 128.0 | 20 | 14 | - | - | - |
| - | Amoxicillin-d4 | - | 369.1 | 370.0 | 114.0 | 28 | 22 | - | - | - |
| - | Nitrofurantoin-13C3 | - | 241.0 | 242.0 | 122.0 | 34 | 22 | - | - | - |
| - | Trimethoprim-d9 | - | 299.2 | 300.2 | 261.1 | 26 | 32 | - | - | - |
| - | Ofloxacin-d3 | - | 364.4 | 365.2 | 321.3 | 56 | 32 | - | - | - |
| - | Ofloxacin desmethyl-d8 | - | 355.2 | 356.0 | 312.2 | 44 | 18 | - | - | - |
| - | Cefalexin-d5 | - | 352.1 | 353.1 | 158.0 | 32 | 15 | - | - | - |
| - | Sulfamethoxazole-d4 | - | 257.1 | 258.1 | 96.1 | 36 | 30 | - | - | - |
| - | Ciprofloxacin-d8 | - | 339.2 | 340.1 | 296.2 | 50 | 35 | - | - | - |
| - | Ampicillin-d5 | - | 354.1 | 355.1 | 160.0 | 40 | 16 | - | - | - |
| - | Chloramphenicol-d5 | - | 327.0 | 328.0 | 309.8 | 20 | 10 | - | - | - |
| - | Azithromycin-d3 | - | 751.5 | 752.5 | 83.1 | 60 | 54 | - | - | - |
| - | Doxycycline-d3 | - | 447.2 | 448.2 | 155.0 | 32 | 36 | - | - | - |
| - | Penicillin G-d7 | - | 341.1 | 342.2 | 183.0 | 48 | 20 | - | - | - |
| - | Flumequine-13C3 | - | 261.1 | 265.2 | 204.9 | 28 | 34 | - | - | - |
| - | Erythromycin-13C,D3 | - | 737.5 | 738.3 | 162.2 | 34 | 38 | - | - | - |
| - | Ketoconazole-d3 | - | 533.2 | 534.3 | 135.2 | 60 | 48 | - | - | - |
| - | Sulfasalazine-d4 | - | 402.1 | 403.0 | 119.0 | 48 | 44 | - | - | - |
| - | Clarithromycin-d3 | - | 750.5 | 751.5 | 161.1 | 40 | 31 | - | - | - |
| - | Rifabutin-d7 | - | 853.5 | 854.5 | 822.5 | 22 | 30 | - | - | - |
| *Differences for solids analyses* | | | | | | | | | | |
| 9 | D-cycloserine | DCS | 102.0 | 102.8 | 57.8 | 18 | 12 | 75.0 | 18 | 8 |
| 14 | 5-Hydroxy-pyrazinoic acid | hPZA | 140.0 | 141.0 | 95.0 | 22 | 18 | 68.0 | 22 | 24 |
| 15 | Pyrazinamide | PZA | 123.0 | 124.0 | 81.1 | 28 | 16 | 107.0 | 28 | 9 |
| 27 | Nitrofurantoin | NIT | 238.0 | 239.0 | 122.0 | 34 | 22 | 139.0 | 34 | 16 |
| 32 | N-acetyl sulfapyridine | aSPY | 291.1 | 292.0 | 198.0 | 34 | 20 | 134.0 | 34 | 30 |
| 47 | Ampicillin | AMP | 349.1 | 350.2 | 160.0 | 35 | 12 | 106.0 | 35 | 17 |
| 50 | Sarafloxacin | SRF | 385.1 | 385.9 | 298.8 | 46 | 28 | 367.8 | 46 | 23 |
| 59 | Hydroxy-norfloxacin | hNOR | 335.1 | 336.2 | 245.1 | 38 | 46 | 273.1 | 38 | 34 |
| 63 | N-desmethyl azithromycin | dmAZM | 734.5 | 735.5 | 144.2 | 44 | 58 | 115.0 | 44 | 58 |
| 81 | Sulfasalazine | SLZ | 398.1 | 399.2 | 199.0 | 48 | 52 | 223.1 | 48 | 34 |

| **Table S7** Equations for calculating concentration, instrument performance, and method performance | | | | |
| --- | --- | --- | --- | --- |
| **Calculation** | **Abbrev.** | **Equation** | **Units** | **Terms / description** |
| Instrument response | R | < An > * ( [ISTD] / <ISTD> ) |  | Analyte (An); Internal standard (ISTD); Area < >; Concentration [ ] |
| LC vial concentration | [Vial] | (R - c) / m | µg L^-1^ | Gradient (m); intercept (c) |
| Aqueous sample concentration | [AQ] | [Vial] / (V _sample_ / V _elution_) | µg L^-1^ | Volume (V) |
| Sediment sample concentration | [SED] | ([Vial] * V _elution_) / M _sample_ | ng g^-1^ | Mass (M) |
| SPM sample concentration | [SPM] | (([Vial] * V _elution_)) / (M _sample_ / M _total_)) / V _sample_ | ng L^-1^ | (or as ng/g, calculated same as [SED]) |
| Instrument quantification limit | IQL | [Vial _m. phase_] _S/N≥10_ | µg L^-1^ | Concentration at which ≥2 mobile phase quality controls achieved S/N≥10 |
| Instrument detection limit | IDL | (IQL/10) *3 | µg L^-1^ | Extrapolated from IQL |
| Method quantification limit | MQL | IQL / (Rec _matrix_ x CF) | µg L^-1^ | Matrix-specific recovery as a decimal (Rec _matrix_); sample concentration factor (CF) |
| Method detection limit | MDL | IDL / (Rec _matrix_ x CF) | µg L^-1^ |  |
| Accuracy |  | ([Spiked _observed_] / [Spiked _theoretical_]) *100 | % | *Desired 100%* |
| Precision |  | Standard deviation (Accuracy) | % | *Desired 0%* |
| Recovery (absolute) | REC _abs_ | ((<Spiked _matrix_> - <Blank _matrix_>) / <Spiked _QC_ >) *100 | % | Analyte recovery relative to quality control (via peak area) |
| Recovery (relative) | REC _rel_ | (([Spiked _observed_] - [Blank _matrix_]) / [Spiked _theoretical_]) *100 | % | Analyte recovery relative to ISTD (via concentration) |

| **Table S8** Method recovery from urban matrices (n ≤ 18), ordered by drug class | | | | | | |  | |  | | |
| --- | --- | --- | --- | --- | --- | --- | --- | --- | --- | --- | --- |
|  |  | **Average percentage recovery from urban matrices (%)** | | | | | | | | |  |
| Drug Class | Abbrev | MilliQ water | River water | Effluent WW | Influent WW | Influent SPM | | River sediment | |  |  |
| **Sulfonamide &** |  |  |  |  |  |  | |  | |  |  |
| **Trimethoprim** | SDZ | 88.6 ± 9.4 | 89.3 ± 10.5 | 79.8 ± 8.2 | 66.1 ± 12.0 | 230.8 ± 18.4 | | 123.1 ± 7.6 | |  |  |
|  | SPY | 91.5 ± 9.7 | 88.7 ± 12.1 | 83.3 ± 13.1 | 113.7 ± 28.1 | 190.0 ± 60.4 | | 109.3 ± 11.1 | |  |  |
|  | SMX | 86.4 ± 5.8 | 94.3 ± 5.6 | 95.3 ± 2.7 | 109.2 ± 17.5 | 104.2 ± 11.4 | | 93.3 ± 3.0 | |  |  |
|  | SLZ | 94.8 ± 17.1 | 108.7 ± 19.3 | 83.0 ± 9.9 | 73.2 ± 13.1 | 83.8 ± 21.1 | | 125.4 ± 32.2 | |  |  |
|  | TMP | 93.7 ± 16.7 | 97.4 ± 24.5 | 161.3 ± 29.5 | 155.2 ± 38.7 | 119.1 ± 29.2 | | 93.1 ± 12.2 | |  |  |
|  | aSDZ | 95.7 ± 16.6 | 102.7 ± 14.6 | 103.5 ± 13.1 | 90.6 ± 17.2 | excluded | | 156.4 ± 21.6 | |  |  |
|  | aSPY | 98.2 ± 8.4 | 109.0 ± 18.6 | 110.8 ± 25.1 | 123.1 ± 26.3 | excluded | | 179.6 ± 24.0 | |  |  |
|  | aSMX | 90.4 ± 14.4 | 101.5 ± 18.6 | 103.4 ± 17.9 | 95.9 ± 37.1 | excluded | | 159.8 ± 24.0 | |  |  |
|  | hTMP | 74.0 ± 8.1 | 116.2 ± 24.2 | 132.4 ± 36.8 | 127.8 ± 23.7 | excluded | | 198.3 ± 21.4 | |  |  |
| **Macrolide** | AZM | excluded | 91.0 ± 10.2 | 112.2 ± 10.9 | insufficient data | insufficient data | | insufficient data | |  |  |
| **& Lincomycin** | ERY | 93.4 ± 9.1 | 103.6 ± 2.7 | 113.6 ± 8.5 | 94.6 ± 14.0 | insufficient data | | 116.2 ± 15.5 | |  |  |
|  | CLR | 110.3 ± 9.1 | 108.2 ± 6.8 | 113.6 ± 9.5 | 109.8 ± 18.2 | 86.0 ± 25.1 | | 102.9 ± 5.2 | |  |  |
|  | CLI | 119.6 ± 39.6 | 101.4 ± 12.1 | 123.4 ± 19.4 | > linear range | > linear range | | 187.8 ± 34.4 | |  |  |
|  | dmAZM | excluded | 117.7 ± 33.1 | 121.4 ± 31.4 | insufficient data | excluded | | 11.1 ± 4.9 | |  |  |
|  | dmERY | 100.9 ± 13.1 | 102.1 ± 6.1 | 111.0 ± 18.4 | 111.0 ± 24.9 | insufficient data | | 117.2 ± 17.5 | |  |  |
|  | dmCLR | 88.7 ± 15.1 | 77.8 ± 9.6 | 81.6 ± 8.8 | 68.0 ± 11.7 | 36.0 ± 27.4 | | 90.0 ± 4.2 | |  |  |
|  | dmCLI | 110.3 ± 31.2 | 146.8 ± 55.4 | 97.7 ± 17.6 | > linear range | > linear range | | > linear range | |  |  |
| **β-LACTAMS** |  |  |  |  |  |  | |  | |  |  |
| Penicillin | AMX | 88.6 ± 30.1 | 42.3 ± 8.6 | 5.6 ± 1.7 | matrix interference | insufficient data | | insufficient data | |  |  |
|  | AMP | 72.2 ± 25.7 | 73.1 ± 18.5 | 9.5 ± 5.2 | 27.0 ± 6.0 | 67.7 ± 16.9 | | 25.3 ± 2.8 | |  |  |
|  | FLX | 110.8 ± 33.4 | excluded | 102.9 ± 16.9 | 91.8 ± 47.5 | 23.8 ± 7.2 | | excluded | |  |  |
|  | PenG | 93.7 ± 21.5 | 126.8 ± 42.3 | 103.8 ± 18.7 | 27.4 ± 5.5 | 11.6 ± 7.5 | | 61.7 ± 9.7 | |  |  |
|  | PenV | 105.2 ± 40.9 | 76.4 ± 21.3 | 107.3 ± 18.6 | 36.9 ± 11.7 | excluded | | 71.0 ± 22.5 | |  |  |
|  | AMXa | 0.3 ± 0.1 | 1.6 ± 0.6 | 7.2 ± 3.5 | 2.0 ± 1.0 | insufficient data | | insufficient data | |  |  |
|  | AMPa | 4.6 ± 3.2 | 11.5 ± 6.7 | 6.3 ± 1.1 | 4.7 ± 1.3 | 8.5 ± 10.9 | | insufficient data | |  |  |
|  | PenGa | 87.6 ± 49.6 | 13.0 ± 3.5 | 18.6 ± 6.9 | 9.7 ± 5.6 | excluded | | 0.3 ± 0.2 | |  |  |
| Cefalosporin | LEX | 66.9 ± 28.4 | 101.9 ± 20.5 | 52.1 ± 7.9 | 63.5 ± 5.9 | excluded | | excluded | |  |  |
|  | CFM | 84.6 ± 20.8 | 112.9 ± 26.3 | excluded | 113.6 ± 23.2 | 65.7 ± 21.6 | | 66.0 ± 16.0 | |  |  |
|  | CTF | 93.1 ± 28.4 | 103.8 ± 22.9 | 108.9 ± 20.1 | excluded | excluded | | 2.2 ± 0.6 | |  |  |
|  | CRO | excluded | 96.0 ± 17.0 | 110.0 ± 11.3 | 155.0 ± 20.7 | insufficient data | | insufficient data | |  |  |
| Monobactam | ATM | 10.7 ± 3.4 | insufficient data | insufficient data | insufficient data | 116.7 ± 29.7 | | 55.8 ± 12.5 | |  |  |
| Carbapenem | IPM | not recovered | not recovered | not recovered | not recovered | not recovered | | not recovered | |  |  |
|  | MEM | not recovered | not recovered | not recovered | not recovered | not recovered | | not recovered | |  |  |
|  |  |  |  |  |  |  | |  | |  |  |
| **Quinolone** | BSF | excluded | 46.3 ± 14.4 | 64.4 ± 26.2 | 119.2 ± 14.5 | insufficient data | | 8.5 ± 4.9 | |  |  |
|  | CIP | 78.6 ± 16.0 | 104.4 ± 18.3 | 88.5 ± 8.4 | 132.7 ± 28.6 | > linear range | | 70.9 ± 28.8 | |  |  |
|  | DFX | 81.0 ± 25.9 | 93.4 ± 14.5 | 96.2 ± 13.9 | 108.1 ± 16.2 | 81.0 ± 14.2 | | 24.5 ± 4.9 | |  |  |
|  | ENR | 126.4 ± 25.3 | 161.0 ± 15.3 | 131.9 ± 23.6 | 121.6 ± 35.9 | 138.4 ± 53.7 | | 46.4 ± 15.5 | |  |  |
|  | FLU | 91.5 ± 7.6 | 103.0 ± 17.6 | 93.5 ± 3.0 | 99.4 ± 2.8 | 110.4 ± 25.4 | | 81.6 ± 13.3 | |  |  |
|  | GAT | 111.7 ± 28.8 | 101.1 ± 14.2 | 116.3 ± 34.9 | 108.6 ± 35.1 | 130.6 ± 44.7 | | 127.2 ± 26.0 | |  |  |
|  | LOM | 115.3 ± 15.9 | 128.4 ± 10.7 | 132.4 ± 16.4 | 105.6 ± 26.2 | 126.5 ± 29.2 | | 134.5 ± 19.6 | |  |  |
|  | MXF | 59.6 ± 19.2 | 112.5 ± 18.9 | 117.4 ± 34.3 | 123.5 ± 9.5 | 141.0 ± 68.1 | | 151.5 ± 28.9 | |  |  |
|  | NAD | 92.7 ± 21.1 | 94.1 ± 19.0 | 134.8 ± 16.0 | 112.1 ± 10.7 | 13.6 ± 9.0 | | 107.9 ± 31.6 | |  |  |
|  | NAL | 112.8 ± 8.6 | 118.1 ± 18.8 | 113.9 ± 10.1 | 91.8 ± 0.8 | excluded | | 95.8 ± 11.7 | |  |  |
|  | NOR | 97.4 ± 32.0 | 124.4 ± 21.2 | 118.5 ± 35.3 | 145.7 ± 21.9 | 109.0 ± 48.7 | | 89.9 ± 24.7 | |  |  |
|  | OFX | 96.8 ± 17.2 | 93.7 ± 12.9 | 89.7 ± 10.0 | 73.2 ± 12.4 | 148.8 ± 47.0 | | 65.6 ± 10.3 | |  |  |
|  | PFLX | 79.1 ± 38.5 | 141.9 ± 35.3 | excluded | 168.1 ± 52.4 | excluded | | excluded | |  |  |
|  | SRF | excluded | 99.9 ± 16.4 | 100.4 ± 10.8 | 99.9 ± 20.0 | 87.0 ± 41.2 | | 75.5 ± 12.5 | |  |  |
|  | deCIP | excluded | 74.0 ± 19.3 | 72.3 ± 21.8 | 103.8 ± 28.1 | 36.8 ± 5.9 | | 25.8 ± 6.7 | |  |  |
|  | hNOR | insufficient data | 52.0 ± 16.1 | insufficient data | 93.3 ± 23.4 | insufficient data | | insufficient data | |  |  |
|  | OFXo | 140.9 ± 50.8 | 148.6 ± 40.8 | 95.6 ± 31.8 | 98.5 ± 17.3 | excluded | | 29.8 ± 14.8 | |  |  |
|  | dmOFX | 130.4 ± 8.4 | 139.1 ± 10.1 | 120.2 ± 10.9 | 112.3 ± 13.6 | 134.6 ± 32.5 | | 125.7 ± 26.9 | |  |  |
|  | UFX | 60.4 ± 25.1 | 94.6 ± 25.0 | 118.8 ± 26.5 | 131.1 ± 34.0 | 110.1 ± 64.5 | | 81.0 ± 21.8 | |  |  |
| **TB DRUGS** |  |  |  |  |  |  | |  | |  |  |
| TB (1st line) | INH | 119.4 ± 30.0 | excluded | excluded | 98.9 ± 24.1 | excluded | | 1.2 ± 1.0 | |  |  |
|  | PZA | 8.3 ± 3.3 | 6.4 ± 0.4 | excluded | excluded | 38.4 ± 31.3 | | 13.5 ± 7.4 | |  |  |
|  | EMB | 25.3 ± 15.3 | 46.8 ± 2.3 | 30.1 ± 3.5 | 23.7 ± 4.0 | 6.7 ± 3.1 | | 0.4 ± 0.2 | |  |  |
|  | RMP | 76.9 ± 31.9 | 80.5 ± 38.5 | 70.2 ± 42.5 | insufficient data | excluded | | 23.3 ± 10.3 | |  |  |
|  | RFB | 79.9 ± 21.7 | 114.5 ± 10.1 | 109.3 ± 6.0 | 89.0 ± 11.4 | 77.5 ± 26.4 | | 89.6 ± 5.3 | |  |  |
|  | INa | 32.1 ± 11.4 | 41.4 ± 25.3 | 71.7 ± 56.1 | 20.8 ± 8.9 | excluded | | 19.6 ± 17.6 | |  |  |
|  | aINH | 97.6 ± 38.6 | 67.3 ± 28.1 | 85.4 ± 36.2 | 41.9 ± 9.8 | 49.5 ± 25.6 | | 122.5 ± 64.2 | |  |  |
|  | |  |  |  |  |  | |  | |  |  |
| *Table S8 continued* | |  |  |  |  |  | |  | |  |  |
| **Drug class** | **Abbrev** | **MilliQ water** | **River water** | **Effluent WW** | **Influent WW** | **Influent SPM** | | **River sediment** | |  |  |
|  | hPZA | excluded | excluded | excluded | excluded | excluded | | excluded | |  |  |
|  | daRMP | 78.4 ± 21.1 | 91.6 ± 16.8 | insufficient data | 99.9 ± 32.0 | insufficient data | | insufficient data | |  |  |
|  | daRFB | 82.9 ± 18.4 | 84.4 ± 36.6 | excluded | 90.4 ± 26.1 | excluded | | 93.1 ± 8.5 | |  |  |
| TB (MDR) | CAP | not recovered | 15.1 ± 1.4 | 0.8 ± 1.5 | 1.5 ± 1.6 | not recovered | | 15.1 ± 1.4 | |  |  |
|  | GEN | insufficient data | 9.9 ± 1.4 | 18.4 ± 0.5 | insufficient data | insufficient data | | 9.9 ± 1.4 | |  |  |
|  | KAN | 4.1 ± 2.8 | 3.9 ± 1.4 | 5.1 ± 4.8 | insufficient data | 4.1 ± 2.8 | | 3.9 ± 1.4 | |  |  |
|  | STR | 25.2 ± 2.5 | 24.2 ± 0.2 | 23.2 ± 1.1 | 4.5 ± 2.0 | 25.2 ± 2.5 | | 24.2 ± 0.2 | |  |  |
|  | DCS | excluded | excluded | excluded | excluded | excluded | | excluded | |  |  |
| TB (other) | DMD | excluded | 50.5 ± 12.7 | 146.2 ± 39.4 | > linear range | 81.7 ± 43.7 | | 63.7 ± 10.7 | |  |  |
|  | BDQ | excluded | 95.5 ± 26.5 | 158.5 ± 25.0 | excluded | insufficient data | | excluded | |  |  |
|  | LZD | 107.5 ± 8.8 | 104.6 ± 19.8 | 91.9 ± 15.9 | 112.9 ± 20.7 | 143.3 ± 27.6 | | 132.4 ± 10.0 | |  |  |
|  | THAL | 92.3 ± 21.6 | 117.5 ± 23.0 | 129.9 ± 37.6 | 86.2 ± 12.0 | 167.1 ± 46.3 | | 156.7 ± 22.1 | |  |  |
| **OTHER** |  |  |  |  |  |  | |  | |  |  |
| Amphenicol | CHL | 104.0 ± 12.0 | 118.3 ± 21.0 | 114.1 ± 15.1 | 114.9 ± 24.2 | 41.3 ± 6.9 | | 99.9 ± 17.9 | |  |  |
|  | FLO | 134.5 ± 21.9 | 143.7 ± 26.0 | 135.1 ± 22.7 | 136.9 ± 20.2 | 125.5 ± 31.5 | | 164.5 ± 22.3 | |  |  |
|  | ANP | 40.9 ± 6.4 | 52.0 ± 18.4 | 24.9 ± 4.6 | 35.5 ± 2.0 | 22.9 ± 12.9 | | 10.5 ± 4.7 | |  |  |
| Cycline | DOX | 96.1 ± 24.5 | 73.3 ± 11.5 | 69.7 ± 8.8 | 29.6 ± 12.7 | insufficient data | | 5.3 ± 4.3 | |  |  |
|  | OTC | 87.4 ± 38.8 | 82.2 ± 17.5 | 45.2 ± 7.9 | 47.3 ± 17.7 | 7.2 ± 5.6 | | 4.1 ± 3.9 | |  |  |
|  | TET | 72.7 ± 4.8 | 65.5 ± 18.1 | 112.8 ± 29.6 | 88.4 ± 39.0 | 18.4 ± 7.3 | | excluded | |  |  |
| Nitrofuran | NIT | 88.0 ± 4.6 | 93.0 ± 23.5 | 127.0 ± 33.3 | 86.7 ± 14.2 | 113.4 ± 24.7 | | 86.9 ± 6.3 | |  |  |
|  | NPAHD | 96.9 ± 15.1 | 80.8 ± 10.5 | 68.7 ± 10.8 | 84.5 ± 12.3 | 46.1 ± 10.7 | | 74.6 ± 7.1 | |  |  |
| Azole | MTZ | 113.9 ± 4.6 | 116.2 ± 5.3 | 113.8 ± 5.3 | 109.7 ± 8.0 | 121.9 ± 7.3 | | 120.5 ± 7.3 | |  |  |
|  | KTC | 52.7 ± 20.7 | 34.6 ± 10.6 | 42.3 ± 12.7 | 103.7 ± 21.2 | excluded | | 51.1 ± 14.5 | |  |  |
|  | hMTZ | 19.9 ± 6.3 | 20.6 ± 5.9 | 59.9 ± 12.4 | 29.6 ± 12.7 | 30.2 ± 8.1 | | insufficient data | |  |  |
|  | daKTC | 97.5 ± 24.1 | excluded | 90.4 ± 23.8 | matrix interference | insufficient data | | insufficient data | |  |  |
| ARV | FTC | 84.3 ± 16.0 | 100.7 ± 22.9 | 93.3 ± 13.7 | 95.5 ± 32.2 | 172.6 ± 37.2 | | 104.4 ± 19.3 | |  |  |
|  | 3TC | 36.3 ± 6.0 | 42.1 ± 9.2 | 71.0 ± 14.3 | 31.5 ± 17.2 | insufficient data | | insufficient data | |  |  |
| Grey text: poor instrument performance (accuracy and precision) | | | | | | | | | |  |  |

| **Table S9** Method detection and quantification limits per matrix, ordered by retention time | | | | | | | | | | | | | |
| --- | --- | --- | --- | --- | --- | --- | --- | --- | --- | --- | --- | --- | --- |
|  |  | **MilliQ water**  **(ng L^-1^)** | | **River water**  **(ng L^-1^)** | | **Effluent WW**  **(ng L^-1^)** | | **Influent WW**  **(ng L^-1^)** | | **Influent SPM**  **(ng g^-1^)** | | **River sediment**  **(ng g^-1^)** | |
| **Class** | **Abbrev** | MDL | MQL | MDL | MQL | MDL | MQL | MDL | MQL | MDL | MQL | MDL | MQL |
| **Sulfonamide &** |  |  |  |  |  |  |  |  |  |  |  |  |  |
| **Trimethoprim** | SDZ | 0.08 | 0.28 | 0.08 | 0.28 | 0.19 | 0.63 | 0.23 | 0.76 | - | - | 0.02 | 0.08 |
|  | SPY | 0.016 | 0.055 | 0.017 | 0.056 | 0.036 | 0.120 | 0.026 | 0.088 | 0.003 | 0.010 | 0.005 | 0.017 |
|  | SMX | 0.009 | 0.029 | 0.008 | 0.026 | 0.016 | 0.052 | 0.014 | 0.046 | 0.003 | 0.009 | 0.003 | 0.010 |
|  | SLZ | 1.98 | 6.59 | 1.72 | 5.75 | 4.52 | 15.1 | 5.13 | 17.09 | 3.44 | 11.5 | 2.21 | 7.38 |
|  | TMP | 0.80 | 2.67 | 0.77 | 2.57 | 0.93 | 3.10 | 0.97 | 3.22 | 0.24 | 0.81 | 0.30 | 0.99 |
|  | aSDZ | 0.11 | 0.37 | 0.10 | 0.34 | 0.20 | 0.68 | 0.23 | 0.77 | - | - | 0.02 | 0.06 |
|  | aSPY | 0.85 | 2.83 | 0.76 | 2.55 | 1.50 | 5.01 | 1.35 | 4.51 | - | - | 0.12 | 0.41 |
|  | aSMX | 0.11 | 0.35 | 0.09 | 0.31 | 0.18 | 0.61 | 0.20 | 0.66 | - | - | - | - |
|  | hTMP | 0.03 | 0.09 | 0.02 | 0.05 | 0.03 | 0.10 | 0.03 | 0.10 | - | - | - | - |
| **Macrolide** | AZM | - | - | 0.08 | 0.27 | 0.13 | 0.45 | - | - | - | - | - | - |
| **& Lincomycin** | ERY | 0.008 | 0.027 | 0.007 | 0.024 | 0.013 | 0.044 | 0.016 | 0.053 | - | - | 0.002 | 0.008 |
|  | CLR | 0.007 | 0.023 | 0.007 | 0.023 | 0.013 | 0.044 | 0.014 | 0.046 | 0.003 | 0.011 | 0.003 | 0.009 |
|  | CLI | 0.63 | 2.09 | 0.74 | 2.46 | 1.22 | 4.05 | - | - | - | - | 0.15 | 0.49 |
|  | dmAZM | - | - | 1.59 | 5.31 | 3.09 | 10.3 | - | - | - | - | 6.28 | 20.9 |
|  | dmERY | 0.01 | 0.04 | 0.01 | 0.04 | 0.02 | 0.07 | 0.02 | 0.07 | - | - | 0.003 | 0.012 |
|  | dmCLR | 0.03 | 0.09 | 0.03 | 0.11 | 0.06 | 0.20 | 0.07 | 0.25 | 0.03 | 0.09 | 0.01 | 0.03 |
|  | dmCLI | 0.007 | 0.023 | 0.005 | 0.017 | 0.015 | 0.051 | - | - | - | - | - | - |
| **β-LACTAMS** |  |  |  |  |  |  |  |  |  |  |  |  |  |
| Penicillin | AMX | 8.46 | 28.2 | 17.7 | 59.0 | 267 | 890 | - | - | - | - | - | - |
|  | AMP | 10.4 | 34.6 | 10.3 | 34.2 | 157 | 524 | 55.6 | 185 | 4.26 | 14.2 | 11.0 | 36.6 |
|  | FLX | 0.68 | 2.26 | - | - | 1.46 | 4.86 | 1.63 | 5.45 | 1.21 | 4.04 | - | - |
|  | PenG | 0.80 | 2.67 | 0.59 | 1.97 | 1.44 | 4.82 | 5.47 | 18.2 | 2.49 | 8.31 | 0.45 | 1.50 |
|  | PenV | 14.3 | 47.5 | 19.6 | 65.4 | 28.0 | 93.2 | 81.3 | 271 | - | - | 7.83 | 26.1 |
|  | AMXa | 445 | 1482 | 96.7 | 322 | 41.5 | 138 | 146 | 488 | - | - | - | - |
|  | AMPa | 16.2 | 53.9 | 6.54 | 21.8 | 23.9 | 79.6 | 31.8 | 106 | 3.39 | 11.3 | - | - |
|  | PenGa | 0.86 | 2.86 | 5.76 | 19.21 | 8.07 | 26.9 | 15.4 | 51.5 | - | - | 83.3 | 278 |
| Cefalosporin | LEX | 2.80 | 9.34 | 1.84 | 6.14 | 7.20 | 24.0 | 5.91 | 19.7 | - | - | - | - |
|  | CFM | 8.87 | 29.6 | 6.65 | 22.2 | - | - | 13.2 | 44.0 | 4.39 | 14.6 | 4.21 | 14.0 |
|  | CTF | 0.81 | 2.69 | 0.72 | 2.41 | 1.38 | 4.59 | - | - | - | - | 12.7 | 42.4 |
|  | CRO | - | - | 39.1 | 130 | 68.2 | 227 | 48.4 | 161 | - | - | - | - |
| Monobactam | ATM | 14.0 | 46.6 | - | - | - | - | - | - | 0.49 | 1.65 | 1.00 | 3.32 |
| Carbapenem | IPM | - | - | - | - | - | - | - | - | - | - | - | - |
|  | MEM | - | - | - | - | - | - | - | - | - | - | - | - |
|  |  |  |  |  |  |  |  |  |  |  |  |  |  |
| **Quinolone** | BSF | - | - | 4.05 | 13.5 | 5.83 | 19.4 | 3.15 | 10.5 | - | - | 8.21 | 27.4 |
|  | CIP | 0.95 | 3.18 | 0.72 | 2.39 | 1.70 | 5.65 | 1.13 | 3.77 | - | - | 0.39 | 1.31 |
|  | DFX | 9.26 | 30.9 | 8.03 | 26.8 | 15.6 | 52.0 | 13.9 | 46.3 | 3.56 | 11.9 | 11.3 | 37.8 |
|  | ENR | 0.12 | 0.40 | 0.09 | 0.31 | 0.23 | 0.76 | 0.25 | 0.82 | 0.04 | 0.14 | 0.12 | 0.40 |
|  | FLU | 0.02 | 0.05 | 0.01 | 0.05 | 0.03 | 0.11 | 0.03 | 0.10 | 0.005 | 0.017 | 0.007 | 0.023 |
|  | GAT | 0.01 | 0.04 | 0.01 | 0.05 | 0.03 | 0.09 | 0.03 | 0.09 | 0.004 | 0.015 | 0.004 | 0.015 |
|  | LOM | 0.13 | 0.43 | 0.12 | 0.39 | 0.23 | 0.76 | 0.28 | 0.95 | 0.05 | 0.15 | 0.04 | 0.14 |
|  | MXF | 3.15 | 10.5 | 1.67 | 5.56 | 3.19 | 10.6 | 3.04 | 10.1 | 0.51 | 1.70 | 0.46 | 1.53 |
|  | NAD | 1.62 | 5.39 | 1.59 | 5.31 | 2.23 | 7.42 | 2.68 | 8.92 | 4.24 | 14.14 | 0.51 | 1.72 |
|  | NAL | 0.01 | 0.04 | 0.01 | 0.04 | 0.03 | 0.09 | 0.03 | 0.11 | - | - | 0.01 | 0.02 |
|  | NOR | 0.015 | 0.051 | 0.012 | 0.040 | 0.025 | 0.084 | 0.021 | 0.069 | 0.005 | 0.018 | 0.006 | 0.021 |
|  | OFX | 0.15 | 0.52 | 0.16 | 0.53 | 0.33 | 1.12 | 0.41 | 1.37 | 0.04 | 0.13 | 0.08 | 0.28 |
|  | PFLX | 1.90 | 6.32 | 1.06 | 3.52 | - | - | 1.78 | 5.95 | - | - | - | - |
|  | SRF | - | - | 0.75 | 2.50 | 1.49 | 4.98 | 1.50 | 5.01 | 0.33 | 1.11 | 0.37 | 1.23 |
|  | deCIP | - | - | 1.01 | 3.38 | 2.07 | 6.91 | 1.44 | 4.82 | 0.78 | 2.61 | 1.08 | 3.59 |
|  | hNOR | - | - | 34.6 | 115 | - | - | 38.6 | 129 | - | - | - | - |
|  | OFXo | 12.8 | 42.6 | 12.1 | 40.4 | 37.7 | 126 | 36.5 | 122 | - | - | 22.4 | 74.6 |
|  | dmOFX | 0.58 | 1.92 | 0.54 | 1.80 | 1.25 | 4.16 | 1.34 | 4.45 | 0.21 | 0.71 | 0.22 | 0.74 |
|  | UFX | 12.4 | 41.4 | 7.93 | 26.4 | 12.6 | 42.1 | 11.4 | 38.1 | 2.62 | 8.73 | 3.43 | 11.4 |
| **TB DRUGS** |  |  |  |  |  |  |  |  |  |  |  |  |  |
| TB (1st line) | INH | 0.63 | 2.09 | - | - | - | - | 1.52 | 5.06 | - | - | 23.4 | 78.0 |
|  | PZA | 90.9 | 303 | 117 | 390 | - | - | - | - | 1.88 | 6.27 | 5.15 | 17.2 |
|  | EMB | 0.06 | 0.20 | 0.03 | 0.11 | 0.10 | 0.33 | 0.13 | 0.42 | 0.09 | 0.29 | 1.25 | 4.18 |
|  | RMP | 2.44 | 8.12 | 2.33 | 7.77 | 5.35 | 17.8 | - | - | - | - | 2.98 | 9.92 |
|  | RFB | 0.94 | 3.13 | 0.66 | 2.18 | 1.37 | 4.58 | 1.68 | 5.62 | 0.37 | 1.24 | 0.31 | 1.03 |
|  | INa | 2.34 | 7.79 | 1.81 | 6.03 | 2.09 | 6.97 | 7.20 | 24.0 | - | - | 1.42 | 4.73 |
|  | aINH | 0.77 | 2.56 | 1.11 | 3.72 | 1.76 | 5.86 | 3.58 | 11.9 | 0.58 | 1.94 | 0.23 | 0.76 |
|  | hPZA | - | - | - | - | - | - | - | - | - | - | - | - |
|  | daRMP | 9.57 | 31.9 | 8.19 | 27.3 | - | - | 15.0 | 50.0 | - | - | - | - |
|  | daRFB | 0.18 | 0.60 | 0.18 | 0.59 | - | - | 0.33 | 1.11 | - | - | 0.06 | 0.20 |
| TB (MDR) | CAPIa | - | - | 223 | 742 | 22652 | 75508 | 4528 | 15094 | - | - | - | - |
|  | CAPIb | - | - | 216 | 720 | 5013 | 16709 | - | - | - | - | - | - |
|  | GEN1 | - | - | - | - | 140 | 466 | - | - | - | - | - | - |
|  | GEN1a | - | - | 389 | 1296 | - | - | - | - | - | - | - | - |
|  | GEN2 | - | - | 153 | 511 | 317 | 1056 | - | - | - | - | - | - |
|  | KAN | 439 | 1462 | 456 | 1519 | 693 | 2311 | - | - | - | - | - | - |
|  | STR | 238 | 794 | 248 | 828 | 518 | 1725 | 2650 | 8835 | - | - | - | - |
|  | DCS | - | - | - | - | - | - | - | - | - | - | - | - |
|  |  |  |  |  |  |  |  |  |  |  |  |  |  |
| *Table S9 continued* | | | | | | | | | | | | |  |
| **#** | **Abbrev** | **MilliQ water** | | **River water** | | **Effluent WW** | | **Influent WW** | | **Influent SPM** | | **River sediment** | |
| TB (other) | DMD | - | - | 1.49 | 4.96 | 1.03 | 3.42 | - | - | 0.35 | 1.18 | 0.44 | 1.45 |
|  | BDQ | - | - | 7.85 | 26.2 | 9.47 | 31.6 | - | - | - | - | - | - |
|  | LZD | 0.14 | 0.47 | 0.14 | 0.48 | 0.33 | 1.09 | 0.27 | 0.89 | 0.04 | 0.13 | 0.04 | 0.14 |
|  | THAL | 1.63 | 5.42 | 1.28 | 4.25 | 2.31 | 7.70 | 3.48 | 11.60 | 0.35 | 1.15 | 0.35 | 1.18 |
| **OTHER** |  |  |  |  |  |  |  |  |  |  |  |  |  |
| Amphenicol | CHL | 0.72 | 2.40 | 0.63 | 2.11 | 1.31 | 4.38 | 1.31 | 4.35 | 0.70 | 2.33 | 0.28 | 0.93 |
|  | FLO | 11.2 | 37.2 | 10.4 | 34.8 | 22.2 | 74.0 | 21.9 | 73.1 | 4.60 | 15.3 | 3.38 | 11.3 |
|  | ANP | 18.3 | 61.1 | 14.4 | 48.1 | 60.1 | 200 | 42.3 | 141 | 1.26 | 4.20 | 2.65 | 8.83 |
| Cycline | DOX | 1.95 | 6.50 | 2.56 | 8.53 | 5.38 | 17.9 | 12.7 | 42.2 | - | - | 13.0 | 43.4 |
|  | OTC | 1.72 | 5.72 | 1.82 | 6.08 | 6.64 | 22.1 | 6.35 | 21.2 | 8.00 | 26.7 | 13.7 | 45.7 |
|  | TET | 1.03 | 3.44 | 1.15 | 3.82 | 1.33 | 4.43 | 1.70 | 5.66 | 1.57 | 5.24 | - | - |
| Nitrofuran | NIT | 1.71 | 5.68 | 1.61 | 5.37 | 2.36 | 7.87 | 3.46 | 11.54 | 2.54 | 8.48 | 3.20 | 10.65 |
|  | NPAHD | 0.15 | 0.52 | 0.19 | 0.62 | 0.44 | 1.46 | 0.35 | 1.18 | 0.13 | 0.42 | 0.07 | 0.25 |
| Azole | MTZ | 0.13 | 0.44 | 0.13 | 0.43 | 0.26 | 0.88 | 0.27 | 0.91 | 0.05 | 0.16 | 0.05 | 0.15 |
|  | KTC | 0.03 | 0.09 | 0.04 | 0.14 | 0.07 | 0.24 | 0.03 | 0.10 | - | - | 0.01 | 0.04 |
|  | hMTZ | 0.75 | 2.51 | 0.73 | 2.43 | 0.50 | 1.67 | 1.01 | 3.38 | 0.19 | 0.64 | - | - |
|  | daKTC | 1.92 | 6.41 | - | - | 4.15 | 13.8 | - | - | - | - | - | - |
| ARV | FTC | 0.89 | 2.97 | 0.74 | 2.48 | 1.61 | 5.36 | 1.57 | 5.24 | 0.17 | 0.56 | 0.27 | 0.89 |
|  | 3TC | 4.13 | 13.8 | 3.56 | 11.9 | 4.22 | 14.1 | 9.51 | 31.7 | - | - | - | - |
| Grey text indicates method limits calculated from matrix recoveries of <15% | | | | | | | | | | | | | |


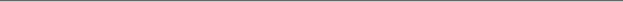


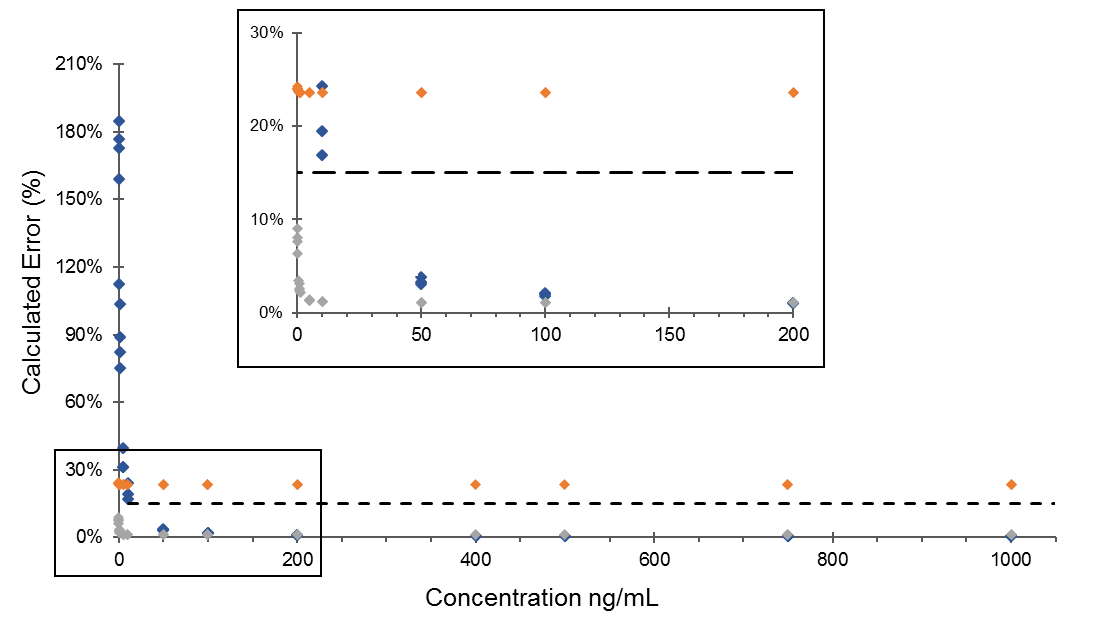


**Figure S1** Three weighting schemes evaluated via percentage error across the full range, an example using azithromycin. No weighting **x** (blue); weighting **1/x** (grey); weighting **1/x^2^** (orange); 15 % maximum error is marked as the FDA's guideline for validation of bioanalytical methods. The origin has been enlarged for clarity.


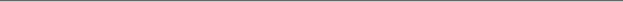


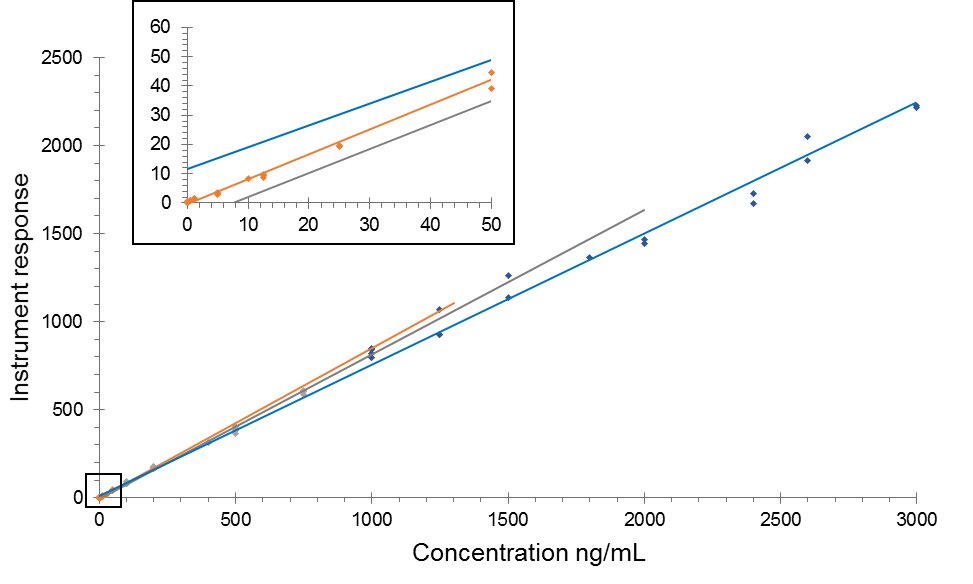


**Figure S2** Example of split calibration ranges with an emphasis on accuracy near the origin (region enlarged), an example using erythromycin. First range (0.005-200 µg L^-1^, r^2^ 0.998, orange); second range (200-1000 µg L^-1^, r^2^ 0.995, grey); third range (1000-3000 µg L^-1^, r^2^ 0.996, blue). Trend lines were extrapolated forwards to exhibit the decrease in gradient as the concentration approached instrument saturation.


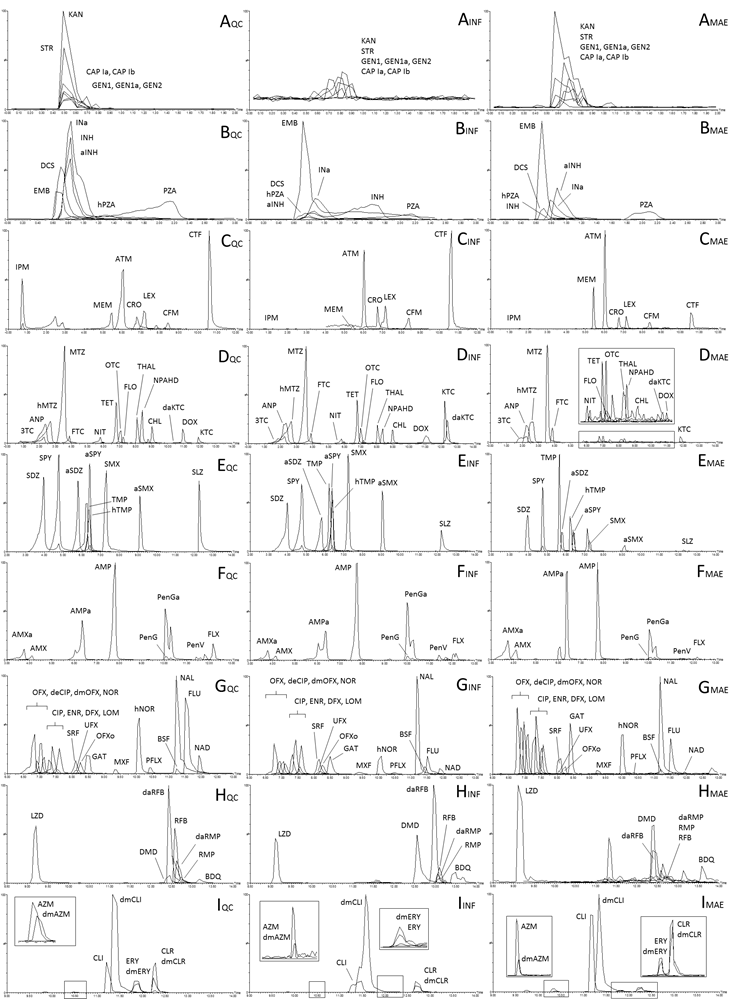


**Figure S3** Overlaid chromatograms, scaled by relative intensity, highlighting regions by chemical class (A-I). Vertically: overlaid extracted ion chromatograms (EICs), sorted by chemical class. Aminoglycosides (A); TB drugs early-eluting (B); cephalosporins and carbapenems (C); ARVs, azoles, nitrofurans, cyclines, amphenicols (D); sulfonamides (E); penicillins (F), quinolones (G); TB drugs late-eluting (H); macrolides and lincomycins (I). Horizontally: matrix effect of spiked wastewater influent (INF) and suspended particulate matter via microwave assisted extraction (MAE); compared to a mobile phase quality control (QC). The EICs displayed have not been smoothed, contrary to the method.


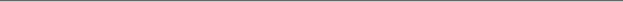


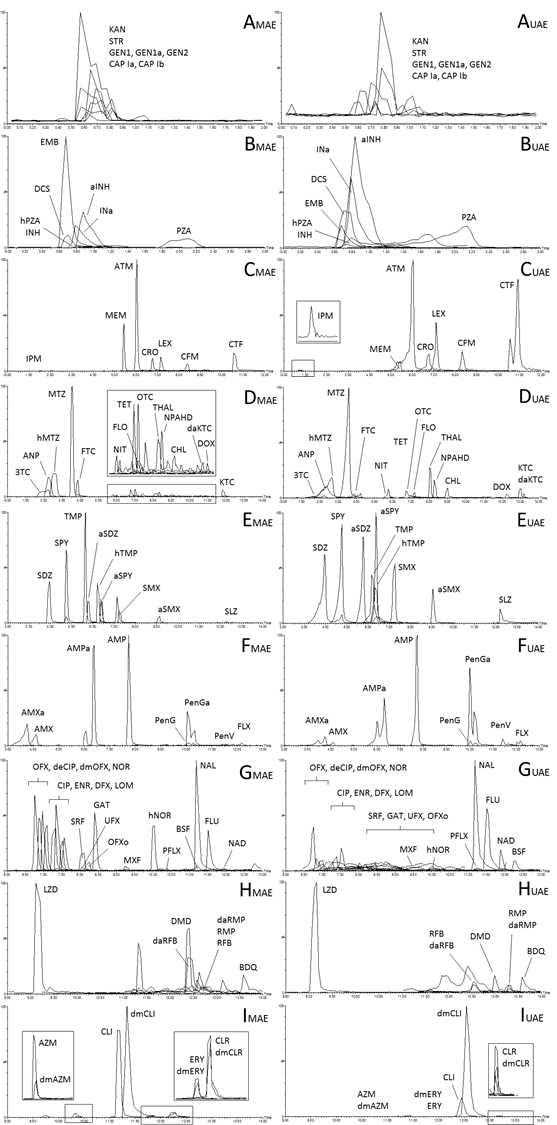


**Figure S4** Overlaid chromatograms, scaled by relative intensity, highlighting regions by chemical class (A-I). Vertically: overlaid extracted ion chromatograms (EICs), sorted by chemical class. Aminoglycosides (A); TB drugs early-eluting (B); cephalosporins and carbapenems (C); ARVs, azoles, nitrofurans, cyclines, amphenicols (D); sulfonamides (E); penicillins (F); fluoroquinolones (G); TB drugs late-eluting (H); macrolides and lincomycins (I). Horizontally: extraction method efficacy for activated sludge, comparing microwave assisted extraction (MAE), and ultra-sonication assisted extraction (UAE). The EICs displayed have not been smoothed, contrary to the method.

| Table S10 Compound structural information, grouped by drug class and corresponding drug metabolites (9 pages) | | | | | | | |  |
| --- | --- | --- | --- | --- | --- | --- | --- | --- |
| Chemical group | **Compound** | **Formula** | **Monoisotopic mass** | **R_1_** | **R_2_** | **R_3_** | **R_4_** | |
| PENICILLINS | Amoxicillin | C_16_H_19_N_3_O_5_S | 365.1045 | phenol | NH_2_ |  |  | |
| 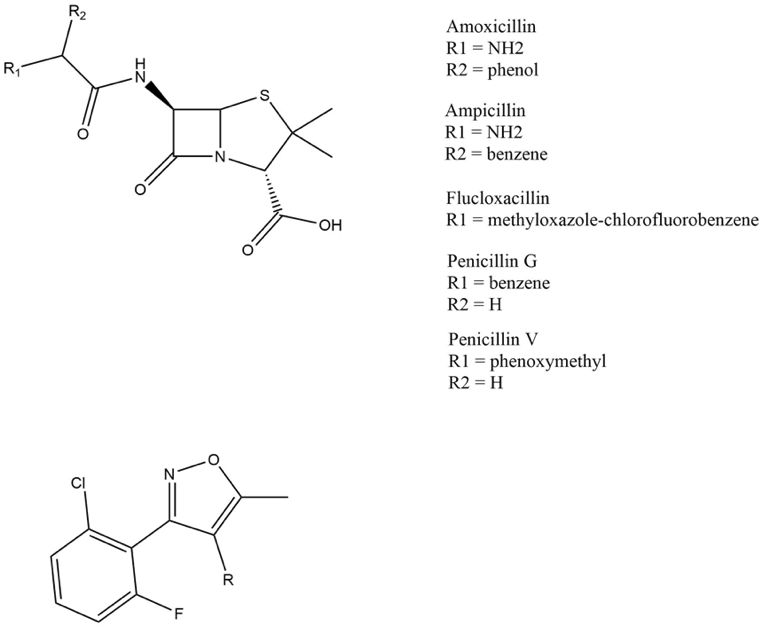 | Ampicillin | C_16_H_19_N_3_O_4_S | 349.1096 | phenyl | NH_2_ |  |  | |
|  | Flucloxacillin | C_19_H_17_ClFN_3_O_5_S | 453.0561 | 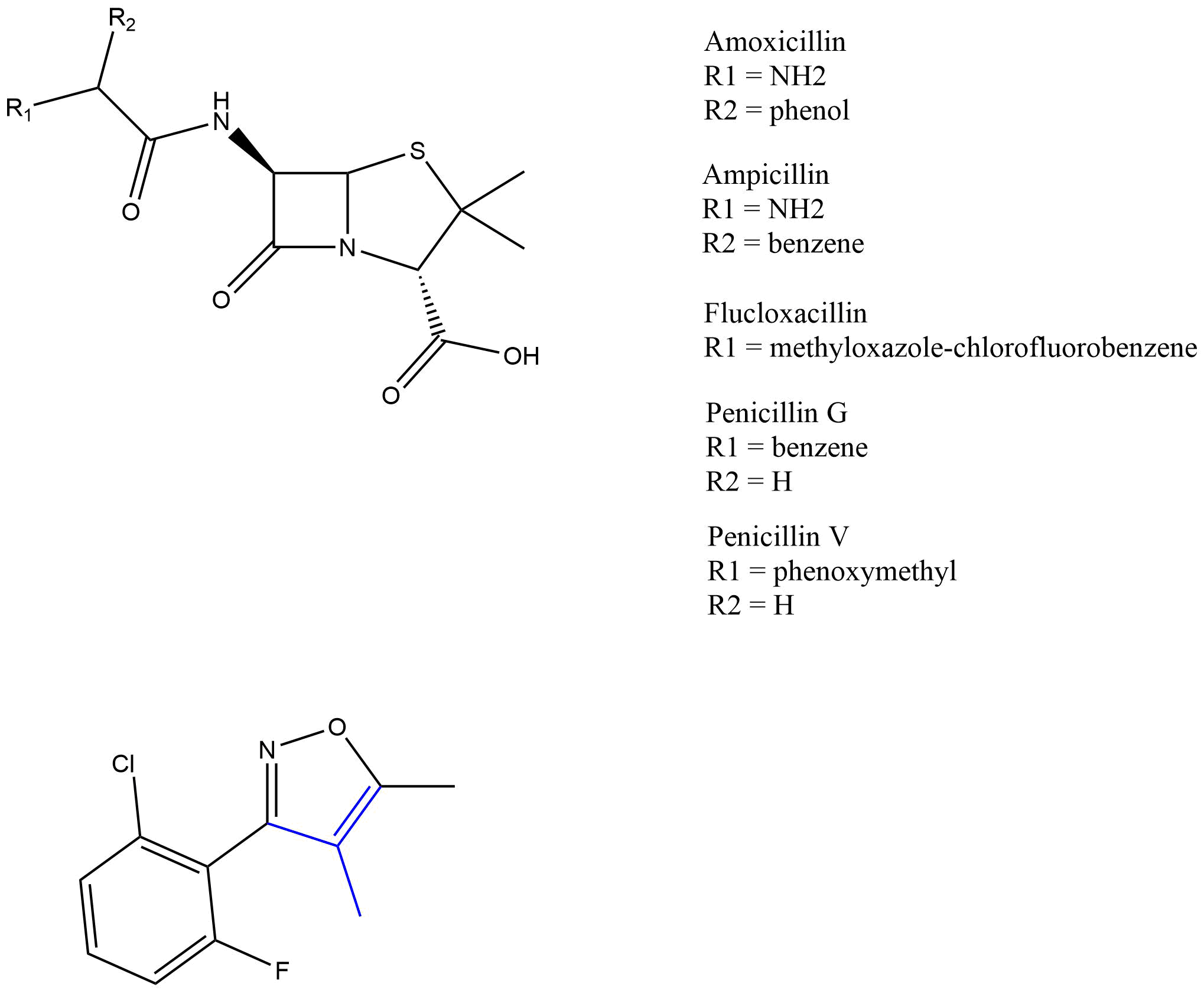 | [See R_1_] |  |  | |
|  | Penicillin G | C_16_H_18_N_2_O_4_S | 334.0987 | phenyl | H |  |  | |
|  | Penicillin V | C_16_H_18_N_2_O_5_S | 350.0936 | phenoxymethyl | H |  |  | |
| PENICILLIN METABOLITES  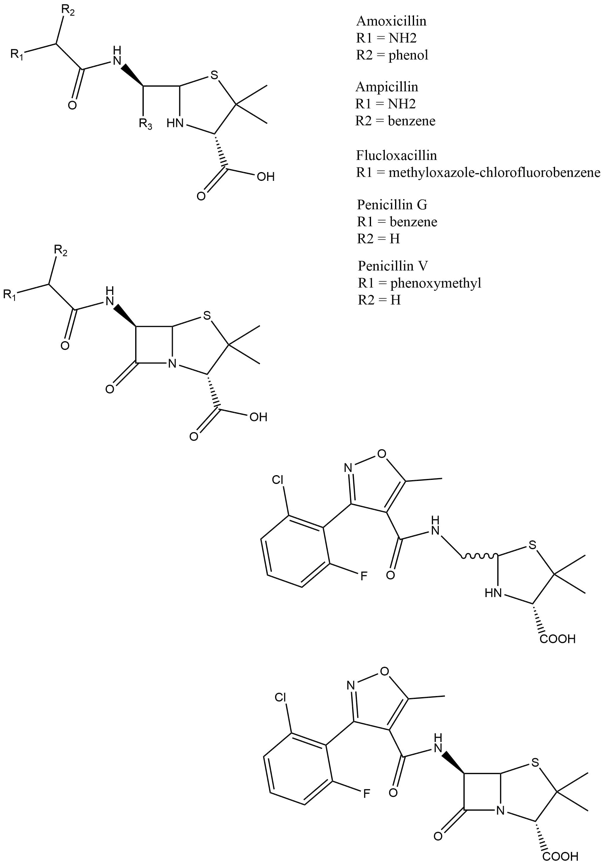 | Amoxicilloic acid | C_16_H_21_N_3_O_6_S | 383.1151 | *As amoxicillin* | *As amoxicillin* | COOH |  | |
|  | Ampicilloic acid | C_16_H_21_N_3_O_5_S | 367.1202 | *As ampicillin* | *As ampicillin* | COOH |  | |
|  | Penicilloic G acid | C_16_H_20_N_2_O_5_S | 352.1093 | *As penicillin G* | *As penicillin G* | COOH |  | |
| CEPHALOSPORINS | Cefaclor ≠ | C_15_H_14_ClN_3_O_4_S | 367.0394 | amine | phenyl | H | Cl | |
| 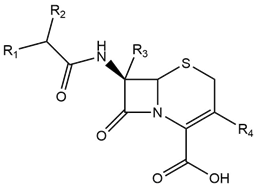 | Cefalexin | C_16_H_17_N_3_O_4_S | 347.0940 | amine | phenyl | H | CH_3_ | |
|  | Cefixime | C_16_H_15_N_5_O_7_S_2_ | 453.0413 | aminothiazole | 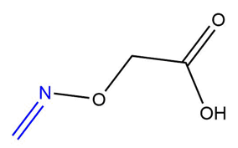 | H | ethane | |
|  | Cefoxitin ≠ | C_16_H_17_N_3_O_7_S_2_ | 427.0508 | aminothiazole | H | methoxy | methylcarbamate | |
|  | Ceftazidime ≠ | C_22_H_22_N_6_O_7_S_2_ | 546.0991 | aminothiazole | 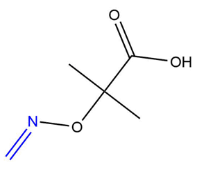 | H | CH_3_-pyridine | |
|  | Ceftiofur | ‎C_19_H_17_N_5_O_7_S_3_ | 523.0290 | aminothiazole | 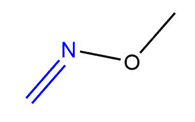 | H | 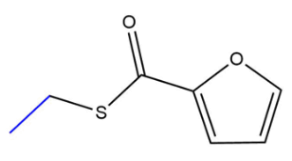 | |
|  | Ceftriaxone | C_18_H_18_N_8_O_7_S_3_ | 554.0461 | aminothiazole | 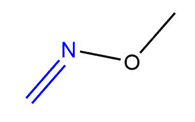 | H | 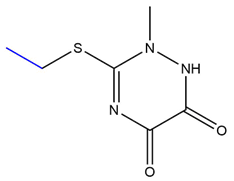 | |

| Chemical group | Compound | Formula | Monoisotopic mass | R_1_ | R_2_ | R_3_ | R_4_ |
| --- | --- | --- | --- | --- | --- | --- | --- |
| ISONIAZID & METABOLITES  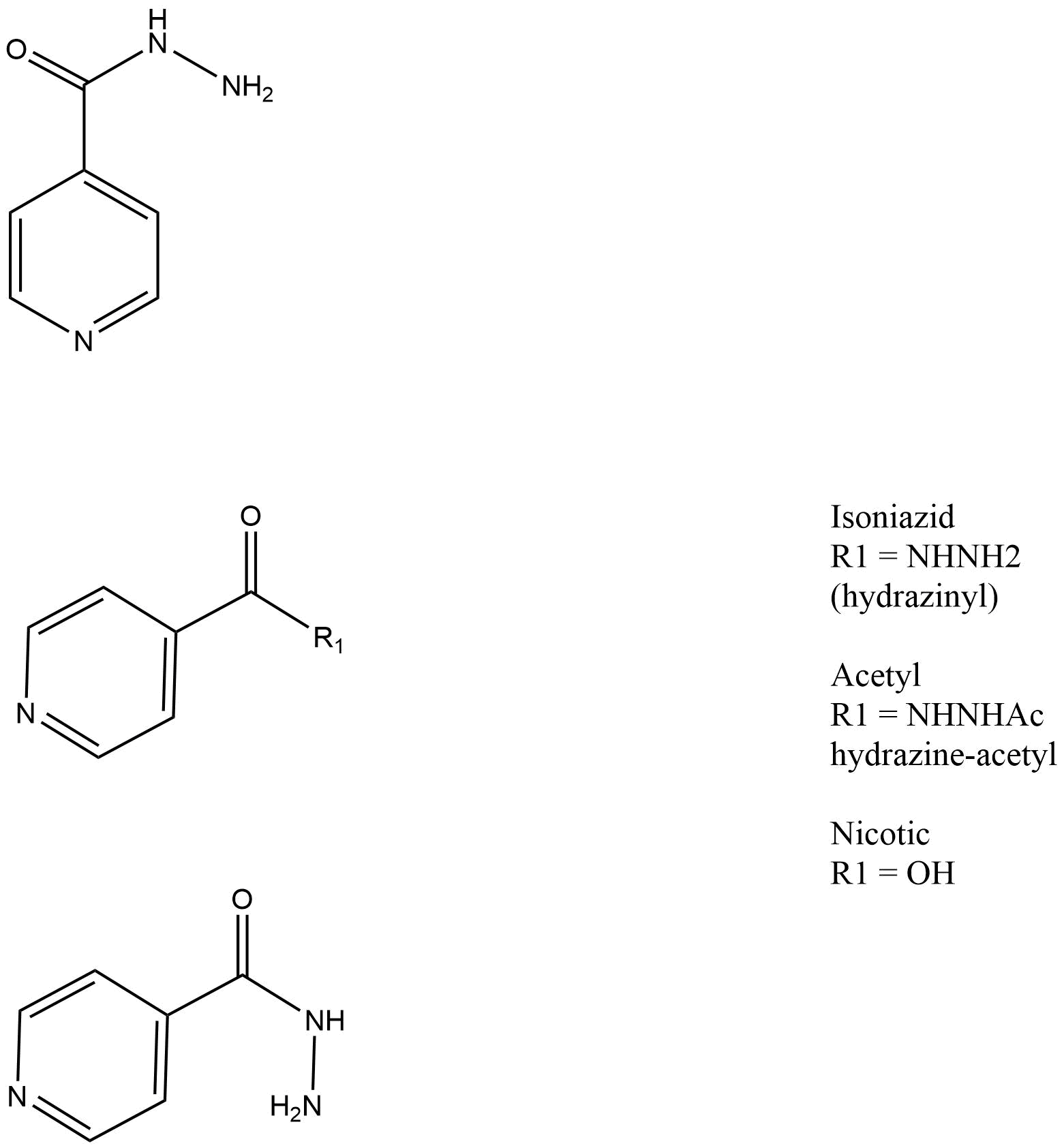 | Isoniazid | C_6_H_7_N_3_O | 137.0589 | hydrazine |  |  |  |
|  | Isoniazid acetyl | C_8_H_9_N_3_O_2_ | 179.0695 | hydrazine-acetyl |  |  |  |
|  | Isonicotinic acid | C_6_H_5_NO_2_ | 123.0320 | OH |  |  |  |
| PYRAZINAMIDE & METABOLITES  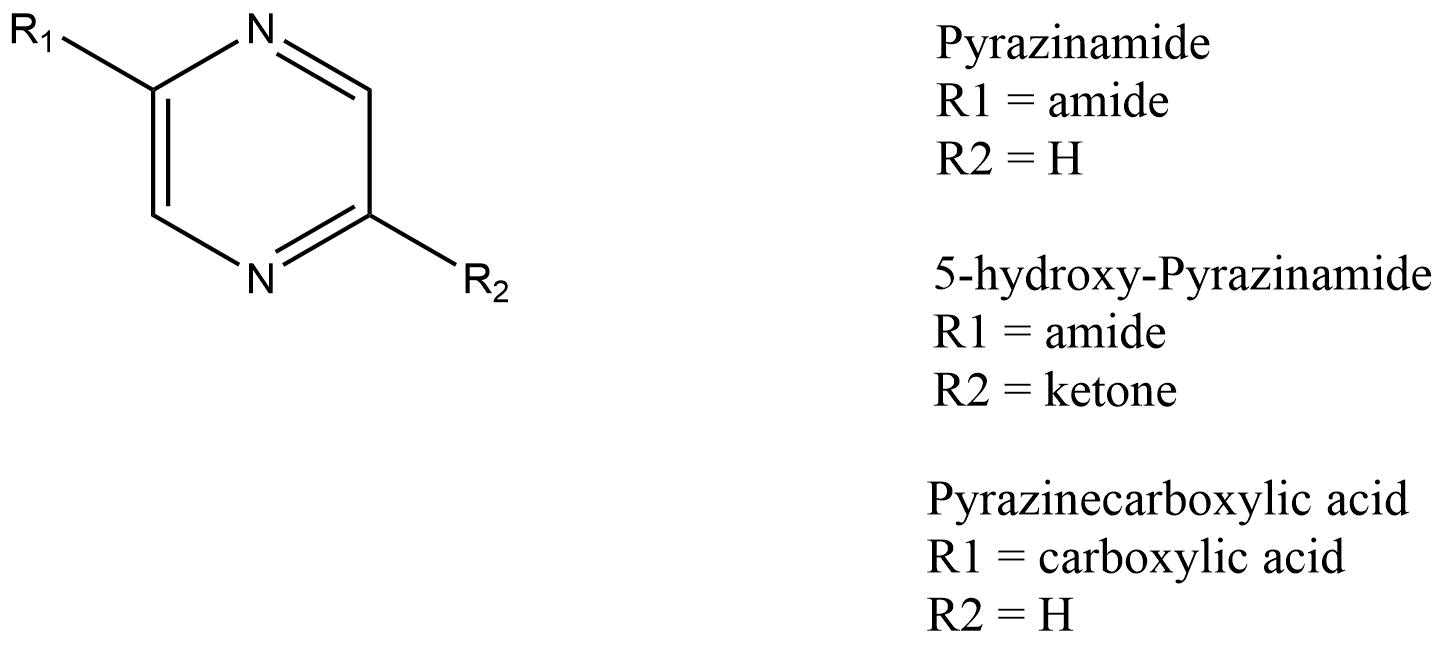 | Pyrazinamide | C_5_H_5_N_3_O | 123.0433 | amide | H |  | **Prodrug** |
|  | Pyrazinoic acid ≠ | C_5_H_4_N_2_O_2_ | 124.0273 | amide | ketone |  | **Active metabolite** |
|  | Pyrazinoic acid  5-hydroxy | C_5_H_4_N_2_O_3_ | 140.0222 | carboxylic acid | H |  |  |
| RIFAMYCINS  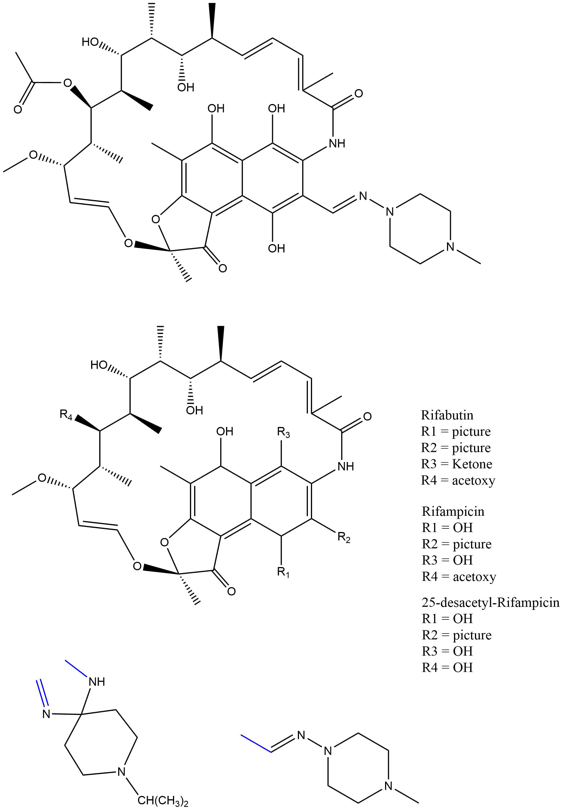 | Rifabutin | C_46_H_62_N_4_O_11_ | 846.4415 | 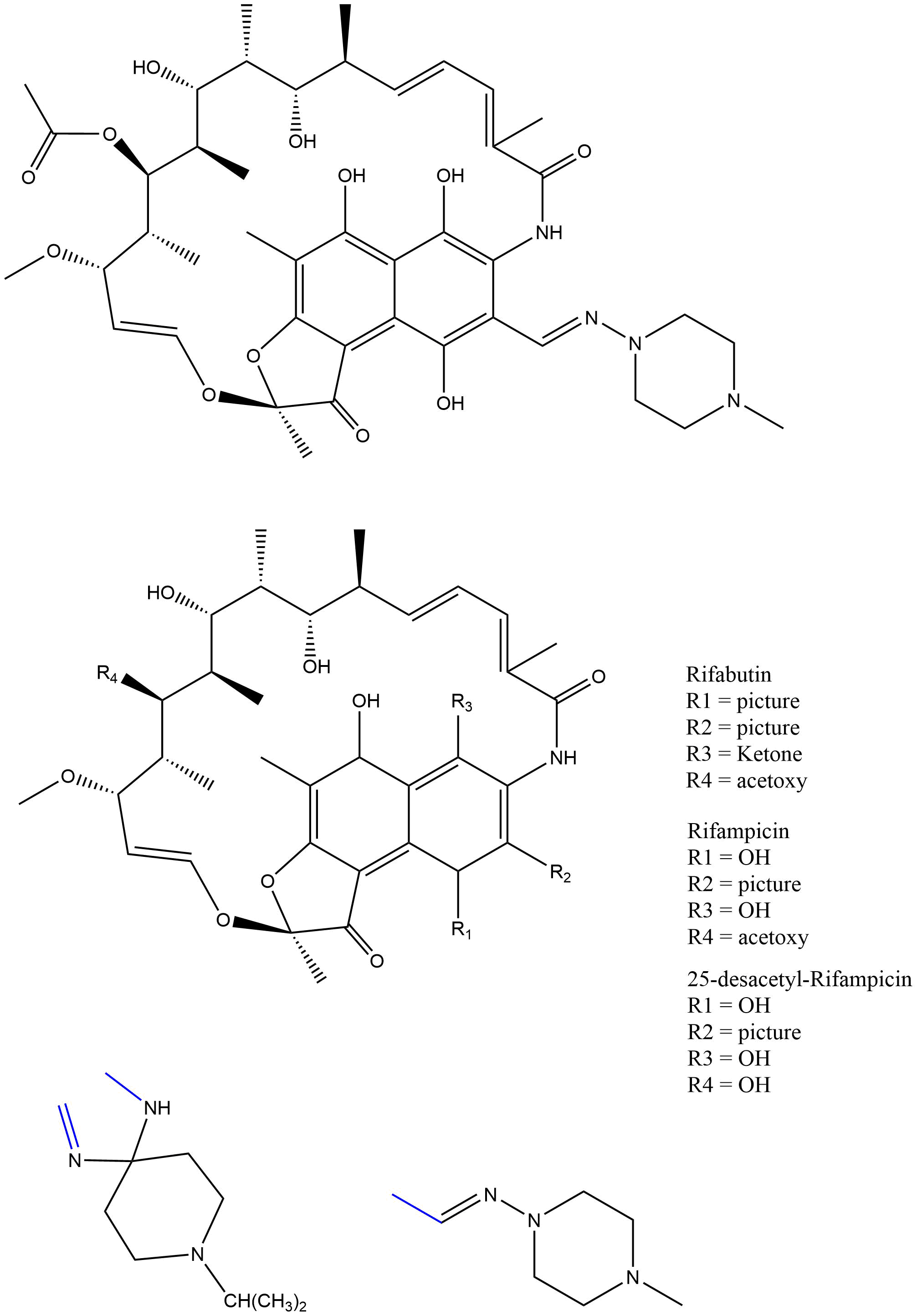 | [See R_1_] | ketone | acetoxy |
|  | Rifampicin | C_43_H_58_N_4_O_12_ | 822.4051 | OH | 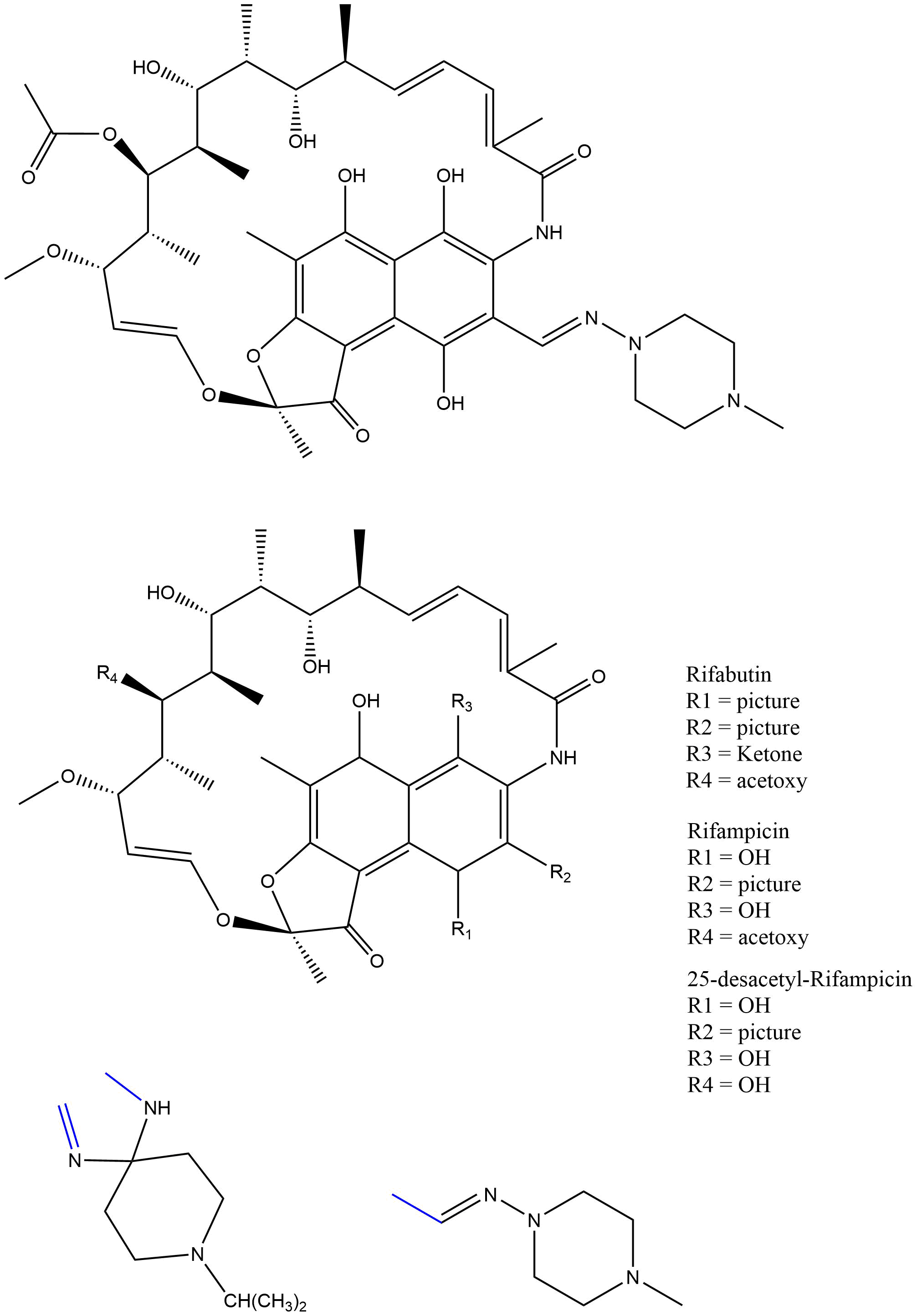 | OH | acetoxy |
| RIFAMYCIN METABOLITES | Rifabutin 25-O-desacetyl | C_44_H_60_N_4_O_10_ | 804.4309 | *As rifabutin* | *As rifabutin* | *As rifabutin* | methoxy |
|  | Rifampicin 25-desacetyl | C_41_H_56_N_4_O_11_ | 780.3946 | *As rifampicin* | *As rifampicin* | *As rifampicin* | methoxy |
| TERIZIDONE & METABOLITE  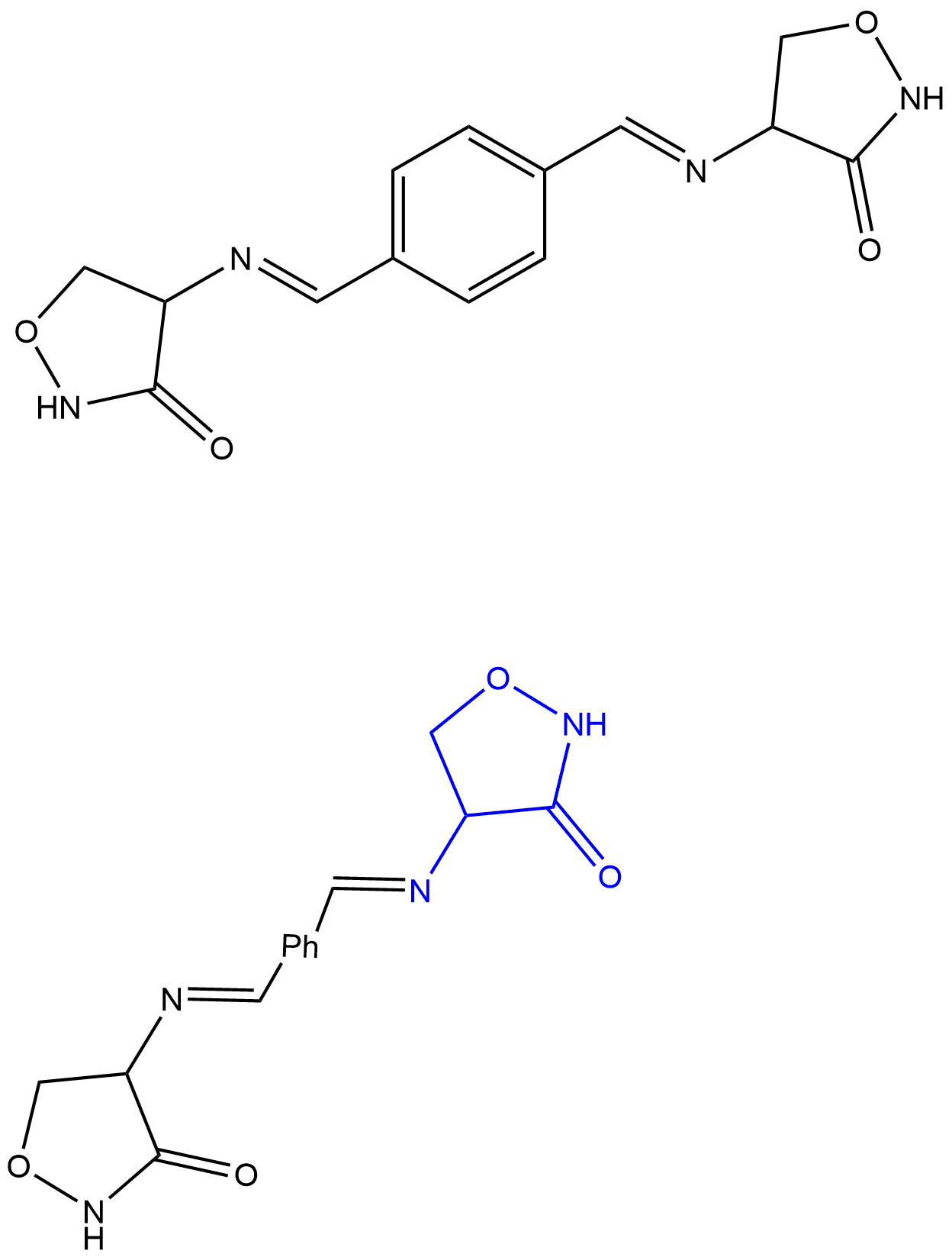 | Terizidone ≠ | C_14_H_14_N_4_O_4_ | 302.1015 | Whole structure  (Ph = 1,4-phenyl) |  | **Prodrug** |  |
|  | Cycloserine | C_3_H_6_N_2_O_2_ | 102.0429 | Amino isoxazolidinone group  (blue) |  | **Active metabolite** |  |

| Chemical group | Compound | Formula | | | Monoisotopic mass | | | R_1_ | | | R_2_ | | R_3_ | | |  | | |  |  |  |
| --- | --- | --- | --- | --- | --- | --- | --- | --- | --- | --- | --- | --- | --- | --- | --- | --- | --- | --- | --- | --- | --- |
| IMIDAZOLES  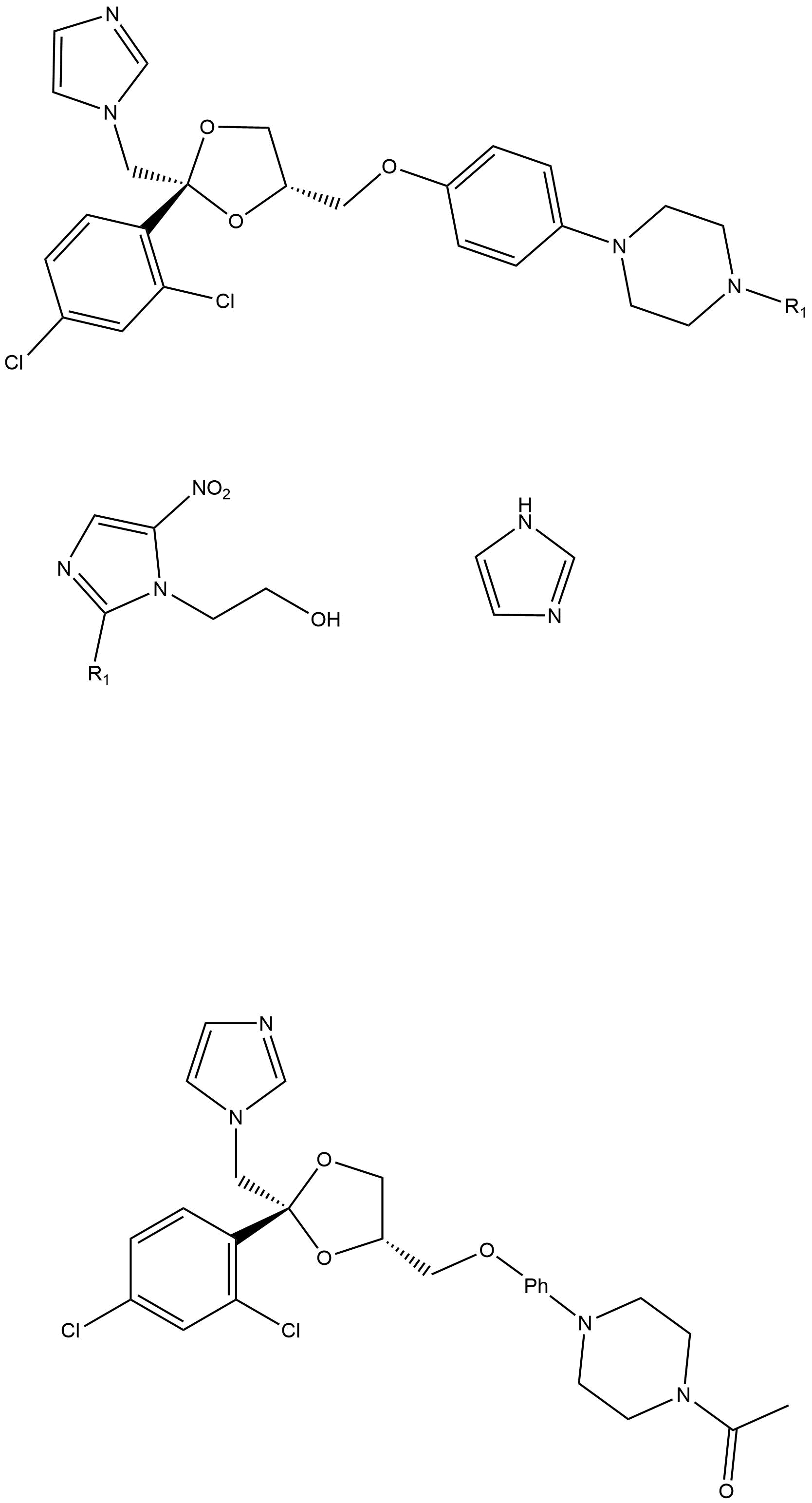 | Ketoconazole | | C_26_H_28_Cl_2_N_4_O_4_ | 530.1488 | | | | | 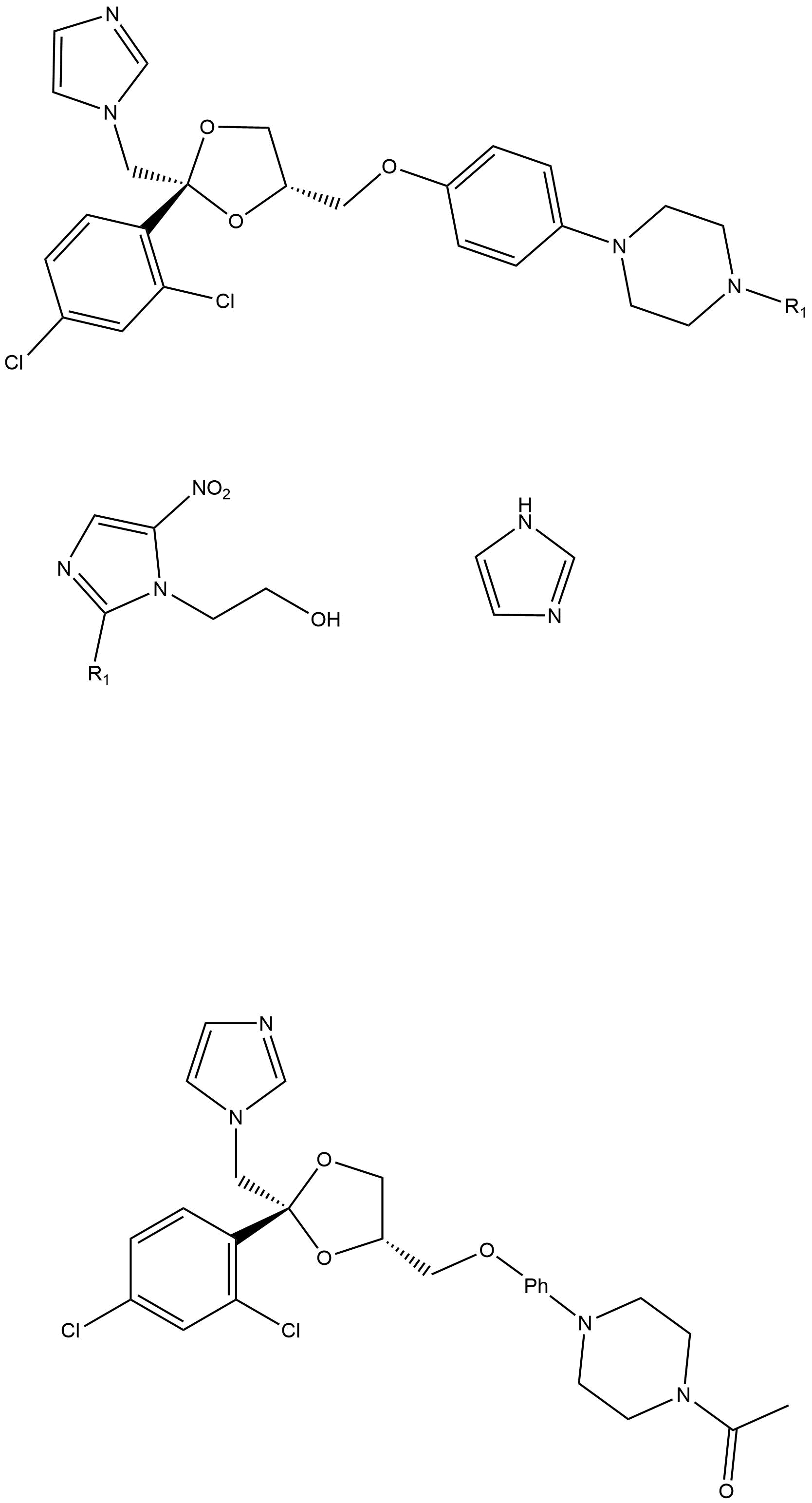 | | | | | R_1_ = acetyl |  | |  | | | | |
|  | Metronidazole | | C_6_H_9_N_3_O_3_ | 171.0644 | | | | | 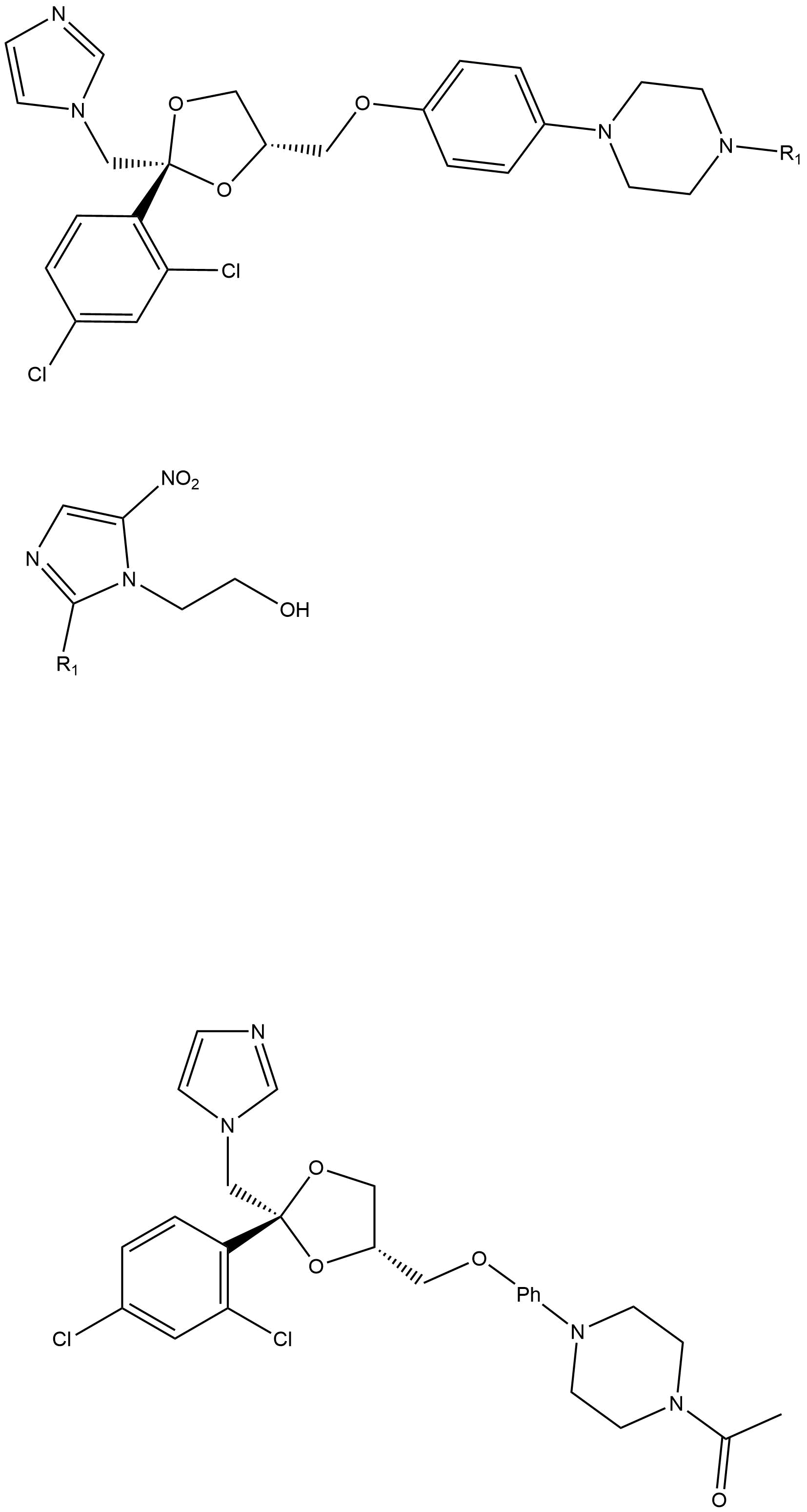 | | | | | R_1_ = CH_3_ |  | |  | | | | |
| IMIDAZOLE METABOLITES | Ketoconazole  deacetyl | | C_24_H_26_Cl_2_N_4_O_3_ | 488.1382 | | | | | *As ketoconazole* | | | | | R_1_ = H |  | |  | | | | |
|  | Metronidazole hydroxy | | C_6_H_9_N_3_O_4_ | 187.0593 | | | | | *As metronidazole* | | | | | R_1_ = ethanol |  | |  | | | | |
| TETRACYCLINES | Doxycycline | C_22_H_24_N_2_O_8_ | | | | 444.1533 | H | | | CH_3_ | | OH | | | |  | | | | |  |
| 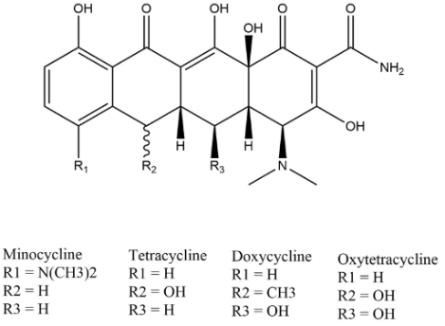 | Minocycline ≠ | C_23_H_27_N_3_O_7_ | | | | 457.1849 | N(CH_3_)_2_ | | | H | | H | | | |  | | | | |  |
|  | Oxytetracycline | C_22_H_24_N_2_O_9_ | | | | 460.1482 | H | | | OH | | OH | | | |  | | | | |  |
|  | Tetracycline | C_22_H_24_N_2_O_8_ | | | | 444.1533 | H | | | OH | | H | | | |  | | | | |  |
| NITROFURANTOIN | Nitrofurantoin | C_8_H_6_N_4_O_5_ | | | | 238.0338 | 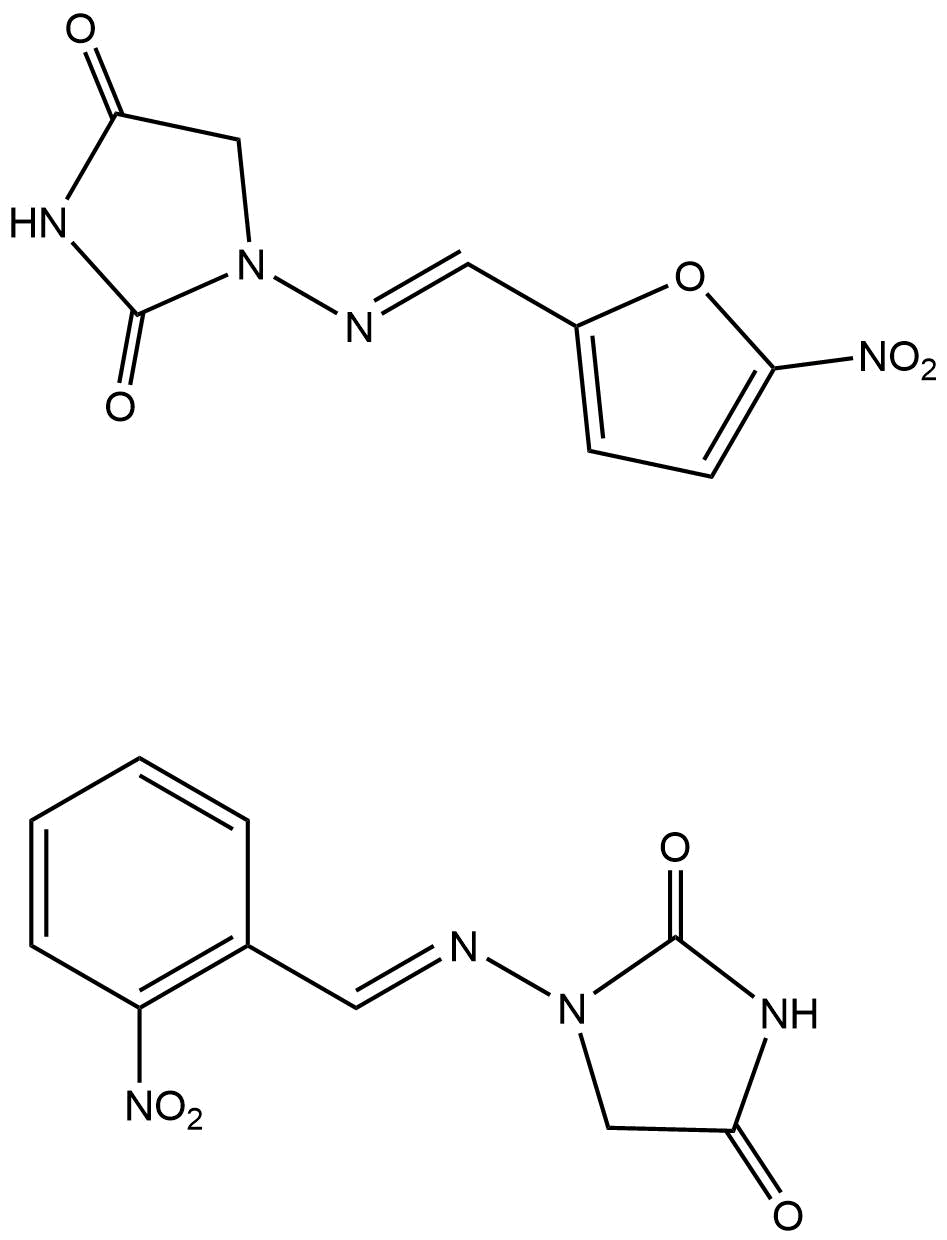 | | | | |  | | | |  | | | | |  |
| NITROFURANTOIN METABOLITE | NP-AHD | C_10_H_8_N_4_O_4_ | | | | 248.0546 | 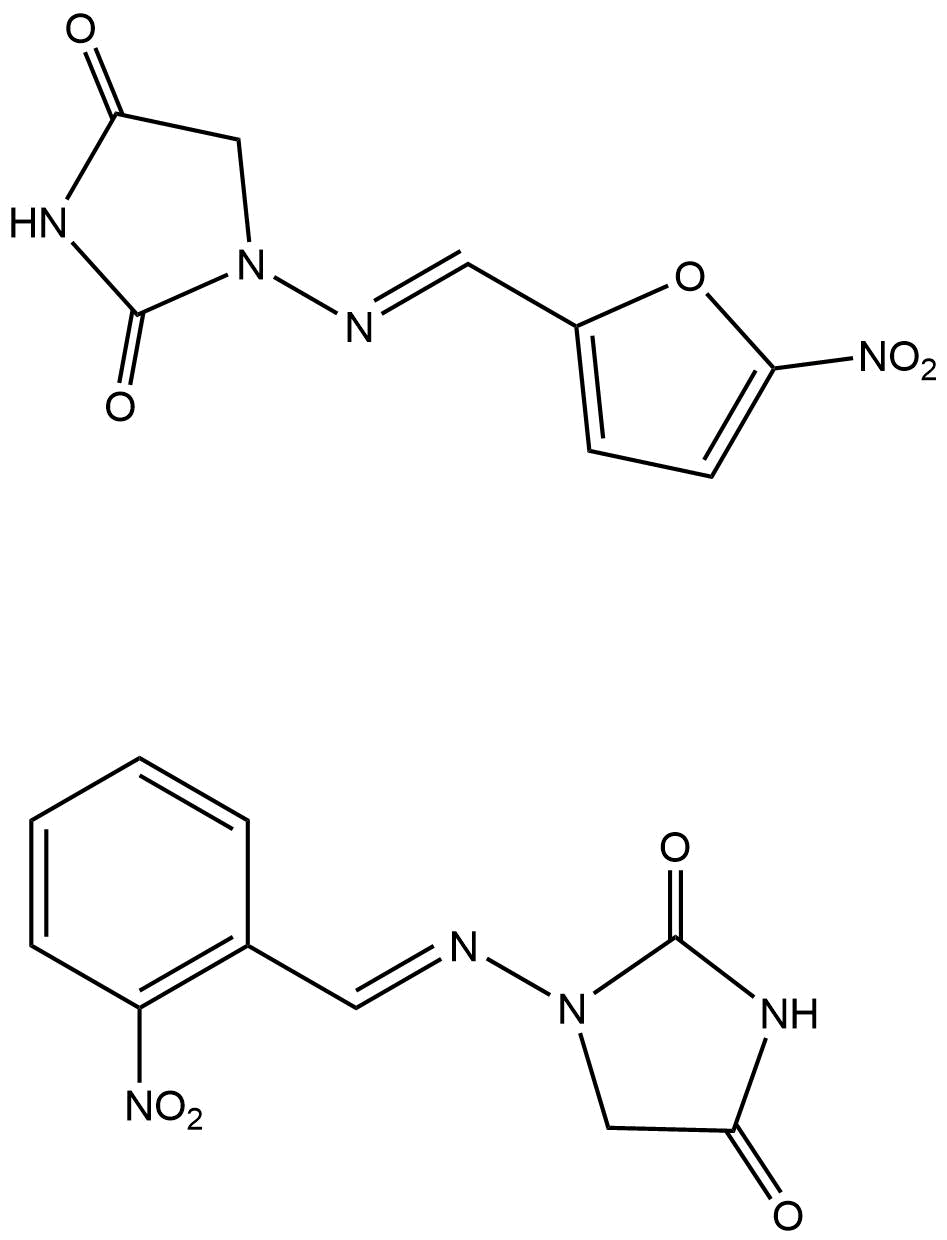 | | | | |  | | | |  | | | | |  |
|  |  |  | | |  | | |  | | |  | |  | | |  | | |  |  |  |
|  |  |  | | |  | | |  | | |  | |  | | |  | | |  |  |  |
|  |  |  | | |  | | |  | | |  | |  | | |  | | |  |  |  |
|  |  |  | | |  | | |  | | |  | |  | | |  | | |  |  |  |
|  |  |  | | |  | | |  | | |  | |  | | |  | | |  |  |  |
| Chemical group | **Compound** | **Formula** | | | **Monoisotopic mass** | | | **R_1_** | | | **R_2_** | | **R_3_** | | |  | | |  |  |  |
| FLUOROQUINOLONES | Besifloxacin | C_19_H_21_ClFN_3_O_3_ | | | 393.1255 | | | cyclopropane | | | Cl | | aminoazepane | | |  | | |  |  |  |
| 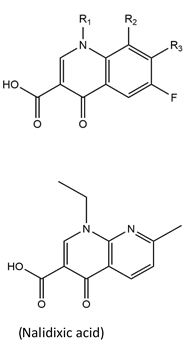 | Ciprofloxacin | C_17_H_18_FN_3_O_3_ | | | 331.1332 | | | cyclopropane | | | H | | piperazine | | |  | |  |  |  |  |
|  | Danofloxacin | C_19_H_20_FN_3_O_3_ | | | 357.1489 | | | cyclopropane | | | H | | 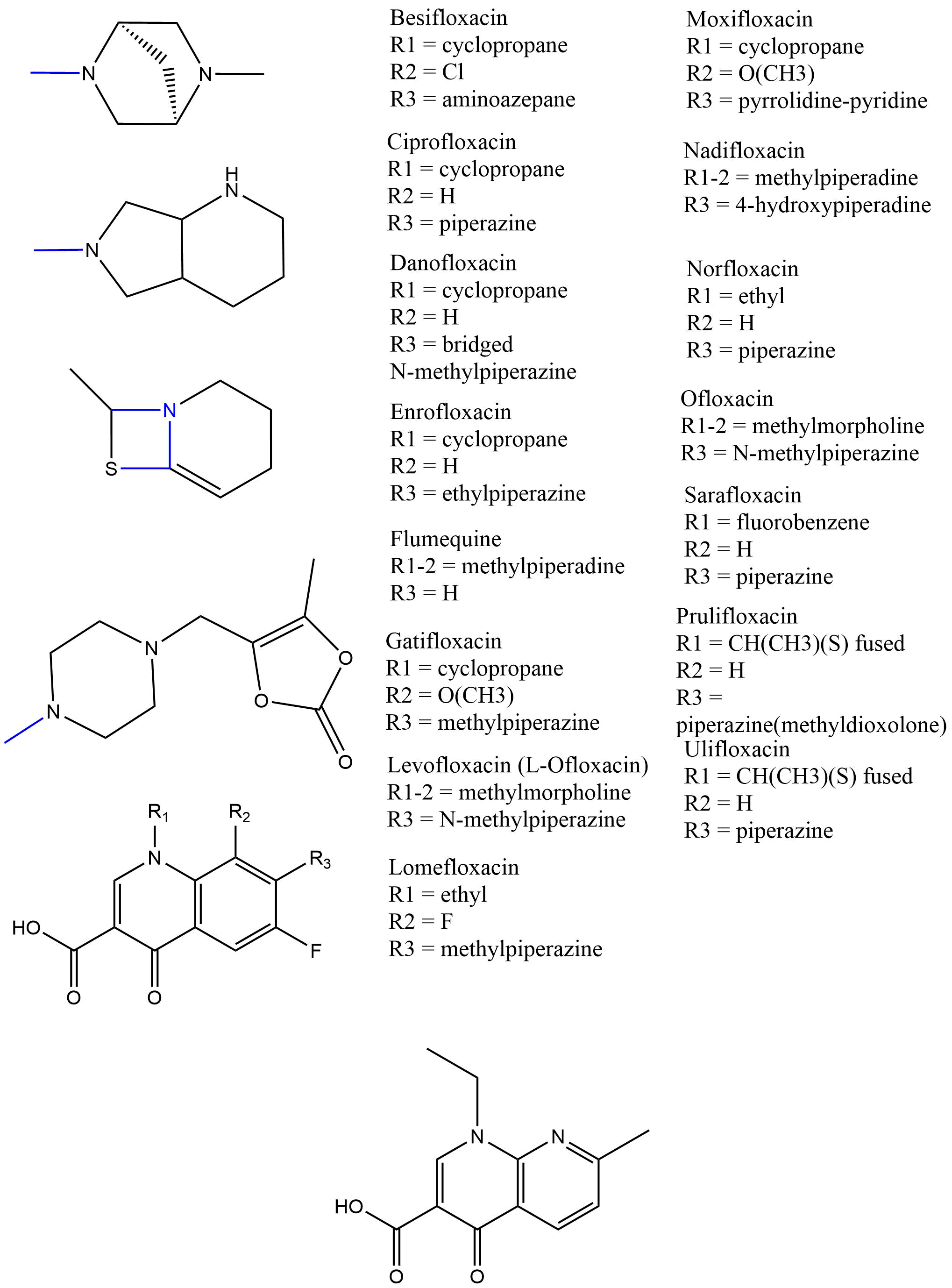 | | |  | | |  |  |  |
|  | Enrofloxacin | C_19_H_22_FN_3_O_3_ | | | 359.1645 | | | cyclopropane | | | H | | ethylpiperazine | | |  | | |  |  |  |
|  | Flumequine | C_14_H_12_FNO_3_ | | | 261.0801 | | | methylpiperadine | | | [See R_1_] | | H | | |  | | |  |  |  |
|  | Gatifloxacin | C_19_H_22_FN_3_O_4_ | | | 375.1594 | | | cyclopropane | | | methoxy | | methylpiperazine | | |  | | |  |  |  |
|  | Lomefloxacin | C17H19F2N3O3 | | | 351.1394 | | | ethyl | | | F | | methylpiperazine | | |  | | |  |  |  |
|  | Moxifloxacin | C_21_H_24_FN_3_O_4_ | | | 401.1751 | | | cyclopropane | | | methoxy | | 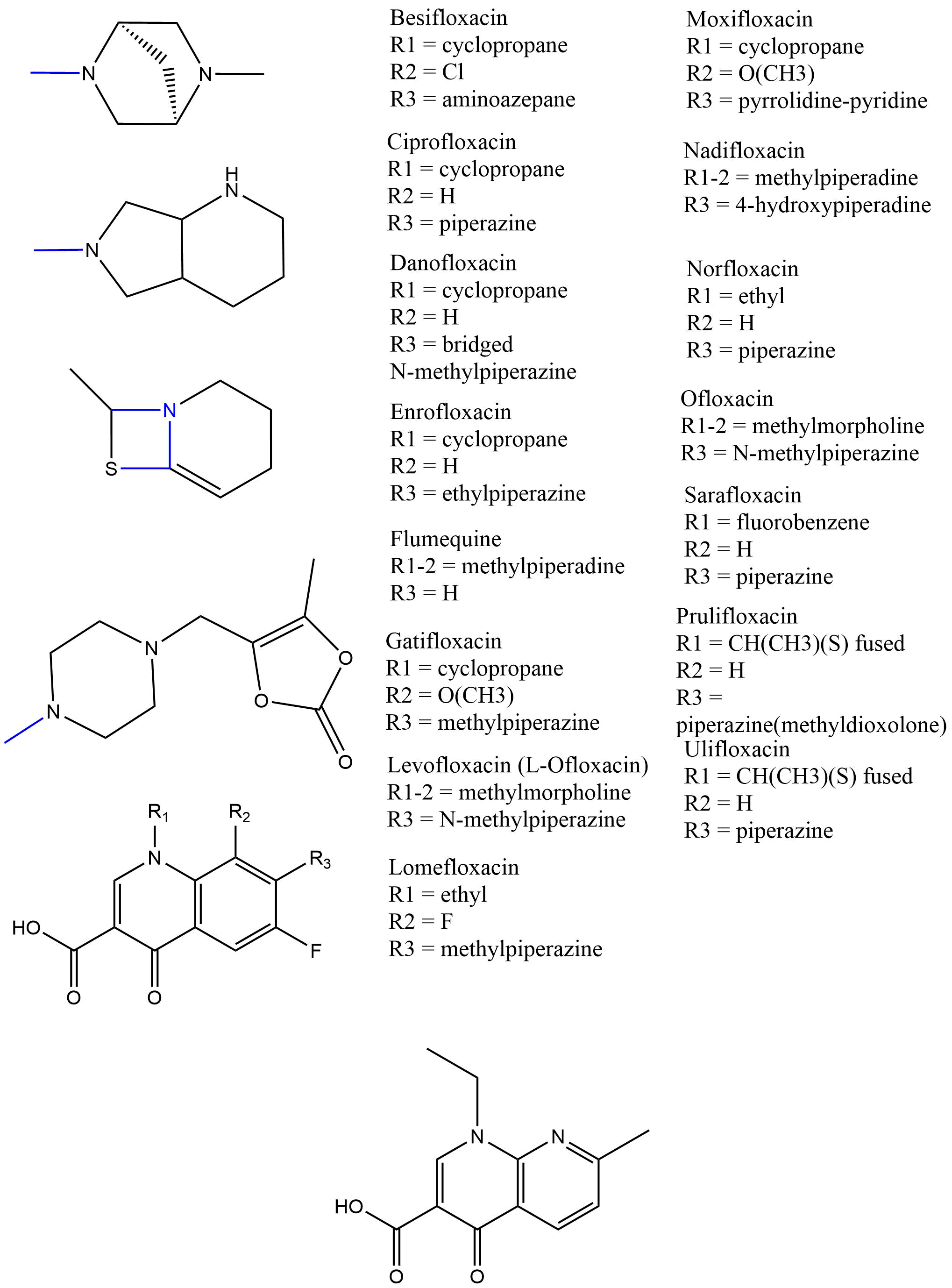 | | |  | | |  |  |  |
|  | Nadifloxacin | C_19_H_21_FN_2_O_4_ | | | 360.1485 | | | methylpiperadine | | | [See R_1_] | | 4-hydroxypiperadine | | |  | | |  |  |  |
|  | Nalidixic acid | C_12_H_12_N_2_O_3_ | | | 232.0848 | | | *pictured (left)* | | |  | |  | | |  | | |  |  |  |
|  | Norfloxacin | C_16_H_18_FN_3_O_3_ | | | 319.1332 | | | ethyl | | | H | | Piperazine | | |  | | |  |  |  |
|  | Ofloxacin | C_18_H_20_FN_3_O_4_ | | | 361.1438 | | | methylmorpholine | | | [See R_1_] | | N-methylpiperazine | | |  | | |  |  |  |
|  | Prulifloxacin | C_21_H_20_FN_3_O_6_S | | | 461.1057 | | | 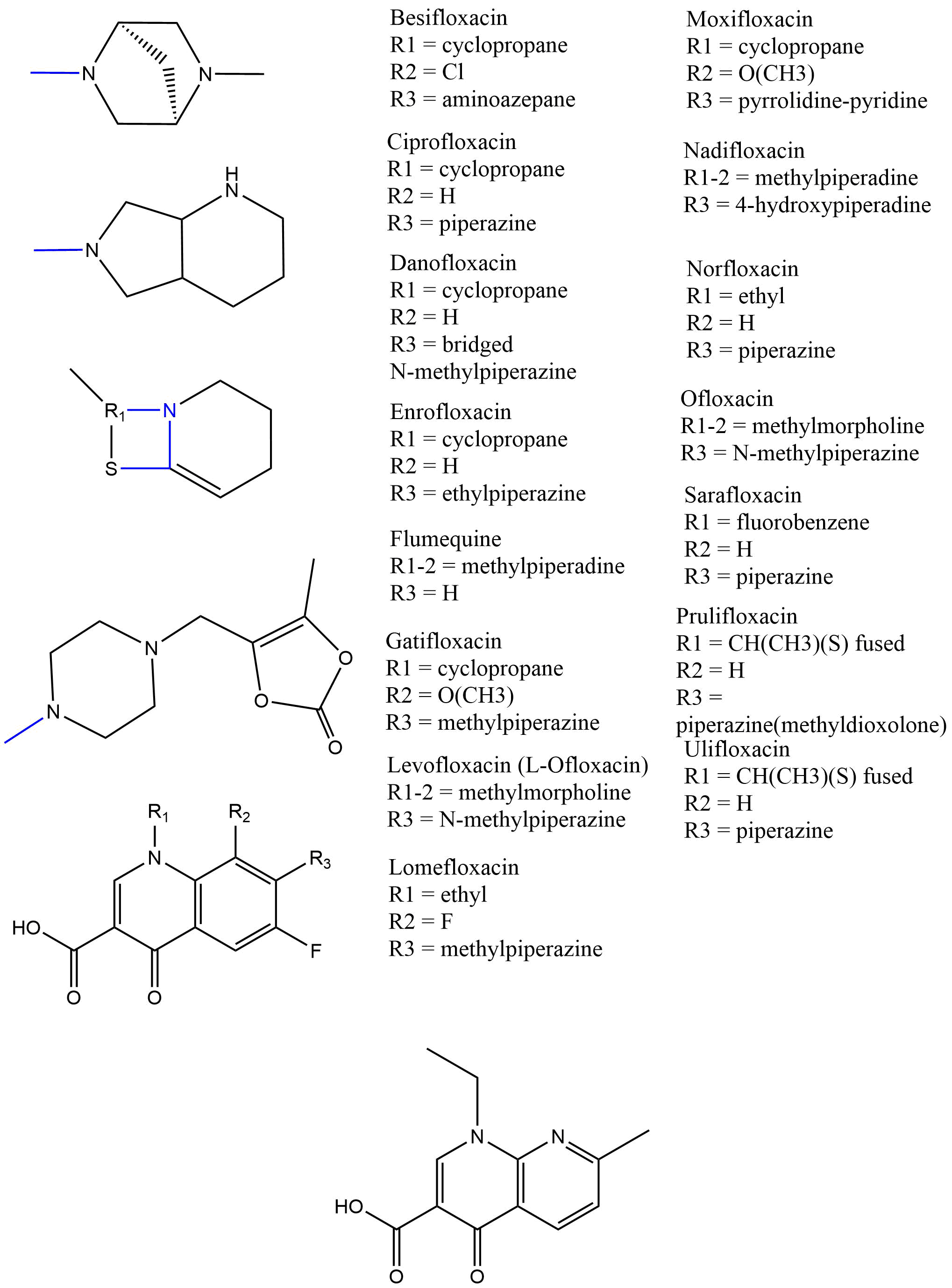 | | | H | | 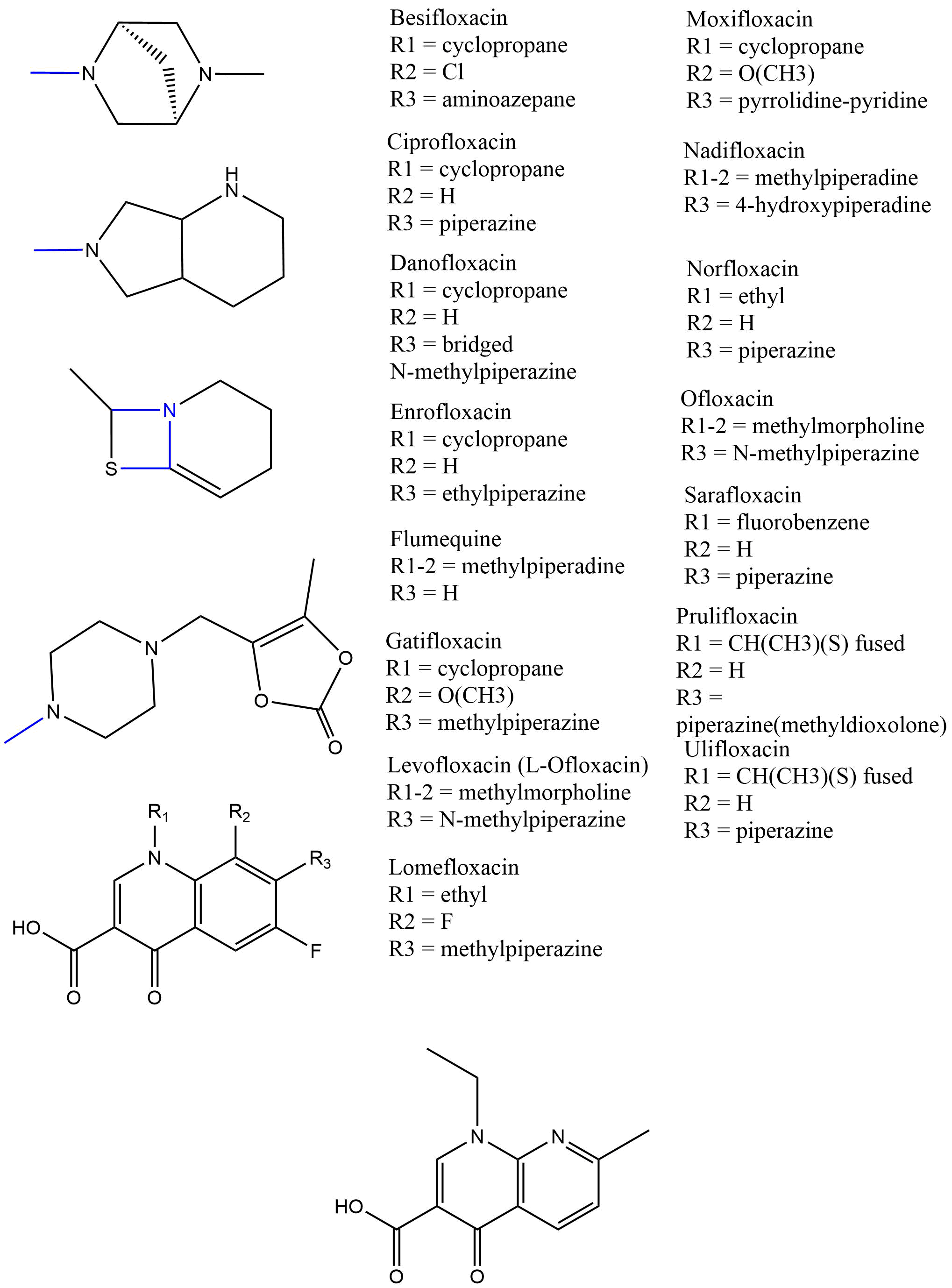 | | |  | | |  |  |  |
|  | Sarafloxacin | C_20_H_17_F_2_N_3_O_3_ | | | 385.1238 | | | fluorobenzene | | | H | | piperazine | | |  | | |  |  |  |
|  | Ulifloxacin | C_16_H_16_FN_3_O_3_S | | | 349.0896 | | | 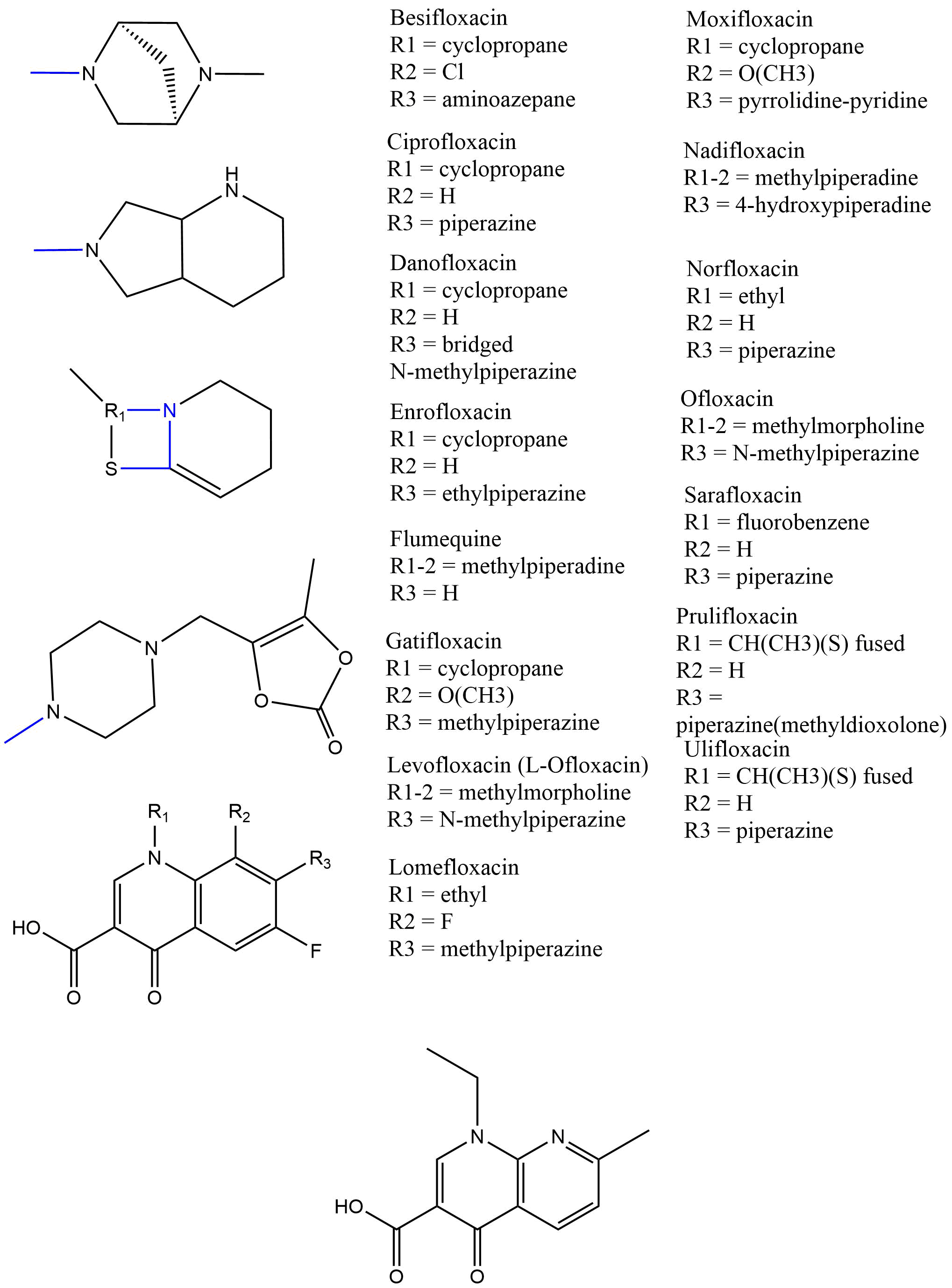 | | | H | | piperazine | | |  | | |  |  |  |
| FLUOROQUINOLONE METABOLITES | Ciprofloxacin desethylene- | C_15_H_16_FN_3_O_3_ | | | 305.1176 | | | *As ciprofloxacin* | | | *As ciprofloxacin* | | diaminoethane | | |  | | |  |  |  |
| 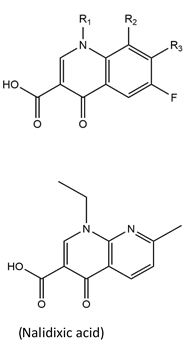 | Ciprofloxacin | C_17_H_18_FN_3_O_3_ | | | 331.1332 | | | *As above* | | | *As above* | | *As above* | | | **Enrofloxacin metabolite** | | |  |  |  |
|  | Ofloxacin desmethyl- | C_17_H_18_FN_3_O_4_ | | | 347.1281 | | | *As ofloxacin* | | | *As ofloxacin* | | piperazine | | |  | | |  |  |  |
|  | Ofloxacin N-oxide | C_18_H_20_FN_3_O_5_ | | | 377.1387 | | | N-oxide methylmorpholine | | | *As ofloxacin* | | *As ofloxacin* | | |  | | |  |  |  |
|  | Ulifloxacin | C_16_H_16_FN_3_O_3_S | | | 349.0896 | | | *As above* | | | *As above* | | *As above* | | | **Prulifloxacin metabolite** | | |  |  |  |
|  | Norfloxacin  N-hydroxy | C_16_H_18_FN_3_O_4_ | | | 335.1281 | | | *As norfloxacin* | | | *As norfloxacin* | | N-hydroxy Piperazine | | |  | | |  |  |  |
|  |  |  | | |  | | |  | | |  | |  | | |  | | |  |  |  |
| Group | **Compound** | **Formula** | | | **Monoisotopic mass** | | | **R_1_** | | | **R_2_** | | **R_3_** | | |  | | |  |  |  |
| SULFONAMIDES | Sulfadiazine | C_10_H_10_N_4_O_2_S | | | 250.0524 | | | NH_2_ | | | 1,3-diazine | |  | | |  | | |  |  |  |
| 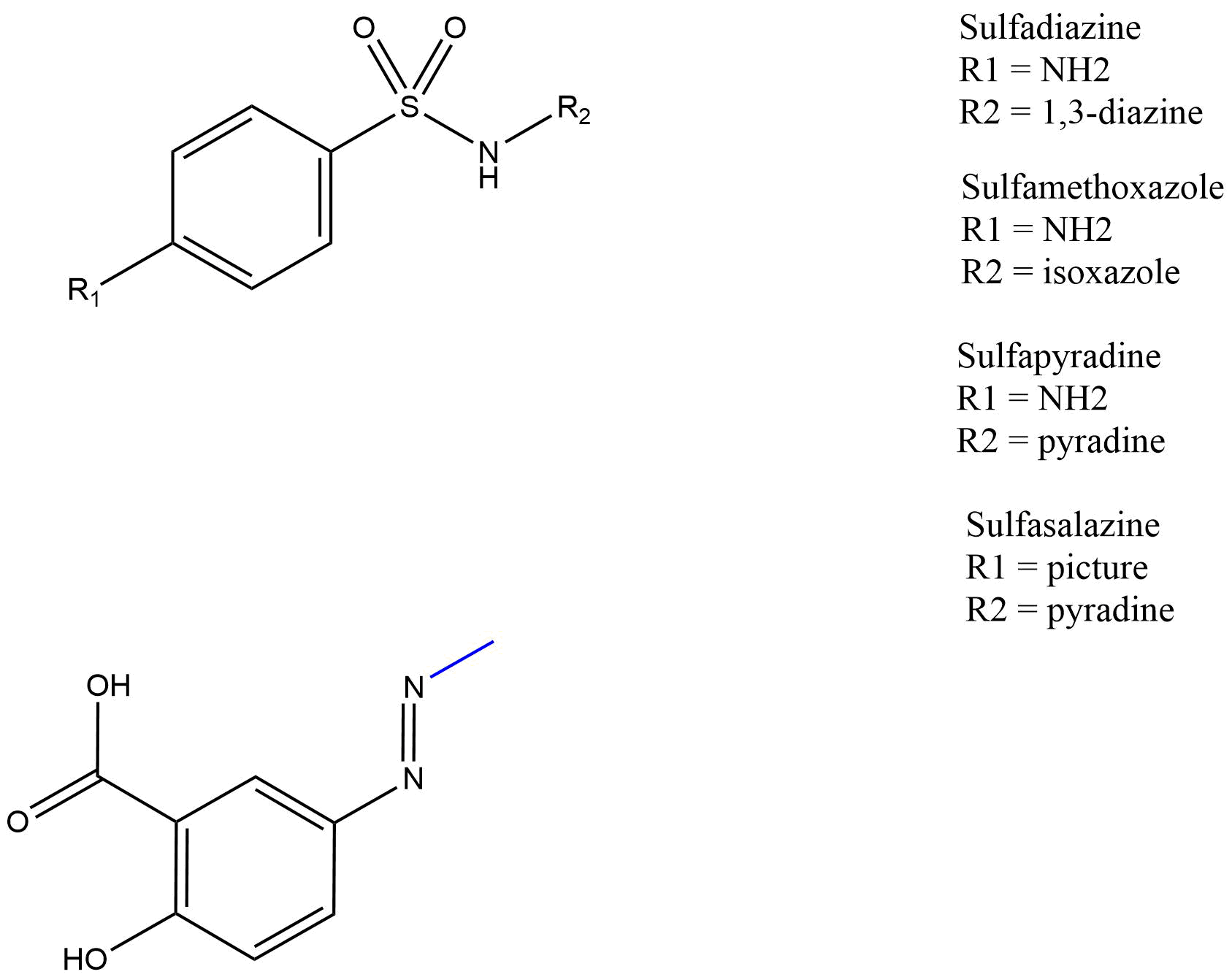 | Sulfamethoxazole | C_10_H_11_N_3_O_3_S | | | 253.0521 | | | NH_2_ | | | isoxazole | |  | | |  | | |  |  |  |
|  | Sulfapyridine | C_11_H_11_N_3_O_2_S | | | 249.0572 | | | NH_2_ | | | pyridine | |  | | |  | | |  |  |  |
|  | Sulfasalazine | C_18_H_14_N_4_O_5_S | | | 398.0685 | | | 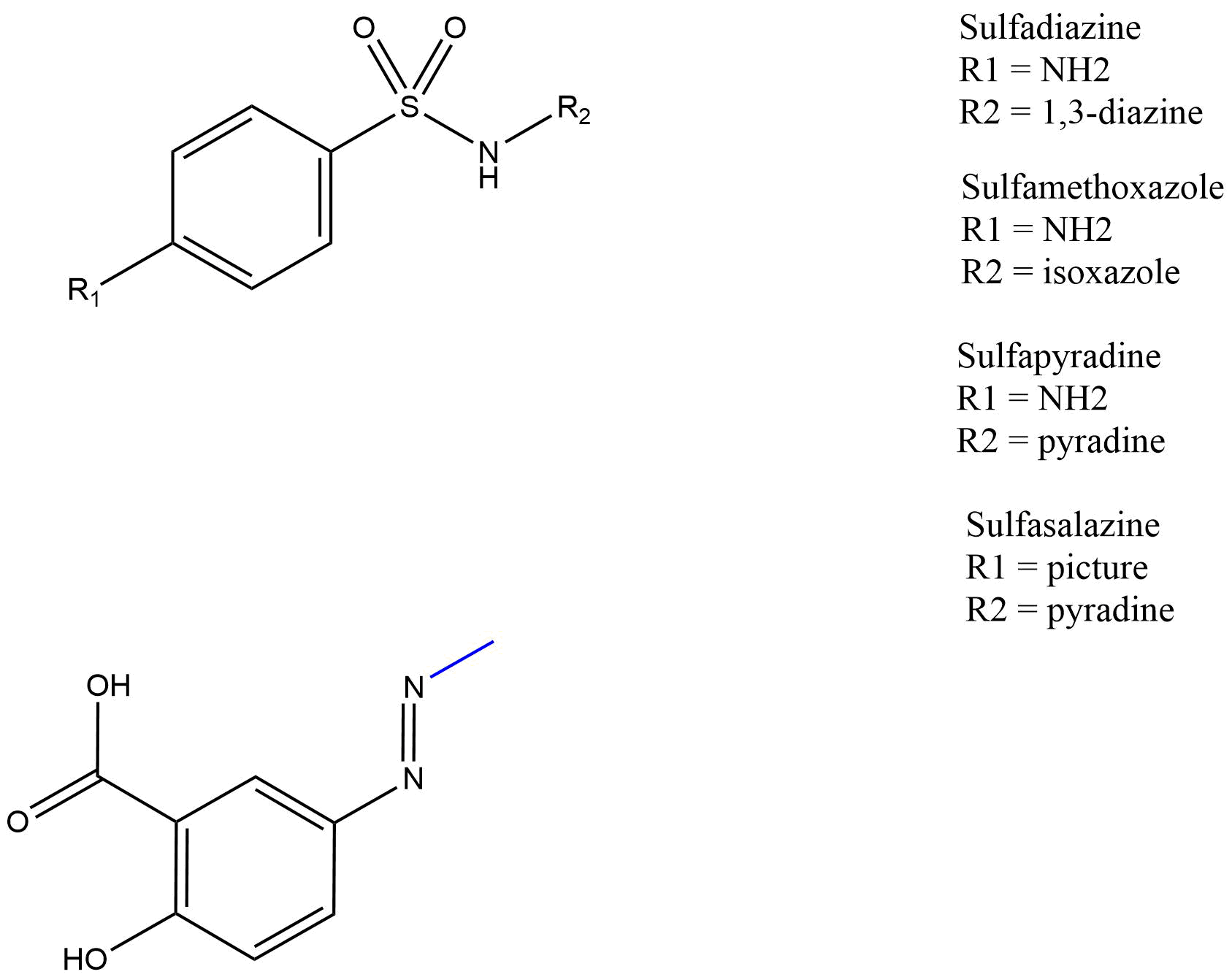 | | | pyridine | |  | | | **Prodrug** | | |  |  |  |
| SULFONAMIDE METABOLTES  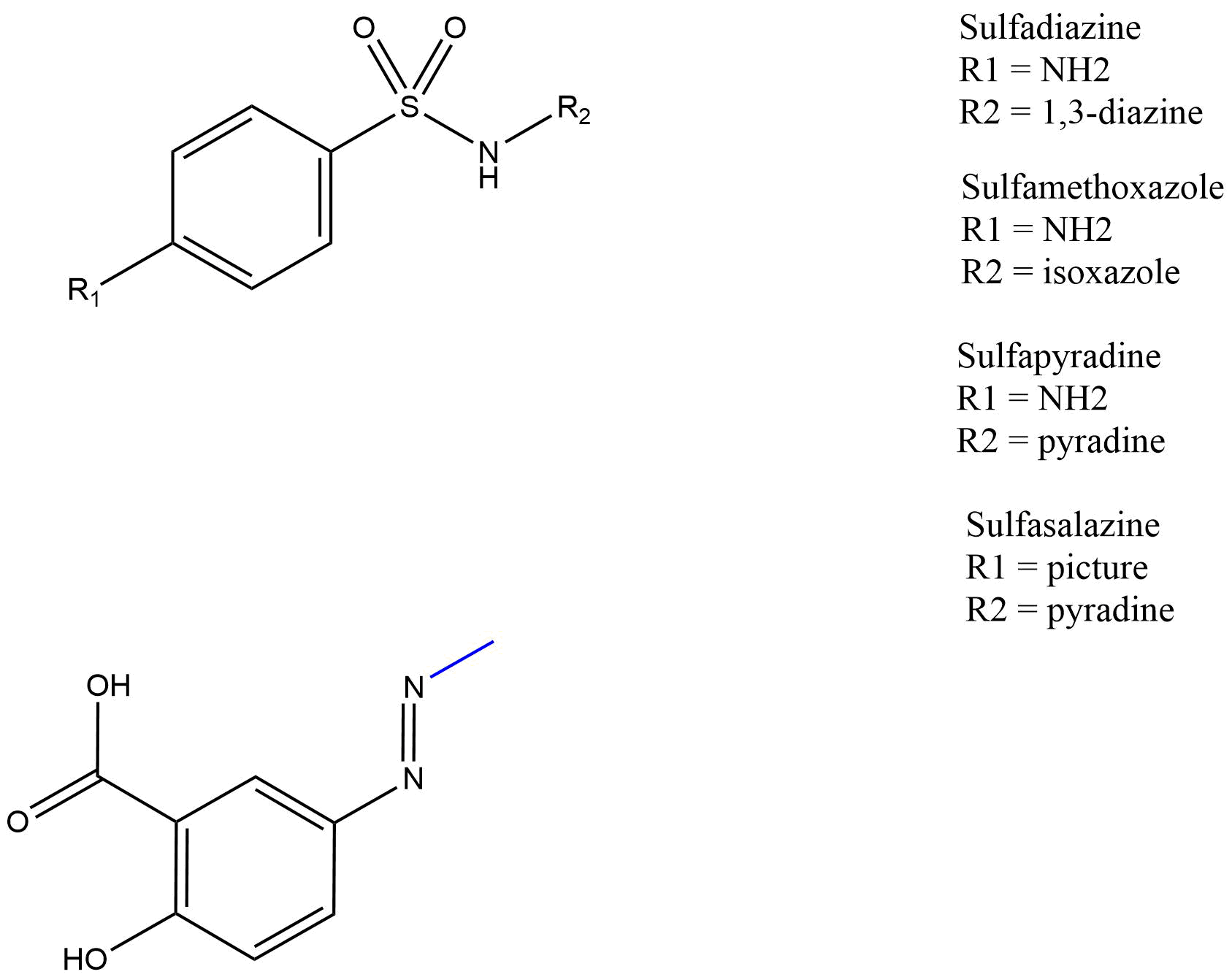 | Sulfadiazine  N-acetyl | C_12_H_12_N_4_O_3_S | | | 292.0630 | | | N-acetyl | | | *As sulfadiazine* | |  | | |  | | |  |  |  |
|  | Sulfamethoxazole N-acetyl | C_12_H_13_N_3_O_4_S | | | 295.0627 | | | N-acetyl | | | *As sulfamethoxazole* | |  | | |  | | |  |  |  |
|  | Sulfapyridine  N-acetyl | C_13_H_13_N_3_O_3_S | | | 291.0678 | | | N-acetyl | | | *As sulfapyridine* | |  | | |  | | |  |  |  |
|  | Sulfapyridine | C_11_H_11_N_3_O_2_S | | | 249.0572 | | | *As above* | | | *As above* | | **Sulfasalazine active metabolite** | | | | | | |  |  |
| TRIMETHOPRIM & METABOLITES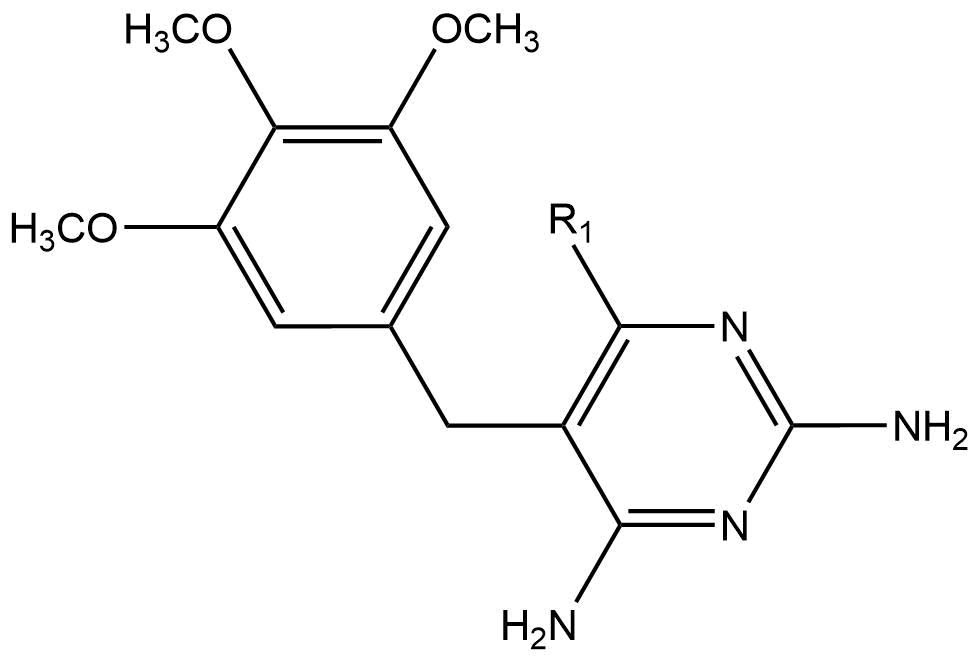 | Trimethoprim | C_14_H_18_N_4_O_3_ | | | 290.1379 | | | H | | |  | |  | | |  | | |  |  |  |
|  | Trimethoprim 4-hydroxy | C_14_H_18_N_4_O_4_ | | | 306.1328 | | | OH | | |  | |  | | |  | | |  |  |  |
|  |  |  | | |  | | |  | | |  | |  | | |  | | |  |  |  |

| Group | Compound | Formula | Monoisotopic mass | R_1_ | R_2_ | R_3_ | | R_4_ | |
| --- | --- | --- | --- | --- | --- | --- | --- | --- | --- |
| AMPHENICOLS  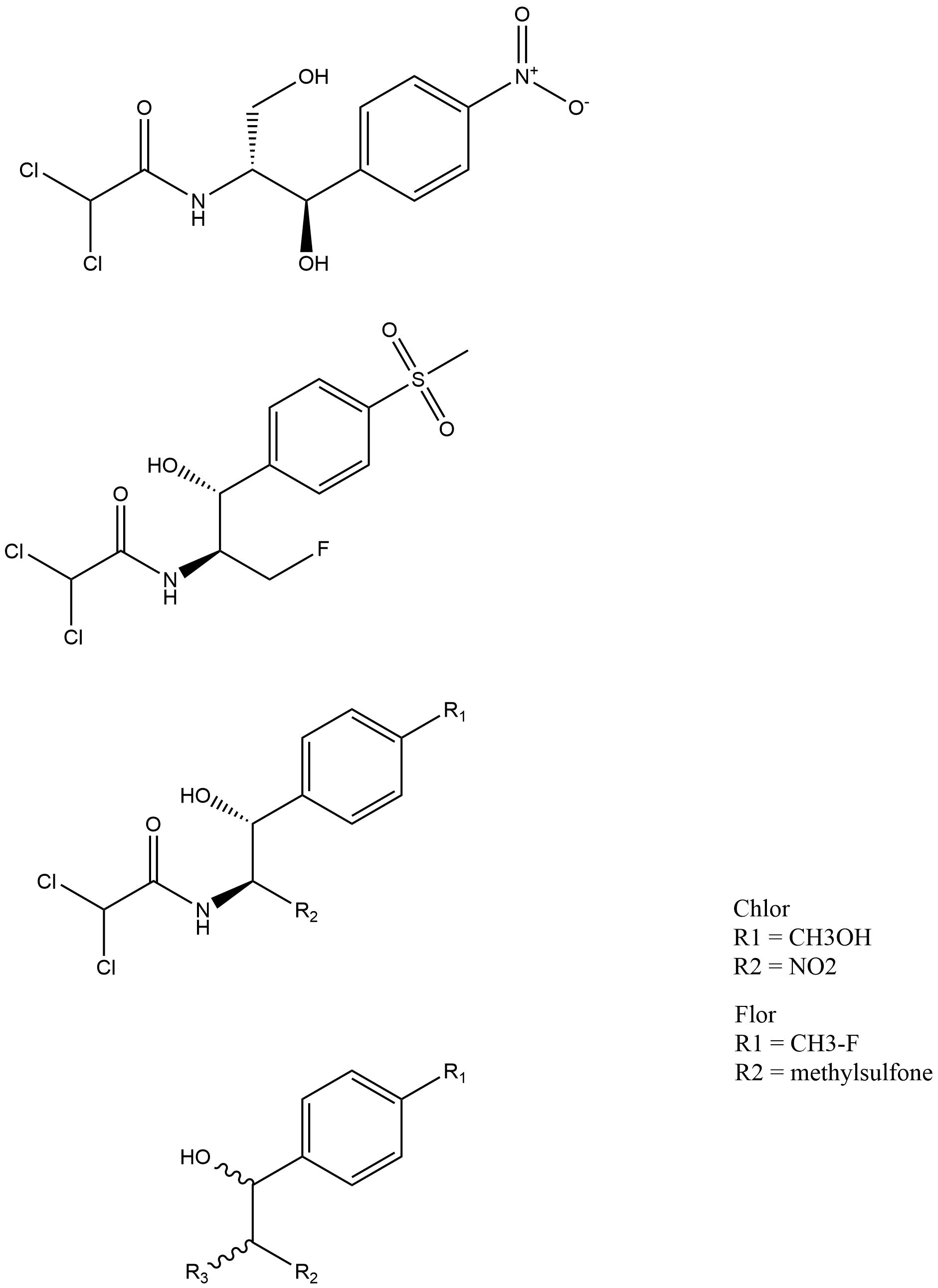 | Chloramphenicol | C_11_H_12_Cl_2_N_2_O_5_ | 322.0123 | NO_2_ | methanol |  | |  | |
|  | Florfenicol | C_12_H_14_Cl_2_FNO_4_S | 357.0005 | methyl sulfone | CH_3_F |  | |  | |
| AMPHENICOL METABOLITES  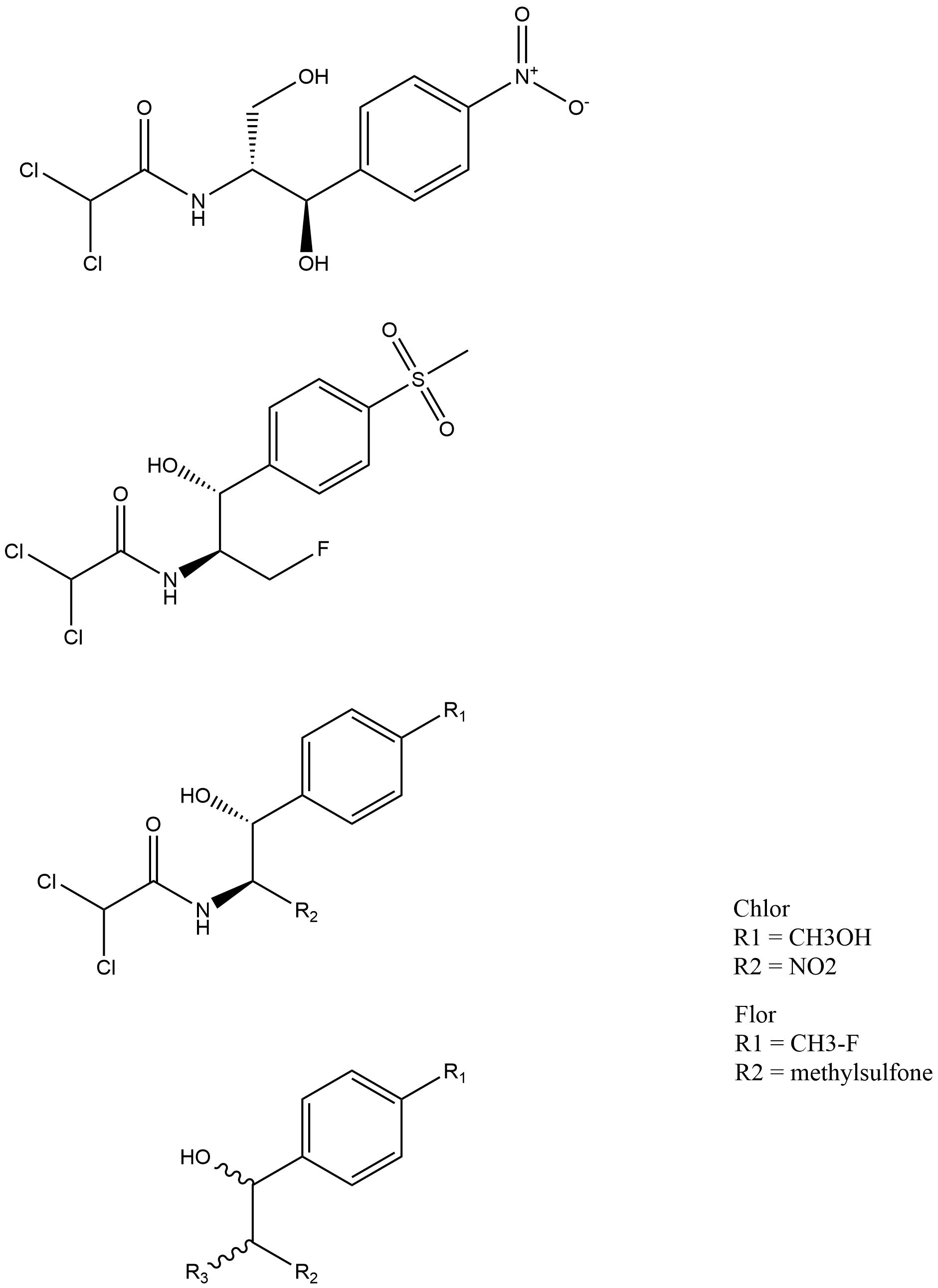 | S,S-ANP | C_9_H_12_N_2_O_4_ | 212.0797 | *As chloramphenicol* | *As chloramphenicol* | NH_2_ | |  | |
| ANTIRETROVIRALS  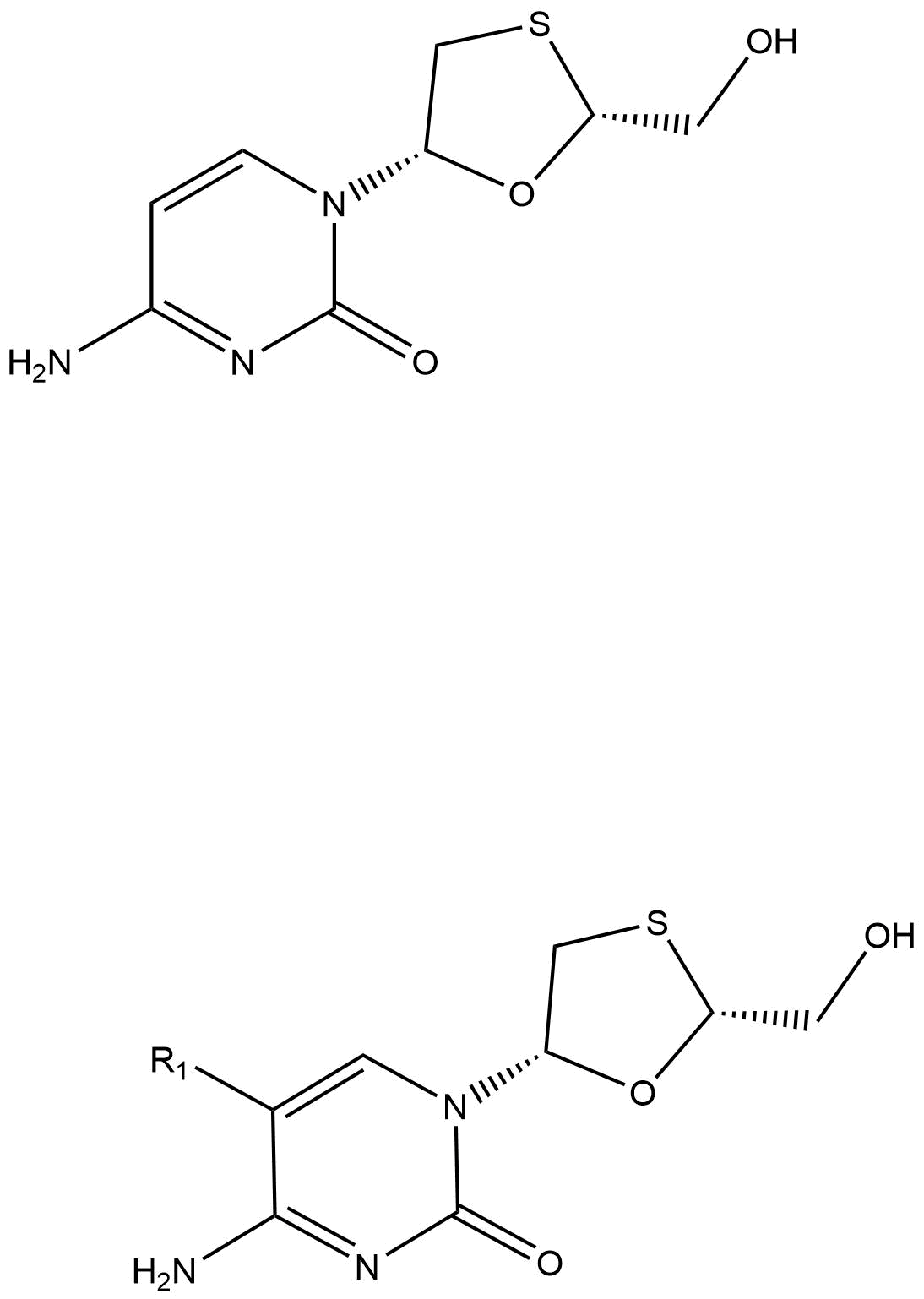 | Emtricitabine | C_8_H_10_FN_3_O_3_S | 247.0427 | F |  |  | |  | |
|  | Lamivudine | C_8_H_11_N_3_O_3_S | 229.0521 | H |  |  | |  | |
| POLYMYXINS & METABOLITES | Colistimethate ≠ | C_53_H_102_N_16_O_16_S | 1250.7380 | 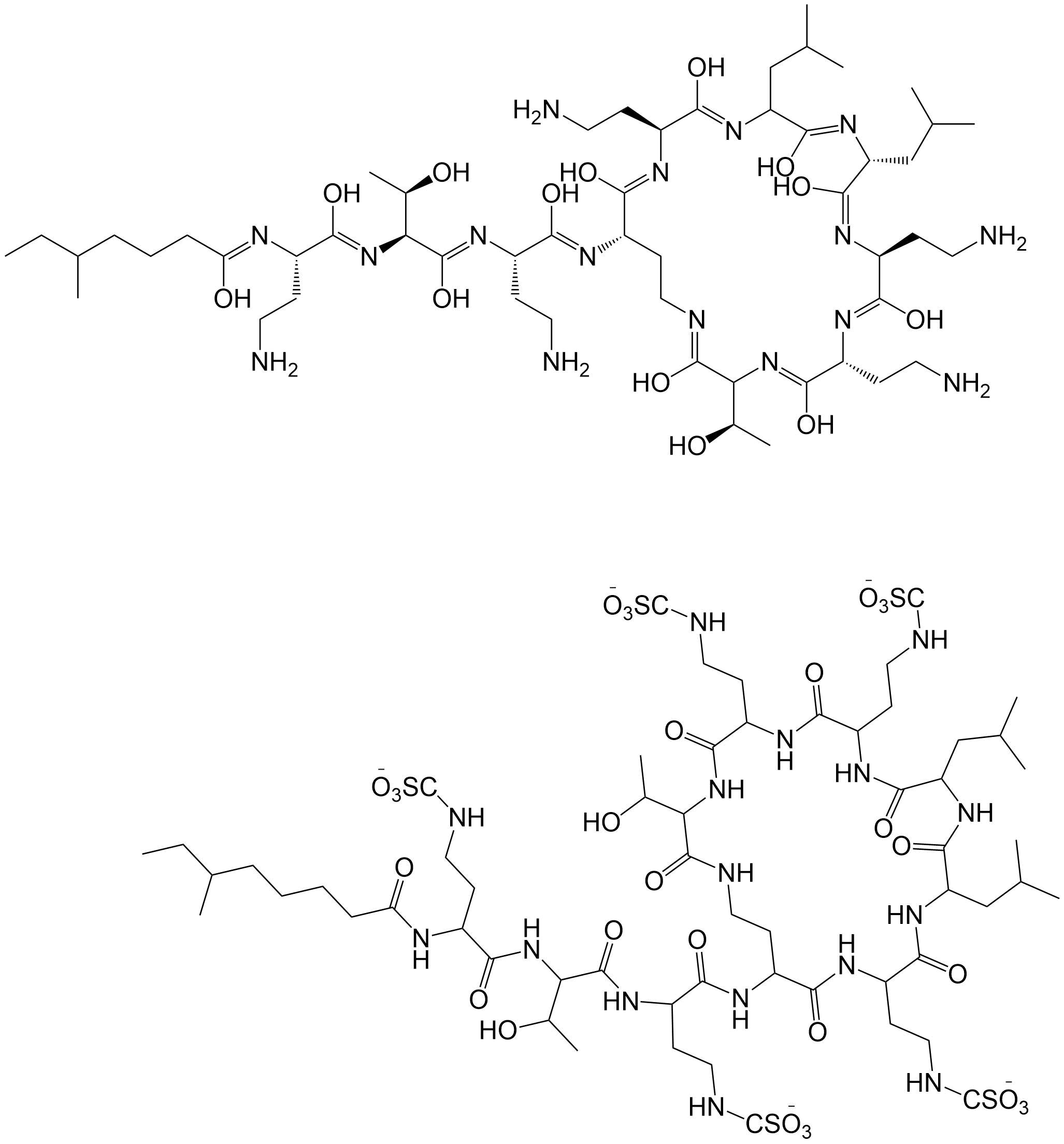 | | | **Prodrug** | |  |
|  | Colistin A ≠  Colistin B ≠ | C_53_H_100_N_16_O_13_  C_52_H_98_N_16_O_13_ | 1168.7656  1154.7499 | R_1_ = CH_3_  R_1_ = H | 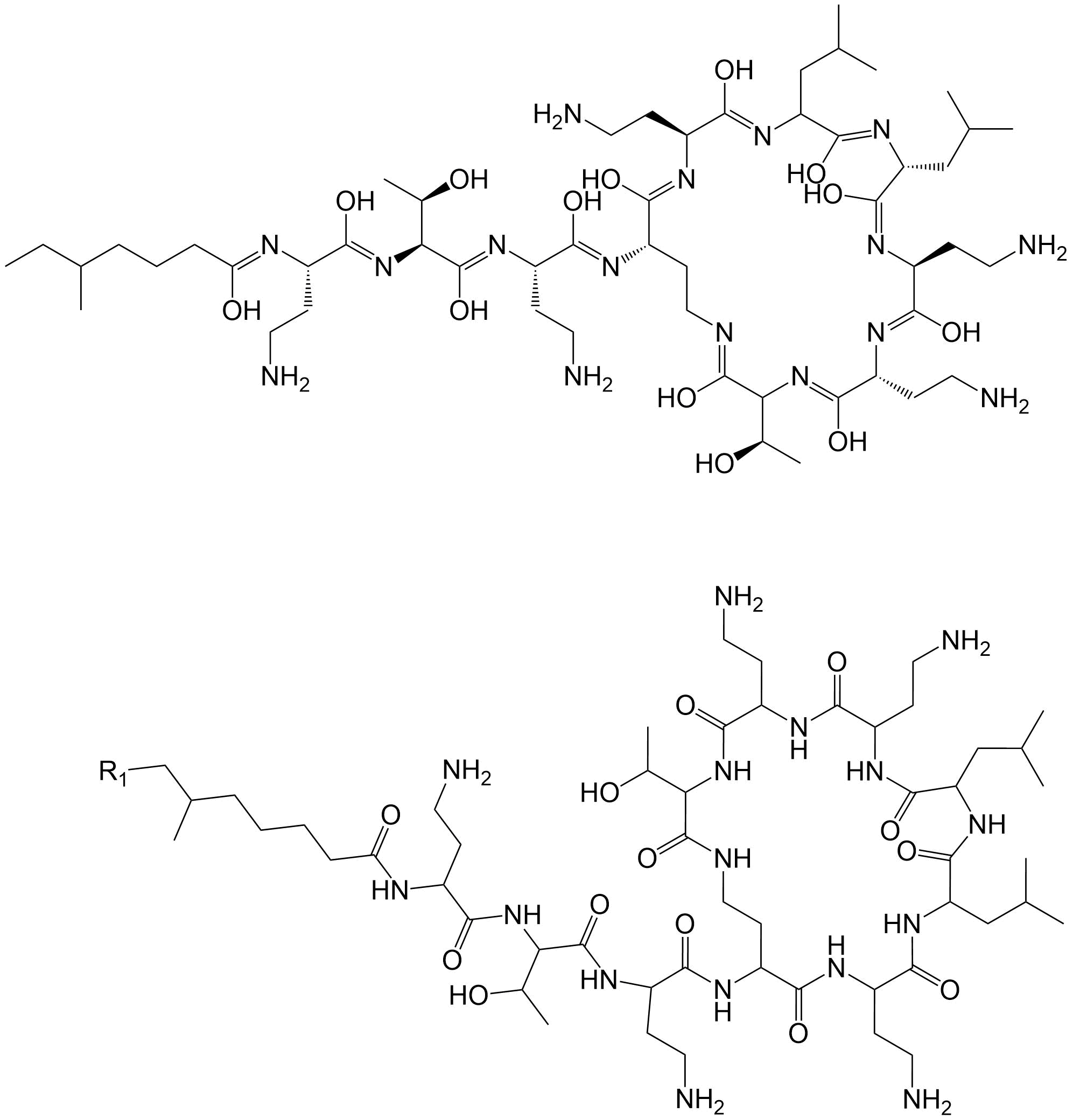 | | **Active metabolite** | |  |

| Group | Compound | Formula | Monoisotopic mass | Aglycone ring | Aglycone ring | Amino sugar (R_1_) | Sugar (R_2_) |
| --- | --- | --- | --- | --- | --- | --- | --- |
| MACROLIDES | Azithromycin A  Azithromycin B ¥ | C_38_H_72_N_2_O_12_  C_38_H_72_N_2_O_11_ | 748.5085  732.5136 | 15-membered  C_22_H_41_NO_5_ | 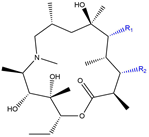 | 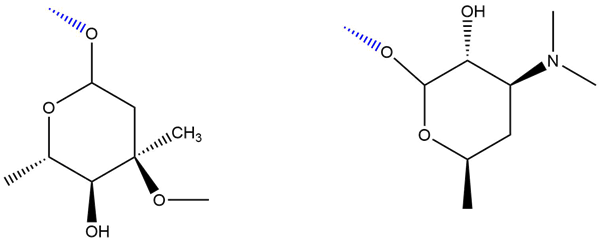 | 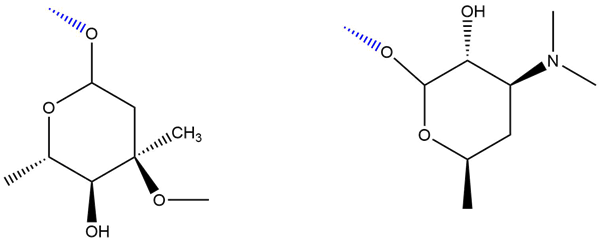 |
| 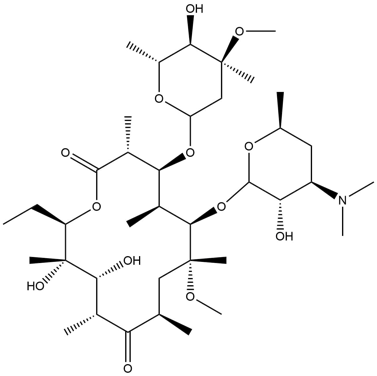*e.g. Clarithromycin* | Clarithromycin | C_38_H_69_NO_13_ | 747.4769 | 14-membered  C_22_H_38_O_6_ | 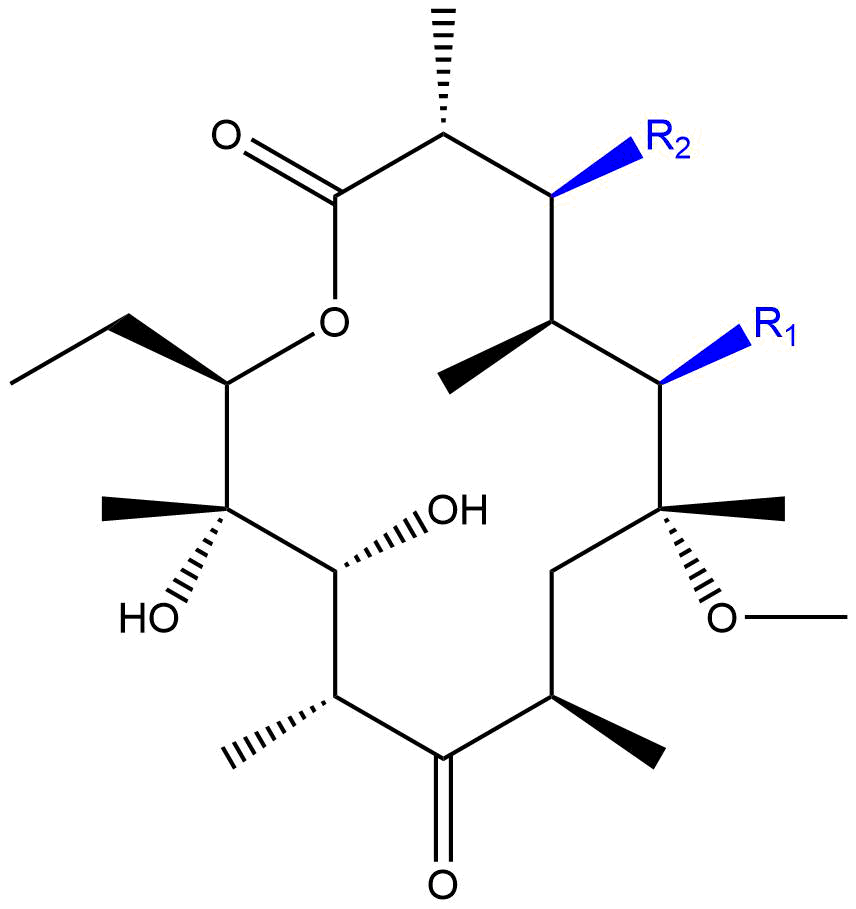 | 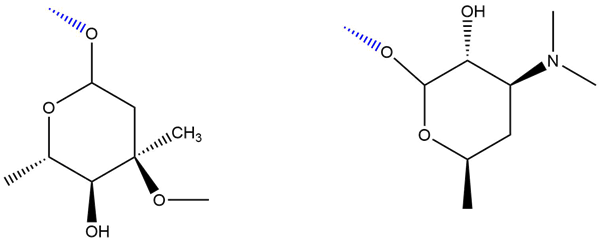 | 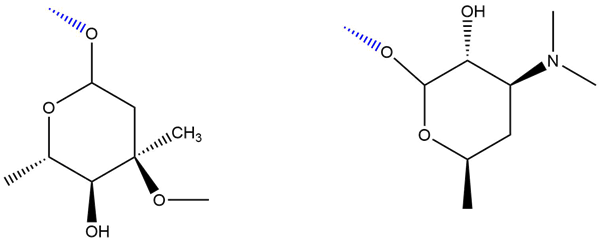 |
|  | Erythromycin A  Erythromycin B ¥  Erythromycin C ¥  Erythromycin D ¥ | C_37_H_67_NO_13_  C_37_H_67_NO_12_  C_36_H_65_NO_13_  C_36_H_65_NO_12_ | 733.4612  717.4663  719.4456  703.4507 | 14-membered  C_21_H_37_O_6_ | 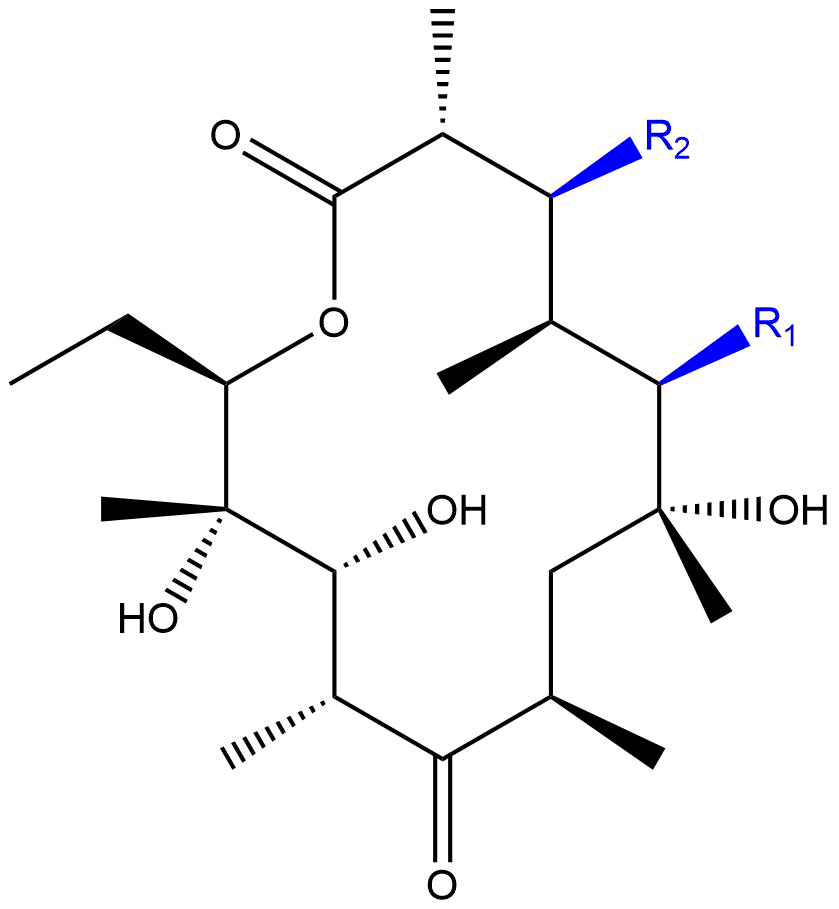 | 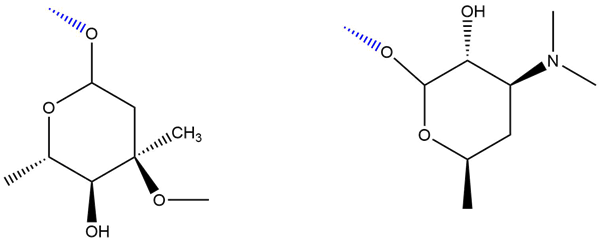 | 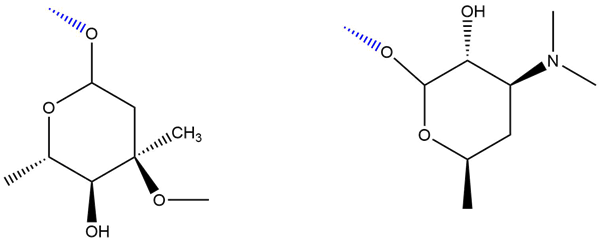 |
|  | Tylosin ≠ | C_46_H_77_NO_17_ | 915.5192 | 16-membered  C_23_H_34_O_5_ | 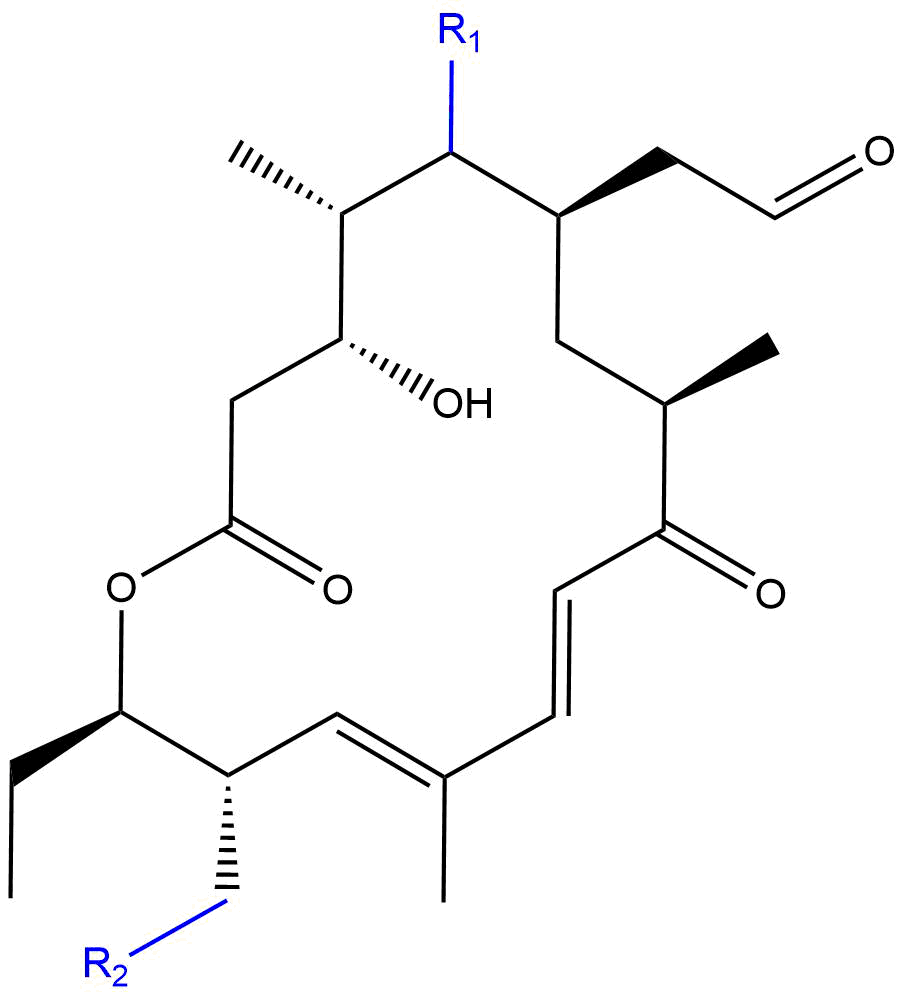 | 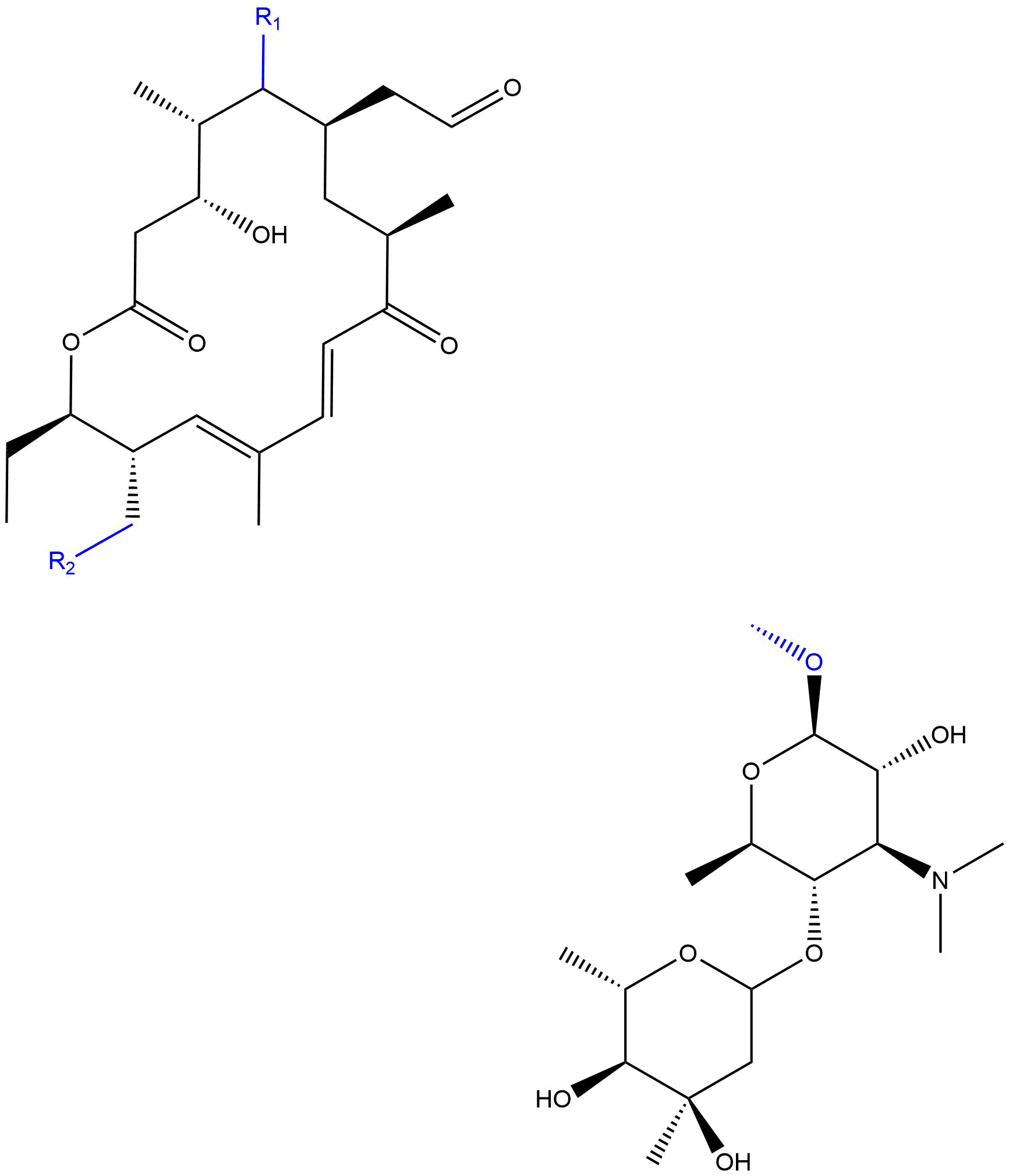 | 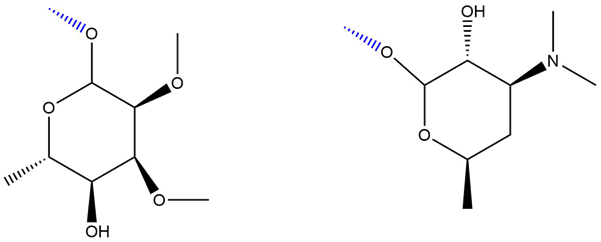 |
| MACROLIDE METABOLITES | Azithromycin A  N’-desmethyl | C_37_H_70_N_2_O_12_ | 734.4929 | *As azithromycin* | *As azithromycin* | N-desmethyl sugar | *As azithromycin* |
|  | Clarithromycin  N-desmethyl | C_37_H_67_NO_13_ | 733.4612 | *As clarithromycin* | *As clarithromycin* | N-desmethyl sugar | *As clarithromycin* |
|  | Erythromycin A  N-desmethyl | C_36_H_65_NO_13_ | 719.4456 | *As erythromycin* | *As erythromycin* | N-desmethyl sugar | *As erythromycin* |

| Group | Compound | Formula | Monoisotopic mass | R_1_ |  |  |  |
| --- | --- | --- | --- | --- | --- | --- | --- |
| LINCOSAMIDES & METABOLITES  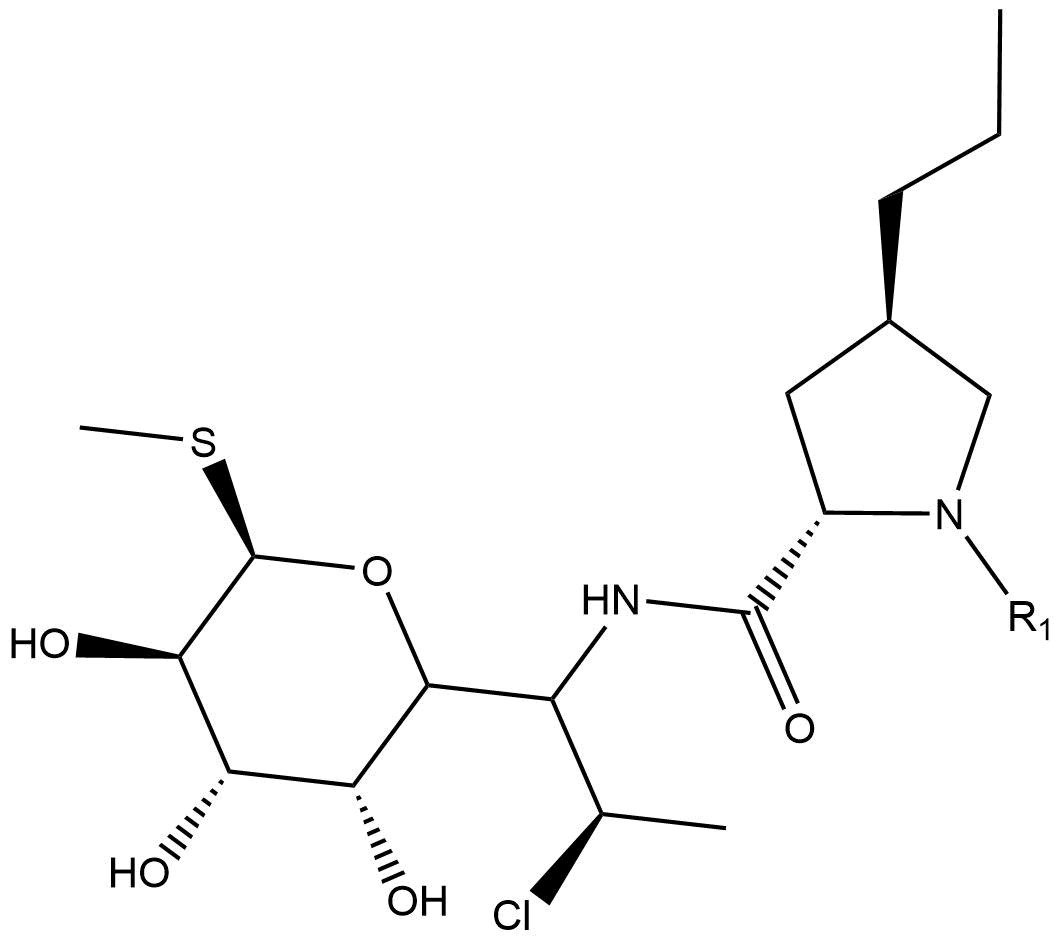 | Clindamycin A  Clindamycin B ¥ | C_18_H_33_ClN_2_O_5_S  C_17_H_31_ClN_2_O_5_S | 424.1799  410.1642 | CH_3_  H |  |  |  |
|  | Clindamycin A  N-desmethyl | C_17_H_31_ClN_2_O_5_S | 410.1642 | H |  |  |  |
| MONOBACTAMS  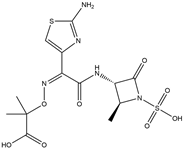 | Aztreonam | C_13_H_17_N_5_O_8_S_2_ | 435.0519 |  |  |  |  |
| CARBPENEMS  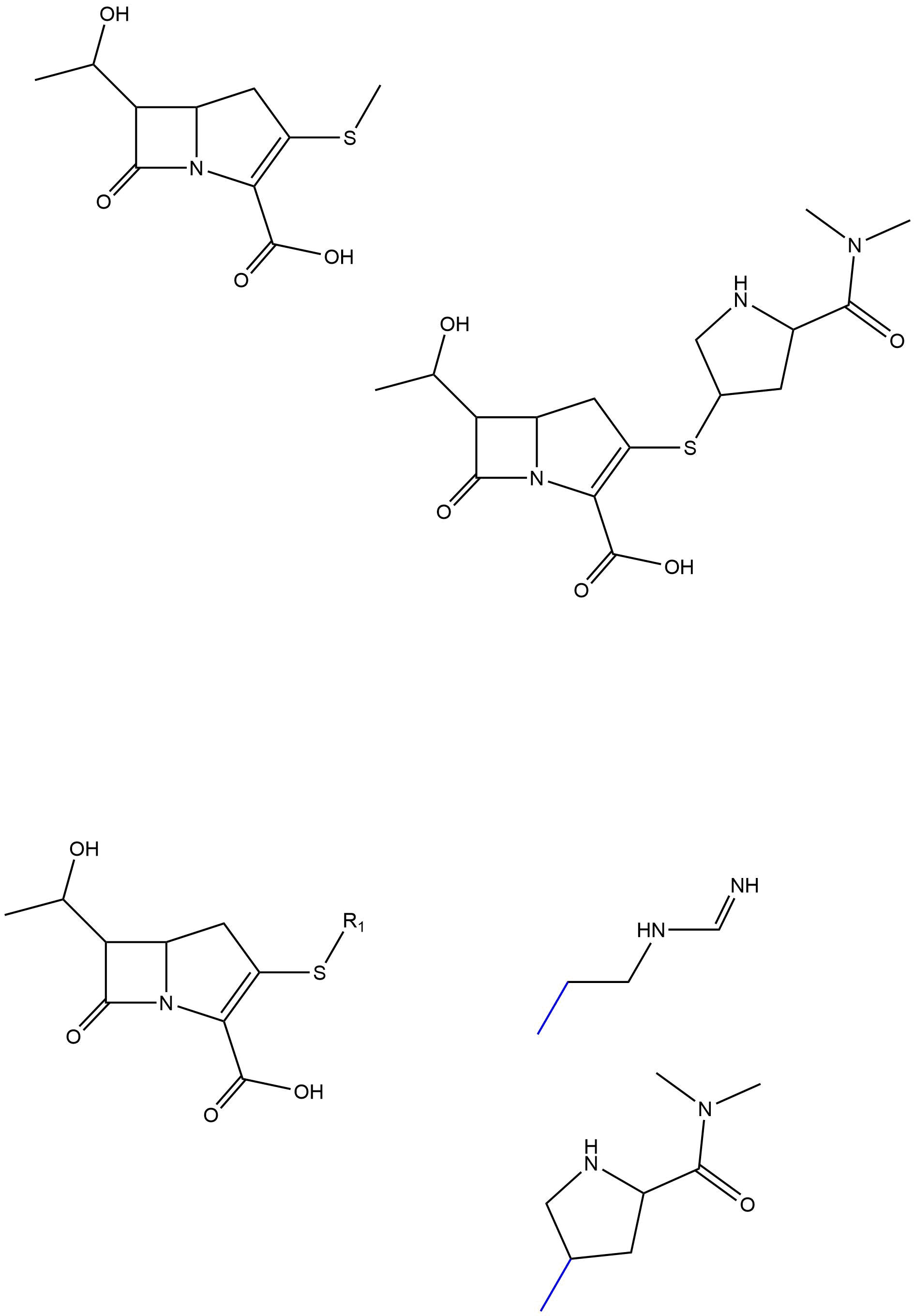 | Meropenem | C_17_H_25_N_3_O_5_S | 383.1515 | 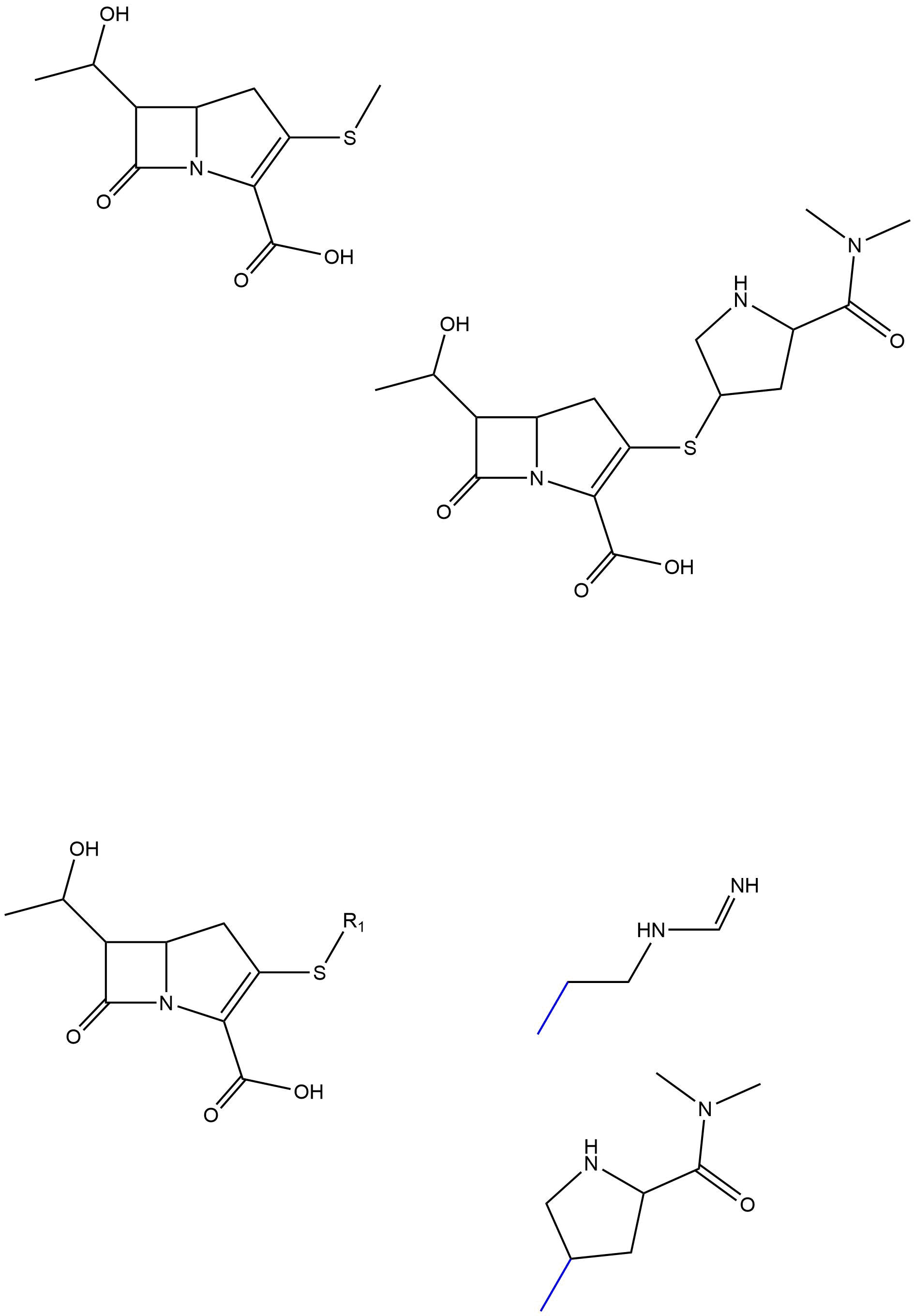 |  |  |  |
|  | Imipenem | C_12_H_17_N_3_O_4_S | 299.0940 | 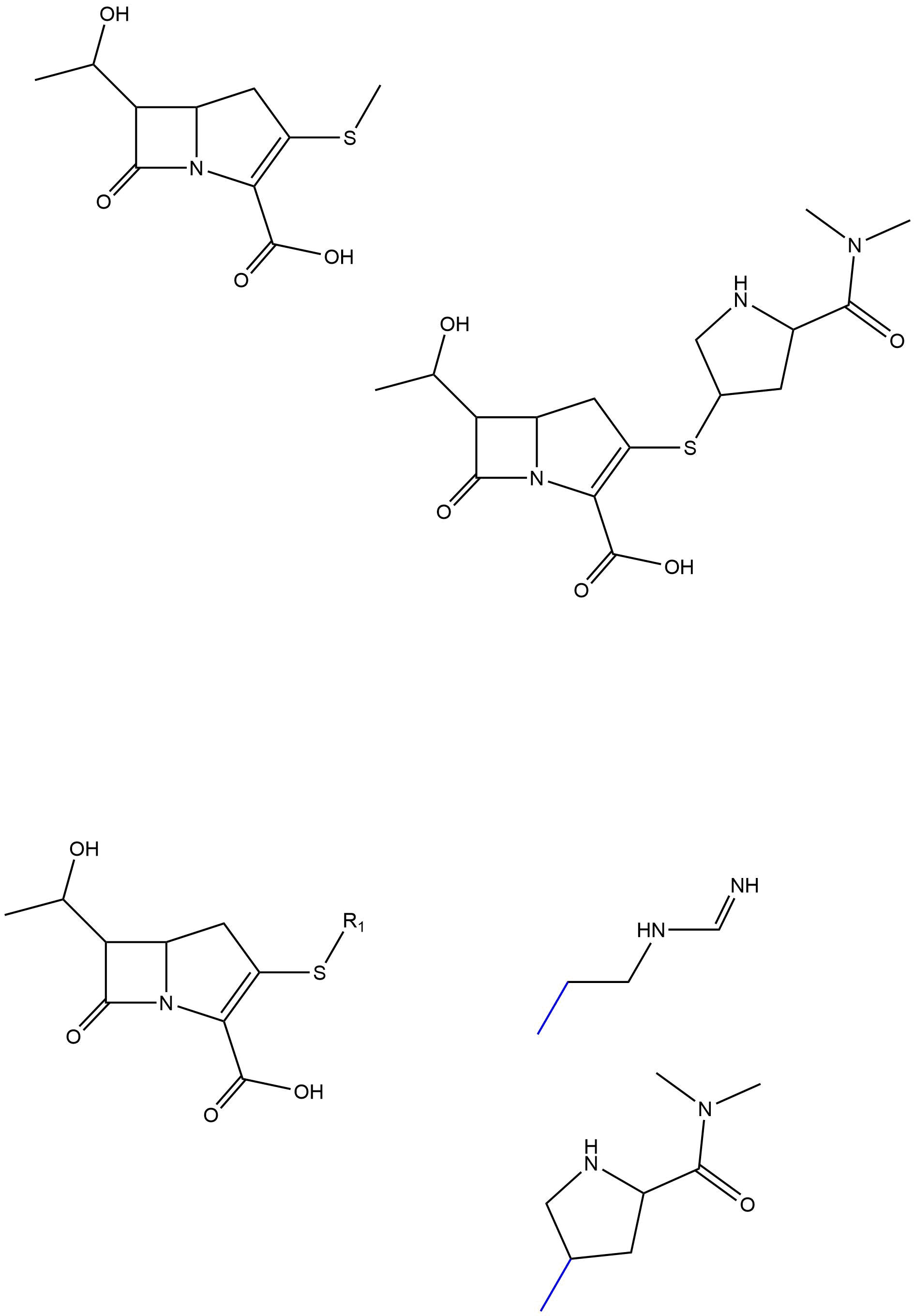 |  |  |  |
| THALIDOMIDE  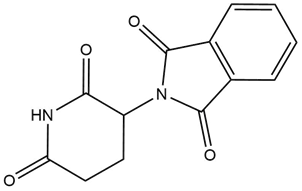 | Thalidomide | C_13_H_10_N_2_O_4_ | 258.0641 |  |  |  |  |

| **Group** | **Compound info** | **Group** | **Compound info** | **Group** | **Compound info** |
| --- | --- | --- | --- | --- | --- |
| **AMINOGLYCOSIDES** |  |  |  |  |  |
| 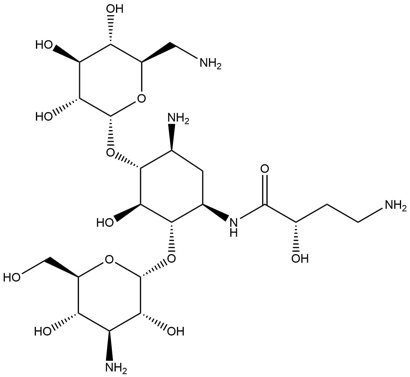 | Amikacin ≠  C_22_H_43_N_5_O_13_  M.M. = 585.2857 | 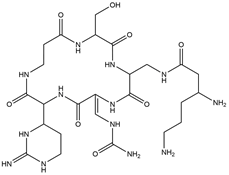 | Capreomycin Ia  C_25_H_44_N_14_O_8_  M.M. = 668.3467  Capreomycin Ib  C_25_H_44_N_14_O_7_  M.M. = 652.3517 | 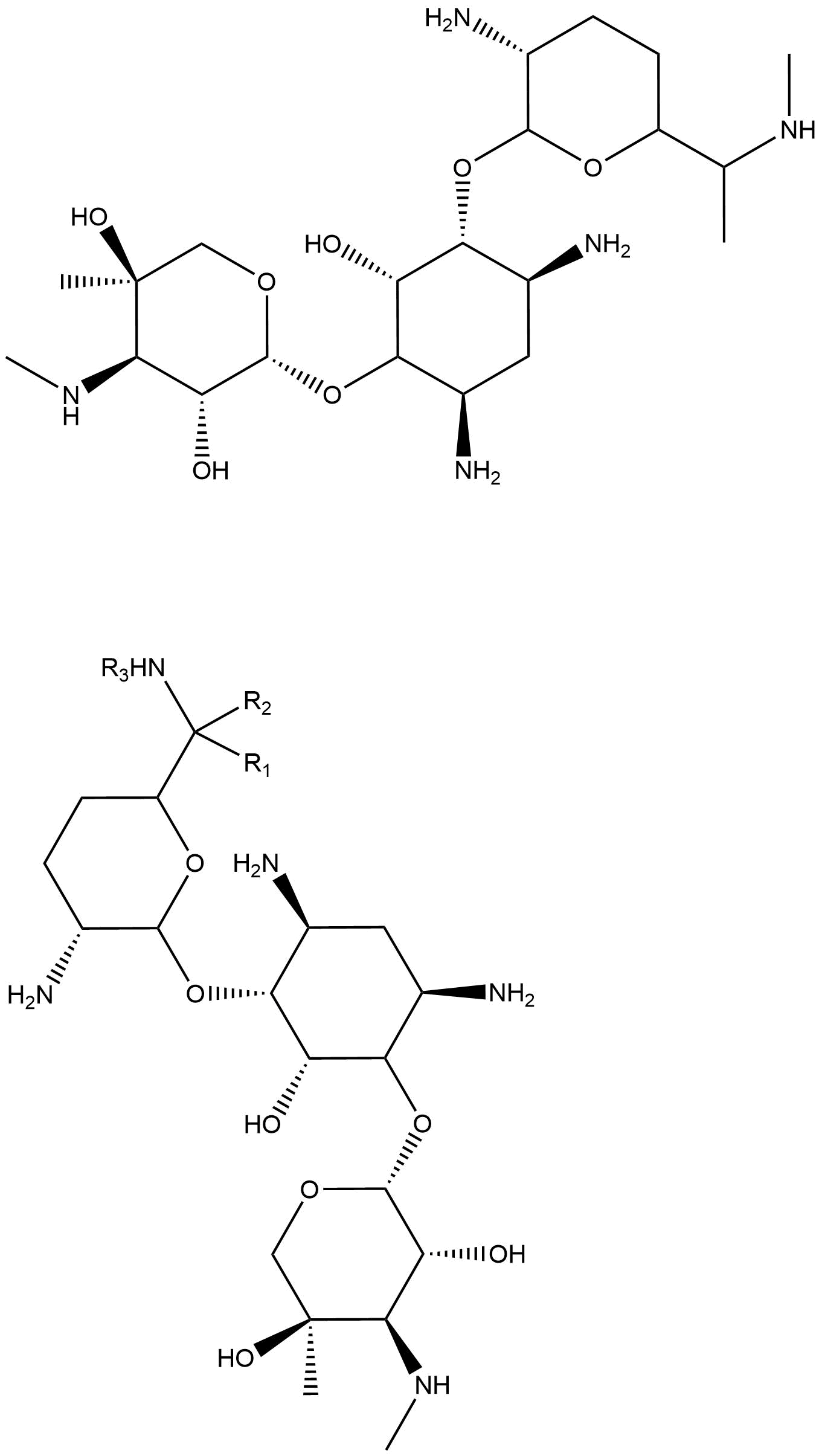 | Gentamicin C1  R_1_, R_2_, R_3_ = Me, H, Me  C_21_H_43_N_5_O_7_: 477.3162  Gentamicin C1a  R_1_, R_2_, R_3_ = H, H, H  C_19_H_39_N_5_O_7_: 449.2849  Gentamicin C2  R_1_, R_2_, R_3_ = Me, H, H  C_20_H_41_N_5_O_7_: 463.3006  Gentamicin C2a  R_1_, R_2_, R_3_ = H, Me, H  C_20_H_41_N_5_O_7_: 463.3006  Gentamicin C2b  R_1_, R_2_, R_3_ = H, H, Me  C_20_H_41_N_5_O_7_: 463.3006 |
| 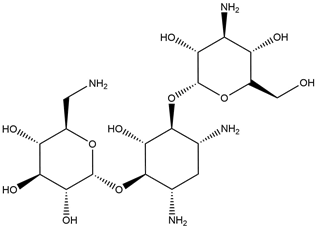 | Kanamycin A (&C)  C_18_H_36_N_4_O_11_  M.M. = 484.2381  B: C_18_H_37_N_5_O_10_ ¥  M.M. = 483.2540  D&X: C_18_H_35_N_3_O_12_ ¥  M.M. = 485.2221 | 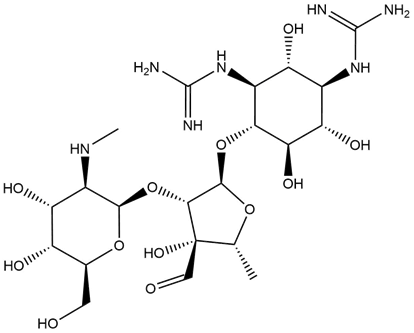 | Streptomycin A  C_21_H_39_N_7_O_12_  M.M. = 581.2657  Streptomycin B ¥  C_27_H_49_N_7_O_17_  M.M. = 743.3185 |  |  |
| **TUBERCULOSIS DRUGS** |  |  |  |  |  |
| 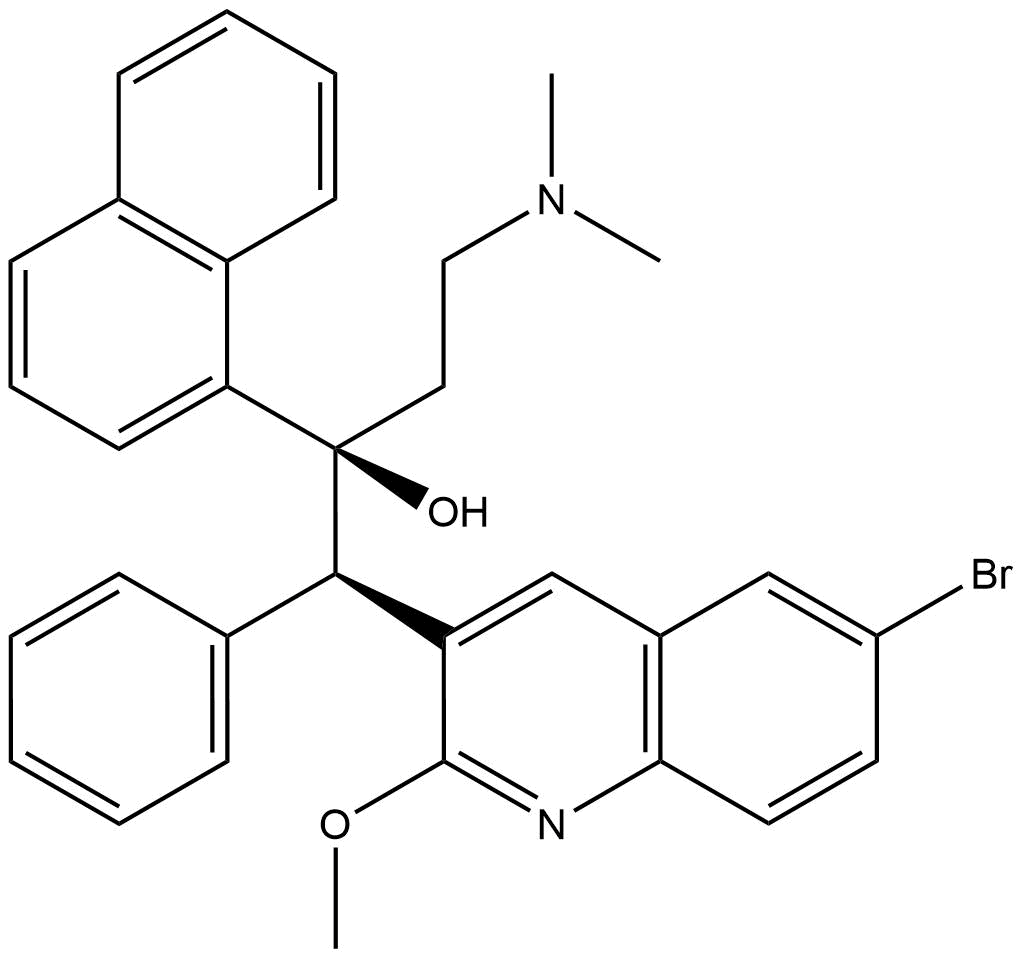 | Bedaquiline  C_32_H_31_BrN_2_O_2_  M.M. = 554.1569 | 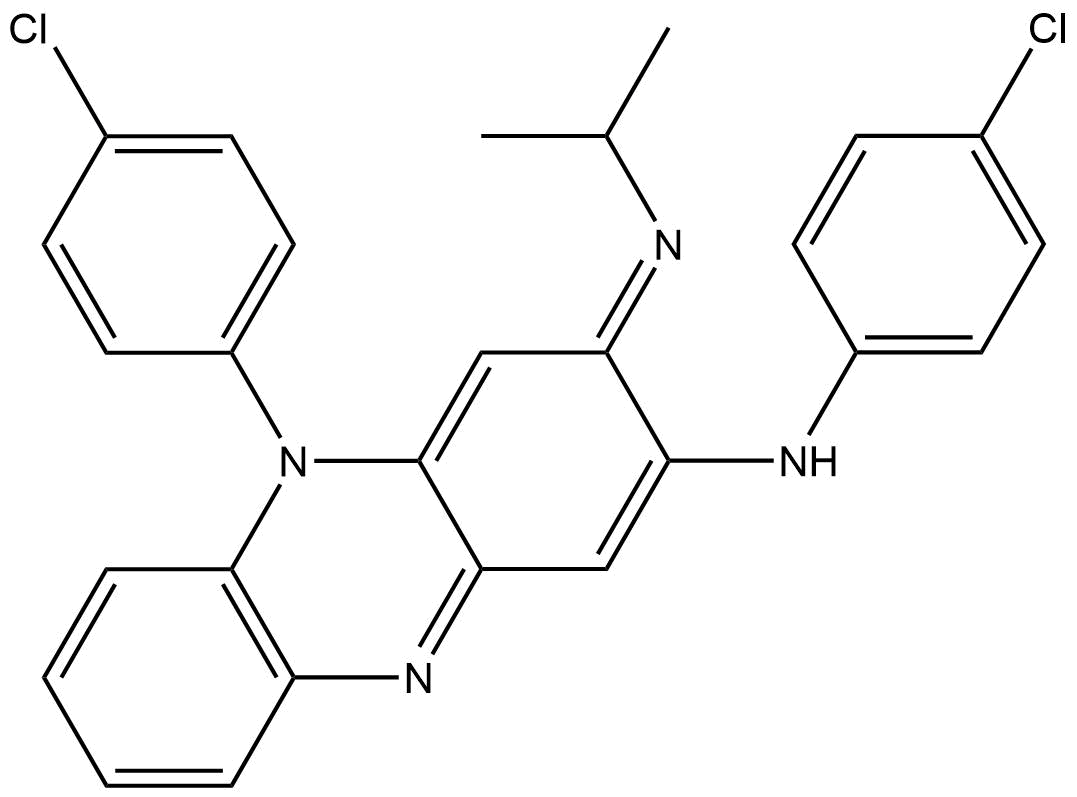 | Clofazimine ≠  C_27_H_22_Cl_2_N_4_  M.M. = 472.1222  **Prodrug** |   Delamanid  C_25_H_25_F_3_N_4_O_6_  M.M. = 534.1726 | |
| 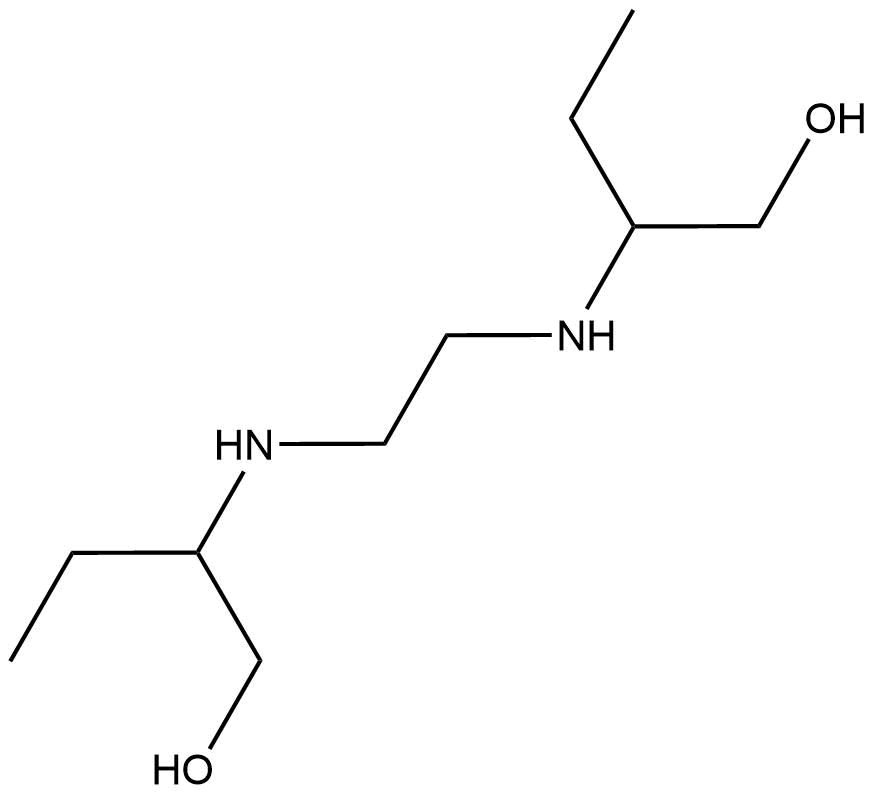 | Ethambutol  C_10_H_24_N_2_O_2_  M.M. = 204.1838 | 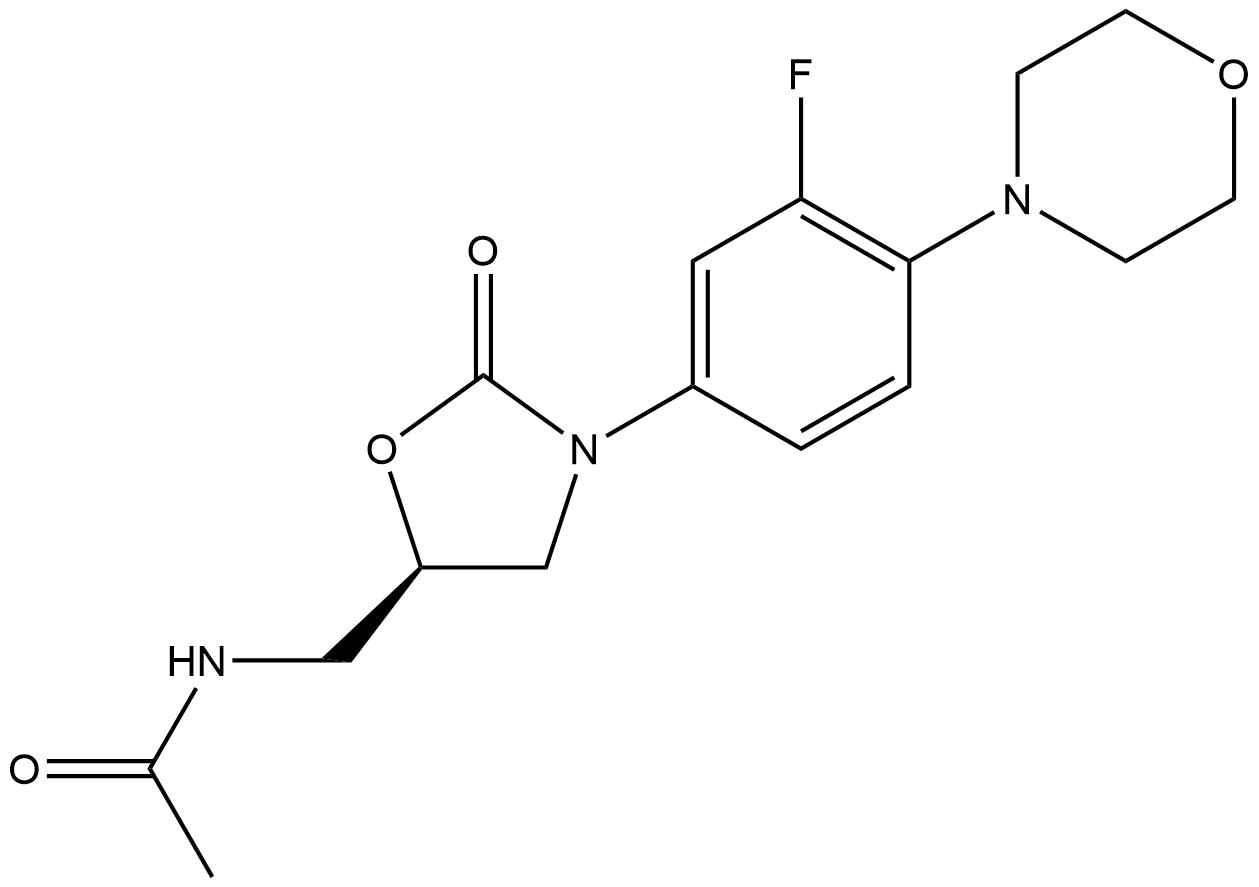 | Linezolid  C_16_H_20_FN_3_O_4_  M.M. = 337.1438 |  |  |

≠ excluded from method; ¥ compound form not analysed, but accounted for in calculations (compound structures display major form only)

Ph = phenyl group; M.M. = Monoisotopic mass; ANP = 2-Amino-1-(4-nitrophenyl)-1,3-propanediol; NP-AHD = 1-[[(2-Nitrophenyl)methylene]amino]-2,4-imidazolidinedione

….
